# Four‐Component Strain‐Release‐Driven Synthesis of Functionalized Azetidines

**DOI:** 10.1002/anie.202214049

**Published:** 2022-11-27

**Authors:** Jasper L. Tyler, Adam Noble, Varinder K. Aggarwal

**Affiliations:** ^1^ School of Chemistry University of Bristol Cantock's Close Bristol BS8 1TS UK

**Keywords:** Azetidines, Bicyclic Compounds, Heterocycles, Multicomponent Reaction, Strained Molecules

## Abstract

Despite the favorable properties that azetidine rings can engender on drug‐compounds, methods for the diversity‐oriented synthesis of azetidine‐based structures are significantly underdeveloped. Herein, we report the successful realization of a multicomponent [1,2]‐Brook rearrangement/strain‐release‐driven anion relay sequence and its application to the modular synthesis of substituted azetidines. The rapidity of the reaction, as confirmed by in situ infra‐red spectroscopy, leverages the strain‐release ring‐opening of azabicyclo[1.1.0]butane to drive the equilibrium of the Brook rearrangement. The three electrophilic coupling partners, added sequentially to azabicyclo[1.1.0]butyl‐lithium, could be individually varied to access a diverse compound library. The utility of this methodology was demonstrated in a 4‐step synthesis of the EP2 receptor antagonist PF‐04418948.

## Graphical abstract

A multicomponent [1,2]‐Brook rearrangement/strain‐release‐driven anion relay sequence and its application to the modular synthesis of substituted azetidines is described. Three electrophilic coupling partners, whose identities can be individually varied, were added sequentially to the highly strained fragment azabicyclo[1.1.0]butyl‐lithium to access a diverse compound library.

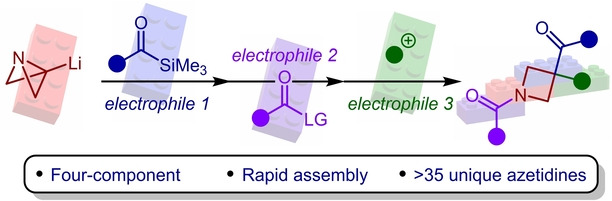

Saturated N‐heterocycles are considered as one of the most important building blocks in medicinal chemistry, highlighted by the prevalence of piperidine, piperazine and pyrrolidine rings in pharmaceuticals.^[1]^ Moving beyond this densely populated region of chemical space, the analogous four‐membered N‐heterocycle, azetidine, has been recognized as possessing a range of desirable characteristics, such as structural rigidity, improved solubility and resistance to metabolic degradation, which greatly improve the chances of clinical success compared to larger ring analogues.[^2^, ^3^] Despite the favorable properties that the inclusion of an azetidine ring can engender on drug‐compounds, methods for the modular and divergent synthesis of azetidine‐based compounds are significantly underdeveloped.[^4^, ^5^] In 2012, Marcaurelle and co‐workers reported, to the authors’ knowledge, the only azetidine‐focused diversity‐oriented library synthesis which was used for the identification of novel central nervous system (CNS) targeting pharmaceuticals.^[6]^ This protocol centered on the elaboration of a preassembled azetidine core, accessed in 5 steps from β‐amino alcohols, and required a further 6–9 steps to generate the spirocyclic scaffold applied to a solid‐phase library synthesis (Figure 1ai). Evidently, more efficient strategies to enable rapid access to these privileged building blocks are required.^[7]^

**Figure 1:**
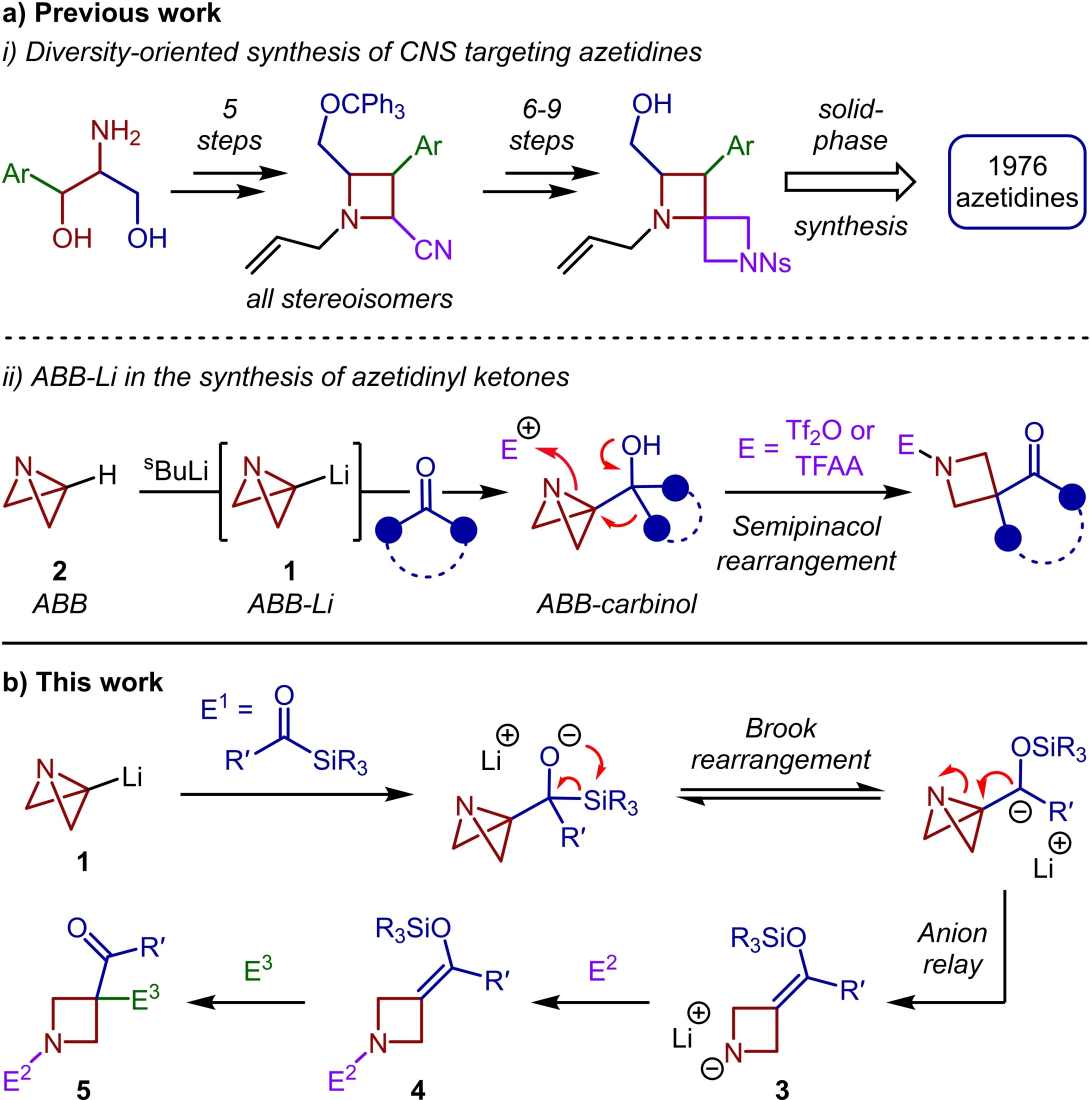
a) Previous work: (i) Diversity‐oriented synthesis of CNS targeting azetidines, (ii) ABB‐Li in the synthesis of acyl‐azetidines. b) This work: Four‐component synthesis of functionalized azetidines.

We aimed to establish a unique multicomponent protocol that would allow the expeditious assembly of functionalized azetidines and address the lack of modularity in traditional four‐membered ring construction.[^4^, ^6^] Our previous strategy towards this objective involved the application of the highly versatile and strained species azabicyclo[1.1.0]butyl‐lithium (ABB‐Li, **1**)^[8]^ which can be readily accessed through the deprotonation of azabicyclo[1.1.0]butane (Figure 1aii, **2**).[^9^, ^10^] Coupling of this carbenoid with ketones provided azabicyclo[1.1.0]butyl carbinols which, upon electrophilic activation, could undergo strain‐release‐driven semipinacol rearrangements to access acyl‐azetidines.^[11]^ While representing a modular synthesis, the identity of the electrophile is dictated by its ability to activate the azabicycle, making the choice of ketone the sole point of variation.

We envisaged a multicomponent approach to access a diverse library of 1,3,3‐trisubstituted azetidines by exploiting the inimitable reactivity of ABB‐Li through its coupling with acyl silanes (Figure 1b, E^1^). The alkoxide intermediate resulting from this 1,2‐addition was predicted to undergo a [1,2]‐Brook rearrangement to generate a carbanion that should instantly collapse to open the central bond of the azabicyclo[1.1.0]butane (ABB) fragment.^[12]^ This ring opening is predicted to possess a low kinetic barrier and a strong thermodynamic driving force to favour the azetidine products, in line with the recent report from Anderson and Duarte.^[13]^ The anion relay should also drive the equilibrium of the Brook rearrangement toward the carbanion, which traditionally requires the presence of anion stabilizing groups.^[14]^ Lithium amide **3** then offers the potential to react with an electrophile (E^2^) at the nitrogen atom, and with a further carbon or heteroatom‐based electrophile (E^3^) at the newly installed silyl enol ether, to give an overall four‐component synthesis of azetidines (**5**). Such a procedure provides the potential to access complementary products to those accessible through the nucleophilic ring opening of 3‐substituted ABB compounds.[^10^, ^15^] Herein, we report the successful realization of this multicomponent [1,2]‐Brook rearrangement/strain‐release‐driven anion relay sequence and its application in the rapid and modular synthesis of substituted azetidines. The identity of the three electrophilic coupling partners (E^1^, E^2^ and E^3^) could be individually varied to access a diverse compound library and, in the case of E^3^, could be extended to nucleophiles via oxidative coupling with silyl enol ether **4**.

We began by investigating the synthesis of silyl enol ether **4 a** in isolation (Table 1). To achieve this, ABB‐Li (**1**) was synthesized according to previous reports from dibromo‐amine **6** and TMEDA‐ligated ^s^BuLi.[^8c^, ^9a^] Acetyltrimethylsilane (**7 a**) was then added directly to **1**, which, upon nucleophilic addition, could participate in the desired [1,2]‐Brook rearrangement/strain‐release‐driven anion relay sequence. Lastly, di‐*tert*‐butyl dicarbonate (Boc_2_O) was employed to functionalize intermediate **3 a** and provide silyl enol ether **4 a** in a single step. Although the desired product could be generated in synthetically useful amounts (Table 1, entry 1), the yield of the reaction could not be reliably reproduced (Table S1). It was discovered that the observed irreproducibility arose during the highly exothermic reaction between lithium amide **3** and Boc_2_O which gave rise to variable degrees of product decomposition. However, this issue could be overcome by first protonating **3** with ^t^BuOH, which allowed access to **4 a** in a reproducible yield of up to 50 % (Table 1, entries 2–4). Using **7 a** as the limiting reagent gave an improved yield of 64 % as it was discovered that excess acyl silane significantly reduced product formation (Table 1, entries 5–7). Finally, the synthesis of the analogous triethylsilyl (TES) and *tert*‐butyldimethylsilyl (TBS) products demonstrated that the improved stability gained from using more hindered substrates comes at the cost of a decrease in the yield of **4 a** (Table 1, entries 8, 9). In line with previous reports, attempts to generate **4 a** via the enolization of the corresponding 3‐acetylazetidine resulted in the selective formation of the terminal silyl enol ether, highlighting the benefits of this unique protocol (see Supporting Information).^[16]^

**Table 1:** Optimization studies.

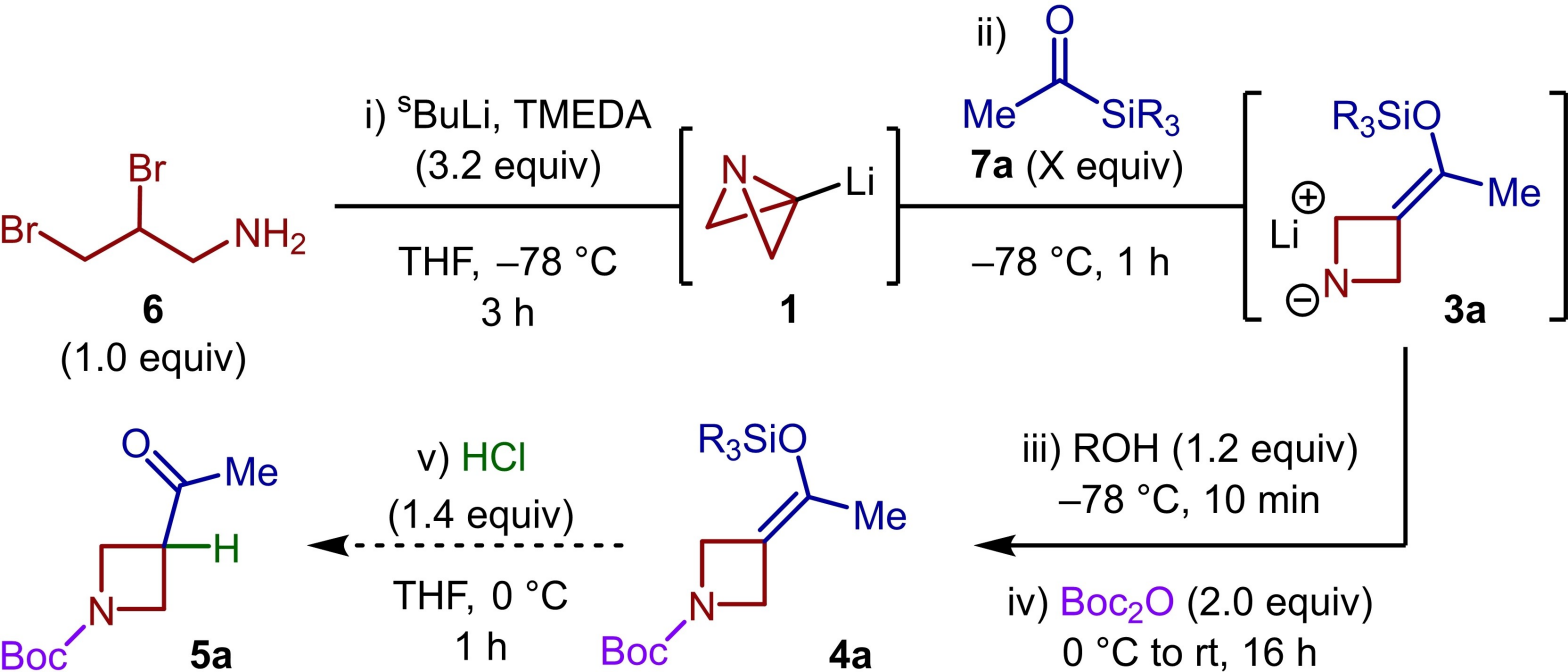				
Entry	SiR_3_	**7 a**; X equiv	ROH	**4 a**; % Yield^[a]^
1	TMS (**7 a**)	1.2	–	19–45^[b]^
2	TMS (**7 a**)	1.2	MeOH	5
3	TMS (**7 a**)	1.2	^i^PrOH	34
4	TMS (**7 a**)	1.2	^t^BuOH	50
5	TMS (**7 a**)	2.0	^t^BuOH	25
6	TMS (**7 a**)	1.0	^t^BuOH	57
7	TMS (**7 a**)	0.7	^t^BuOH	64 (63)^[c]^
8	TES (**7a′**)	0.7	^t^BuOH	50^[d]^
9	TBS (**7 a′′**)	0.7	^t^BuOH	43^[d]^

Reactions performed with 0.46 mmol of **6**. [a] ^1^H NMR yield of **4 a**. [b] Range of yields across 5 experiments, no detectable byproducts observed. [c] Yield of isolated **5 a** (relative to limiting reagent **7**) after silyl enol ether hydrolysis. [d] Yield of isolated **4 a** relative to limiting reagent **7**.

With these optimized conditions, in situ IR spectroscopy was used to investigate this complex reaction sequence (Figure 2).^[17]^ An unexpected observation was made during the synthesis of ABB‐Li (**1**) from **6**, where, despite being reported to *require* 3 hours at −78 °C,^[9a]^ this transformation was complete within the time taken for ^s^BuLi addition. During this process, consumption of **6** (839 cm^−1^) was accompanied by the appearance of a transient peak at 802 cm^−1^, corresponding to **2**, before complete conversion to **1** (772 cm^−1^).^[18]^ The absorption assigned to **1** (772 cm^−1^) showed a partial signal bleach upon addition of acyl silane **7 a**, with the simultaneous generation of a signal at 1048 cm^−1^_,_ attributed to **3 a**. Interestingly, **7 a** reacted so rapidly that the carbonyl stretch (1645 cm^−1^) was not observed on the timescale of the experiment.[^19^, ^20^] A steady state was reached once the addition of **7 a** was complete, indicating that the [1,2]‐Brook rearrangement/strain‐release‐driven anion relay sequence occurred essentially instantaneously at −78 °C and that the ring opening of ABB is a near barrierless process.[^13^, ^20^] Addition of ^t^BuOH resulted in an increase in absorption intensity at 1048 cm^−1^, indicating protonation of **3 a** to form **3 a‐H**, and quenching of excess **1** (772 cm^−1^) to regenerate **2** (802 cm^−1^). After warming to 0 °C, during which the relative absorptions of all wavenumbers are inherently affected, Boc_2_O was added, resulting in a decrease in intensity of the peak at 1048 cm^−1^. This occurred concomitantly with an increase in the product carbonyl stretch (1704 cm^−1^), which reached maximum intensity within 3 minutes. The present study clearly demonstrates the rapidity of the reaction, showing that the total time in which reactivity is occurring (≈15 minutes) is almost entirely dictated by the addition rate of the reagents.

**Figure 2:**
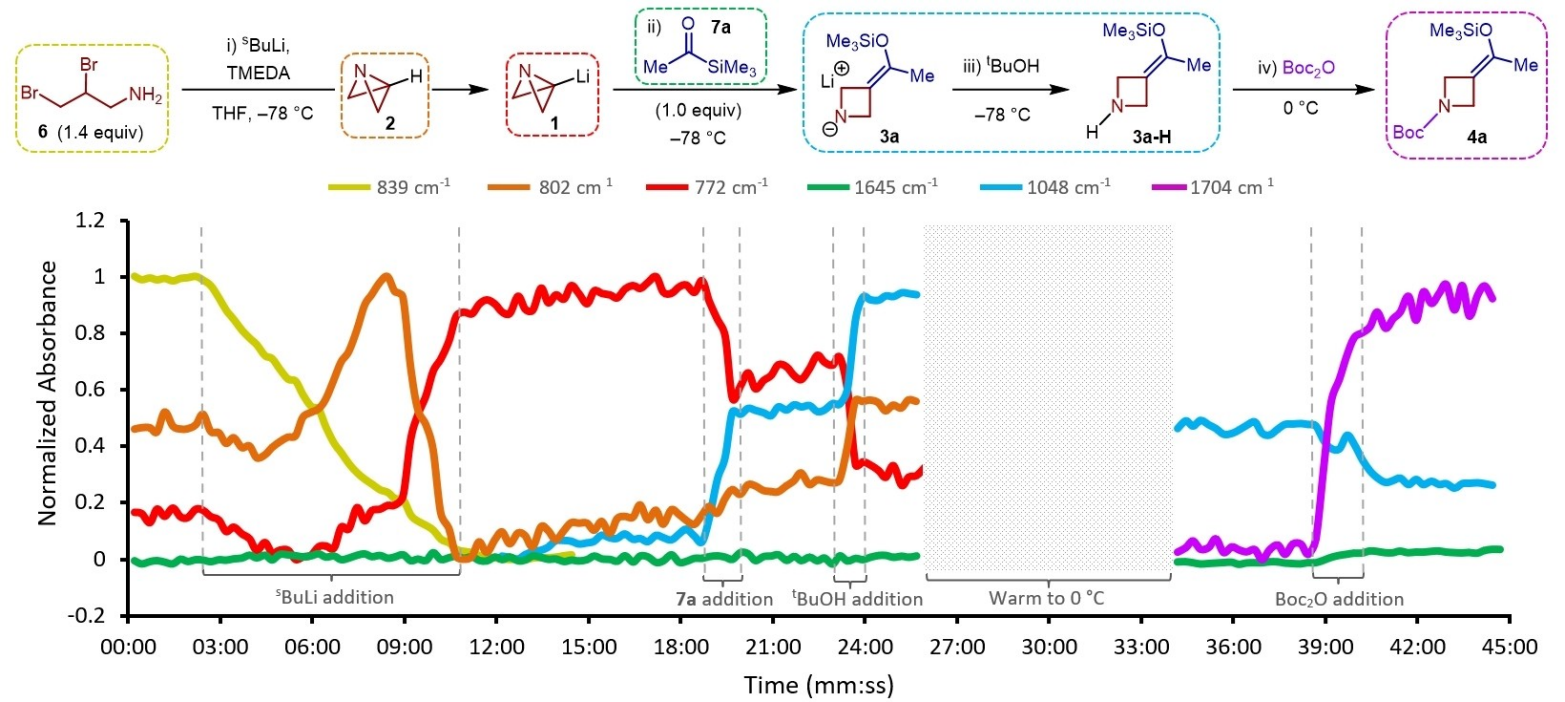
In situ IR spectroscopy studies. Assignments made based on steady state IR measurements and relative absorbance changes, see Supporting Information. Absorbances normalized to values between 0 and 1.

With a greater understanding of the reaction parameters, the scope with respect to E^1^ and E^2^ was investigated (Scheme 1). Pleasingly, the truncated reaction times for the synthesis of model substrate **5 a** gave an improved yield of 72 % after hydrolysis with aqueous HCl. Encouraged by this result, we successfully engaged other aliphatic acyl silanes bearing phenyl, alkenyl and cyclopropyl moieties to generate azetidine products **5 b**–**5 d**. Furthermore, protected alcohol and aldehyde fragments were tolerated in comparable yields, providing further sites for potential derivatization (**5 e**, **5 f**). Silyl enol ethers derived from benzoyltrimethylsilane were found to be susceptible to premature hydrolysis under the reaction conditions. However, employing the corresponding triethyl acylsilane allowed the exclusive formation of the silyl enol ether which could later be selectively hydrolyzed after aqueous workup to give phenyl ketone **5 g** in 40 % yield.

**Scheme 1:**
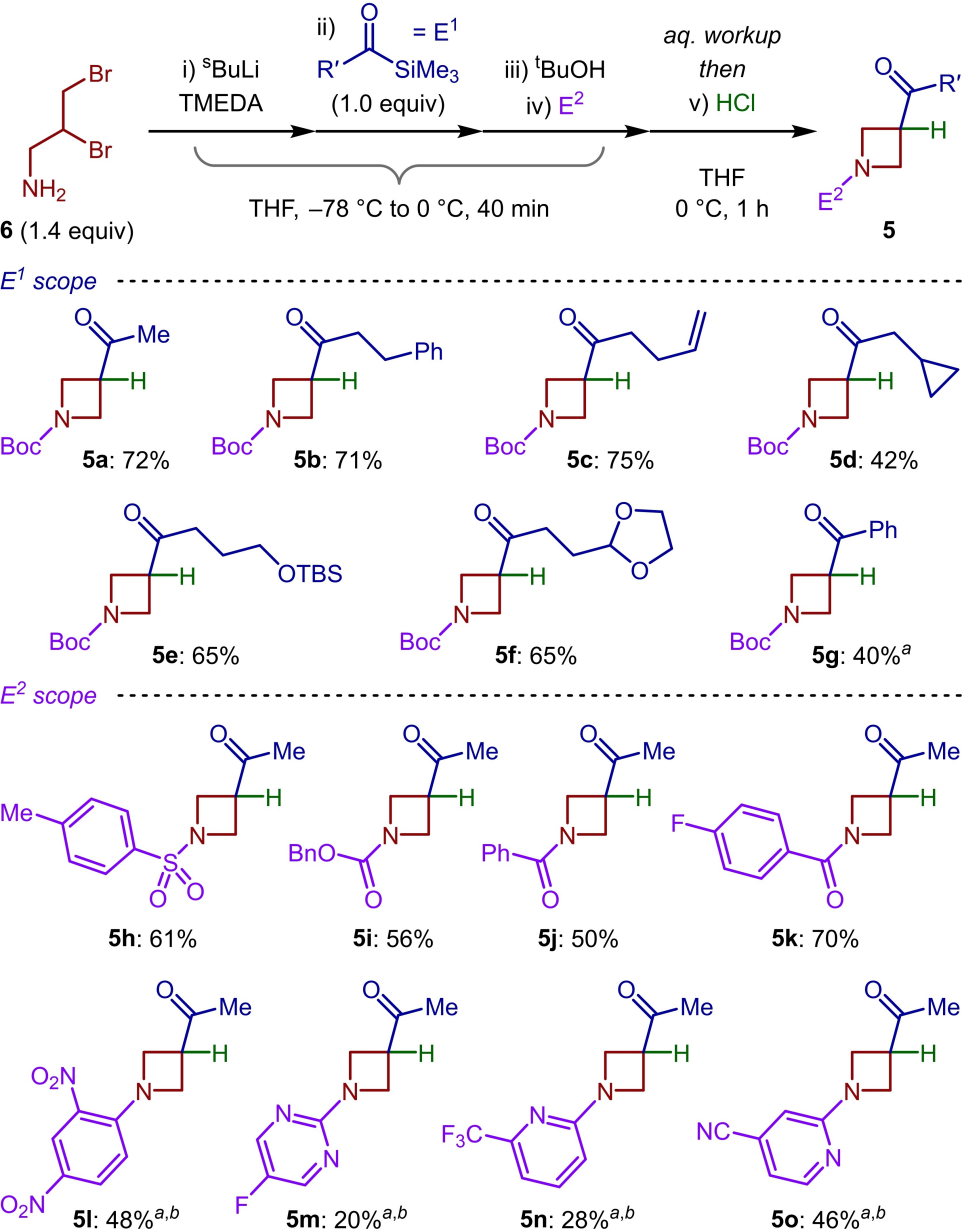
Scope of the [1,2]‐Brook rearrangement/strain‐release‐driven anion relay reaction. Reactions performed on 0.322 mmol of acyl silane E^1^. Isolated yields given. ^*a*^ Using the corresponding triethyl acylsilane. ^*b*^ Reactions warmed to rt for 16 h.

We then proceeded to explore the scope of E^2^ and were gratified to discover that, as well as Boc_2_O, toluenesulfonyl chloride (TsCl) and benzyl chloroformate (CbzCl) were similarly effective in accessing the corresponding sulfonamide and carbamate products (**5 h**, **5 i**). Amides **5 j** and **5 k** were also synthesized in good yields upon addition of the requisite benzoyl chlorides. Finally, it was demonstrated that the secondary amine intermediates could be engaged with aryl halides to access S_*N*_Ar products **5 l**–**5 o**. Again, premature hydrolysis under the reaction conditions necessitated the use of the more hindered acetyltriethylsilane, which improves silyl enol ether stability but comes at the cost of a reduced reaction yield.

The full realization of the four‐component coupling protocol was achieved through the addition of either N‐bromosuccinimide (NBS) or phenylselenyl chloride (PhSeCl) to silyl enol ether **4 a** in a single pot. Despite product formation being observed in reasonable yields considering the overall complexity of the reaction (28 % and 25 % for **8 a** and **8 d**, respectively, Scheme 2a), we were concerned by the efficiency of the final transformation. However, we discovered that performing an intermediate aqueous workup gave a near pure sample of silyl enol ether **4 a** (as determined by NMR spectroscopy) which could participate in a range of functionalization reactions in much improved yields. Exploring the scope of carbon–heteroatom bond formation allowed the generation of bromo, chloro and fluoro‐functionalized products (**8 a**–**8 c**). As well as selenide **8 d**, sulfide **8 e** was accessed in high yield from **4 a**. Similarly, employing Rubottom oxidation conditions provided the corresponding α‐hydroxy products **8 f** and **8 g**, with silyl deprotection dictated by the pH of the aqueous workup. We were also pleased to discover that carbon–carbon bonds were readily forged to access azetidine quaternary carbon centers. Aliphatic and aromatic aldehydes participated in the desired Mukaiyama aldol reaction, to give **8 h**–**8 j** in high yields. Furthermore, electrophilic organic salts could be employed to synthesize Mannich product **8 k** using Eschenmoser's salt, and compounds **8 l** and **8 m** from tropylium and benzodithiolylium tetrafluoroborate, respectively.

**Scheme 2:**
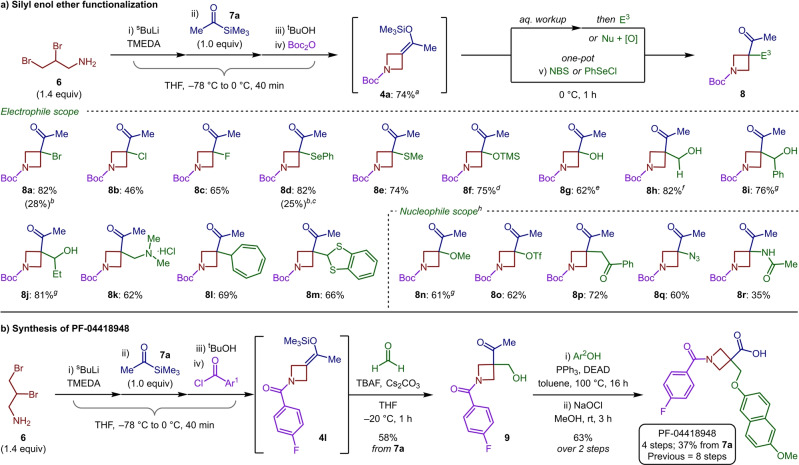
[1,2]‐Brook rearrangement/strain‐release‐driven anion relay reaction. a) Scope of silyl enol ether functionalization. b) Application to the synthesis of PF‐04418948. Reactions performed with 0.20 mmol of crude **4 a**, see Supporting Information for full conditions. Isolated yields from **4 a** given. ^*a* 1^H NMR yield. ^*b*^ One‐pot reaction from **7 a**. ^*c*^ Reaction warmed to rt for 16 h. ^*d*^ Basic aqueous workup. ^*e*^ Acidic aqueous workup. ^*f*^ With TBAF and Cs_2_CO_3_. ^*g*^ With BF_3_⋅OEt_2_. ^*h*^ With TsN=IPh or CAN.

Employing [N‐(*p*‐toluenesulfonyl)imino]phenyliodinane (TsN=IPh)^[21]^ facilitated the addition of nucleophiles such as methanol (**8 n**) and triflate (**8 o**) to this electron‐rich fragment. Silyl enol ether coupling could also be achieved using ceric ammonium nitrate (CAN)^[22]^ and 1‐phenyl‐1‐trimethylsiloxyethylene to provide 1,4‐dicarbonyl **8 p** in 72 % yield. Owing to the synthetic value of unnatural amino‐acid analogues^[23]^ we then targeted α‐amino azetidinyl ketone structures. To this end, azide **8 q** and acetamide **8 r**, the product of the Ritter reaction with acetonitrile, were accessed in 60 % and 35 % yield, respectively. The versatility of the silyl enol ether intermediates, with respect to the breadth of potential coupling partners, goes beyond what has been demonstrated with the comparable building block 3‐cyano azetidine, with the present protocol having the additional benefits of increased substrate diversity associated with a multicomponent approach.^[15a]^

Finally, to highlight the utility of this methodology, the synthesis of the orally active and selective EP2 receptor antagonist PF‐04418948 was targeted (Scheme 2b).^[24]^ This 1,3,3‐trisubstituted azetidine‐based pharmaceutical, previously accessed in 8 steps,^[25]^ was determined to possess nanomolar affinity for the target prostaglandin receptor (IC_50_=16 nM).^[24]^ Using the newly developed four‐component [1,2]‐Brook rearrangement/strain‐release‐driven anion relay protocol, key intermediate **9** was rapidly assembled in 58 % yield from **7 a**. From here, a Mitsunobu coupling with 6‐methoxy‐2‐naphthol and subsequent haloform reaction, to convert the methyl ketone to the corresponding carboxylic acid, delivered the pharmaceutical in an overall yield of 37 % over 4 steps, requiring only 2 chromatographic purifications.

In conclusion, we have developed a novel strategy for the highly modular multicomponent synthesis of substituted azetidines. The strategy harnesses the strain‐release reactivity of ABB to drive a [1,2]‐Brook rearrangement, with the rapidity of the reaction sequence demonstrated using in situ infra‐red spectroscopy. The reaction shows broad substrate scope with respect to the three electrophilic coupling partners and was applied to the 4‐step synthesis of the pharmaceutical PF‐04418948.

## Conflict of interest

The authors declare no conflict of interest.

## Supporting information

As a service to our authors and readers, this journal provides supporting information supplied by the authors. Such materials are peer reviewed and may be re‐organized for online delivery, but are not copy‐edited or typeset. Technical support issues arising from supporting information (other than missing files) should be addressed to the authors.

Supporting Information

## Data Availability

The data that support the findings of this study are available in the Supporting Information of this article.
