# Supplementary material for: Four‐Component Strain‐Release‐Driven Synthesis of Functionalized Azetidines
Source: Angew Chem Int Ed Engl. 2022 Nov 27;61(52):e202214049. doi: 10.1002/anie.202214049 (PMC10099845; doi:10.1002/anie.202214049)
Supplement: Supplementary file 1 — Supporting Information [file ANIE-61-0-s001.pdf]

Angewandte Chemie
Eine Zeitschrift der Gesellschaft Deutscher Chemiker

Supporting Information

Four-Component Strain-Release-Driven Synthesis of Functionalized Azetidines

*J. L. Tyler, A. Noble, V. K. Aggarwal**

TABLE OF CONTENTS

LIST OF SUPPLEMENTARY SCHEMES, FIGURES AND TABLES 2

LIST OF CHARACTERISED PRODUCTS 2

1. MATERIALS AND GENERAL METHODS 4
  1.1. Glassware, Solvents and Reagents 4
  1.2. Chromatography and Instrumentation 4
  1.3. Naming of Compounds 4

2. EXPERIMENTAL DATA 5
  2.1. Reaction Optimisation 5
    2.1.1. Reaction irreproducibility 5
    2.1.2. Protonation of lithium amide intermediate (**3a**) 6
    2.1.3. Acyl silane stoichiometry 7
    2.1.4. Reaction time 8
  2.2. Study of Silyl Enol Ether Hydrolysis 9
    2.2.1. Attempted synthesis of **4a** from *tert*-butyl 3-acetylazetidine-1-carboxylate 10
  2.3. General Procedures 11
    2.3.1. General Procedure A: Synthesis of acetyl(trialkyl)silanes 11
    2.3.2. General Procedure B: Synthesis of acyl silanes 11
    2.3.3. Synthesis of amine **6** 12
    2.3.4. General Procedure C: Synthesis of azetidines **5a-5o** 12
    2.3.5. General Procedure D: Synthesis of azetidines **8n-8r** 13
  2.4. Synthesis of Acyl Silanes 14
  2.5. Synthesis of Azetidines 20
  2.6. Four-Component One-Pot Synthesis of Azetidines 43
  2.7. Synthesis of PF-04418948 44
  2.8. Unsuccessful Substrates 47
  2.9. In Situ Infra-Red Spectroscopy Experiments 49
    2.9.1. React-IR setup procedure 49
    2.9.2. Steady state infra-red spectra 49
    2.9.3. Formation of ABB-Li 50
    2.9.4. [1,2]-Brook rearrangement/strain-release-driven anion relay reaction 51
    2.9.5. Reaction **3a-H** with Boc_2O 52

5. SPECTROSCOPIC DATA 53

6. REFERENCES 109

LIST OF SUPPLEMENTARY SCHEMES, FIGURES AND TABLES

Table S1: Irreproducibility of the [1,2]-Brook rearrangement/strain-release-driven anion relay reaction.......... S5

Table S2: Optimisation of proton source S6

Table S3: Optimisation of acyl silane stoichiometry S7

Table S4: Optimisation of reaction time........ S8

Scheme S1: Unsuccessful substrates in the synthesis of silyl enol ether **4**........ S47

Scheme S2: Unsuccessful aldol/Mannich reactions of **4a**........ S47

Scheme S3: Unsuccessful electrophilic functionalisation reactions of **4a**........ S48

Scheme S4: Unsuccessful oxidative nucleophilic coupling reactions of **4a** S48

Figure S1: Steady state infra-red spectra: (a) **4a**; (b) **6**; (c) Boc_2O; (d) ^{t}BuOH; (e) **7a**........ S49

Figure S2: 2D and 3D plots of absorbance versus time for species involved in the synthesis of ABB-Li (**1**) . S50

Figure S3: 2D and 3D plots of absorbance versus time for species involved in the [1,2]-Brook rearrangement/strain-release-driven anion relay reaction S51

Figure S4: 2D and 3D plots of absorbance versus time for the reaction of **3a-H** with Boc_2O........ S52

LIST OF CHARACTERISED PRODUCTS

1-(Triethylsilyl)ethan-1-one (**7a′**)........ S14

1-(*tert*-Butyldimethylsilyl)ethan-1-one (**7a″**)........ S14

3-Phenyl-1-(trimethylsilyl)propan-1-one (**7b**)........ S15

1-(Trimethylsilyl)pent-4-en-1-one (**7c**)........ S15

2-Cyclopropyl-1-(trimethylsilyl)ethan-1-one (**7d**) S16

4-((*tert*-Butyldimethylsilyl)oxy)-1-(trimethylsilyl)butan-1-one (**7e**)........ S16

3-(1,3-Dioxolan-2-yl)-1-(trimethylsilyl)propan-1-one (**7f**) S17

Phenyl(trimethylsilyl)methanone (**7g**) S18

Phenyl(triethylsilyl)methanone (**7g′**) S18

tert-Butyl 3-(1-((trimethylsilyl)oxy)ethylidene)azetidine-1-carboxylate (**4a**) S20

tert-Butyl 3-(1-((triethylsilyl)oxy)ethylidene)azetidine-1-carboxylate (**4a′**) S20

tert-Butyl 3-(1-((tert-butyldimethylsilyl)oxy)ethylidene)azetidine-1-carboxylate (**4a″**) S21

tert-Butyl 3-acetylazetidine-1-carboxylate (**5a**)........ S21

tert-Butyl 3-(3-phenylpropanoyl)azetidine-1-carboxylate (**5b**)........ S22

tert-Butyl 3-(pent-4-enoyl)azetidine-1-carboxylate (**5c**)........ S23

tert-Butyl 3-(2-cyclopropylacetyl)azetidine-1-carboxylate (**5d**)........ S23

tert-Butyl 3-(4-((tert-butyldimethylsilyl)oxy)butanoyl)azetidine-1-carboxylate (**5e**)........ S24

tert-Butyl 3-(3-(1,3-dioxolan-2-yl)propanoyl)azetidine-1-carboxylate (**5f**) S25

tert-Butyl 3-benzoylazetidine-1-carboxylate (**5g**)........ S25

1-(1-Tosylazetidin-3-yl)ethan-1-one (**5h**)........ S26

Benzyl 3-acetylazetidine-1-carboxylate (**5i**) S26

1-(1-Benzoylazetidin-3-yl)ethan-1-one (**5j**)........ S27

1-(1-(4-Fluorobenzoyl)azetidin-3-yl)ethan-1-one (**5k**) S28

1-(1-(2,4-Dinitrophenyl)azetidin-3-yl)ethan-1-one (**5l**)........ S28

1-(1-(5-Fluoropyrimidin-2-yl)azetidin-3-yl)ethan-1-one (**5m**)........ S29

1-(1-(6-(Trifluoromethyl)pyridin-2-yl)azetidin-3-yl)ethan-1-one (**5n**)........ S30

1-(1-(4-Cyanopyridin-2-yl)azetidin-3-yl)ethan-1-one (**5o**)........ S30
tert-Butyl 3-acetyl-3-bromoazetidine-1-carboxylate (**8a**) S31
tert-Butyl 3-acetyl-3-chloroazetidine-1-carboxylate (**8b**) S32
tert-Butyl 3-acetyl-3-fluoroazetidine-1-carboxylate (**8c**) S32
tert-Butyl 3-acetyl-3-(phenylselanyl)azetidine-1-carboxylate (**8d**) S33
tert-Butyl 3-acetyl-3-(methylthio)azetidine-1-carboxylate (**8e**)........ S34
tert-Butyl 3-acetyl-3-((trimethylsilyl)oxy)azetidine-1-carboxylate (**8f**) S34
tert-Butyl 3-acetyl-3-hydroxyazetidine-1-carboxylate (**8g**)........ S35
tert-Butyl 3-acetyl-3-(hydroxymethyl)azetidine-1-carboxylate (**8h**) S36
tert-Butyl 3-acetyl-3-(hydroxy(phenyl)methyl)azetidine-1-carboxylate (**8i**)........ S36
tert-Butyl 3-acetyl-3-(1-hydroxypropyl)azetidine-1-carboxylate (**8j**) S37
tert-Butyl 3-acetyl-3-((dimethylamino)methyl)azetidine-1-carboxylate hydrochloride (**8k**)........ S38
tert-Butyl 3-acetyl-3-(cyclohepta-2,4,6-trien-1-yl)azetidine-1-carboxylate (**8l**) S38
tert-Butyl 3-acetyl-3-(benzo[*d*][1,3]dithiol-2-yl)azetidine-1-carboxylate (**8m**) S39
tert-Butyl 3-acetyl-3-methoxyazetidine-1-carboxylatecarboxylate (**8n**)........ S40
tert-Butyl 3-acetyl-3-(((trifluoromethyl)sulfonyl)oxy)azetidine-1-carboxylate (**8o**) S40
tert-Butyl 3-acetyl-3-(2-oxo-2-phenylethyl)azetidine-1-carboxylate (**8p**)........ S41
tert-Butyl 3-acetyl-3-azidoazetidine-1-carboxylate carboxylate (**8q**) S41
tert-Butyl 3-acetamido-3-acetylazetidine-1-carboxylate (**8r**) S42
(4-Fluorophenyl)(3-(1-((trimethylsilyl)oxy)ethylidene)azetidin-1-yl)methanone (**4l**) S44
1-(1-(4-Fluorobenzoyl)-3-(hydroxymethyl)azetidin-3-yl)ethan-1-onemethanone (**9**)........ S44
1-(1-(4-Fluorobenzoyl)-3-(((6-methoxynaphthalen-2-yl)oxy)methyl)azetidin-3-yl)ethan-1-one (**10**) S45
1-(4-Fluorobenzoyl)-3-(((6-methoxynaphthalen-2-yl)oxy)methyl)azetidine-3-carboxylic acid (PF-04418948) S46

1. MATERIALS AND GENERAL METHODS

1.1. Glassware, Solvents and Reagents

All reactions were conducted under an inert atmosphere of nitrogen using Schlenk manifold techniques unless stated otherwise. All glassware was flame-dried prior to use. All anhydrous solvents were commercially supplied or dried using an Anhydrous Engineering alumina column drying system (THF, hexane, toluene, MeCN, Et_2O, CH_2Cl_2) and stored over 3 Å mol sieves. Reagents were purchased from commercial sources and used as received. ***Exceptions***: *N*,*N*,*N′*,*N′*-tetramethylethylenediamine (TMEDA) and trimethylsilyl chloride (TMSCl) were distilled over CaH_2 under an inert atmosphere at standard pressure. *n*-Butyllithium (1.6 M in hexane) and *s*-butyllithium (1.3 M in cyclohexane/hexane (92/8)) were purchased from Acros organics, *t*-Butyllithium (1.7M in pentane) was purchased from Sigma Aldrich and all were titrated against N-benzylbenzamide prior to use.

1.2. Chromatography and Instrumentation

Thin layer chromatography (TLC) was performed to monitor reactions when practical using Merck Kieselgel 60 F254 fluorescent treated silica, which was visualised under UV light, or by staining with aqueous basic potassium permanganate followed by heating. **Flash column chromatography** (FCC) was carried out using Sigma-Aldrich silica gel (60 Å, 230–400 mesh, 40–63 μm). **NMR spectra** were recorded at various field strengths, as indicated, using Varian VNMR 400 MHz, Varian VNMR 500 MHz, or Bruker Cryo 500 MHz for ^{1}H, ^{13}C and ^{19}F acquisitions. All NMR spectra were recorder at 25 °C unless otherwise stated. Chemical shifts (δ) are reported in parts per million (ppm) and referenced to $CDCl_3$ (^{1}H: 7.26 ppm; ^{13}C: 77.16 ppm). Coupling constants (*J*) are given in Hertz (Hz) and refer to corresponding multiplicities (s = singlet, d = doublet, t = triplet, q = quartet, quin = quintet, sex = hextet, h = heptet, m = multiplet, app = apparent, br. = broad signal, dd = doublet of doublets, etc.). The ^{1}H NMR spectra are reported as follows: chemical shift (multiplicity, coupling constants, number of protons, assignment). NMR assignments were made according to spin systems, using two-dimensional NMR spectroscopy (COSY, HSQC, HMBC) to assist the characterisation. Where an assignment could not be made unambiguously, no assignments are given. NMR yields were determined by ^{1}H NMR analysis using dibromomethane as an internal standard. **High resolution mass spectra (HRMS)** were recorded on a Bruker Daltonics MicrOTOF II by Electrospray Ionisation (ESI) on a Bruker ultrafleXtreme 2 (TOF-TOF) by matrix-assisted laser desorption/ionisation (MALDI), on a Thermo Scientific QExactive by electron ionisation (EI) or recorded on a Thermo Scientific Orbitrap Elite by ESI or Atmospheric Pressure Chemical Ionisation (APCI). **IR spectra** were recorded neat as a thin film on a Perkin Elmer Spectrum One FT-IR. Selected absorption maxima (ν_{max}) are reported in wavenumbers (cm^{-1}). **In Situ IR spectroscopy (React-IR)** experiments were monitored using Mettler Toledo React-IR 15 mid-infrared spectrometer equipped with a Silver Halide (AgX) FiberConduit with integrated DiComp probe, using the iC IR Reaction Analysis software (version 4.3). Prior to the use of the React-IR machine for data collection, the contrast and align, performance (3 runs) and stability (duration 5 min) were tested and saved.

1.3. Naming of Compounds

Compound names are those generated by ChemDraw Professional 20.0 software (PerkinElmer), following the IUPAC nomenclature.

2. EXPERIMENTAL DATA

2.1. Reaction Optimisation

Reactions were performed using **6** (0.46 mmol) according to a modified **General Procedure C** in which no silyl enol ether hydrolysis was performed. Modifications to standard conditions and key observations from each study are stated.

2.1.1. Reaction irreproducibility

Table S1: Irreproducibility of the [1,2]-Brook rearrangement/strain-release-driven anion relay reaction

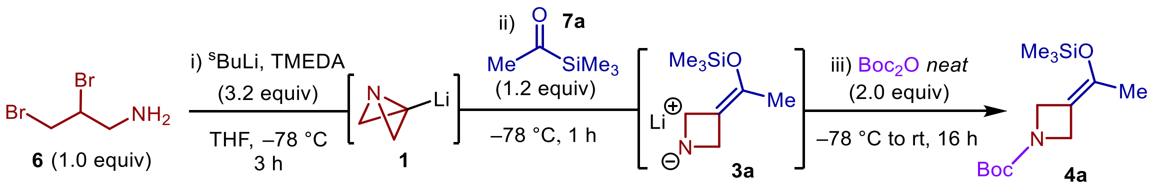

Entry	Deviation from standard conditions	% Yield[a]
**1**	*As above*	45
**2**	*As above*	40
**3**	*As above*	29
**4**	*As above*	36
**5**	*As above*	19
**6**	Boc_2O added in THF (1 M)	30
**7**	3 equiv of ^{s}BuLi	20
**8**	THF degassed	23

[a] Yields were determined by ^{1}H NMR analysis using dibromomethane as an internal standard.

Key observations: Standard reaction conditions could not be reproduced across multiple experiments (entries 1-5). Attempted column chromatography of **4a** resulted in almost complete loss of product due to hydrolysis. Slow addition of Boc_2O as a solution in THF, removing the excess base and degassing the solvent all failed to recover the initially observed yields (entries 6-8).

2.1.2. Protonation of lithium amide intermediate (3a)

Table S2: Optimisation of proton source

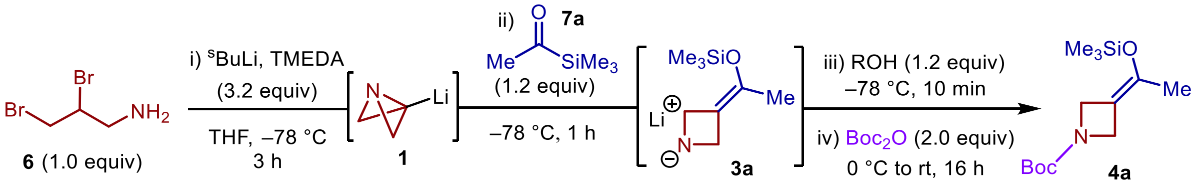

Entry	ROH	% Yield[a]
1	MeOH	5
2	^{i}PrOH	34
**3**	**^{t}BuOH**	**50**[b]
4	^{t}amyl alcohol	46
5	2,6-di-*tert*-butylphenol	9
6	^{t}BuOH	38[c]
7	^{t}BuOH	41[c,d]

[a] Yields were determined by ^{1}H NMR analysis using dibromomethane as an internal standard. [b] Result replicated across 9 experiments. [c] Boc_2O added at −78 °C. [d] Boc_2O added as a solution in THF.

Key observations: Protonation of lithium amide intermediate (**3a**) before addition of Boc_2O improved the yield of product formation and allowed the reaction result to be replicated across multiple repeat experiments. ^{t}BuOH was found to be optimal in the reaction (entry 3). Adding Boc_2O, both neat and as a solution in THF, at −78 °C before warming to 0 °C resulted in a lower yield of product (entries 6-7).

2.1.3. Acyl silane stoichiometry

Table S3: Optimisation of acyl silane stoichiometry

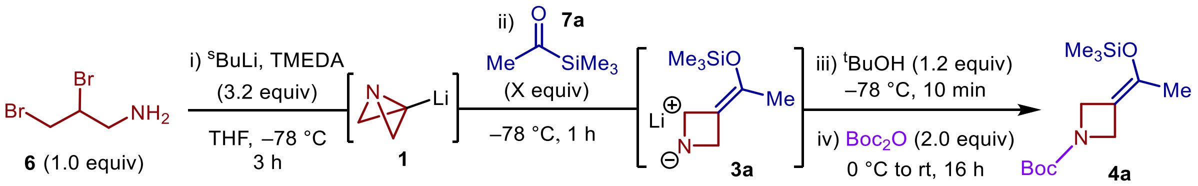

Entry	7a; X equiv	% Yield[a]
**1**	1.2	50
**2**	1.5	35
**3**	2.0	25
**4**	3.0	15
**5**	1.0	57
**6**	0.80	61[b]
**7**	**0.70**	**64**[b]
**8**	0.55	33[b]

[a] Yields were determined by ^{1}H NMR analysis using dibromomethane as an internal standard. [b] Yields were calculated relative to limiting reagent **7a**.

Key observations: Increasing the equivalents of acyl silane was found to promote product decomposition (entries 1-4). However, inverting the stoichiometry to have organolithium **1** in excess gave improved yields in the reaction (entries 5-8).

2.1.4. Reaction time

Table S4: Optimisation of reaction time

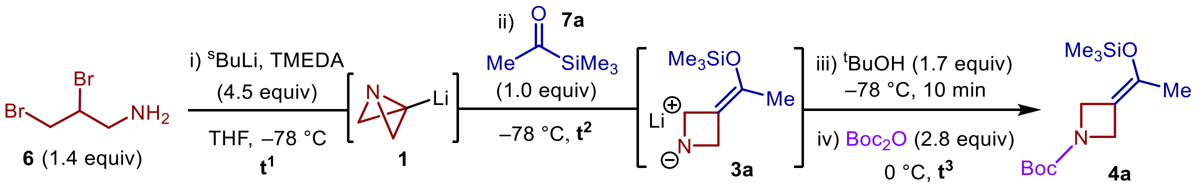

Entry	t¹	t²	t³	% Yield[a]
**1**	3 h	1 h	16 h[b]	64
**2**	3 h	2 h	16 h[b]	58
**3**	3 h	10 min	16 h[b]	62
**4**	15 min	10 min	16 h[b]	64
**5**	15 min	10 min	1 h	69
**6**	15 min	10 min	5 min	**74**

[a] Yields were determined by ^{1}H NMR analysis using dibromomethane as an internal standard and calculated relative to limiting reagent **7a**. [b] Reaction warmed to room temperature.

Key observations: In situ infra-red spectroscopy showed that the individual reactions took place much quicker than expected. Reaction times were systematically reduced, and it was demonstrated that the truncated reaction times could significantly improve the yield of **4a**. Presumably, this results from product instability under the reaction conditions.

2.2. Study of Silyl Enol Ether Hydrolysis

Reactions were performed using **7a** (0.322 mmol) according to a modified **General Procedure C** in which the reaction sequence and hydrolysis were performed in one-pot. Modifications to standard conditions and key observations from each study are stated.

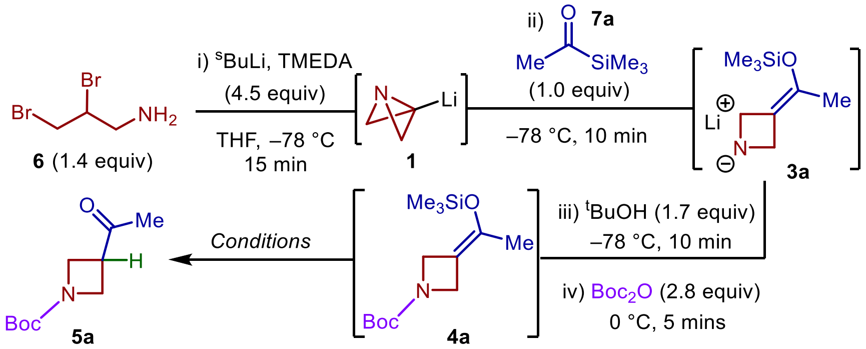

Entry	Conditions	4a; % Yield[a]	5a; % Yield[a]
1	1 M HCl (7.0 equiv), THF, 0 ºC, 3 h	72	0
2	1 M HCl (7.0 equiv), THF:MeOH (1:1), 0 ºC, 3 h	69	4
3	1 M HCl (7.0 equiv), THF:acetone (1:1), 0 ºC, 1 h	73	0
4	1 M HCl (7.0 equiv), THF:acetone (1:5), 0 ºC, 1 h	0	60
5	1 M TBAF (2.0 equiv), THF, 0 ºC, 30 min	0	56
6[b]	**0.1 M HCl (2.0 equiv), THF, 0 ºC, 1 h**	**0**	**72[c]**

[a] Yields were determined by ^{1}H NMR analysis using dibromomethane as an internal standard. [b] Performed aqueous workup on **4a** before subjecting to hydrolysis. [c] Isolated yield of **5a**.

Key observations: Employing HCl in organic solvents was not feasible as this promoted the polymerisation of THF. Due to the nature of the reaction solvent mixture, the addition of aqueous HCl resulted in a biphasic solution which prevented hydrolysis from occurring (entries 1-3). A single-phase solution could be achieved upon addition of 10 mL of acetone, generating hydrolysis product **5a** in 60% NMR yield (entry 4). However, this large increase in solvent volume is undesirable and not amenable to scale up. Using tetra-^{n}butylammonium fluoride (TBAF) also gave exclusively the hydrolysis product but the decrease in yield compared to the theoretical maximum (56% v 74%) suggests this process is inefficient. Finally, we discovered that performing an aqueous workup on **4a** before redissolving the crude mixture in THF allowed a single-phase solution to be formed upon addition of aqueous HCl and a near quantitative hydrolysis was achieved under mild conditions (entry 6).

2.2.1. Attempted synthesis of 4a from *tert*-butyl 3-acetylazetidine-1-carboxylate

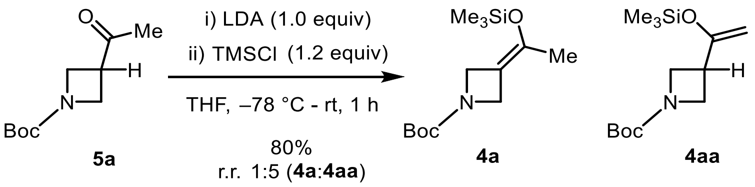

To a solution of diisopropylamine (14.1 µL, 1.00 equiv) in THF (0.5 mL) at 0 °C was added ^{n}BuLi (1.60 M in hexane, 62.5 µL, 1.00 equiv)[A] dropwise and the resulting solution was stirred for 15 min at 0 °C then cooled to −78 °C. **5a** (19.9 mg, 0.100 mmol, 1.00 equiv) in THF (0.5 mL). was added dropwise and the reaction was stirred for 30 min before the addition of TMSCl (15.2 µL, 1.20 equiv). The reaction was then warmed to room temperature over 30 min, quenched with H_2O (10 mL) and the mixture was extracted with EtOAc (3× 10 mL). The combined organic phases were dried ($MgSO_4$), filtered and concentrated under reduced pressure. Crude silyl enol ether **4a**/**4aa** (0.080 mmol, 80% as calculated by quantitative ^{1}H NMR using dibromomethane as internal standard) was determined to have a regiomeric ratio (r.r.) of 1:5.[B]

Notes: (**A**) Organolithiums should be carefully titrated prior to use. (**B**) This ratio is in line with previous reports of 3-acetylazetidine enolization.[1]

2.3. General Procedures

2.3.1. General Procedure A: Synthesis of acetyl(trialkyl)silanes

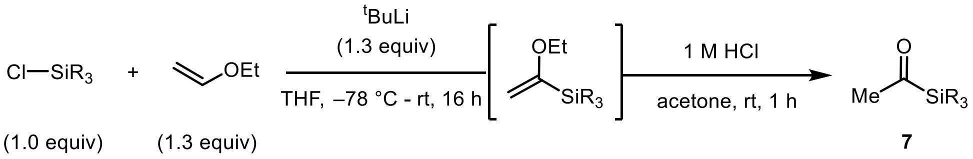

Following a modified literature procedure.[2]

To a solution of ethyl vinyl ether (1.24 mL, 1.30 equiv) in THF (15 mL) at −78 °C was added ^{t}BuLi (1.70 M in pentane, 7.65 mL, 1.30 equiv) [A] at a rate of 0.25 mL/min *via* syringe pump. The resulting solution was warmed to room temperature and stirred for 1 h before being cooled to −78 °C. To this was added silyl chloride (10 mmol, 1.0 equiv) and the reaction was allowed to slowly warm to room temperature over 15 h. H_2O (20 mL) was then added to quench the reaction, and the mixture was extracted with Et_2O (3× 20 mL). The combined organic phases were dried ($MgSO_4$), filtered, and concentrated under reduced pressure. The crude residue was then dissolved in acetone (12 mL) and to this was added 1 M HCl (6 mL). The solution was stirred at room temperature for 1 h then diluted with H_2O (20 mL) and extracted with Et_2O (3× 20 mL). The combined organic phases were dried ($MgSO_4$), filtered, concentrated under reduced pressure and the residue was purified by flash column chromatography on silica gel to yield the corresponding acyl silane (**7**).[B]

Notes: (**A**) Organolithiums should be carefully titrated prior to use. (**B**) Acetyl(trialkyl)silanes can most effectively be visualised on silica gel thin-layer chromatography (TLC) plates using a phosphomolybdic acid stain.

2.3.2. General Procedure B: Synthesis of acyl silanes

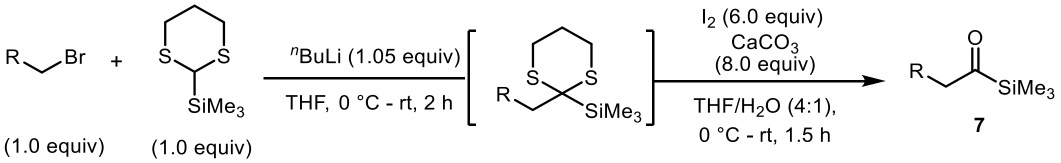

Following a modified literature procedure.[3]

To a solution of (1,3-dithian-2-yl)trimethylsilane (0.38 mL, 2.0 mmol, 1.0 equiv) in THF (8.5 mL) at 0 °C was added ^{n}BuLi (1.60 M in hexane, 1.31 mL, 1.05 equiv) [A] dropwise and stirred for 30 mins at 0 °C. To this solution was added alkyl bromide (2.0 mmol, 1.0 equiv) and the reaction was allowed to warm to room temperature over 2 h. Sat. aq. NH_4Cl (10 mL) was then added to quench the reaction, and the mixture was extracted with Et_2O (3× 10 mL). The combined organic phases were dried ($MgSO_4$), filtered, and concentrated under reduced pressure. The crude residue was then dissolved in THF:H_2O (4:1, 15 mL) and cooled to 0 °C. To this solution was added $CaCO_3$ (1.6 g, 8.0 equiv) and I_2 (3.05 g, 6.00 equiv) then warmed to room temperature and stirred for 1.5 h. The reaction was then quenched with sat. aq. $Na_2S_2O_3$ (4 mL) and filtered through a short plug of silica gel eluting with Et_2O. The resulting filtrate was diluted with H_2O (15 mL) and extracted with Et_2O (3× 15 mL). The combined organic phases were dried ($MgSO_4$), filtered, concentrated under reduced pressure and the residue was purified by flash column chromatography on silica gel to yield the corresponding acyl silane (**7**).

Notes: (**A**) Organolithiums should be carefully titrated prior to use.

2.3.3. Synthesis of amine 6

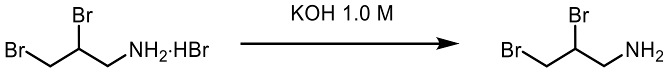

6

Following a modified literature procedure.[4] Reaction performed in the absence of light.

1-Amino-2,3-dibromopropane hydrobromide (0.25 g, 0.84 mmol) was dissolved in KOH (aq.) (1.0 M, 1.6 mL) and subsequently extracted with CH_2Cl_2 (2× 1.6 mL). The combined organic phases were dried ($MgSO_4$), filtered, and concentrated under reduced pressure to give amine **6** (0.167 g, 0.770 mmol) as a colourless oil. The amine was immediately used for the subsequent reaction as polymerisation of **6** could be detected within 1 h at room temperature.

2.3.4. General Procedure C: Synthesis of azetidines 5a-5o

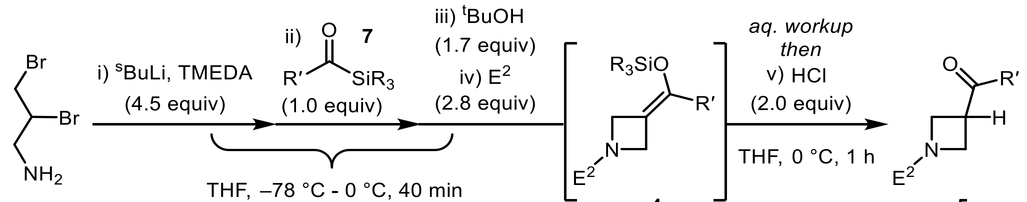

To a solution of freshly made **6** (99.8 mg, 1.40 equiv) and TMEDA (0.221 mL, 4.50 equiv)[A] in THF (2.3 mL) at −78 °C was added ^{s}BuLi (1.30 M in cyclohexane/hexane (92/8), 1.13 mL, 4.50 equiv)[B] dropwise and the resulting solution was stirred for 15 min at −78 °C. Acyl silane **7** (0.322 mmol, 1.00 equiv) was added dropwise and the reaction was stirred for 10 min before the addition of ^{t}BuOH (53.4 µL, 1.70 equiv) in THF (0.3 mL). The reaction was stirred for 10 min at −78 °C then warmed to 0 °C. E^2 (2.80 equiv) was added, and the resulting solution was stirred for 5 min. H_2O (10 mL) was then added to quench the reaction, and the mixture was extracted with EtOAc (3× 10 mL). The combined organic phases were dried ($MgSO_4$), filtered, and concentrated under reduced pressure. The crude silyl enol ether was dissolved in THF (6 mL) and cooled to 0 °C. To this was added 0.1 M aq. HCl (6.4 mL, 2.0 equiv) and the reaction was stirred at 0 °C for 1 h. Sat. aq. $NaHCO_3$ (10 mL) was added to quench the reaction and the resulting solution was diluted with H_2O and extracted with EtOAc (3× 10 mL). The combined organic phases were dried ($MgSO_4$), filtered, concentrated under reduced pressure and the residue was purified by flash column chromatography on silica gel to yield the corresponding azetidine (**5**).

Notes: (**A**) TMEDA should be distilled over CaH_2 prior to use. (**B**) Organolithiums should be carefully titrated prior to use.

2.3.5. General Procedure D: Synthesis of azetidines 8n-8r

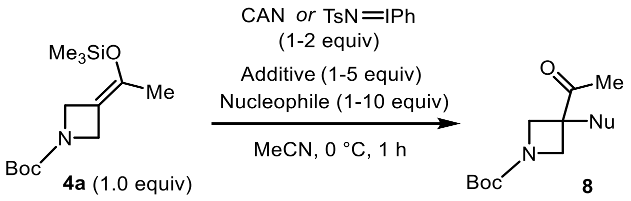

To a solution of crude **4a** (0.20 mmol, 1.0 equiv) and ceric ammonium nitrate (CAN) or [N-(*p*-toluenesulfonyl)imino]phenyliodinane (TsN=IPh) (1-2 equiv) in MeCN (2 mL) at 0 °C was added a nucleophile (1-10 equiv) and an additive (1-5 equiv) the resulting mixture was stirred for 1 h at 0 °C. H_2O (5 mL) was added, and the mixture was extracted with EtOAc (3× 10 mL). The combined organic phases were dried ($MgSO_4$), filtered, concentrated under reduced pressure and the residue was purified by flash column chromatography to yield the corresponding azetidine (**8**).

2.4. Synthesis of Acyl Silanes

1-(Triethylsilyl)ethan-1-one (7a′)

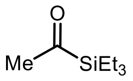

7a′

Synthesised following **General Procedure A** using: triethylsilyl chloride (1.68 mL, 10.0 mmol, 1.00 equiv). Purified by flash column chromatography (SiO_2; 99:1 to 98:2 pentane:Et_2O) to afford **7a′** (0.671 g, 4.24 mmol, 42%) as a colourless oil.

TLC: R_f = 0.22 (99:1 pentane:Et_2O).

NMR Spectroscopy (*see spectra*):

^{1}**H NMR** (400 MHz, $CDCl_3$): δ_H 2.25 (s, 3H, C*H*$_3$), 0.98 (t, *J* = 7.9 Hz, 9H, Si(CH_2C*H*$_3$)$_3$), 0.74 (q, *J* = 7.9 Hz, 6H, Si(C*H*$_2$$CH_3$)$_3$) ppm;

^{13}**C NMR** (126 MHz, $CDCl_3$): δ_C 247.6 (*C*=O), 37.5 (*C*H$_3$), 7.4 (Si(CH_2*C*H$_3$)$_3$), 2.2 (Si(*C*H$_2$$CH_3$)$_3$) ppm.

HRMS (EI^+): m/z calc'd for $C_7H_{15}OSi$ $[M-Me]^+$: 143.0887, found: 143.0886.

IR (film): ν_{max} 2955, 2877, 1641 (C=O), 1458, 1413, 1338, 1135, 1009 cm^{-1}.

1-(*tert*-Butyldimethylsilyl)ethan-1-one (7a″)

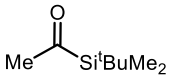

7a″

Synthesised following **General Procedure A** using: *tert*-butyldimethylsilyl chloride (1.51 g, 10.0 mmol, 1.00 equiv). Purified by flash column chromatography (SiO_2; 90:10 hexane:EtOAc) to afford **7a″** (0.707 g, 4.47 mmol, 45%) as a colourless oil.

TLC: R_f = 0.45 (90:10 hexane:EtOAc).

NMR Spectroscopy (*see spectra*):

^{1}**H NMR** (400 MHz, $CDCl_3$): δ_H 2.27 (s, 3H, C*H*$_3$), 0.94 (s, 9H, SiC(C*H*$_3$)$_3$), 0.19 (s, 6H, Si(C*H*$_3$)$_2$) ppm;

^{13}**C NMR** (126 MHz, $CDCl_3$): δ_C 247.1(*C*=O), 37.8 (*C*H$_3$), 26.5 (SiC(*C*H$_3$)$_3$), 16.6 (Si*C*(CH_3)$_3$), −6.9 (Si(*C*H$_3$)$_2$) ppm.

HRMS (EI^+): m/z calc'd for $C_7H_{15}OSi$ $[M-Me]^+$: 143.0887, found: 143.0886.

IR (film): ν_{max} 2954, 2930, 2859, 1641 (C=O), 1464, 1339, 1249, 1134, 1008, 836 cm^{-1}.

3-Phenyl-1-(trimethylsilyl)propan-1-one (7b)

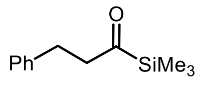

7b

Synthesised following **General Procedure B** using: 2-bromoethylbenzene (0.272 mL, 2.00 mmol, 1.00 equiv). Purified by flash column chromatography (SiO_2; 98:2 pentane:Et_2O) to afford **7b** (0.260 g, 1.26 mmol, 63%) as a colourless oil.

TLC: R_f = 0.26 (98:2 pentane:Et_2O).

NMR Spectroscopy (*see spectra*):

> ^{1}**H NMR** (500 MHz, $CDCl_3$): δ_H 7.30 – 7.25 (m, 2H, ArC*H*), 7.21 – 7.15 (m, 3H, ArC*H*), 2.97 – 2.90 (m, 2H, (C=O)C*H*$_2$), 2.87 – 2.81 (m, 2H, PhC*H*$_2$), 0.19 (s, 9H, Si(C*H*$_3$)$_3$) ppm;
>
> ^{13}**C NMR** (126 MHz, $CDCl_3$): δ_C 247.2 (*C*=O), 141.8 (Ar*C*), 128.6 (Ar*C*H), 128.5 (Ar*C*H), 126.1 (Ar*C*H), 50.2 ((C=O)*C*H$_2$), 28.4 (Ph*C*H$_2$), −3.1 (Si(*C*H$_3$)$_3$) ppm.

HRMS (EI^+): m/z calc'd for $C_{12}H_{17}OSi$ $[M-H]^+$: 205.1043, found: 205.1042.

IR (film): ν_{max} 3027, 2957, 1642 (C=O), 1497, 1249, 835 cm^{-1}.

1-(Trimethylsilyl)pent-4-en-1-one (7c)

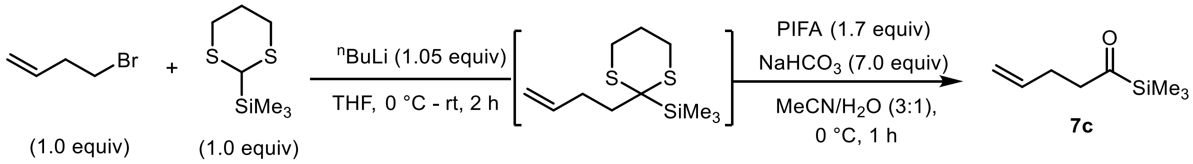

7c

Following a modified literature procedure.[5]

To a solution of (1,3-dithian-2-yl)trimethylsilane (0.38 mL, 2.0 mmol, 1.0 equiv) in THF (8.5 mL) at 0 °C was added ^{n}BuLi (1.60 M in hexane, 1.31 mL, 1.05 equiv) [A] dropwise and stirred for 30 mins at 0 °C. To this solution was added 4-bromobut-1-ene (0.203 mL, 2.00 mmol, 1.00 equiv) and the reaction was allowed to warm to room temperature over 2 h. Sat. aq. NH_4Cl (10 mL) was then added to quench the reaction, and the mixture was extracted with Et_2O (3× 10 mL). The combined organic phases were dried ($MgSO_4$), filtered, and concentrated under reduced pressure. The crude residue, dissolved in MeCN (3 mL), was then added to $NaHCO_3$ (1.18 g, 7.00 equiv) in H_2O (2 mL) and cooled to 0 °C. To this was added (bis(trifluoroacetoxy)iodo)benzene (PIFA) (1.46 g, 1.70 equiv) in MeCN (3 mL) and the solution was stirred at 0 °C for 1 h. The reaction was then diluted with H_2O (10 mL) and extracted with Et_2O (3× 15 mL). The combined organic phases were dried ($MgSO_4$), filtered, concentrated under reduced pressure and the residue was purified by flash column chromatography (SiO_2; 90:10 to 70:30 pentane:CH_2Cl_2) to afford **7c** (0.252 g, 1.61 mmol, 81%) as a colourless oil.

Notes: (**A**) Organolithiums should be carefully titrated prior to use.

TLC: R_f = 0.24 (70:30 pentane:CH_2Cl_2).

NMR Spectroscopy (*see spectra*):

^{1}H NMR (500 MHz, $CDCl_3$): δ_H 5.79 (ddt, *J* = 16.9, 10.2, 6.6 Hz, 1H, *H*C=CH_2), 5.00 (app-dq, *J* = 16.9, 1.7 Hz, 1H, HC=C*H*$_2$), 4.95 (ddt, *J* = 10.2, 1.7, 1.3 Hz, 1H, HC=C*H*$_2$), 2.70 (t, *J* = 7.4 Hz, 2H, (C=O)C*H*$_2$), 2.31 – 2.23 (m, 2H, C*H*$_2$(HC=CH_2)), 0.20 (s, 9H, Si(C*H*$_3$)$_3$) ppm;

^{13}C NMR (126 MHz, $CDCl_3$): δ_C 247.4 (*C*=O), 137.8 (H*C*=CH_2), 115.0 (HC=*C*H$_2$), 47.5 ((C=O)*C*H$_2$), 26.3 (*C*H$_2$(HC=CH_2)), −3.1 (Si(*C*H$_3$)$_3$) ppm.

HRMS (EI^+): m/z calc'd for $C_8H_{16}OSi$ $[M-H]^+$: 155.0887, found: 155.0886.

IR (film): ν_{max} 2924, 1641 (C=O), 1594, 1520, 1462, 1344, 1203, cm^{-1}.

2-Cyclopropyl-1-(trimethylsilyl)ethan-1-one (7d)

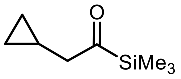

7d

Synthesised following **General Procedure B** using: (bromomethyl)cyclopropane (0.194 mL, 2.00 mmol, 1.00 equiv). Purified by flash column chromatography (SiO_2; 98:2 to 97:3 pentane:Et_2O) to afford **7d** (0.239 g, 1.53 mmol, 76%) as a colourless oil.

TLC: R_f = 0.18 (98:2 pentane:Et_2O).

NMR Spectroscopy (*see spectra*):

^{1}H NMR (500 MHz, $CDCl_3$): δ_H 2.48 (d, *J* = 6.8 Hz, 2H, C*H*$_2$(C=O)), 1.04 – 0.91 (m, 1H, cyclopropane C*H*), 0.62 – 0.47 (m, 2H, cyclopropane C*H*$_2$), 0.20 (s, 9H, Si(C*H*$_3$)$_3$), 0.11 – 0.01 (m, 2H, cyclopropane C*H*$_2$) ppm;

^{13}C NMR (126 MHz, $CDCl_3$): δ_C 248.4 (*C*=O), 53.9 ((C=O)*C*H$_2$), 5.0 (cyclopropane *C*H), 4.5 (cyclopropane 2×*C*H$_2$), −3.0 (Si(*C*H$_3$)$_3$) ppm.

HRMS (EI^+): m/z calc'd for $C_8H_{16}OSi$ $[M-H]^+$: 155.0887, found: 155.0887.

IR (film): ν_{max} 3080, 3005, 2960, 1643 (C=O), 1249, 1018, 842 cm^{-1}.

4-((*tert*-Butyldimethylsilyl)oxy)-1-(trimethylsilyl)butan-1-one (7e)

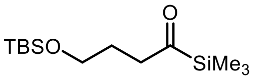

7e

Synthesised following **General Procedure B** using: (3-bromopropoxy)(*tert*-butyl)dimethylsilane (0.463 mL,

2.00 mmol, 1.00 equiv). Purified by flash column chromatography (SiO_2; 99:1 to 96:4 pentane:Et_2O) to afford **7e** (0.346 g, 1.26 mmol, 63%) as a colourless oil.

TLC: R_f = 0.25 (98:2 pentane:Et_2O).

NMR Spectroscopy (see spectra):

^{1}**H NMR** (500 MHz, $CDCl_3$): δ_H 3.58 (t, *J* = 6.2 Hz, 2H, C*H*$_2$OSi), 2.68 (t, *J* = 7.2 Hz, 2H, (C=O)C*H*$_2$), 1.72 (tt, *J* = 7.2, 6.2 Hz, 2H, (C=O)CH$_2$C*H*$_2$), 0.89 (s, 9H, OSiC(C*H*$_3$)$_3$), 0.20 (s, 9H, Si(C*H*$_3$)$_3$), 0.03 (s, 6H, OSi(C*H*$_3$)$_2$) ppm;

^{13}**C NMR** (126 MHz, $CDCl_3$): δ_C 248.2 (*C*=O), 62.5 (*C*H$_2$OSi), 44.8 ((C=O)*C*H$_2$), 26.1 (OSiC(*C*H$_3$)$_3$), 25.3 ((C=O)CH$_2$*C*H$_2$), 18.5 (Si*C*(CH$_3$)$_3$), −3.0 (Si(*C*H$_3$)$_3$), −5.2 (Si(*C*H$_3$)$_2$) ppm.

HRMS (ESI$^+$): m/z calc'd for $C_{13}H_{20}O_2Si_2$ $[M+H]^+$: 275.1857, found: 275.1860.

IR (film): ν_{max} 2956, 2858, 1645 (C=O), 1472, 1251, 1101, 837 cm^{-1}.

3-(1,3-Dioxolan-2-yl)-1-(trimethylsilyl)propan-1-one (7f)

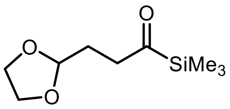

7f

Synthesised following **General Procedure B** using: 2-(2-bromoethyl)-1,3-dioxolane (0.235 mL, 2.00 mmol, 1.00 equiv). Purified by flash column chromatography (SiO_2; 80:20 pentane:Et_2O) to afford **7f** (0.346 g, 1.26 mmol, 63%) as a colourless oil.

TLC: R_f = 0.25 (80:20 pentane:Et_2O).

NMR Spectroscopy (see spectra):

^{1}**H NMR** (500 MHz, $CDCl_3$): δ_H 4.88 (t, *J* = 4.5 Hz, 1H, C*H*(OCH$_2$)$_2$), 4.00 – 3.90 (m, 2H, OC*H*$_2$C*H*$_2$O), 3.88 – 3.79 (m, 2H, OC*H*$_2$C*H*$_2$O), 2.73 (t, *J* = 7.3 Hz, 2H, (C=O)C*H*$_2$), 1.92 (td, *J* = 7.3, 4.5 Hz, C*H*$_2$CH$_2$(C=O)), 0.20 (s, 9H, Si(C*H*$_3$)$_3$) ppm;

^{13}**C NMR** (126 MHz, $CDCl_3$): δ_C 246.6 (*C*=O), 103.8 (*C*H(OCH$_2$)$_2$), 65.1 (O*C*H$_2$*C*H$_2$O), 42.2 ((C=O)*C*H$_2$), 26.1 ((C=O)CH$_2$*C*H$_2$), −3.0 (Si(*C*H$_3$)$_2$) ppm.

HRMS (ESI$^+$): m/z calc'd for $C_9H_{18}O_3Si$ $[M+H]^+$: 203.1098, found: 203.1099.

IR (film): ν_{max} 2959, 2887, 1644 (C=O), 1409, 1250, 1139, 1139, 1031, 843 cm^{-1}.

Phenyl(trimethylsilyl)methanone (7g)

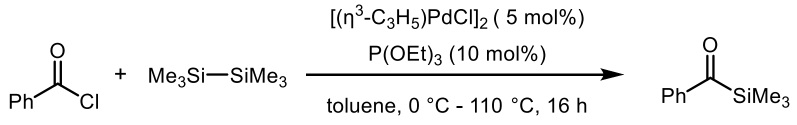

Following a literature procedure.[6]

Benzoyl chloride (0.581 mL, 5.00 mmol, 1.00 equiv) was added dropwise to a stirred solution of allylpalladium chloride dimer (91.5 mg, 5 mol%), triethyl phosphite (85.8 µL, 10 mol%) and hexamethyldisilane (1.69 mL, 1.65 equiv) in toluene (3 mL) at 0 °C and stirred for 5 min. The mixture was heated to 110 °C and stirred at this temperature for 16 h. After cooling to room temperature, the solution was filtered through a short plug of silica eluting with Et_2O. The solvent was removed under reduced pressure and the residue was purified by flash column chromatography (SiO_2; 98:2 pentane:Et_2O) to afford **7g** (0.401 g, 2.25 mmol, 45%) as a yellow oil.

TLC: R_f = 0.23 (98:2 pentane:Et_2O).

NMR Spectroscopy (*see spectra*):

^{1}H NMR (400 MHz, $CDCl_3$): δ_H 7.87 – 7.80 (m, 2H, ArC*H*), 7.58 – 7.51 (m, 1H, ArC*H*), 7.51 – 7.44 (m, 2H, ArC*H*), 0.38 (s, 9H, Si(C*H*$_3$)$_3$) ppm;

^{13}C NMR (126 MHz, $CDCl_3$): δ_C 236.0 (*C*=O), 141.5 (Ar*C*), 132.9 (Ar*C*H), 128.8 (Ar*C*H), 127.6 (Ar*C*H), −1.2 (Si(*C*H$_3$)$_3$) ppm.

HRMS (EI^+): m/z calc'd for $C_{10}H_{13}OSi$ $[M-H]^+$: 177.0730, found: 177.0729.

IR (film): ν_{max} 3062, 2960, 2901, 1613 (C=O), 1446, 1250, 1209, 833 cm^{-1}.

Phenyl(triethylsilyl)methanone (7g′)

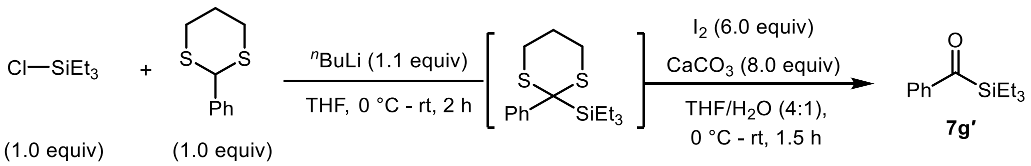

Following a modified **General Procedure B**.

To a solution of 2-phenyl-1,3-dithiane (589 mg, 3.00 mmol, 1.00 equiv) in THF (12 mL) at 0 °C was added ^{n}BuLi (1.60 M in hexane, 2.06 mL, 1.10 equiv)[A] dropwise and the resulting mixture was stirred for 30 mins at 0 °C. To this solution was added trimethylsilyl chloride (0.504 mL, 3.00 mmol, 1.00 equiv) and the reaction was allowed to warm to room temperature over 2 h. Sat. aq. NH_4Cl (15 mL) was then added to quench the reaction, and the mixture was extracted with Et_2O (3× 15 mL). The combined organic phases were dried ($MgSO_4$), filtered, and concentrated under reduced pressure. The crude residue was then dissolved in THF:H_2O 4:1 (22.5 mL) and cooled to 0 °C. To this solution was added $CaCO_3$ (2.4 g, 8.0 equiv) and I_2 (4.57 g, 6.00 equiv) then warmed to room temperature and stirred for 1.5 h. The reaction was then quenched with sat. aq. $Na_2S_2O_3$ (4 mL) and

filtered through a short plug of silica gel eluting with Et_2O. The resulting filtrate was diluted with H_2O (15 mL) and extracted with Et_2O (3× 15 mL). The combined organic phases were dried ($MgSO_4$), filtered, concentrated under reduced pressure and the residue was purified by flash column chromatography (SiO_2; 99:1 to 98:2 pentane:Et_2O) to afford **7g′** (0.524 g, 2.38 mmol, 79%) as a yellow oil.

Notes: (**A**) Organolithiums should be carefully titrated prior to use.

TLC: R_f = 0.30 (99:1 pentane:Et_2O).

NMR Spectroscopy (*see spectra*):

^{1}**H NMR** (400 MHz, $CDCl_3$): δ_H 7.84 – 7.75 (m, 2H, ArC*H*), 7.59 – 7.43 (m, 3H, ArC*H*), 1.02 – 0.96 (m, 9H, Si(CH_2CH_3)$_3$), 0.95 – 0.87 (m, 6H, Si(CH_2CH_3)$_3$) ppm;

^{13}**C NMR** (126 MHz, $CDCl_3$): δ_C 236.3 (*C*=O), 142.6 (Ar*C*), 132.8 (Ar*C*H), 128.8 (Ar*C*H), 127.3 (Ar*C*H), 7.6 (Si(CH_2CH_3)$_3$), 3.8 (Si(CH_2CH_3)$_3$) ppm.

HRMS (EI^+): m/z calc'd for $C_{13}H_{19}OSi$ $[M-H]^+$: 219.1200, found: 219.1199.

IR (film): ν_{max} 3062, 2954, 2875, 1612 (C=O), 1576, 1446, 1207, 1170, 1012, 767 cm^{-1}.

2.5. Synthesis of Azetidines

tert-Butyl 3-(1-((trimethylsilyl)oxy)ethylidene)azetidine-1-carboxylate (4a)

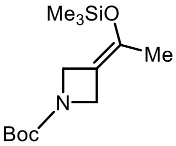

4a

Synthesised following a modified **General Procedure C** using: acetyltrimethylsilane (46.2 µL, 0.322 mmol, 1.00 equiv) and Boc_2O (0.211 mL, 2.80 equiv). After aqueous workup, crude silyl enol ether **4a** (0.238 mmol, 74% as calculated by quantitative ^{1}H NMR using dibromomethane as internal standard) was used without further purification in the subsequent reactions.

NMR Spectroscopy (_see spectra_):

^{1}H NMR (400 MHz, $CDCl_3$): δ_H 4.41 – 4.35 (m, 4H, 2×NCH_2), 1.63 (quin, J = 1.6 Hz, 3H, CH_3), 1.45 (s, 9H, OC(CH_3)$_3$), 0.17 (s, 9H, Si(CH_3)$_3$) ppm;

^{13}C NMR (126 MHz, $CDCl_3$): δ_C 156.6 (*C*=O), 141.1 (*C*=*C*OSi), 105.7 (NCH$_2$(*C*=C)), 79.6 (O*C*(CH$_3$)$_3$), 56.2 (N*C*H$_2$), 54.8 (N*C*H$_2$), 28.6 (OC(*C*H$_3$)$_3$), 18.6 (*C*H$_3$), 0.8 (Si(*C*H$_3$)$_3$) ppm.

HRMS: Molecular ion peak not observed by standard ionization modes (ESI, EI or APCI). Hydrolysis performed to confirm identity matched **5a**.

IR (film): ν_{max} 2964, 2864, 1704 (C=O), 1389, 1243, 1153, 1109, 1069, 989, 845 cm^{-1}.

tert-Butyl 3-(1-((triethylsilyl)oxy)ethylidene)azetidine-1-carboxylate (4a′)

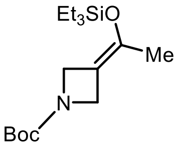

4a′

Synthesised following a modified **General Procedure C** using: **7a′** (51.0 mg, 0.322 mmol, 1.00 equiv) and Boc_2O (0.211 mL, 2.80 equiv). After aqueous workup the crude silyl enol ether was purified by flash column chromatography (SiO_2; 94:6 hexane:EtOAc) to afford **4a′** (57.8 mg, 0.184 mmol, 57%)[**A**] as a colourless oil.

Notes: (**A**) Yield of **4a′** was determined to be 50% using the unoptimised conditions with extended reaction times. For these reaction time details see Table S4, entry 1.

TLC: R_f = 0.21 (94:6 hexane:EtOAc).

NMR Spectroscopy (_see spectra_):

^{1}H NMR (400 MHz, $CDCl_3$): δ_H 4.45 – 4.36 (m, 4H, 2×NCH_2), 1.65 (quin, J = 1.6 Hz, 3H, CH_3), 1.44 (s, 9H, OC(CH_3)$_3$), 0.97 (t, J = 7.9 Hz, 9H, Si(CH$_2$CH_3)$_3$), 0.63 (q, J = 7.9 Hz, 6H, Si(CH_2CH$_3$)$_3$) ppm;

^{13}**C NMR** (126 MHz, $CDCl_3$): δ_C 156.6 (*C*=O), 141.2 (*C*=*C*OSi), 105.2 (NCH_2(*C*=C)), 79.6 (O*C*(CH_3)$_3$), 55.8 (N*C*H_2), 55.0 (N*C*H_2), 28.5 (OC(*C*H_3)$_3$), 18.6 (*C*H_3), 6.8 (Si(CH_2*C*H_3)$_3$), 5.5 (Si(*C*H_2CH_3)$_3$) ppm.

HRMS (ESI^+): m/z calc'd for $C_{16}H_{31}NNaO_3Si$ $[M+Na]^+$: 336.1971, found: 336.1970.

IR (film): ν_{max} 2957, 2877, 1704 (C=O), 1389, 1246, 1150, 1109, 1068, 989, 777 cm^{-1}.

tert-Butyl 3-(1-((tert-butyldimethylsilyl)oxy)ethylidene)azetidine-1-carboxylate (4a″)

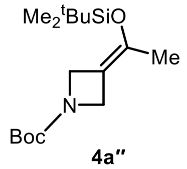

4a″

Synthesised following a modified **General Procedure C** using: **7a″** (51.0 mg, 0.322 mmol, 1.00 equiv) and Boc_2O (0.211 mL, 2.80 equiv). After aqueous workup the crude silyl enol ether was purified by flash column chromatography (SiO_2; 94:6 hexane:EtOAc) to afford **4a″** (49.2 mg, 0.157 mmol, 49%)[A] as a colourless oil.

Notes: (**A**) Yield of **4a″** was determined to be 43% using the unoptimised conditions with extended reaction times. For these reaction time details see Table S4, entry 1.

TLC: R_f = 0.27 (92:8 hexane:EtOAc).

NMR Spectroscopy (*see spectra*):

^{1}**H NMR** (400 MHz, $CDCl_3$): δ_H 4.42 – 4.36 (m, 4H, 2×NC*H*$_2$), 1.63 (quin, *J* = 1.6 Hz, 3H, C*H*$_3$), 1.45 (s, 9H, OC(C*H*$_3$)$_3$), 0.91 (s, 9H, SiC(C*H*$_3$)$_3$), 0.12 (s, 6H, Si(C*H*$_3$)$_2$) ppm;

^{13}**C NMR** (126 MHz, $CDCl_3$): δ_C 156.6 (*C*=O), 141.2 (*C*=*C*OSi), 105.4 (NCH_2(*C*=C)), 79.6 (O*C*(CH_3)$_3$), 55.7 (N*C*H_2), 54.9 (N*C*H_2), 28.6 (OC(*C*H_3)$_3$), 25.7 (SiC(*C*H_3)$_3$), 18.6 (*C*H_3), 18.1 (Si*C*(CH_3)$_3$), −3.9 (Si(*C*H_3)$_2$) ppm. *Weak azetidine ^{13}C NMR signals due to severe peak broadening, chemical shift confirmed with HSQC spectroscopy.*

HRMS: Molecular ion peak not observed by standard ionization modes (ESI, EI or APCI). Hydrolysis performed to confirm identity matched **5a**.

IR (film): ν_{max} 2957, 2930, 2850, 1703 (C=O), 1378, 1243, 1149, 1109, 1068, 988, 832 cm^{-1}.

tert-Butyl 3-acetylazetidine-1-carboxylate (5a)

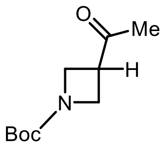

5a

Synthesised following **General Procedure C** using: acetyltrimethylsilane (46.2 µL, 0.322 mmol, 1.00 equiv) and Boc_2O (0.211 mL, 2.80 equiv). Purified by flash column chromatography (SiO_2; 65:35 hexane:EtOAc) to afford **5a** (46.2 mg, 0.232 mmol, 72%) as a colourless oil.

TLC: R_f = 0.25 (65:35 hexane:EtOAc).

NMR Spectroscopy (*see spectra*):

^{1}H NMR (400 MHz, $CDCl_3$): δ_H 4.04 (d, *J* = 7.6 Hz, 4H, 2×NC*H*$_2$), 3.41 (quin, *J* = 7.6 Hz, 1H, C*H*(C=O)), 2.17 (s, 3H, C*H*$_3$), 1.43 (s, 9H, OC(C*H*$_3$)$_3$) ppm;

^{13}C NMR (126 MHz, $CDCl_3$): δ_C 206.0 (*C*=O), 156.4 ((*C*=O)OC(CH$_3$)$_3$), 80.0 (O*C*(CH$_3$)$_3$), 50.6 (2×N*C*H$_2$), 39.5 (*C*H(C=O)), 28.5 (OC(*C*H$_3$)$_3$), 27.8 (*C*H$_3$) ppm.

HRMS (ESI$^+$): m/z calc'd for $C_{10}H_{17}NNaO_3$ $[M+Na]^+$: 222.1201, found: 222.1093.

IR (film): ν_{max} 2974, 2876, 1700 (C=O), 1394, 1365, 1133, 1066 cm^{-1}.

***tert*-Butyl 3-(3-phenylpropanoyl)azetidine-1-carboxylate (5b)**

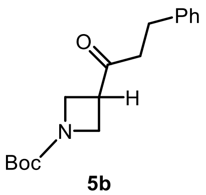

5b

Synthesised following **General Procedure C** using: **7b** (66.5 mg, 0.322 mmol, 1.00 equiv) and Boc_2O (0.211 mL, 2.80 equiv). Purified by flash column chromatography (SiO_2; 75:25 hexane:EtOAc) to afford **5b** (66.2 mg, 0.229 mmol, 71%) as a colourless oil.

TLC: R_f = 0.23 (75:25 hexane:EtOAc).

NMR Spectroscopy (*see spectra*):

^{1}H NMR (500 MHz, $CDCl_3$): δ_H 7.30 – 7.26 (m, 2H, ArC*H*), 7.22 – 7.15 (m, 3H, ArC*H*), 3.99 (d, *J* = 7.5 Hz, 4H, 2×NC*H*$_2$), 3.37 (quin, *J* = 7.5 Hz, 1H, C*H*(C=O)), 2.93 (t, *J* = 7.6 Hz, 2H, PhC*H*$_2$), 2.74 (t, *J* = 7.6 Hz, 2H, (C=O)C*H*$_2$), 1.42 (s, 9H, OC(C*H*$_3$)$_3$) ppm;

^{13}C NMR (126 MHz, $CDCl_3$): δ_C 207.3 (*C*=O), 156.3 ((*C*=O)OC(CH$_3$)$_3$), 140.8 (Ar*C*), 128.7 (Ar*C*H), 128.4 (Ar*C*H), 126.5 (Ar*C*H), 79.9 (O*C*(CH$_3$)$_3$), 50.6 (2×N*C*H$_2$), 42.5 ((C=O)*C*H$_2$), 38.8 (*C*H(C=O)), 29.6 (Ph*C*H$_2$), 28.5 (OC(*C*H$_3$)$_3$) ppm.

HRMS (ESI$^+$): m/z calc'd for $C_{17}H_{23}NNaO_3$ $[M+Na]^+$: 312.1570, found: 312.1567.

IR (film): ν_{max} 2975, 2889, 1699 (C=O), 1402, 1366, 1134, 700 cm^{-1}.

tert-Butyl 3-(pent-4-enoyl)azetidine-1-carboxylate (5c)

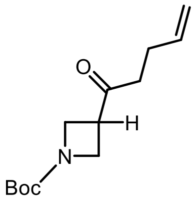

5c

Synthesised following **General Procedure C** using: **7c** (50.3 mg, 0.322 mmol, 1.00 equiv) and Boc_2O (0.211 mL, 2.80 equiv). Purified by flash column chromatography (SiO_2; 75:25 hexane:EtOAc) to afford **5c** (58.1 mg, 0.243 mmol, 75%) as a colourless oil.

TLC: R_f = 0.24 (75:25 hexane:EtOAc).

NMR Spectroscopy (_see spectra_):

> **^{1}H NMR** (500 MHz, $CDCl_3$): δ_H 5.79 (ddt, *J* = 16.6, 10.0, 6.3 Hz, 1H, *H*C=CH$_2$), 5.06 – 5.01 (m, 1H, HC=C*H*$_2$), 4.99 (ddd, *J* = 10.0, 2.0, 0.9 Hz, 1H, HC=C*H*$_2$), 4.08 – 3.95 (m, 4H, 2×NC*H*$_2$), 3.42 (quin, *J* = 7.5 Hz, 1H, C*H*(C=O)), 2.52 (t, *J* = 7.3 Hz, 2H, (C=O)C*H*$_2$), 2.39 – 2.30 (m, 2H, C*H*$_2$(HC=CH$_2$)), 1.43 (s, 9H, OC(C*H*$_3$)$_3$) ppm;

> **^{13}C NMR** (126 MHz, $CDCl_3$): δ_C 207.4 (*C*=O), 156.3 ((*C*=O)OC(CH$_3$)$_3$), 136.8 (H*C*=CH$_2$), 115.8 (HC=*C*H$_2$), 79.9 (O*C*(CH$_3$)$_3$), 50.7 (2×N*C*H$_2$), 40.0 (*C*H(C=O)), 38.6 (*C*H(C=O)), 28.5 (OC(*C*H$_3$)$_3$), 27.5 (*C*H$_2$(HC=CH$_2$)) ppm.

HRMS (ESI$^+$): m/z calc'd for $C_{13}H_{22}NO_3$ $[M+H]^+$: 240.1594, found: 240.1595.

IR (film): ν_{max} 2977, 2890, 1697 (C=O), 1395, 1365, 1130, 912 cm^{-1}.

tert-Butyl 3-(2-cyclopropylacetyl)azetidine-1-carboxylate (5d)

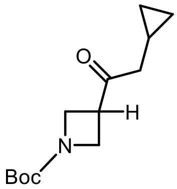

5d

Synthesised following **General Procedure C** using: **7d** (50.3 mg, 0.322 mmol, 1.00 equiv) and Boc_2O (0.211 mL, 2.80 equiv). Purified by flash column chromatography (SiO_2; 75:25 hexane:EtOAc) to afford **5d** (32.3 mg, 0.135 mmol, 42%) as a colourless oil.

TLC: R_f = 0.22 (75:25 hexane:EtOAc).

NMR Spectroscopy (_see spectra_):

> **^{1}H NMR** (500 MHz, $CDCl_3$): δ_H 4.13 – 3.99 (m, 4H, 2×NC*H*$_2$), 3.58 – 3.47 (m, 1H, C*H*(C=O)), 2.31 (d, *J* =

7.0 Hz, 2H, C*H*$_2$(C=O)), 1.43 (s, 9H, OC(C*H*$_3$)$_3$), 1.05 – 0.85 (m, 1H, cyclopropane C*H*), 0.72 – 0.51 (m, 2H, cyclopropane C*H*$_2$), 0.23 – 0.01 (m, 2H, cyclopropane C*H*$_2$) ppm;

^{13}C NMR (126 MHz, $CDCl_3$): δ_C 208.1 (*C*=O), 156.4 ((*C*=O)OC(CH$_3$)$_3$), 79.9 (O*C*(CH$_3$)$_3$), 50.6 (2×N*C*H$_2$), 46.5 ((C=O)*C*H$_2$), 38.2 (*C*H(C=O)), 28.5 (OC(*C*H$_3$)$_3$), 6.1 (cyclopropane *C*H), 4.8 (cyclopropane 2×*C*H$_2$) ppm.

HRMS (ESI$^+$): m/z calc'd for $C_{13}H_{22}NO_3$ $[M+H]^+$: 240.1594, found: 240.1600.

IR (film): v_{max} 2975, 2889, 1699 (C=O), 1408, 1392, 1133 cm^{-1}.

***tert*-Butyl 3-(4-((tert-butyldimethylsilyl)oxy)butanoyl)azetidine-1-carboxylate (5e)**

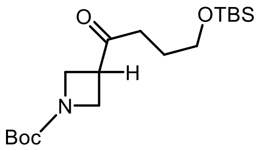

5e

Synthesised following **General Procedure C** using: **7e** (88.4 mg, 0.322 mmol, 1.00 equiv) and Boc_2O (0.211 mL, 2.80 equiv). Purified by flash column chromatography (SiO_2; 75:25 hexane:EtOAc) to afford **5e** (74.9 mg, 0.209 mmol, 65%) as a colourless oil.

TLC: R_f = 0.26 (75:25 hexane:EtOAc).

NMR Spectroscopy (*see spectra*):

^{1}H NMR (500 MHz, $CDCl_3$): δ_H 4.11 – 3.93 (m, 4H, 2×NC*H*$_2$), 3.61 (t, *J* = 6.0 Hz, 2H, C*H*$_2$OSi), 3.52 – 3.37 (m, 1H, C*H*(C=O)), 2.50 (t, *J* = 7.1 Hz, 2H, (C=O)C*H*$_2$), 1.80 (tt, *J* = 7.1, 6.0 Hz, 2H, (C=O)CH$_2$C*H*$_2$), 1.43 (s, 9H, OC(C*H*$_3$)$_3$), 0.87 (s, 9H, OSiC(C*H*$_3$)$_3$), 0.03 (s, 6H, OSi(C*H*$_3$)$_2$) ppm;

^{13}C NMR (126 MHz, $CDCl_3$): δ_C 208.2 (*C*=O), 156.4 ((*C*=O)OC(CH$_3$)$_3$), 79.9 (O*C*(CH$_3$)$_3$), 62.0 (*C*H$_2$OSi), 50.7 (2×N*C*H$_2$), 38.7 (*C*H(C=O)), 37.2 ((C=O)*C*H$_2$), 28.5 (OC(*C*H$_3$)$_3$), 26.7 ((C=O)CH$_2$*C*H$_2$), 26.1 (OSiC(*C*H$_3$)$_3$), 18.4 (OSi*C*(CH$_3$)$_3$), −5.2 (OSi(*C*H$_3$)$_2$) ppm.

HRMS (ESI$^+$): m/z calc'd for $C_{18}H_{35}NNaO_4Si$ $[M+Na]^+$: 380.2228, found: 380.2231.

IR (film): v_{max} 2956, 2930, 2888, 1704 (C=O), 1401, 1366, 1256, 1134, 1101, 836 cm^{-1}.

tert-Butyl 3-(3-(1,3-dioxolan-2-yl)propanoyl)azetidine-1-carboxylate (5f)

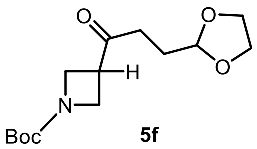

5f

Synthesised following **General Procedure C** using: **7f** (65.2 mg, 0.322 mmol, 1.00 equiv) and Boc_2O (0.211 mL, 2.80 equiv). Purified by flash column chromatography (SiO_2; 50:50 hexane:EtOAc) to afford **5f** (59.7 mg, 0.209 mmol, 65%) as a colourless oil.

TLC: R_f = 0.21 (50:50 hexane:EtOAc).

NMR Spectroscopy (*see spectra*):

^{1}**H NMR** (500 MHz, $CDCl_3$): δ_H 4.91 (t, *J* = 4.2 Hz, 1H, C*H*(OCH_2)$_2$), 4.12 – 3.99 (m, 4H, 2×NC*H*$_2$), 3.98 – 3.90 (m, 2H, OC*H*$_2$C*H*$_2$O), 3.89 – 3.80 (m, 2H, OC*H*$_2$C*H*$_2$O), 3.44 (quin, *J* = 7.5 Hz, 1H, C*H*(C=O)), 2.53 (t, *J* = 7.2 Hz, 2H, (C=O)C*H*$_2$), 2.01 (td, *J* = 7.2, 4.2 Hz, 2H, C*H*$_2$$CH_2$(C=O)), 1.43 (s, 9H, OC(C*H*$_3$)$_3$) ppm;

^{13}**C NMR** (126 MHz, $CDCl_3$): δ_C 207.4 (*C*=O), 156.4 ((*C*=O)OC(CH_3)$_3$), 103.1 (*C*H(OCH_2)$_2$), 79.9 (O*C*(CH_3)$_3$), 65.2 (O*C*H$_2$*C*H$_2$O), 50.8 (2×N*C*H$_2$), 38.6 (*C*H(C=O)), 34.5 ((C=O)*C*H$_2$), 28.5 (OC(*C*H$_3$)$_3$), 27.3 ((C=O)CH_2*C*H$_2$) ppm.

HRMS (ESI^+): m/z calc'd for $C_{14}H_{23}NNaO_5$ $[M+Na]^+$: 308.1468, found: 308.1473.

IR (film): ν_{max} 2953, 2888, 1699 (C=O), 1402, 1366, 1134, 1032 cm^{-1}.

tert-Butyl 3-benzoylazetidine-1-carboxylate (5g)

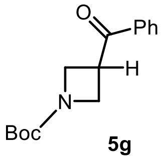

5g

Synthesised following a modified **General Procedure C** using: **7g′** (57.4 mg, 0.322 mmol, 1.00 equiv) and Boc_2O (0.211 mL, 2.80 equiv). The intermediate triethylsilyl enol ether required 0.1 M aq. HCl (2.8 equiv) and warming to room temperature for 4 h to achieve complete hydrolysis. Purified by flash column chromatography (SiO_2; 90:10 to 80:20 hexane:EtOAc) to afford **5g** (33.8 mg, 0.129 mmol, 40%) as a colourless oil.

TLC: R_f = 0.21 (90:10 hexane:EtOAc).

NMR Spectroscopy (*see spectra*):

^{1}**H NMR** (500 MHz, $CDCl_3$): δ_H 7.87 – 7.79 (m, 2H, ArC*H*), 7.65 – 7.56 (m, 1H, ArC*H*), 7.54 – 7.41 (m, 2H, ArC*H*), 4.31 – 4.08 (m, 5H, 2×NC*H*$_2$, C*H*(C=O)), 1.44 (s, 9H, OC(C*H*$_3$)$_3$) ppm;

^{13}**C NMR** (126 MHz, $CDCl_3$): δ_C 197.3 (*C*=O), 156.3 ((*C*=O)OC(CH_3)$_3$), 135.0 (Ar*C*), 133.8 (Ar*C*H), 129.1

(Ar*C*H), 128.4 (Ar*C*H), 79.9 (O*C*(CH3)3), 51.0 (2×N*C*H2), 35.8 (*C*H(C=O)), 28.5 (OC(*C*H3)3) ppm.

HRMS (ESI+): m/z calc'd for $C_{15}H_{19}NNaO_3$ $[M+Na]^+$: 284.1257, found: 284.1260.

IR (film): ν_{max} 2976, 2890, 1699 (C=O), 1449, 1398, 1345, 1223, 1136, 943 cm^{-1}.

1-(1-Tosylazetidin-3-yl)ethan-1-one (5h)

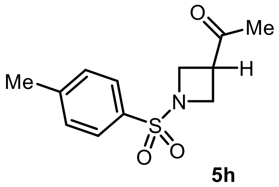

5h

Synthesised following **General Procedure C** using: acetyltrimethylsilane (46.2 µL, 0.322 mmol, 1.00 equiv) and 4-toluenesulfonyl chloride (TsCl) (175 mg, 2.80 equiv). Purified by flash column chromatography (SiO_2; 50:50 hexane:EtOAc) to afford **5h** (49.7 mg, 0.196 mmol, 61%) as a white solid.

TLC: R_f = 0.26 (50:50 hexane:EtOAc).

NMR Spectroscopy (*see spectra*):

^{1}H NMR (500 MHz, $CDCl_3$): δ_H 7.72 (d, *J* = 8.0 Hz, 2H, ArC*H*), 7.37 (d, *J* = 8.0 Hz, 2H, ArC*H*), 3.95 (dd, *J* = 9.0, 8.4 Hz, 2H, NC*H*2), 3.85 (dd, *J* = 8.4, 6.8 Hz, 2H, NC*H*2), 3.31 (tt, *J* = 9.0, 6.8 Hz, 1H, C*H*(C=O)), 2.46 (s, 3H, ArC*H*3), 2.05 (s, 3H, C*H*3) ppm;

^{13}C NMR (126 MHz, $CDCl_3$): δ_C 204.5 (*C*=O), 144.5 (Ar*C*), 131.4 (Ar*C*), 130.0 (Ar*C*H), 128.5 (Ar*C*H), 52.0 (2×N*C*H2), 38.8 (*C*H(C=O)), 27.6 (*C*H3), 21.8 (Ar*C*H3) ppm.

HRMS (ESI+): m/z calc'd for $C_{12}H_{16}NO_3S$ $[M+H]^+$: 254.0845, found: 254.0848.

IR (film): ν_{max} 2955, 3885, 1717 (C=O), 1342 (S=O), 1157, 1095, 817, 677 cm^{-1}.

Benzyl 3-acetylazetidine-1-carboxylate (5i)

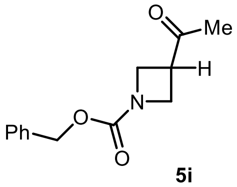

5i

Synthesised following **General Procedure C** using: acetyltrimethylsilane (46.2 µL, 0.322 mmol, 1.00 equiv) and benzyl chloroformate (CbzCl) (0.131 mL, 2.80 equiv). Purified by flash column chromatography (SiO_2; 50:50 hexane:EtOAc) to afford **5i** (42.3 mg, 0.181 mmol, 56%) as a colourless oil.

TLC: R_f = 0.26 (50:50 hexane:EtOAc).

NMR Spectroscopy (*see spectra*):

^{1}H NMR (500 MHz, $CDCl_3$): δ_H 7.44 – 7.28 (m, 5H, ArC*H*), 5.09 (s, 2H, OC*H*$_2$Ph), 4.14 (d, *J* = 7.7 Hz, 4H, 2×NC*H*$_2$), 3.46 (quin, *J* = 7.7 Hz, 1H, C*H*(C=O)), 2.17 (s, 3H, C*H*$_3$) ppm;

^{13}C NMR (126 MHz, $CDCl_3$): δ_C 205.5 (*C*=O), 156.5 ((*C*=O)OCH$_2$Ph), 136.6 (Ar*C*), 128.6 (Ar*C*H), 128.3 (Ar*C*H), 128.2 (Ar*C*H), 67.0 (O*C*H$_2$Ph), 50.8 (2×N*C*H$_2$), 39.8 (*C*H(C=O)), 27.9 (*C*H$_3$) ppm.

HRMS (ESI$^+$): m/z calc'd for $C_{13}H_{16}NO_3$ $[M+H]^+$: 234.1125, found: 234.1127.

IR (film): ν_{max} 2961, 2890, 1701 (C=O), 1416, 1354, 1175, 1127, 767, 699 cm^{-1}.

1-(1-Benzoylazetidin-3-yl)ethan-1-one (5j)

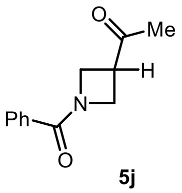

5j

Synthesised following **General Procedure C** using: acetyltrimethylsilane (46.2 µL, 0.322 mmol, 1.00 equiv) and benzoyl chloride (0.107 mL, 2.80 equiv). Purified by flash column chromatography (SiO_2; 20:80 hexane:EtOAc) to afford **5j** (32.9 mg, 0.162 mmol, 50%) as a colourless oil.

TLC: R_f = 0.23 (20:80 hexane:EtOAc).

NMR Spectroscopy (*see spectra*):

^{1}H NMR (500 MHz, $CDCl_3$): δ_H 7.64 – 7.59 (m, 2H, ArC*H*), 7.50 – 7.45 (m, 1H, ArC*H*), 7.44 – 7.39 (m, 2H, ArC*H*), 4.58 – 4.46 (m, 1H, NC*H*$_2$), 4.46 – 4.18 (m, 3H, NC*H*$_2$), 3.56 (tt, *J* = 9.2, 6.0 Hz, 1H, C*H*(C=O)), 2.21 (s, 3H, C*H*$_3$) ppm;

^{13}C NMR (126 MHz, $CDCl_3$): δ_C 205.4 (*C*=O), 170.8 ((*C*=O)Ph), 132.9 (Ar*C*), 131.4 (Ar*C*H), 128.6 (Ar*C*H), 128.0 (Ar*C*H), 53.9 (N*C*H$_2$), 50.7 (N*C*H$_2$), 40.0 (*C*H(C=O)), 27.9 (*C*H$_3$) ppm.

Doubling of azetidine signals due to rotamers.

HRMS (ESI$^+$): m/z calc'd for $C_{12}H_{14}NO_2$ $[M+H]^+$: 204.1019, found: 204.1020.

IR (film): ν_{max} 2954, 2885, 1713 (C=O), 1631 (C=O), 1575, 1450, 1418, 1362, 1177, 712 cm^{-1}.

1-(1-(4-Fluorobenzoyl)azetidin-3-yl)ethan-1-one (5k)

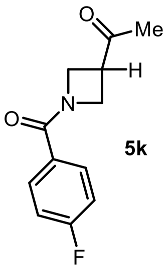

5k

Synthesised following General Procedure C using: acetyltrimethylsilane (46.2 µL, 0.322 mmol, 1.00 equiv) and 4-fluorobenzoyl chloride (0.109 mL, 2.80 equiv). Purified by flash column chromatography (SiO_2; 20:80 hexane:EtOAc) to afford **5k** (49.8 mg, 0.225 mmol, 70%) as a colourless oil.

TLC: R_f = 0.23 (20:80 hexane:EtOAc).

NMR Spectroscopy (*see spectra*):

^{1}H NMR (500 MHz, $CDCl_3$): δ_H 7.68 – 7.59 (m, 2H, ArC*H*), 7.14 – 7.03 (m, 2H, ArC*H*), 4.51 (br. s, 1H, NC*H*$_2$), 4.46 – 4.15 (m, 3H, NC*H*$_2$), 3.56 (tt, *J* = 9.1, 6.0 Hz, 1H, C*H*(C=O)), 2.21 (s, 3H, C*H*$_3$) ppm;

^{13}C NMR (126 MHz, $CDCl_3$): δ_C 205.3 (*C*=O), 169.7 ((*C*=O)Ar), 164.6 (d, *J* = 252.0 Hz, Ar*C*F), 130.4 (d, *J* = 8.8 Hz, Ar*C*H), 129.0 (d, *J* = 3.2 Hz, Ar*C*), 115.7 (d, *J* = 21.8 Hz, Ar*C*H), 53.9 (N*C*H$_2$), 50.8 (N*C*H$_2$), 39.9 (*C*H(C=O)), 28.0 (*C*H$_3$) ppm.

^{19}F NMR (376 MHz, $CDCl_3$): δ_F –107.9 ppm.

Doubling of azetidine signals due to rotamers.

HRMS (ESI^+): m/z calc'd for $C_{12}H_{13}NFO_2$ $[M+H]^+$: 222.0925, found: 222.0926.

IR (film): ν_{max} 2955, 2886, 1712 (C=O), 1627 (C=O), 1510, 1426, 1362, 1226, 1157, 851, 759 cm^{-1}.

1-(1-(2,4-Dinitrophenyl)azetidin-3-yl)ethan-1-one (5l)

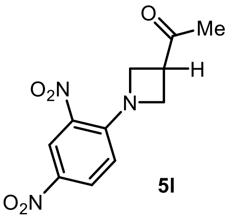

5l

Synthesised following a modified General Procedure C using: **7a′** (51.0 mg, 0.322 mmol, 1.00 equiv) and 1-fluoro-2,4-dinitrobenzene (0.173 mL, 4.20 equiv). After addition of the aryl halide the reaction was allowed to warm to room temperature over 16 h. The intermediate triethylsilyl enol ether required 0.1 M aq. HCl (2.8 equiv) and warming to room temperature for 4 h to achieve complete hydrolysis. Purified by flash column chromatography (SiO_2; 55:45 hexane:EtOAc) to afford **5l** (41.1 mg, 0.155 mmol, 48%) as a yellow oil.

TLC: R_f = 0.20 (55:45 hexane:EtOAc).

NMR Spectroscopy (*see spectra*):

^{1}**H NMR** (500 MHz, $CDCl_3$): δ_H 8.74 (d, *J* = 2.6 Hz, 1H, ArC*H*), 8.20 (dd, *J* = 9.4, 2.6 Hz, 1H, ArC*H*), 6.61 (d, *J* = 9.4 Hz, 1H, ArC*H*), 4.34 (dd, *J* = 10.2, 9.0 Hz, 2H, NCH_2), 4.25 (dd, *J* = 10.2, 6.0 Hz, 2H, NCH_2), 3.70 (tt, *J* = 9.0, 6.0 Hz, 1H, C*H*(C=O)), 2.24 (s, 3H, CH_3) ppm;

^{13}**C NMR** (126 MHz, $CDCl_3$): δ_C 204.6 (*C*=O), 147.3 (Ar*C*), 136.8 (Ar*C*), 133.5 (Ar*C*), 128.2 (Ar*C*H), 123.9 (Ar*C*H), 115.0 (Ar*C*H), 55.1 (2×N*C*H_2), 39.5 (*C*H(C=O)), 28.3 (*C*H_3) ppm.

HRMS (MALDI): m/z calc'd for $C_{11}H_{11}N_3NaO_5$ $[M+Na]^+$: 288.0591, found: 288.0587.

IR (film): ν_{max} 3092, 2940, 2877, 1704 (C=O), 1609, 1523 (N=O), 1502 (N=O), 1325, 1139, 742 cm^{-1}.

1-(1-(5-Fluoropyrimidin-2-yl)azetidin-3-yl)ethan-1-one (5m)

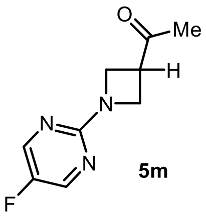

5m

Synthesised following a modified **General Procedure C** using: **7a′** (51.0 mg, 0.322 mmol, 1.00 equiv) and 2-chloro-5-fluoropyrimidine (0.127 mL, 4.20 equiv). After addition of the aryl halide the reaction was allowed to warm to room temperature over 16 h. The intermediate triethylsilyl enol ether required warming to room temperature for 4 h to achieve complete hydrolysis. Purified by flash column chromatography (SiO_2; 55:45 hexane:EtOAc) to afford **5m** (12.6 mg, 0.0646 mmol, 20%) as an orange oil.

TLC: R_f = 0.26 (55:45 hexane:EtOAc).

NMR Spectroscopy (*see spectra*):

^{1}**H NMR** (400 MHz, $CDCl_3$): δ_H 8.21 (s, 2H, ArC*H*), 4.32 – 4.16 (m, 4H, 2×NCH_2), 3.62 (tt, *J* = 8.4, 6.4 Hz, 1H, C*H*(C=O)), 2.23 (s, 3H, CH_3) ppm;

^{13}**C NMR** (126 MHz, $CDCl_3$): δ_C 206.2 (*C*=O), 160.2 (Ar*C*), 152.6 (d, *J* = 248.9 Hz, Ar*C*F), 145.6 (d, *J* = 21.9 Hz, Ar*C*H), 52.1 (2×N*C*H_2), 40.3 (*C*H(C=O)), 28.0 (*C*H_3) ppm.

^{19}**F NMR** (376 MHz, $CDCl_3$): δ_F –155.2 ppm.

HRMS (ESI^+): m/z calc'd for $C_9H_{11}FN_3O$ $[M+H]^+$: 196.0881, found: 196.0882.

IR (film): ν_{max} 2958, 2881, 1714 (C=O), 1558, 1502, 1474, 1386, 931, 787 cm^{-1}.

1-(1-(6-(Trifluoromethyl)pyridin-2-yl)azetidin-3-yl)ethan-1-one (5n)

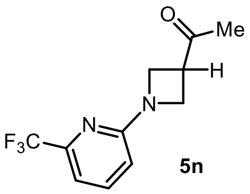

5n

Synthesised following a modified **General Procedure C** using: **7a′** (51.0 mg, 0.322 mmol, 1.00 equiv) and 2-fluoro-6-(trifluoromethyl)pyridine (0.163 mL, 4.20 equiv). After addition of the aryl halide the reaction was allowed to warm to room temperature over 16 h. The intermediate triethylsilyl enol ether required warming to room temperature for 4 h to achieve complete hydrolysis. Purified by flash column chromatography (SiO_2; 70:30 hexane:EtOAc) to afford **5n** (21.8 mg, 0.0893 mmol, 28%) as a colourless oil.

TLC: R_f = 0.24 (70:30 hexane:EtOAc).

NMR Spectroscopy (*see spectra*):

^{1}H NMR (500 MHz, $CDCl_3$): δ_H 7.55 (dd, *J* = 8.4, 7.4, 1H, ArC*H*), 6.97 (d, *J* = 7.4 Hz, 1H, ArC*H*), 6.44 (d, *J* = 8.4 Hz, 1H, ArC*H*), 4.25 – 4.15 (m, 4H, 2×NCH_2), 3.65 (tt, *J* = 8.6, 6.3 Hz, 1H, C*H*(C=O)), 2.23 (s, 3H, CH_3) ppm;

^{13}C NMR (126 MHz, $CDCl_3$): δ_C 206.1 (*C*=O), 160.3 (Ar*C*), 146.8 (q, *J* = 33.9 Hz, Ar*C*(CF_3)), 138.0 (Ar*C*H), 121.8 (q, *J* = 274.0 Hz, *C*F_3), 109.5 (q, *J* = 3.3 Hz, Ar*C*H), 109.0 (Ar*C*H), 52.1 (2×N*C*H_2), 40.8 (*C*H(C=O)), 27.8 (*C*H_3) ppm.

^{19}F NMR (376 MHz, $CDCl_3$): δ_F –68.6 ppm.

HRMS (ESI^+): m/z calc'd for $C_{11}H_{12}N_2F_3O$ $[M+H]^+$: 245.0896, found: 245.0897.

IR (film): ν_{max} 2958, 2877, 1716 (C=O), 1605, 1475, 1363, 1329, 1189, 1131, 801 cm^{-1}.

1-(1-(4-Cyanopyridin-2-yl)azetidin-3-yl)ethan-1-one (5o)

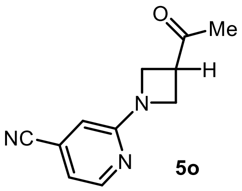

5o

Synthesised following a modified **General Procedure C** using: **7a′** (51.0 mg, 0.322 mmol, 1.00 equiv) and 4-cyano-2-fluoropyridine (169 mg, 4.20 equiv). After addition of the aryl halide the reaction was allowed to warm to room temperature over 16 h. The intermediate triethylsilyl enol ether required warming to room temperature for 4 h to achieve complete hydrolysis. Purified by flash column chromatography (SiO_2; 40:60 hexane:EtOAc) to afford **5o** (29.8 mg, 0.148 mmol, 46%) as a white solid.

TLC: R_f = 0.23 (40:60 hexane:EtOAc).

NMR Spectroscopy (*see spectra*):

^{1}H NMR (500 MHz, $CDCl_3$): δ_H 8.24 (d, *J* = 5.1 Hz, 1H, ArC*H*), 6.77 (d, *J* = 5.1 Hz, 1H, ArC*H*), 6.46 (s, 1H, ArC*H*), 4.30 – 4.09 (m, 4H, 2×NCH_2), 3.68 (tt, *J* = 8.7, 6.1 Hz, 1H, C*H*(C=O)), 2.23 (s, 3H, CH_3) ppm;

^{13}C NMR (126 MHz, $CDCl_3$): δ_C 205.7 (*C*=O), 159.9 (Ar*C*), 149.7 (Ar*C*H), 121.2 (Ar*C*CN), 117.2 (*C*N), 113.8 (Ar*C*H), 107.9 (Ar*C*H), 51.9 (2×N*C*H_2), 40.6 (*C*H(C=O)), 28.0 (*C*H_3) ppm.

HRMS (ESI^+): m/z calc'd for $C_{11}H_{12}N_3O$ $[M+H]^+$: 202.0975, found: 202.0975.

IR (film): ν_{max} 2957, 2876, 2235 (C≡N) 1713 (C=O), 1595, 1540, 1476, 1364, 1296, 1174, 904, 726 cm^{-1}.

***tert*-Butyl 3-acetyl-3-bromoazetidine-1-carboxylate (8a)**

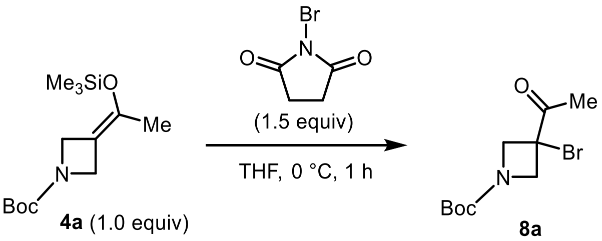

4a (1.0 equiv) **8a**

To a solution of crude **4a** (0.20 mmol, 1.0 equiv) in THF (2 mL) at 0 °C was added N-bromosuccinimide (53.4 mg, 1.50 equiv) and the resulting mixture was stirred for 1 h at 0 °C. H_2O (5 mL) was added and the mixture was extracted with EtOAc (3× 10 mL). The combined organic phases were dried ($MgSO_4$), filtered, concentrated under reduced pressure and the residue was purified by flash column chromatography (SiO_2; 80:20 hexane:EtOAc) to afford **8a** (45.6 mg, 0.164 mmol, 82%) as a colourless oil.

TLC: R_f = 0.29 (80:20 hexane:EtOAc).

NMR Spectroscopy (*see spectra*):

^{1}H NMR (500 MHz, $CDCl_3$): δ_H 4.59 (d, *J* = 10.3 Hz, 2H, NCH_2), 4.23 (d, *J* = 10.3 Hz, 2H, NCH_2), 2.39 (s, 3H, CH_3), 1.44 (s, 9H, OC(CH_3)$_3$) ppm;

^{13}C NMR (126 MHz, $CDCl_3$): δ_C 199.5 (*C*=O), 156.0 ((*C*=O)OC(CH_3)$_3$), 80.8 (O*C*(CH_3)$_3$), 60.8 (2×N*C*H_2), 52.0 (*C*Br), 28.4 (OC(*C*H_3)$_3$), 24.3 (*C*H_3) ppm.

HRMS (ESI^+): m/z calc'd for $C_{10}H_{16}NO_3NaBr$ $[M+Na]^+$: 300.0211, found: 300.0207.

IR (film): ν_{max} 2977, 1707 (C=O), 1697 (C=O), 1388, 1365, 1249, 1147 cm^{-1}.

tert-Butyl 3-acetyl-3-chloroazetidine-1-carboxylate (8b)

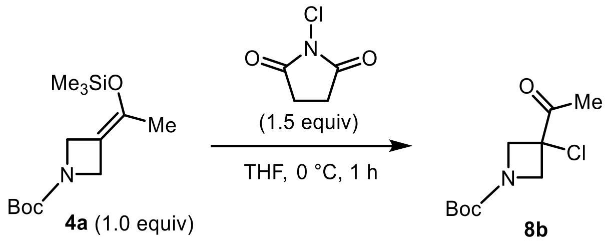

To a solution of crude **4a** (0.20 mmol, 1.0 equiv) in THF (2 mL) at 0 °C was added N-chlorosuccinimide (40.1 mg, 1.50 equiv) and the resulting mixture was stirred for 1 h at 0 °C. H_2O (5 mL) was added and the mixture was extracted with EtOAc (3× 10 mL). The combined organic phases were dried ($MgSO_4$), filtered, concentrated under reduced pressure and the residue was purified by flash column chromatography (SiO_2; 82.5:17.5 hexane:EtOAc) to afford **8b** (21.7 mg, 0.0929 mmol, 46%) as a colourless oil.

TLC: R_f = 0.27 (82.5:17.5 hexane:EtOAc).

NMR Spectroscopy (*see spectra*):

^{1}**H NMR** (500 MHz, $CDCl_3$): δ_H 4.50 (d, *J* = 9.8 Hz, 2H, NC*H*$_2$), 4.10 (d, *J* = 9.8 Hz, 2H, NC*H*$_2$), 2.36 (s, 3H, C*H*$_3$), 1.44 (s, 9H, OC(C*H*$_3$)$_3$) ppm;

^{13}**C NMR** (126 MHz, $CDCl_3$): δ_C 200.1 (*C*=O), 155.9 ((*C*=O)OC(CH$_3$)$_3$), 80.8 (O*C*(CH$_3$)$_3$), 62.0 (*C*Cl), 60.5 (2×N*C*H$_2$), 28.4 (OC(*C*H$_3$)$_3$), 24.6 (*C*H$_3$) ppm.

HRMS (EI$^+$): m/z calc'd for $C_{10}H_{16}NO_3$ [M-Cl]$^+$: 198.1125, found: 198.1125.

IR (film): ν_{max} 2977, 1709 (C=O), 1393, 1253, 1153 cm^{-1}.

tert-Butyl 3-acetyl-3-fluoroazetidine-1-carboxylate (8c)

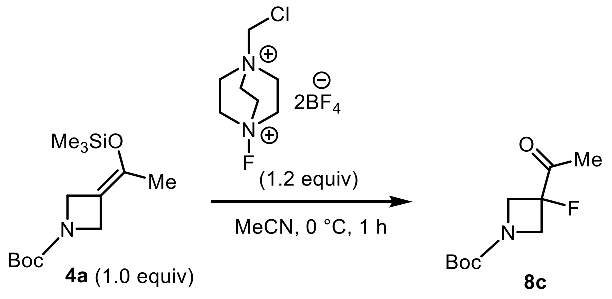

To a solution of crude **4a** (0.20 mmol, 1.0 equiv) in MeCN (2 mL) at 0 °C was added Selectfluor™ (85.0 mg, 1.20 equiv) and the resulting mixture was stirred for 1 h at 0 °C. H_2O (5 mL) was added and the mixture was extracted with EtOAc (3× 10 mL). The combined organic phases were dried ($MgSO_4$), filtered, concentrated under reduced pressure and the residue was purified by flash column chromatography (SiO_2; 80:20 hexane:EtOAc) to afford **8c** (28.4 mg, 0.131 mmol, 65%) as a colourless oil.

TLC: R_f = 0.24 (80:20 hexane:EtOAc).

NMR Spectroscopy (see spectra):

^{1}H NMR (500 MHz, $CDCl_3$): δ_H 4.25 (dd, J = 18.8, 10.0 Hz, 2H, NCH_2), 4.07 (ddd, J = 21.7, 10.0, 1.4 Hz, 2H, NCH_2), 2.31 (d, J = 4.4 Hz, 3H, CH_3), 1.45 (s, 9H, OC(CH_3)$_3$) ppm;

^{13}C NMR (126 MHz, $CDCl_3$): δ_C 204.6 (d, J = 31.3 Hz, *C*=O), 156.0 ((*C*=O)OC(CH_3)$_3$), 93.0 (d, J = 220.2 Hz, *C*F), 80.7 (O*C*(CH_3)$_3$), 58.5 (br., 2×NCH_2), 28.4 (OC(CH_3)$_3$), 25.6(CH_3) ppm.

^{19}F NMR (376 MHz, $CDCl_3$): δ_F –162.3 ppm.

HRMS (ESI$^+$): m/z calc'd for $C_{10}H_{16}NNaO_3F$ [M+Na]$^+$: 240.1012, found: 240.1013.

IR (film): ν_{max} 2978, 1707 (C=O), 1393, 1367, 1288, 1174, 1065 cm^{-1}.

***tert*-Butyl 3-acetyl-3-(phenylselanyl)azetidine-1-carboxylate (8d)**

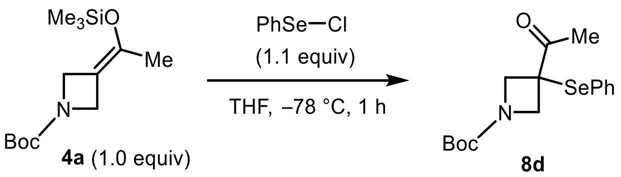

To a solution of crude **4a** (0.20 mmol, 1.0 equiv) in THF (1 mL) at −78 °C was added PhSeCl (42.1 mg, 1.10 equiv) in THF (1 mL) dropwise and the resulting mixture was stirred for 1 h at −78 °C. H_2O (5 mL) was added and the mixture was extracted with EtOAc (3× 10 mL). The combined organic phases were dried ($MgSO_4$), filtered, concentrated under reduced pressure and the residue was purified by flash column chromatography (SiO_2; 80:20 hexane:EtOAc) to afford **8d** (58.0 mg, 0.164 mmol, 82%) as a white solid.

TLC: R_f = 0.26 (80:20 hexane:EtOAc).

NMR Spectroscopy (see spectra):

^{1}H NMR (500 MHz, $CDCl_3$): δ_H 7.48 – 7.43 (m, 2H, ArC*H*), 7.40 – 7.35 (m, 1H, ArC*H*), 7.33 – 7.29 (m, 2H, ArC*H*), 4.36 (d, J = 9.4 Hz, 2H, NCH_2), 4.02 (d, J = 9.4 Hz, 2H, NCH_2), 2.31 (s, 3H, CH_3), 1.42 (s, 9H, OC(CH_3)$_3$) ppm;

^{13}C NMR (126 MHz, $CDCl_3$): δ_C 202.7 (*C*=O), 156.3 ((*C*=O)OC(CH_3)$_3$), 135.6 (Ar*C*), 129.6 (Ar*C*H), 129.6 (Ar*C*H), 126.5 (Ar*C*H), 80.4 (O*C*(CH_3)$_3$), 57.7 (br., 2×NCH_2), 47.9 (*C*SePh), 28.4 (OC(CH_3)$_3$), 24.7 (CH_3) ppm.

HRMS (ESI$^+$): m/z calc'd for $C_{16}H_{21}NNaO_3Se$ [M+Na]$^+$: 378.0584, found: 378.0583.

IR (film): ν_{max} 2976, 2883, 1700 (C=O), 1392, 1366, 1248, 1144 cm^{-1}.

tert-Butyl 3-acetyl-3-(methylthio)azetidine-1-carboxylate (8e)

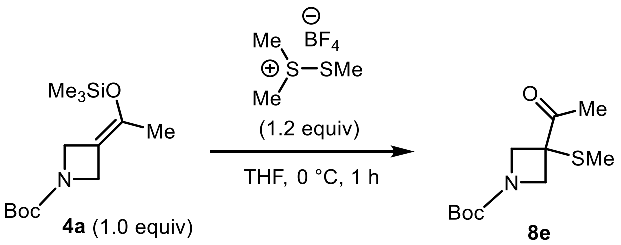

To a solution of crude **4a** (0.20 mmol, 1.0 equiv) in THF (2 mL) at 0 °C was added dimethyl(methylthio)sulfonium tetrafluoroborate (47.1 mg, 1.20 equiv) and the resulting mixture was stirred for 1 h at 0 °C. H_2O (5 mL) was added and the mixture was extracted with EtOAc (3× 10 mL). The combined organic phases were dried ($MgSO_4$), filtered, concentrated under reduced pressure and the residue was purified by flash column chromatography (SiO_2; 80:20 hexane:EtOAc) to afford **8e** (36.5 mg, 0.149 mmol, 74%) as a colourless oil.

TLC: R_f = 0.26 (80:20 hexane:EtOAc).

NMR Spectroscopy (see spectra):

> **^{1}H NMR** (500 MHz, $CDCl_3$): δ_H 4.29 (d, J = 9.1 Hz, 2H, NCH_2), 3.85 (d, J = 9.1 Hz, 2H, NCH_2), 2.26 (s, 3H, CH_3), 1.92 (s, 3H, SCH_3), 1.43 (s, 9H, OC(CH_3)$_3$) ppm;

> **^{13}C NMR** (126 MHz, $CDCl_3$): δ_C 202.4 (*C*=O), 156.2 ((*C*=O)OC(CH$_3$)$_3$), 80.4 (O*C*(CH$_3$)$_3$), 56.1 (br., 2×N*C*H$_2$), 50.6 (*C*SCH$_3$), 28.4 (OC(*C*H$_3$)$_3$), 23.8 (*C*H$_3$), 12.5 (S*C*H$_3$) ppm.

HRMS (ESI$^+$): m/z calc'd for $C_{11}H_{19}NNaO_3S$ [M+Na]$^+$: 268.0983, found: 268.0977.

IR (film): ν_{max} 2976, 2924, 2884, 1703 (C=O), 1695 (C=O), 1391, 1366, 1250, 1139 cm^{-1}.

tert-Butyl 3-acetyl-3-((trimethylsilyl)oxy)azetidine-1-carboxylate (8f)

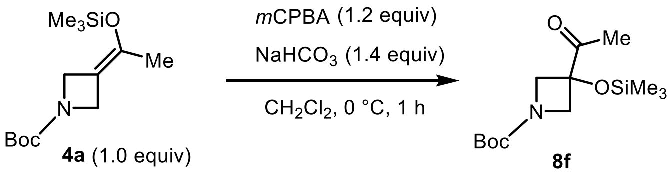

To a suspension of crude **4a** (0.20 mmol, 1.0 equiv) and $NaHCO_3$ (23.5 mg, 1.40 equiv) in CH_2Cl_2 (2 mL) at 0 °C was added *meta*-chloroperoxybenzoic acid (75% by weight, 55.2 mg, 1.20 equiv) and the resulting mixture was stirred for 1 h at 0 °C. The reaction was then quenched with sat. aq. $Na_2S_2O_3$ (4 mL) and the mixture was extracted with CH_2Cl_2 (3× 10 mL). The combined organic phases were dried ($MgSO_4$), filtered, concentrated under reduced pressure and the residue was purified by flash column chromatography (SiO_2; 85:15 hexane:EtOAc) to afford **8f** (43.2 mg, 0.150 mmol, 75%) as a colourless oil.

TLC: R_f = 0.26 (85:15 hexane:EtOAc).

NMR Spectroscopy (see spectra):

^{1}H NMR (500 MHz, $CDCl_3$): δ_H 4.19 (d, *J* = 9.2 Hz, 2H, NC*H*$_2$), 3.88 (d, *J* = 9.2 Hz, 2H, NC*H*$_2$), 2.25 (s, 3H, C*H*$_3$), 1.44 (s, 9H, OC(C*H*$_3$)$_3$), 0.18 (s, 9H, Si(C*H*$_3$)$_3$) ppm;

^{13}C NMR (126 MHz, $CDCl_3$): δ_C 207.0 (*C*=O), 156.3 ((*C*=O)OC(CH$_3$)$_3$), 80.2 (O*C*(CH$_3$)$_3$), 76.1 (*C*OSi), 59.6 (br., 2×N*C*H$_2$), 28.5 (OC(*C*H$_3$)$_3$), 24.2 (*C*H$_3$), 1.5 (Si(*C*H$_3$)$_3$) ppm.

HRMS (ESI$^+$): m/z calc'd for $C_{13}H_{25}NNaO_4Si$ [M+Na]$^+$: 310.1451, found: 310.1443.

IR (film): ν_{max} 2967, 1708 (C=O), 1393, 1367, 1255, 1165, 1131, 844 cm^{-1}.

***tert*-Butyl 3-acetyl-3-hydroxyazetidine-1-carboxylate (8g)**

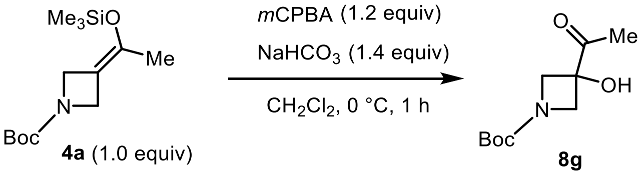

To a suspension of crude **4a** (0.20 mmol, 1.0 equiv) and $NaHCO_3$ (23.5 mg, 1.40 equiv) in CH_2Cl_2 (2 mL) at 0 °C was added *meta*-chloroperoxybenzoic acid (75% by weight, 55.2 mg, 1.20 equiv) and the resulting mixture was stirred for 1 h at 0 °C. The reaction was then quenched with sat. aq. $Na_2S_2O_3$ (4 mL) and the mixture was extracted with CH_2Cl_2 (3× 10 mL). The combined organic phases were then washed with 1 M aq. HCl (3× 10 mL) shaking vigorously for 5 min each time then dried ($MgSO_4$), filtered and concentrated under reduced pressure. The residue was purified by flash column chromatography (SiO_2; 40:60 EtOAc:hexane) to afford **8g** (28.8 mg, 0.123 mmol, 62%) as a colourless oil.

TLC: R_f = 0.28 (40:60 EtOAc:hexane).

NMR Spectroscopy (*see spectra*):

^{1}H NMR (400 MHz, $CDCl_3$): δ_H 4.28 (s, 1H, O*H*), 4.14 (d, *J* = 9.9 Hz, 2H, NC*H*$_2$), 4.09 (d, *J* = 9.9 Hz, 2H, NC*H*$_2$), 2.48 (s, 3H, C*H*$_3$), 1.47 (s, 9H, OC(C*H*$_3$)$_3$) ppm;

^{13}C NMR (126 MHz, $CDCl_3$): δ_C 207.0 (*C*=O), 156.6 ((*C*=O)OC(CH$_3$)$_3$), 80.7 (O*C*(CH$_3$)$_3$), 73.7 (*C*OH), 60.0 (br., 2×N*C*H$_2$), 28.5 (OC(*C*H$_3$)$_3$), 22.6 (*C*H$_3$) ppm.

HRMS (ESI$^+$): m/z calc'd for $C_{10}H_{17}NNaO_4$ [M+Na]$^+$: 238.1055, found: 238.1048.

IR (film): ν_{max} 3389 (O-H), 2978, 2877, 1705 (C=O), 1673 (C=O), 1420, 1368, 1163 cm^{-1}.

tert-Butyl 3-acetyl-3-(hydroxymethyl)azetidine-1-carboxylate (8h)

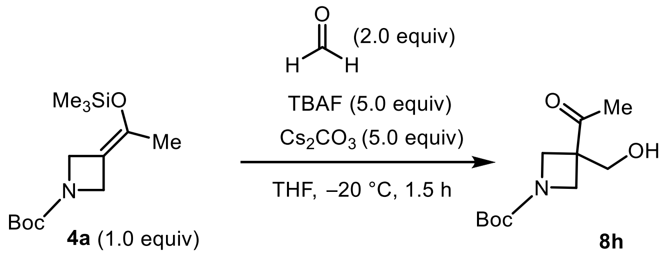

To a suspension of crude **4a** (0.20 mmol, 1.0 equiv) and Cs_2CO_3 (326 mg, 5.00 equiv) in THF (2 mL) at −20 °C was added formaldehyde (37 wt. % in H_2O, 29.8 µL, 2.00 equiv) and the resulting mixture was stirred for 30 min at −20 °C. To this was added tetra-*n*-butylammonium fluoride (1.0 M in THF, 1.0 mL, 5.0 equiv) and the solution was stirred for 1 h at −20 °C. Sat. aq. Na_2CO_3 (5 mL) was added, and the mixture was extracted with EtOAc (3× 10 mL). The combined organic phases were dried ($MgSO_4$), filtered, concentrated under reduced pressure and the residue was purified by flash column chromatography (SiO_2; 40:60 hexane:EtOAc) to afford **8h** (37.6 mg, 0.164 mmol, 82%) as a colourless oil.

TLC: R_f = 0.26 (40:60 hexane:EtOAc).

NMR Spectroscopy (_see spectra_):

^{1}H NMR (400 MHz, $CDCl_3$): δ_H 4.08 (d, *J* = 9.0 Hz, 2H, NC*H*$_2$), 3.97 (d, *J* = 5.8 Hz, 2H, C*H*$_2$OH), 3.82 (d, *J* = 9.0 Hz, 2H, NC*H*$_2$), 2.33 (s, 3H, C*H*$_3$), 2.11 (t, *J* = 5.8 Hz, 1H, CH$_2$O*H*), 1.45 (s, 9H, OC(C*H*$_3$)$_3$) ppm;

^{13}C NMR (126 MHz, $CDCl_3$): δ_C 208.8 (*C*=O), 156.5 ((*C*=O)OC(CH$_3$)$_3$), 80.4 (O*C*(CH$_3$)$_3$), 65.7 (*C*H$_2$OH), 53.6 (br., 2×N*C*H$_2$), 49.9 (*C*(C=O)), 28.5 (OC(*C*H$_3$)$_3$), 26.0 (*C*H$_3$) ppm.

HRMS (APCI$^-$): m/z calc'd for $C_{11}H_{19}NO_4$ [M+Cl]$^-$: 264.0997, found: 264.0987.

IR (film): ν_{max} 3409 (O-H), 2976, 2929, 1700 (C=O), 1508, 1414, 1366, 1163, 1044 cm^{-1}.

tert-Butyl 3-acetyl-3-(hydroxy(phenyl)methyl)azetidine-1-carboxylate (8i)

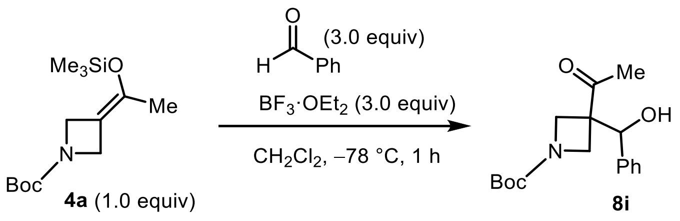

To a solution of crude **4a** (0.20 mmol, 1.0 equiv) and benzaldehyde (61.2 µL, 3.00 equiv) in CH_2Cl_2 (2 mL) at −78 °C was added $BF_3{\cdot}OEt_2$ (74.1 µL, 3.00 equiv) and the resulting mixture was stirred for 1 h at −78 °C. Sat. aq. $NaHCO_3$ (5 mL) was added, and the mixture was extracted with EtOAc (3× 10 mL). The combined organic phases were dried ($MgSO_4$), filtered, concentrated under reduced pressure and the residue was purified by flash column chromatography (SiO_2; 60:40 hexane:EtOAc) to afford **8i** (46.7 mg, 0.153 mmol, 76%) as a white solid.

TLC: R_f = 0.26 (60:40 hexane:EtOAc).

NMR Spectroscopy (*see spectra*):

^{1}H NMR (500 MHz, $CDCl_3$): δ_H 7.38 – 7.26 (m, 5H, ArC*H*), 5.05 (d, *J* = 4.4 Hz, 1H, C*H*OH), 4.17 (d, *J* = 9.1 Hz, 1H, NC*H*$_2$), 4.13 – 4.08 (m, 1H, NC*H*$_2$), 4.06 – 3.98 (m, 2H, NC*H*$_2$), 2.89 (br. s, 1H, CH(O*H*)), 2.14 (s, 3H, C*H*$_3$), 1.39 (s, 9H, OC(C*H*$_3$)$_3$) ppm;

^{13}C NMR (126 MHz, $CDCl_3$): δ_C 209.3 (*C*=O), 156.4 ((*C*=O)OC(CH$_3$)$_3$), 139.5 (Ar*C*), 128.8 (Ar*C*H), 128.8 (Ar*C*H), 126.8 (Ar*C*H), 80.1 (O*C*(CH$_3$)$_3$), 75.5 (*C*H(OH)), 53.7 (*C*(C=O)), 53.2 (br., 2×N*C*H$_2$), 28.4 (OC(*C*H$_3$)$_3$), 27.7 (*C*H$_3$) ppm.

HRMS (ESI$^+$): m/z calc'd for $C_{17}H_{24}NNaO_4$ [M+Na]$^+$: 306.1705, found: 306.1709.

IR (film): ν_{max} 3399 (O-H), 2976, 2891, 1699 (C=O), 1674 (C=O), 1407, 1366, 1251, 1154, 1043, 760 cm^{-1}.

***tert*-Butyl 3-acetyl-3-(1-hydroxypropyl)azetidine-1-carboxylate (8j)**

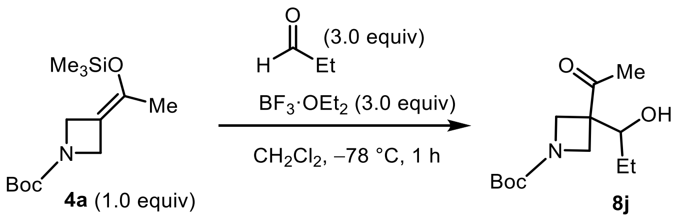

To a solution of crude **4a** (0.20 mmol, 1.0 equiv) and propionaldehyde (43.0 µL, 3.00 equiv) in CH_2Cl_2 (2 mL) at −78 °C was added $BF_3 \cdot OEt_2$ (74.1 µL, 3.00 equiv) and the resulting mixture was stirred for 1 h at −78 °C. Sat. aq. $NaHCO_3$ (5 mL) was added, and the mixture was extracted with EtOAc (3× 10 mL). The combined organic phases were dried ($MgSO_4$), filtered, concentrated under reduced pressure and the residue was purified by flash column chromatography (SiO_2; 55:45 hexane:EtOAc) to afford **8j** (41.5 mg, 0.161 mmol, 81%) as a colourless oil.

TLC: R_f = 0.27 (55:45 hexane:EtOAc).

NMR Spectroscopy (*see spectra*):

^{1}H NMR (500 MHz, $CDCl_3$): δ_H 4.08 (d, *J* = 9.0 Hz, 1H, NC*H*$_2$), 4.03 (d, *J* = 9.0 Hz, 1H, NC*H*$_2$), 4.00 (d, *J* = 9.0 Hz, 1H, NC*H*$_2$), 3.88 – 3.80 (m, 2H, NC*H*$_2$, C*H*OH), 2.27 (br. s, 4H, (C=O)C*H*$_3$, CHO*H*), 1.50 – 1.37 (m, 10H, OC(C*H*$_3$)$_3$, C*H*$_2$CH$_3$), 1.38 – 1.28 (m, 1H, C*H*$_2$CH$_3$), 1.02 (t, *J* = 7.4 Hz, 3H, CH$_2$C*H*$_3$) ppm;

^{13}C NMR (126 MHz, $CDCl_3$): δ_C 209.0 (*C*=O), 156.5 ((*C*=O)OC(CH$_3$)$_3$), 80.2 (O*C*(CH$_3$)$_3$), 75.7 (*C*HOH), 53.5 (br., 2×N*C*H$_2$), 53.1 (*C*(C=O)), 28.5 (OC(*C*H$_3$)$_3$), 27.3 ((C=O)*C*H$_3$), 25.4 (*C*H$_2$CH$_3$), 10.9 (CH$_2$*C*H$_3$) ppm.

HRMS (ESI$^+$): m/z calc'd for $C_{13}H_{23}NNaO_4$ [M+Na]$^+$: 280.1525, found: 280.1528.

IR (film): ν_{max} 3424 (O-H), 2967, 2934, 2886, 1700 (C=O), 1676 (C=O), 1420, 1413, 1366, 1258, 1153, 1102, 1021, 801 cm^{-1}.

tert-Butyl 3-acetyl-3-((dimethylamino)methyl)azetidine-1-carboxylate hydrochloride (8k)

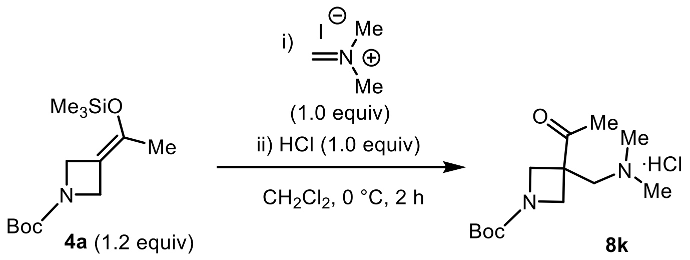

To a solution of crude **4a** (0.24 mmol, 1.2 equiv) in CH_2Cl_2 (2 mL) at −78 °C was added N,N-dimethylmethyleneiminium iodide (37.0 mg, 0.200 mmol, 1.00 equiv) and the resulting mixture was stirred for 2 h at 0 °C. H_2O (5 mL) and 0.5 M NaOH (5 mL) were added, and the mixture was extracted with Et_2O (3× 10 mL). To the combined organic phases was added HCl (2.0 M in Et_2O, 0.10 mL, 1.0 equiv) and the resulting precipitate was filtered and washed with Et_2O (5× 5 mL) to afford **8k** (36.5 mg, 0.125 mmol, 62%) as a white solid.

NMR Spectroscopy (*see spectra*):

^{1}**H NMR** (500 MHz, $CDCl_3$): δ_H 12.59 (s, 1H, N*H*), 4.28 (br. s, 2H, NC*H*$_2$), 3.99 (br. s, 2H, NC*H*$_2$), 3.57 (br. s, 2H, C*H*$_2$N(CH$_3$)$_2$), 2.87 (s, 6H, N(C*H*$_3$)$_2$), 2.63 (s, 3H, C*H*$_3$), 1.46 (s, 9H, OC(C*H*$_3$)$_3$) ppm;

^{13}**C NMR** (126 MHz, $CDCl_3$): δ_C 206.1 (*C*=O), 156.0 ((*C*=O)OC(CH$_3$)$_3$), 81.2 (O*C*(CH$_3$)$_3$), 61.0 (*C*H$_2$N(CH$_3$)$_2$), 55.1 (br., 2×N*C*H$_2$), 47.0 (*C*(C=O)), 46.7 (N(*C*H$_3$)$_2$), 28.5 (OC(*C*H$_3$)$_3$), 25.7 (*C*H$_3$) ppm.

HRMS (ESI$^+$): m/z calc'd for $C_{13}H_{25}N_2O_3$ [M-Cl]$^+$: 257.1865, found: 257.1862.

IR (film): ν_{max} 3411 (N-H), 2971, 2612 (N-H), 1700 (C=O), 1459, 1398, 1367, 1157 cm^{-1}.

tert-Butyl 3-acetyl-3-(cyclohepta-2,4,6-trien-1-yl)azetidine-1-carboxylate (8l)

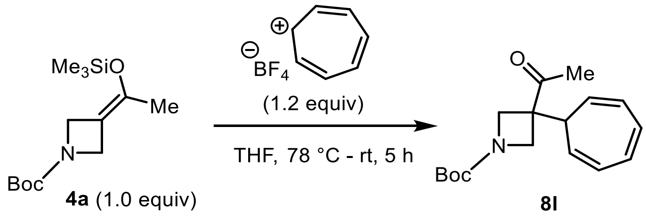

To a solution of crude **4a** (0.20 mmol, 1.0 equiv) in THF (2 mL) at −78 °C was added tropylium tetrafluoroborate (42.7 mg, 1.20 equiv) and the resulting mixture was allowed to warm slowly to room temperature over 5 h. H_2O (5 mL) was added and the mixture was extracted with EtOAc (3× 10 mL). The combined organic phases were dried ($MgSO_4$), filtered, concentrated under reduced pressure and the residue was purified by flash column chromatography (SiO_2; 90:10 toluene:Et_2O) to afford **8l** (39.9 mg, 0.138 mmol, 69%) as a colourless oil.

TLC: R_f = 0.20 (90:10 toluene:Et_2O).

NMR Spectroscopy (*see spectra*):

^{1}**H NMR** (500 MHz, $CDCl_3$): δ_H 6.77 – 6.69 (m, 2H, 2×*H*C=C), 6.35 – 6.27 (m, 2H, 2×*H*C=C), 5.32 – 5.09 (m, 2H, 2×*H*C=C), 4.25 (br. s, 2H, NC*H*$_2$), 3.95 (d, J = 9.0 Hz, 2H, NC*H*$_2$), 2.08 (s, 3H, C*H*$_3$), 1.98 (tt, J =

5.5, 1.5 Hz, 1H, C*H*(C=C)), 1.46 (s, 9H, OC(C*H*$_3$)$_3$) ppm;

^{13}C NMR (126 MHz, CDCl$_3$): δ_C 207.4 (*C*=O), 156.4 ((*C*=O)OC(CH$_3$)$_3$), 131.4 (2×*C*=C), 126.9 (2×*C*=C), 120.6 (2×*C*=C), 80.2 (O*C*(CH$_3$)$_3$), 53.4 (br., 2×N*C*H$_2$), 49.9 (*C*(C=O)), 42.2 (*C*H(C=C)), 28.5 (OC(*C*H$_3$)$_3$), 25.2 (*C*H$_3$) ppm.

HRMS (ESI$^+$): m/z calc'd for $C_{17}H_{23}NNaO_3$ [M+Na]$^+$: 312.1576, found: 312.1575.

IR (film): ν_{max} 2974, 1703 (C=O), 1699 (C=O), 1391, 1365, 1249, 1146, 703 cm^{-1}.

***tert*-Butyl 3-acetyl-3-(benzo[*d*][1,3]dithiol-2-yl)azetidine-1-carboxylate (8m)**

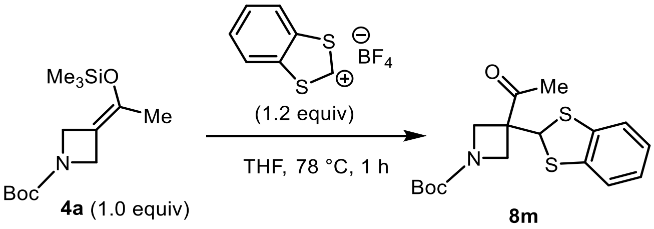

To a solution of crude **4a** (0.20 mmol, 1.0 equiv) in THF (2 mL) at −78 °C was added 1,3-benzodithiolylium tetrafluoroborate (57.6 mg, 1.20 equiv) and the resulting mixture was stirred at −78 °C for 1 h. H_2O (5 mL) was added and the mixture was extracted with EtOAc (3× 10 mL). The combined organic phases were dried ($MgSO_4$), filtered, concentrated under reduced pressure and the residue was purified by flash column chromatography (SiO_2; 87.5:12.5 toluene:Et_2O) to afford **8m** (46.4 mg, 0.132 mmol, 66%) as a colourless oil.

TLC: R_f = 0.20 (87.5:12.5 toluene:Et_2O).

NMR Spectroscopy (*see spectra*):

^{1}H NMR (400 MHz, CDCl$_3$): δ_H 7.19 – 7.12 (m, 2H, ArC*H*), 7.07 – 6.98 (m, 2H, ArC*H*), 5.39 (s, 1H, C*H*(SAr)$_2$), 4.00 (s, 4H, 2×NC*H*$_2$), 2.39 (s, 3H, C*H*$_3$), 1.44 (s, 9H, OC(C*H*$_3$)$_3$) ppm;

^{13}C NMR (126 MHz, CDCl$_3$): δ_C 206.5 (*C*=O), 156.3 ((*C*=O)OC(CH$_3$)$_3$), 136.9 (Ar*C*), 125.9 (Ar*C*H), 122.0 (Ar*C*H), 80.5 (O*C*(CH$_3$)$_3$), 54.9 (*C*(C=O)), 53.8 (*C*H(SAr)$_2$), 53.1 (2×N*C*H$_2$), 28.5 (OC(*C*H$_3$)$_3$), 26.6 (*C*H$_3$) ppm.

HRMS (ESI$^+$): m/z calc'd for $C_{17}H_{22}NNaO_3S_2$ [M+Na]$^+$: 352.1041, found: 352.1035.

IR (film): ν_{max} 2975, 1701 (C=O), 1697 (C=O), 1447, 1391, 1365, 1160, 1120, 744 cm^{-1}.

tert-Butyl 3-acetyl-3-methoxyazetidine-1-carboxylatecarboxylate (8n)

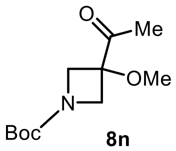

8n

Synthesised following **General Procedure D** using: TsN=IPh (74.6 mg, 1.00 equiv), $BF_3·OEt_2$ (24.7 µL, 1.00 equiv) and MeOH (40.5 µL, 5.00 equiv). Purified by flash column chromatography (SiO_2; 82:18 hexane:EtOAc) to afford **8n** (28.0 mg, 0.122 mmol, 61%) as a colourless oil.

TLC: R_f = 0.20 (82:18 hexane:EtOAc).

NMR Spectroscopy (*see spectra*):

^{1}H NMR (500 MHz, $CDCl_3$): δ_H 4.11 (d, J = 9.4 Hz, 2H, NCH_2), 3.89 (d, J = 9.4 Hz, 2H, NCH_2), 3.26 (s, 3H, OCH_3), 2.22 (s, 3H, CH_3), 1.44 (s, 9H, OC(CH_3)$_3$) ppm;

^{13}C NMR (126 MHz, $CDCl_3$): δ_C 206.4 (*C*=O), 156.4 ((*C*=O)OC(CH_3)$_3$), 80.2 (O*C*(CH_3)$_3$), 79.9 (*C*(C=O)), 54.8 (br., 2×N*C*H_2), 53.1 (O*C*H_3), 28.5 (OC(*C*H_3)$_3$), 24.1 (*C*H_3) ppm.

HRMS (APCI$^+$): m/z calc'd for $C_6H_{11}NO_2$ [M+H–Boc]$^+$: 130.0863, found: 130.0864.

IR (film): ν_{max} 2977, 2939, 2881, 1697 (C=O), 1397, 1365, 1221, 1159 cm^{-1}.

tert-Butyl 3-acetyl-3-(((trifluoromethyl)sulfonyl)oxy)azetidine-1-carboxylate (8o)

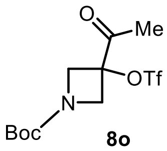

8o

Synthesised following **General Procedure D** using: TsN=IPh (74.6 mg, 1.00 equiv) and $Cu(OTf)_2$ (72.3 mg, 1.00 equiv). Purified by flash column chromatography (SiO_2; 75:25 hexane:EtOAc) to afford **8o** (43.1 mg, 0.124 mmol, 62%) as a white solid.

TLC: R_f = 0.25 (75:25 hexane:EtOAc).

NMR Spectroscopy (*see spectra*):

^{1}H NMR (500 MHz, $CDCl_3$): δ_H 4.38 (d, J = 10.9 Hz, 2H, NCH_2), 4.30 (d, J = 10.9 Hz, 2H, NCH_2), 2.47 (s, 3H, CH_3), 1.47 (s, 9H, OC(CH_3)$_3$) ppm;

^{13}C NMR (126 MHz, $CDCl_3$): δ_C 198.1 (*C*=O), 155.8 ((*C*=O)OC(CH_3)$_3$), 118.2 (q, J = 319.6 Hz, *C*F_3), 86.8 (*C*(C=O)), 81.7 (O*C*(CH_3)$_3$), 57.3 (br., 2×N*C*H_2), 28.3 (OC(*C*H_3)$_3$), 24.0 (*C*H_3) ppm.

^{19}F NMR (376 MHz, $CDCl_3$): δ_F –74.9 ppm.

HRMS (APCI$^+$): m/z calc'd for $C_6H_8NO_4SF_3$ [M+H–Boc]$^+$: 248.0199, found: 248.0201.

IR (film): ν_{max} 2985, 2937, 1733 (C=O), 1693 (C=O), 1402 (S=O), 1369, 1207, 1142, 947, 906 cm^{-1}.

tert-Butyl 3-acetyl-3-(2-oxo-2-phenylethyl)azetidine-1-carboxylate (8p)

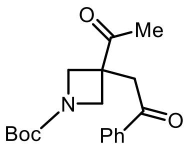

8p

Synthesised following **General Procedure D** using: CAN (219 mg, 2.00 equiv), 1-phenyl-1-trimethylsiloxyethylene (0.410 mL, 10.0 equiv) and $NaHCO_3$ (67.2 mg, 4.00 equiv). Purified by flash column chromatography (SiO_2; 99:2 to 90:10 CH_2Cl_2:Et_2O) to afford **8p** (45.8 mg, 0.144 mmol, 72%) as a white solid.

TLC: R_f = 0.30 (90:10 CH_2Cl_2:Et_2O).

NMR Spectroscopy (_see spectra_):

^{1}**H NMR** (400 MHz, $CDCl_3$): δ_H 7.96 – 7.88 (m, 2H, ArC*H*), 7.61 – 7.56 (m, 1H, ArC*H*), 7.52 – 7.44 (m, 2H, ArC*H*), 4.26 (d, *J* = 9.1 Hz, 2H, NCH_2), 3.86 (d, *J* = 9.1 Hz, 2H, NCH_2), 3.71 (br. s, 2H, CH_2(C=O)), 2.48 (s, 3H, CH_3), 1.45 (s, 9H, OC(CH_3)$_3$) ppm;

^{13}**C NMR** (126 MHz, $CDCl_3$): δ_C 207.4 (*C*=O), 197.4 (Ph(*C*=O)), 156.3 ((*C*=O)OC(CH$_3$)$_3$), 136.2 (Ar*C*), 133.8 (Ar*C*H), 128.9 (Ar*C*H), 128.2 (Ar*C*H), 80.3 (O*C*(CH$_3$)$_3$), 56.8 (br., 2×N*C*H$_2$), 45.5 (*C*H$_2$(C=O)), 45.1 (*C*(C=O)), 28.5 (OC(*C*H$_3$)$_3$), 25.7 (*C*H$_3$) ppm.

HRMS (ESI$^+$): m/z calc'd for $C_{18}H_{23}NNaO_4$ $[M+Na]^+$: 340.1519, found: 340.1520.

IR (film): ν_{max} 2974, 1697 (C=O), 1449, 1397, 1365, 1221, 1159 cm^{-1}.

tert-Butyl 3-acetyl-3-azidoazetidine-1-carboxylate carboxylate (8q)

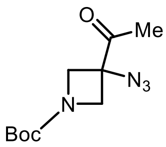

8q

Synthesised following **General Procedure D** using: CAN (219 mg, 2.00 equiv), sodium azide (39.0 mg, 3.00 equiv) and $NaHCO_3$ (67.2 mg, 4.00 equiv). Purified by flash column chromatography (SiO_2; 82:18 hexane:EtOAc) to afford **8q** (28.6 mg, 0.119 mmol, 60%) as a white solid.

TLC: R_f = 0.20 (82:18 hexane:EtOAc).

NMR Spectroscopy (_see spectra_):

^{1}**H NMR** (500 MHz, $CDCl_3$): δ_H 4.27 (d, *J* = 9.5 Hz, 2H, NCH_2), 3.98 (d, *J* = 9.5 Hz, 2H, NCH_2), 2.31 (s, 3H, CH_3), 1.45 (s, 9H, OC(CH_3)$_3$) ppm;

^{13}**C NMR** (126 MHz, $CDCl_3$): δ_C 202.3 (*C*=O), 156.0 ((*C*=O)OC(CH$_3$)$_3$), 80.9 (O*C*(CH$_3$)$_3$), 64.1 (*C*N$_3$), 56.5 (2×N*C*H$_2$), 28.4 (OC(*C*H$_3$)$_3$), 25.2 (*C*H$_3$) ppm.

HRMS (APCI$^+$): m/z calc'd for $C_5H_8N_4O$ [M+H–Boc]$^+$: 141.0771, found: 141.0772.

IR (film): ν_{max} 2978, 2114 (N=N=N), 1703 (C=O), 1392, 1367, 1255, 1149 cm^{-1}.

***tert*-Butyl 3-acetamido-3-acetylazetidine-1-carboxylate (8r)**

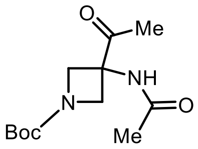

8r

Synthesised following **General Procedure D** using: TsN=IPh (74.6 mg, 1.00 equiv), $TsNH_2$ (171 mg, 5.00 equiv)[A] and $BF_3{\cdot}OEt_2$ (24.7 µL, 1.00 equiv). Purified by flash column chromatography (SiO_2; EtOAc) to afford **8r** (18.1 mg, 0.0706 mmol, 35%) as a colourless oil.

Notes: (**A**) The specific role of $TsNH_2$ is unknown, however the formation of product is severely compromised when this additive is omitted.

TLC: R_f = 0.32 (EtOAc).

NMR Spectroscopy (*see spectra*):

^{1}**H NMR** (500 MHz, $CDCl_3$): δ_H 6.82 – 6.43 (m, 1H, N*H*), 4.32 – 4.22 (m, 2H, NCH_2), 4.21 – 4.08 (m, 2H, NCH_2), 2.36 (s, 3H, CH_3), 2.05 (s, 3H, NH(C=O)CH_3), 1.46 (s, 9H, OC(CH_3)$_3$) ppm.

^{13}**C NMR** (126 MHz, $CDCl_3$): δ_C 204.0 (*C*=O), 170.4 (NH(*C*=O)CH$_3$), 156.7 ((*C*=O)OC(CH$_3$)$_3$), 80.6 (O*C*(CH$_3$)$_3$), 58.3 (*C*(C=O)), 56.1 (2×NCH$_2$), 28.4 (OC(*C*H$_3$)$_3$), 23.8 (*C*H$_3$), 23.4 (NH(C=O)*C*H$_3$) ppm.

HRMS (ESI$^+$): m/z calc'd for $C_{12}H_{20}N_2NaO_4$ [M+Na]$^+$: 279.1315, found: 279.1318.

IR (film): ν_{max} 3287 (N-H), 2977, 2901, 1708 (C=O), 1679 (C=O), 1532, 1408, 1368, 1225, 1154, 1077 cm^{-1}.

2.6. Four-Component One-Pot Synthesis of Azetidines

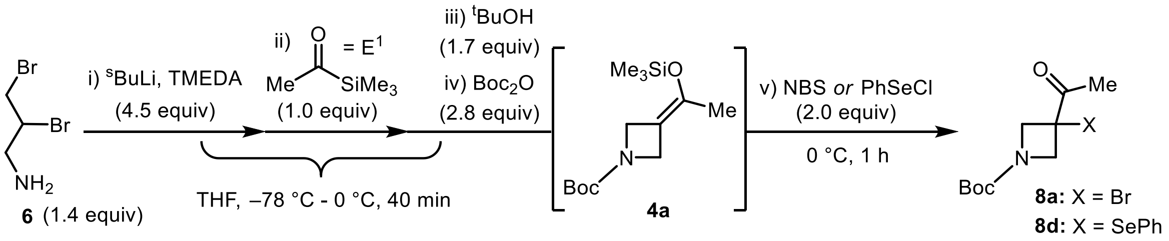

To a solution of freshly made **6** (99.8 mg, 1.40 equiv) and TMEDA (0.221 mL, 4.50 equiv)[A] in THF (2.3 mL) at −78 °C was added ˢBuLi (1.30 M in cyclohexane/hexane (92/8), 1.13 mL, 4.50 equiv)[B] dropwise and the resulting solution was stirred for 15 min at −78 °C. Acetyltrimethylsilane (46.2 µL, 0.322 mmol, 1.00 equiv) was added dropwise and the reaction was stirred for 10 min before the addition of ᵗBuOH (53.4 µL, 1.70 equiv) in THF (0.3 mL). The reaction was stirred for 10 min at −78 °C then warmed to 0 °C. Boc_2O (0.211 mL, 2.80 equiv) was added, and the resulting solution was stirred for 5 min. To this was added either N-bromosuccinimide (115 mg, 2.00 equiv) or PhSeCl (123 mg, 2.00 equiv) and the reaction was stirred at 0°C for a final 1 h.[C] H_2O (10 mL) was then added to quench the reaction, and the mixture was extracted with EtOAc (3× 10 mL). The combined organic phases were dried ($MgSO_4$), filtered, concentrated under reduced pressure and the residue was purified by flash column chromatography on silica gel to yield either **8a** (25.0 mg, 0.0899 mmol, 28%) or **8d** (28.5 mg, 0.0804 mmol, 25%), respectively.[D]

Notes: (**A**) TMEDA should be distilled over CaH_2 prior to use. (**B**) Organolithiums should be carefully titrated prior to use. (**C**) In the case of PhSeCl, the reaction was warmed to room temperature over 16 h. (**D**) For purification and characterisation details see **8a** and **8d**.

2.7. Synthesis of PF-04418948

(4-Fluorophenyl)(3-(1-((trimethylsilyl)oxy)ethylidene)azetidin-1-yl)methanone (4l)

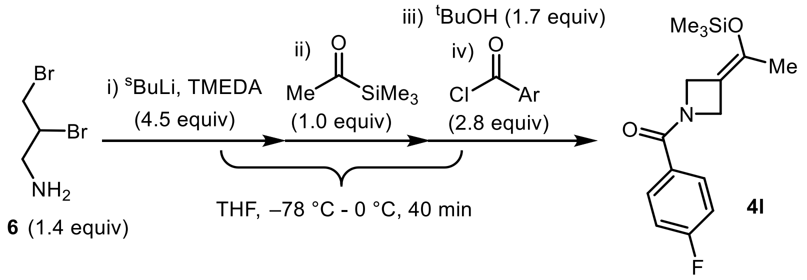

To a solution of freshly made **6** (304 mg, 1.40 equiv) and TMEDA (0.672 mL, 4.50 equiv)[A] in THF (7.0 mL) at −78 °C was added ^{s}BuLi (1.30 M in cyclohexane/hexane (92/8), 3.45 mL, 4.50 equiv)[B] dropwise and the resulting solution was stirred for 15 min at −78 °C. Acetyltrimethylsilane (0.140 mL, 0.980 mmol, 1.00 equiv) was added dropwise and the reaction was stirred for 10 min before the addition of ^{t}BuOH (0.163 mL, 1.70 equiv) in THF (1 mL). The reaction was stirred for 10 min at −78 °C then warmed to 0 °C. 4-Fluorobenzoyl chloride (0.331 mL, 2.80 equiv) was added, and the resulting solution was stirred for 5 min. H_2O (10 mL) was then added to quench the reaction, and the mixture was extracted with EtOAc (3× 10 mL). The combined organic phases were dried ($MgSO_4$), filtered, and concentrated under reduced pressure to yield crude silyl enol ether **4l** (0.686 mmol, 70% as calculated by quantitative ^{1}H NMR using dibromomethane as internal standard) which was used without further purification in the subsequent step.

Notes: (**A**) TMEDA should be distilled over CaH_2 prior to use. (**B**) Organolithiums should be carefully titrated prior to use.

1-(1-(4-Fluorobenzoyl)-3-(hydroxymethyl)azetidin-3-yl)ethan-1-onemethanone (9)

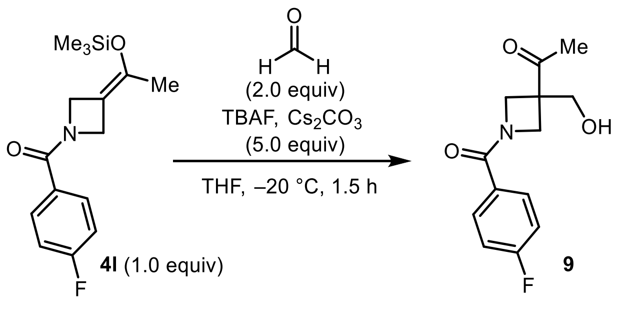

To a suspension of crude **4l** (0.60 mmol, 1.0 equiv) and Cs_2CO_3 (977 mg, 5.00 equiv) in THF (6 mL) at −20 °C was added formaldehyde (37 wt. % in H_2O, 89.3 µL, 2.00 equiv) and the resulting mixture was stirred for 30 min at −20 °C. To this was added tetra-*n*-butylammonium fluoride (1.0 M in THF, 3.0 mL, 5.0 equiv) and the solution was stirred for 1 h at −20 °C. Sat. aq. Na_2CO_3 (15 mL) was added, and the mixture was extracted with EtOAc (3× 20 mL). The combined organic phases were dried ($MgSO_4$), filtered, concentrated under reduced pressure and the residue was purified by flash column chromatography (SiO_2; 10:90 to 0:100 hexane:EtOAc) to afford **9** (125 mg, 0.498 mmol, 83%) as a colourless oil.

TLC: R_f = 0.18 (10:90 hexane:EtOAc).

NMR Spectroscopy (*see spectra*):

^{1}H NMR (400 MHz, $CDCl_3$): δ_H 7.73 – 7.60 (m, 2H, ArC*H*), 7.20 – 7.04 (m, 2H, ArC*H*), 4.52 (br. s, 1H, NC*H*$_2$), 4.28 (d, *J* = 10.4 Hz, 1H, NC*H*$_2$), 4.23 – 3.95 (m, 4H, NC*H*$_2$, C*H*$_2$OH), 2.54 – 2.36 (m, 1H, O*H*), 2.32 (2×s, 3H, C*H*$_3$) ppm;

^{13}C NMR (126 MHz, $CDCl_3$): δ_C 207.6 (*C*=O), 169.8 ((*C*=O)Ar), 164.7 (d, *J* = 252.4 Hz, Ar*C*F), 130.5 (d, *J* = 8.9 Hz, Ar*C*H), 128.7 (d, *J* = 3.2 Hz, Ar*C*), 115.8 (d, *J* = 21.8 Hz, Ar*C*H), 65.4 (*C*H$_2$OH), 57.2 (N*C*H$_2$), 53.1 (N*C*H$_2$), 50.5 (*C*(C=O)), 26.0 (*C*H$_3$) ppm.

^{19}F NMR (376 MHz, $CDCl_3$): δ_F –107.4 ppm.

Doubling of signals due to rotamers.

HRMS (ESI$^+$): m/z calc'd for $C_{13}H_{14}NNaO_3F$ [M+H]$^+$: 252.1030, found: 252.1026.

IR (film): ν_{max} 3381 (O-H), 2950, 2881, 1709 (C=O), 1636 (C=O), 1440, 1350, 1229, 1157, 1046, 851 cm^{-1}.

1-(1-(4-Fluorobenzoyl)-3-(((6-methoxynaphthalen-2-yl)oxy)methyl)azetidin-3-yl)ethan-1-one (10)

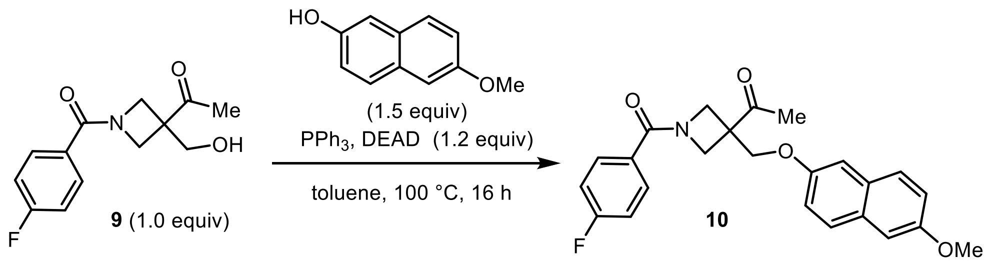

Following a modified literature procedure.[7]

To a solution of **9** (101 mg, 0.400 mmol, 1.00 equiv), PPh_3 (126 mg, 1.20 equiv) and 6-methoxynaphthalen-2-ol (105 mg, 1.50 equiv) in toluene (4 mL) at room temperature was added diethyl azodicarboxylate (DEAD) (75.5 µL, 1.20 equiv) and the resulting mixture was stirred for 16 h at 100 °C. After this time, the reaction was cooled to room temperature, the solvent was removed under reduced pressure and the residue was purified by flash column chromatography (SiO_2; 80:20 to 0:100 hexane:EtOAc) to afford **10** (118 mg, 0.289 mmol, 72%) as a white solid.

TLC: R_f = 0.20 (40:60 hexane:EtOAc).

NMR Spectroscopy (*see spectra*):

^{1}H NMR (400 MHz, $CDCl_3$): δ_H 7.87 – 7.55 (m, 4H, ArC*H*), 7.20 – 6.99 (m, 6H, ArC*H*), 4.74 (d, *J* = 9.5 Hz, 1H, NC*H*$_2$), 4.52 – 4.42 (m, 2H, C*H*$_2$OAr), 4.37 (d, *J* = 9.5 Hz, 1H, NC*H*$_2$), 4.26 (d, *J* = 9.5 Hz, 2H, NC*H*$_2$), 3.90 (s, 3H, OC*H*$_3$), 2.35 (s, 3H, C*H*$_3$) ppm;

^{13}C NMR (126 MHz, $CDCl_3$): δ_C 205.7 (*C*=O), 169.9 ((*C*=O)Ar), 164.7 (d, *J* = 252.2 Hz, Ar*C*F), 156.7 (Ar*C*O), 154.6 (Ar*C*O), 130.5 (d, *J* = 9.1 Hz, Ar*C*H), 130.4 (Ar*C*), 129.6 (Ar*C*), 128.8 (d, *J* = 3.2 Hz, Ar*C*),

128.7 (Ar*C*H), 128.4 (Ar*C*H), 119.5 (Ar*C*H), 118.7 (Ar*C*H), 115.8 (d, *J* = 21.8 Hz, Ar*C*H), 107.6 (Ar*C*H), 106.2 (Ar*C*H), 70.4 (CH_2OAr), 57.3 (NCH_2), 55.5 (OCH_3), 53.3 (NCH_2), 49.2 (*C*(C=O)), 26.1 (CH_3) ppm.

^{19}F NMR (376 MHz, $CDCl_3$): δ_F –107.6 ppm.

Doubling of azetidine signals due to rotamers.

HRMS (ESI$^+$): m/z calc'd for $C_{124}H_{22}NO_4F$ [M+H]$^+$: 408.1606, found: 408.1620.

IR (film): ν_{max} 2960, 1713 (C=O), 1634 (C=O), 1508, 1422, 1392, 1231, 1156, 1114, 1027, 848 cm^{-1}.

1-(4-Fluorobenzoyl)-3-(((6-methoxynaphthalen-2-yl)oxy)methyl)azetidine-3-carboxylic acid (PF-04418948)

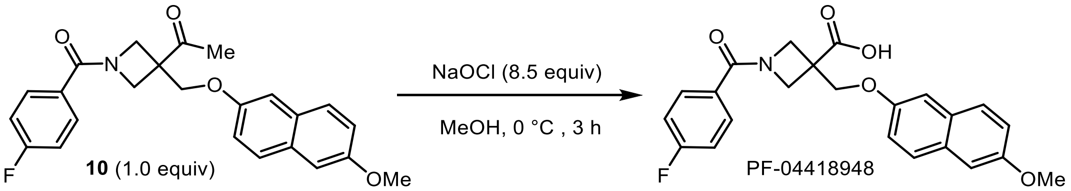

Following a modified literature procedure.[8]

To a solution of **10** (81.5 mg, 0.200 mmol, 1.00 equiv) in MeOH (2 mL) at 0 °C was added NaOCl (8% solution, 1.43 mL, 8.50 equiv) and the resulting mixture was stirred for 3 h at 0 °C. The reaction was quenched with sat. aq. Na_2SO_3 (1 mL) and then extracted with Et_2O (2× 5 mL). The aqueous layer was acidified to pH 2 with 1 M aq. HCl and extracted with EtOAc (3× 5 mL). The combined organic phases were dried ($MgSO_4$), filtered, concentrated under reduced pressure to afford pure **PF-04418948** (71.4 mg, 0.174 mmol, 87%) as a white solid.

NMR Spectroscopy (*see spectra*):

^{1}H NMR (400 MHz, $CDCl_3$): δ_H 8.06 (br. s, 1H, O*H*), 7.73 – 7.64 (m, 2H, ArC*H*), 7.63 – 7.56 (m, 2H, ArC*H*), 7.15 – 7.02 (m, 6H, ArC*H*), 4.75 (d, *J* = 9.1 Hz, 1H, NCH_2), 4.52 (d, *J* = 10.5 Hz, 1H, NCH_2), 4.48 – 4.27 (m, 4H, NCH_2, CH_2OH), 3.88 (s, 3H, OCH_3) ppm;

^{13}C NMR (126 MHz, $CDCl_3$): δ_C 174.9 ((*C*=O)OH), 169.9 ((*C*=O)Ar), 164.7 (d, *J* = 252.5 Hz, Ar*C*F), 156.6 (Ar*C*O), 154.7 (Ar*C*O), 130.5 (d, *J* = 8.9 Hz, Ar*C*H), 130.4 (Ar*C*), 129.6 (Ar*C*), 128.6 (d, *J* = 3.2 Hz, Ar*C*), 128.6 (Ar*C*H), 128.4 (Ar*C*H), 119.4 (Ar*C*H), 118.8 (Ar*C*H), 115.8 (d, *J* = 21.8 Hz, Ar*C*H), 107.7 (Ar*C*H), 106.2 (Ar*C*H), 69.4 (CH_2OAr), 58.1 (NCH_2), 55.5 (OCH_3), 54.1 (NCH_2), 43.4 (*C*(C=O)) ppm.

^{19}F NMR (376 MHz, $CDCl_3$): δ_F –107.1 ppm.

Doubling of azetidine signals due to rotamers.

HRMS (ESI$^+$): m/z calc'd for $C_{23}H_{20}NO_5F$ [M+H]$^+$: 410.1398, found: 410.1384.

IR (film): ν_{max} 3100-2600 (O-H), 2937, 1729 (C=O), 1603 (C=O), 1509, 1452, 1392, 1230, 1158, 1115, 1029, 848 cm^{-1}.

2.8. Unsuccessful Substrates

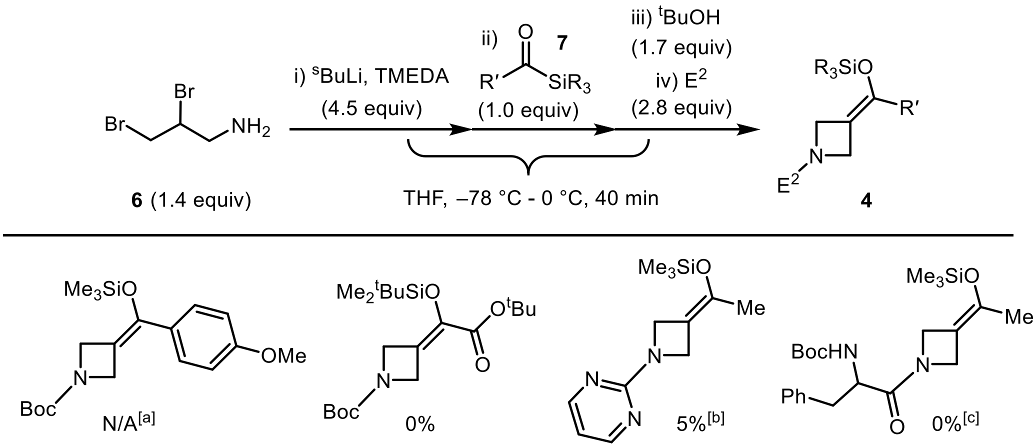

Scheme S1: Unsuccessful substrates in the synthesis of silyl enol ether 4

Reactions were performed using **7** (0.322 mmol) according to a modified General Procedure C in which no silyl enol ether hydrolysis was performed. Yield was determined by ^{1}H NMR analysis using dibromomethane as an internal standard. [a] Acyl silane (4-Methoxyphenyl(trimethylsilyl)methanone) was discovered to have decomposed. [b] Aryl chloride not soluble in reaction mixture. [c] From corresponding amino acid pre-activated with EDCI·HCl or CDI.

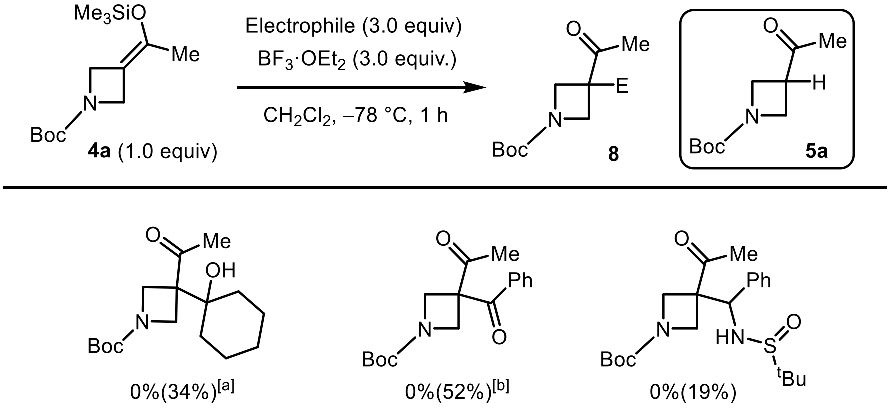

Scheme S2: Unsuccessful aldol/Mannich reactions of 4a

All reactions were carried out using **4a** (0.20 mmol) according to the conditions used for the synthesis of 8i. Yields determined by ^{1}H NMR analysis using dibromomethane as an internal standard, with yields of **5a** given in parenthesis. [a] 50% returned starting material observed. [b] Using benzoyl chloride. 21% returned starting material observed.

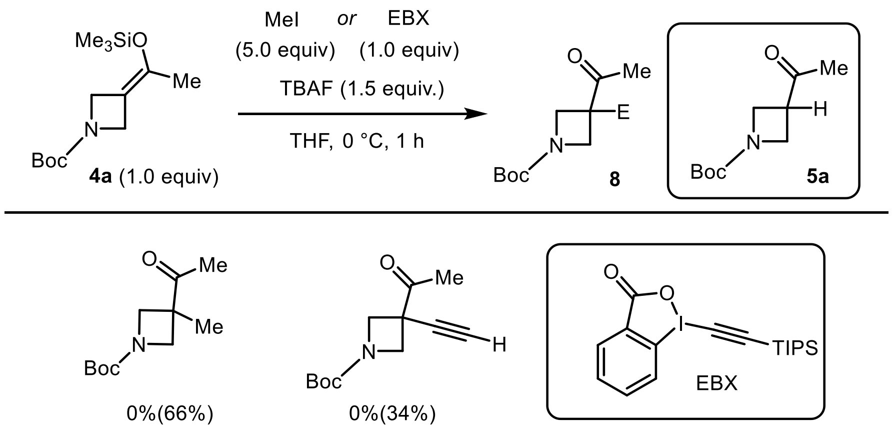

Scheme S3: Unsuccessful electrophilic functionalisation reactions of 4a

All reactions were carried out using **4a** (0.20 mmol) according to the conditions shown. Yields determined by ^{1}H NMR analysis using dibromomethane as an internal standard, with yields of **5a** given in parenthesis. In all cases, full starting material consumption was observed.

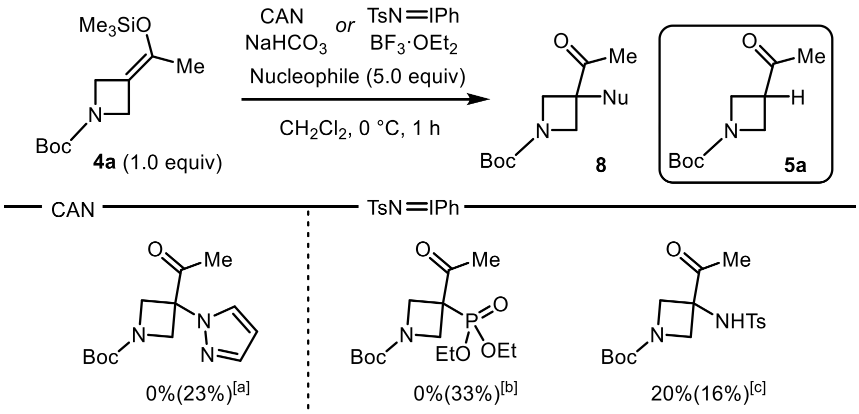

Scheme S4: Unsuccessful oxidative nucleophilic coupling reactions of 4a

All reactions were carried out using **4a** (0.20 mmol) according to a modified General Procedure D. Yields determined by ^{1}H NMR analysis using dibromomethane as an internal standard, with yields of **5a** given in parenthesis. In all cases, full starting material consumption was observed. [a] MeCN used as reaction solvent. [b] Using triethylphosphite. [c] Product could not be isolated pure by column chromatography.

2.9. In Situ Infra-Red Spectroscopy Experiments

2.9.1. React-IR setup procedure

To set up a reaction with the React-IR probe, a 3 necked 25 mL flask was used. The recently cleaned probe was then inserted into the top of the flask using a Teflon adaptor (supplied with the instrument) inside a glass adaptor, secured with a clip, and placed at a height where it would be immersed in the reaction mixture, but did not touch the stirring bar. The other necks of the flask were fitted with a Schlenk line adapter and a suba seal to allow the addition of reagents. The contrast and align, performance (5 runs) and stability (duration of 5 min) tests were then performed and saved. After the tests, the flask was flame dried and placed under vacuum. The background was taken of dibromo-amine **6** and TMEDA in the reaction solvent at −78 °C. Once set up, the experiment was started with a fixed scan interval of 15 seconds and all reagents were sequentially added to the reaction. The resistance temperature detector (RTD) on the probe was not used. Note: It is important to turn the React-IR instrument on 1 – 3 h before an experiment is started. Once ready the 'TEMP OK' display under the 'contrast and align' test will change from 'false' to 'okay'. This could take anywhere from 1 – 3 h.

2.9.2. Steady state infra-red spectra

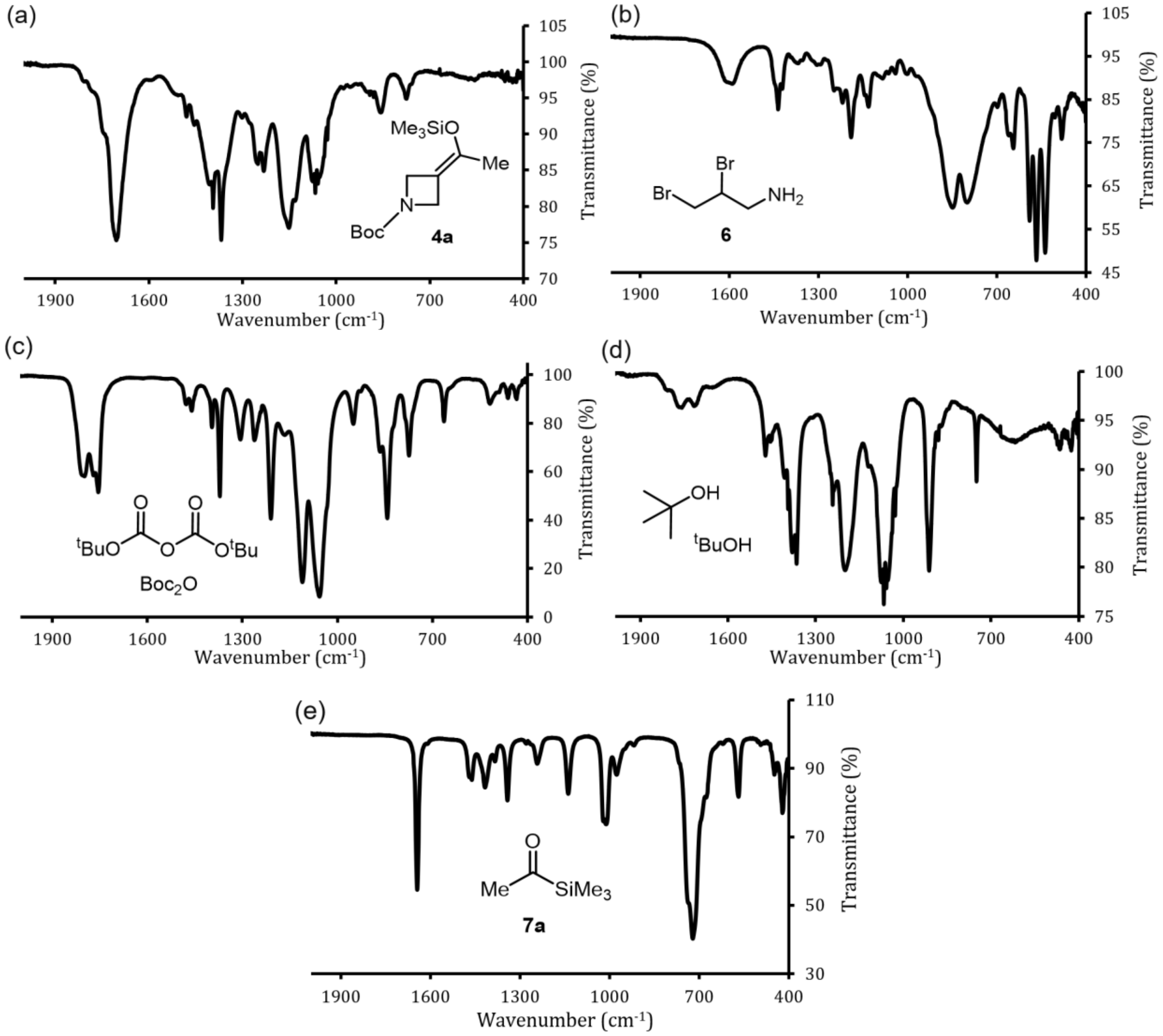

Figure S1: Steady state infra-red spectra: (a) 4a; (b) 6; (c) Boc_2O; (d) ^tBuOH; (e) 7a

2.9.3. Formation of ABB-Li

The addition of ^sBuLi was performed over 9 min during which time the formation of ABB-Li (**1**) was complete. Starting material consumption (839 cm^{-1}) occurs gradually and does not occur fully until the generation of **1** (772 cm^{-1}) is complete (Figure S2). This suggests that ABB-Li (**1**) forms when starting material is still present. The delay in the generation of this signal (772 cm^{-1}) would be expected for ABB-Li as the bicycle must first be assembled and then deprotonated. The intermediate on this pathway, ABB (**2**), can also be observed at 802 cm^{-1}, where the intensity of this signal is shown to increase and then immediately decrease as the addition of ^sBuLi progresses. The initial decrease in signal intensity for both these peaks is due to overlapping signals at 783 cm^{-1} and 810 cm^{-1} which decrease in absorption intensity upon addition of ^sBuLi. After the addition of base is complete, the IR spectrum reaches a steady state, indicating that no further bond breaking/forming processes are occurring.

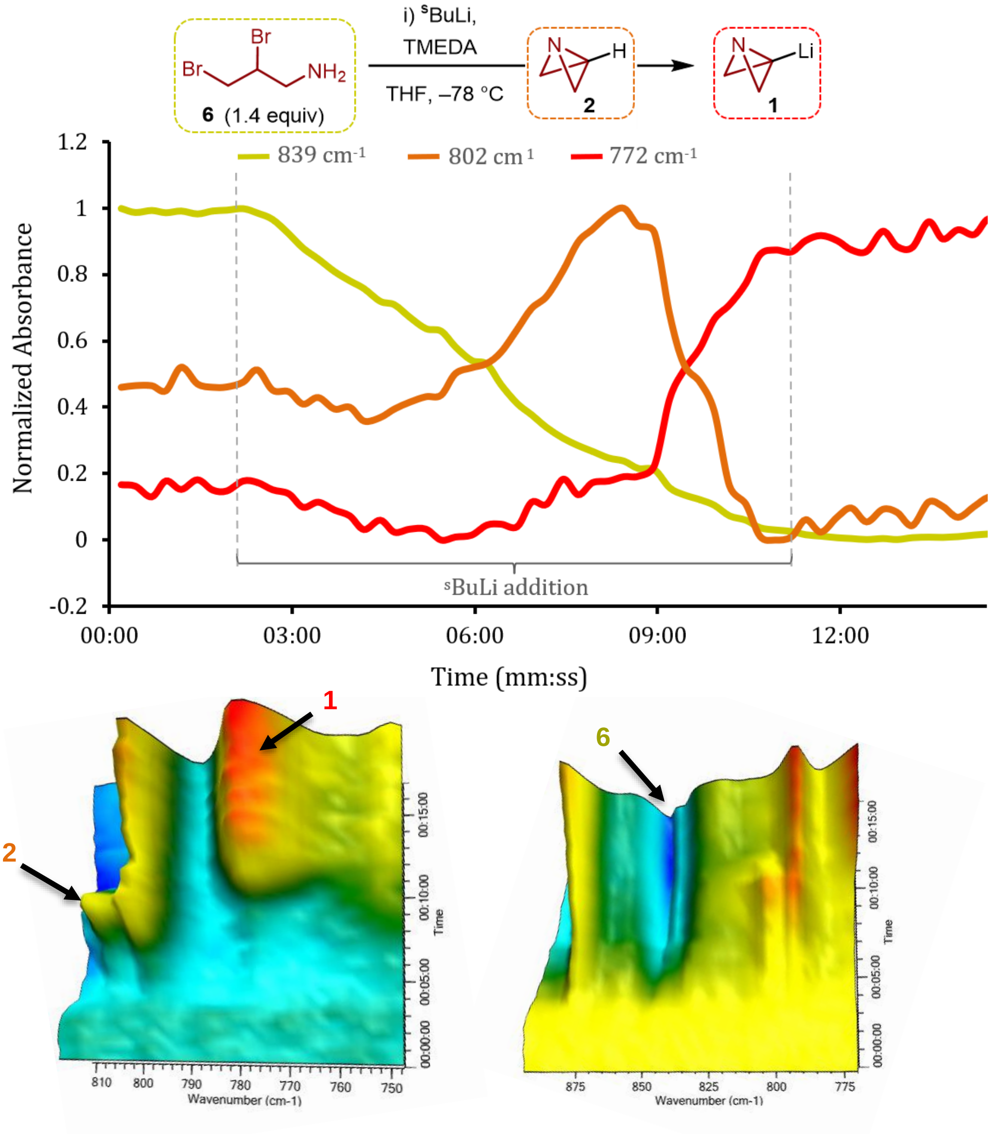

All absorption intensities are normalised to values between 0 and 1.

Figure S2: 2D and 3D plots of absorbance versus time for species involved in the synthesis of ABB-Li (1)

2.9.4. [1,2]-Brook rearrangement/strain-release-driven anion relay reaction

The absorption intensity at 772 cm^{-1} decreased partially upon addition of **7a**, in-line with what would be expected for intermediate **1** (Figure S3). As **1** is in excess it is not fully consumed until the addition of ^{t}BuOH, where protonation of this organolithium regenerates ABB (**2**), as seen by an increase in the absorption at 802 cm^{-1}. Despite the clear observations of reactivity occurring upon adding acyl silane **7a**, this starting material reacted so rapidly that the carbonyl stretch (1645 cm^{-1}) was not observed on the timescale of the experiment. Presumably the 1,2-addition of **1** to **7a** occurs instantaneously during dropwise addition preventing any build-up of **7a** in solution. Moreover, a steady state was reached once the addition of **7a** was complete, indicating that the entire [1,2]-Brook rearrangement/strain-release-driven anion relay sequence occurs essentially instantaneously at −78 °C. Upon addition of **7a** a new signal at 1048 cm^{-1} was observed that showed a further increase in absorption intensity during the addition of ^{t}BuOH. However, it is unclear whether this second increase occurs due to the formation of **3a-H** or due to an overlapping signal associated with ^{t}BuOH or ^{t}BuOLi.

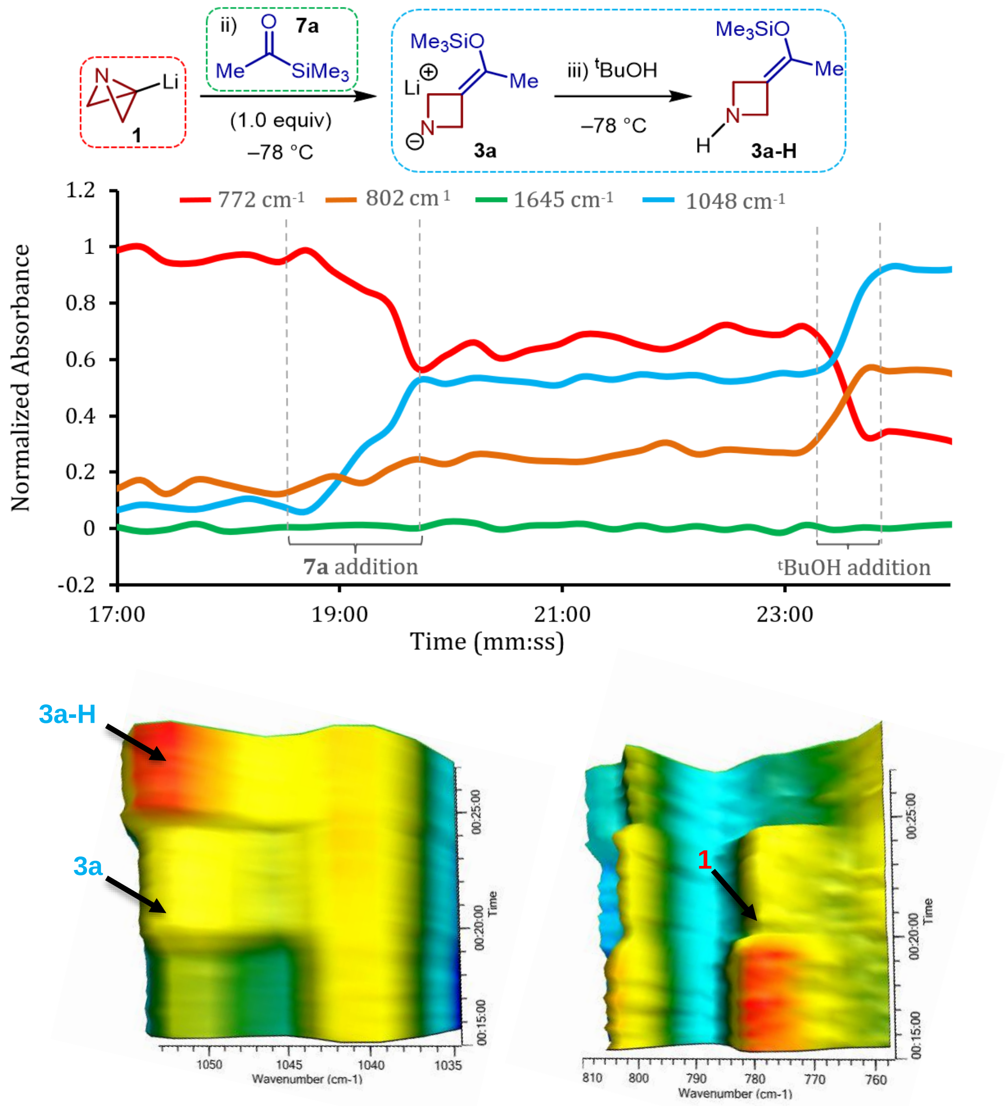

All absorption intensities are normalised to values between 0 and 1.

Figure S3: 2D and 3D plots of absorbance versus time for species involved in the [1,2]-Brook rearrangement/strain-release-driven anion relay reaction

2.9.5. Reaction 3a-H with Boc_2O

When the reaction is warmed to 0 °C the intensities of all wavenumbers are affected, as shown in Figure S4 by the decrease in absorption intensities (1048 cm^{-1} and 1704 cm^{-1} are shown). Therefore, no meaningful data can be collected during this time. However, upon di-*tert*-butyl dicarbonate (Boc_2O) addition, the formation of the product can be clearly observed by its carbonyl stretch at 1704 cm^{-1}, which reaches a steady state after approximately 3 min. This occurs simultaneously with a decrease in the absorption intensity at 1048 cm^{-1}.

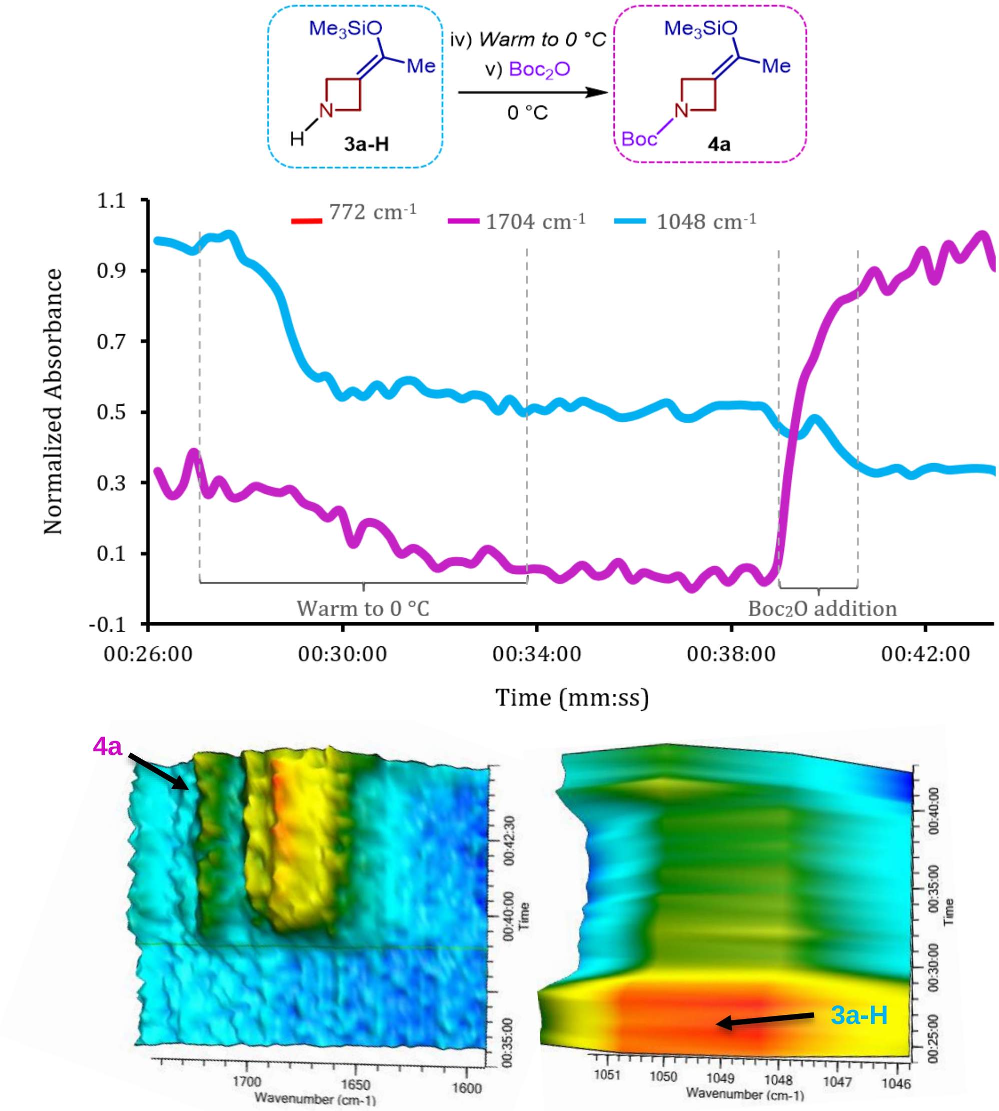

All absorption intensities are normalised to values between 0 and 1.

Figure S4: 2D and 3D plots of absorbance versus time for the reaction of 3a-H with Boc_2O

5. SPECTROSCOPIC DATA

^{1}H NMR (400 MHz, $CDCl_3$) of **7a′** (*see procedure*)

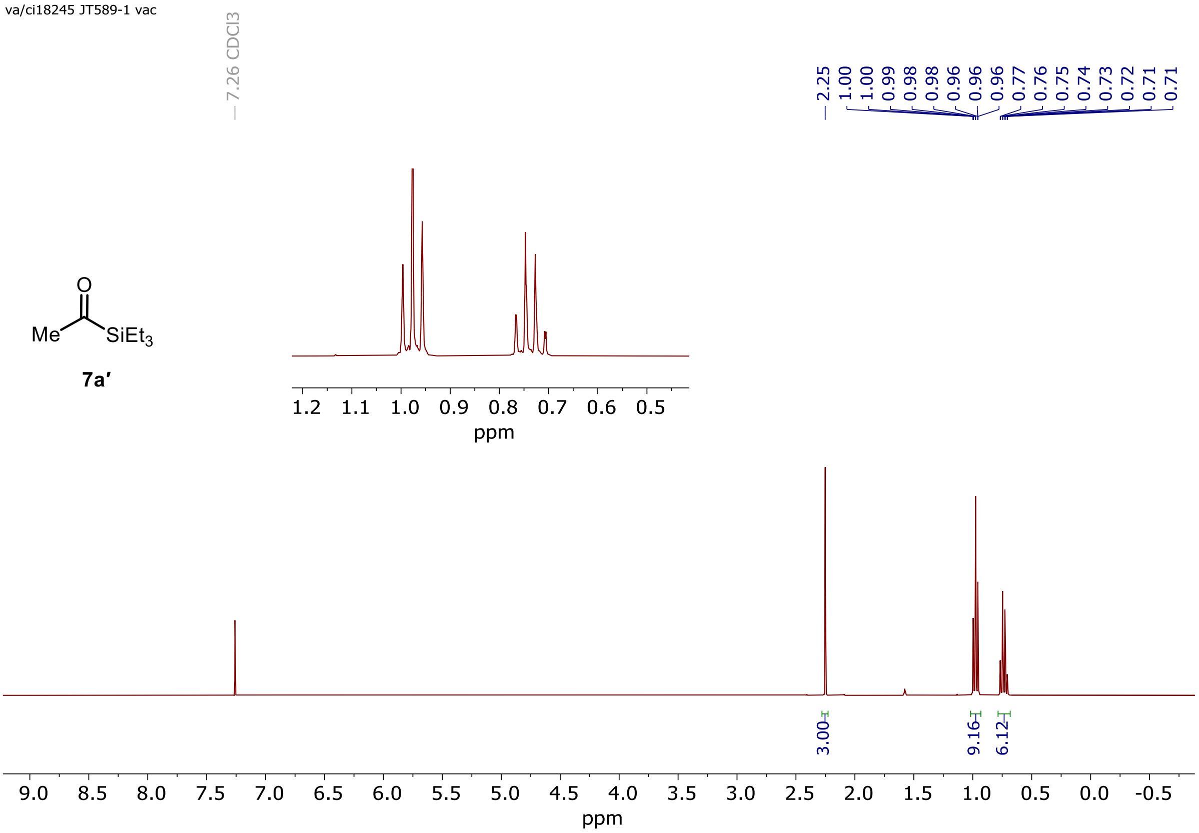

^{13}C NMR (126 MHz, $CDCl_3$) of **7a′**

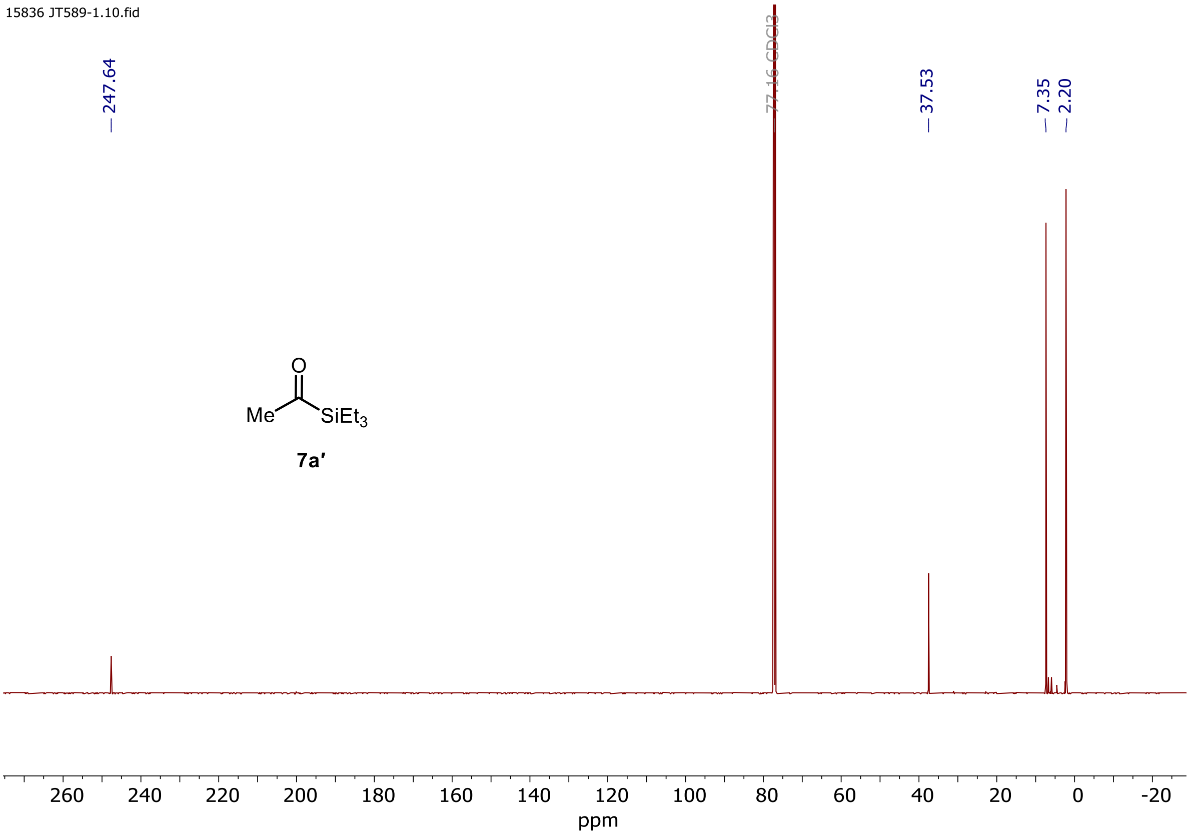

^{1}H NMR (400 MHz, $CDCl_3$) of **7a″** (*see procedure*)

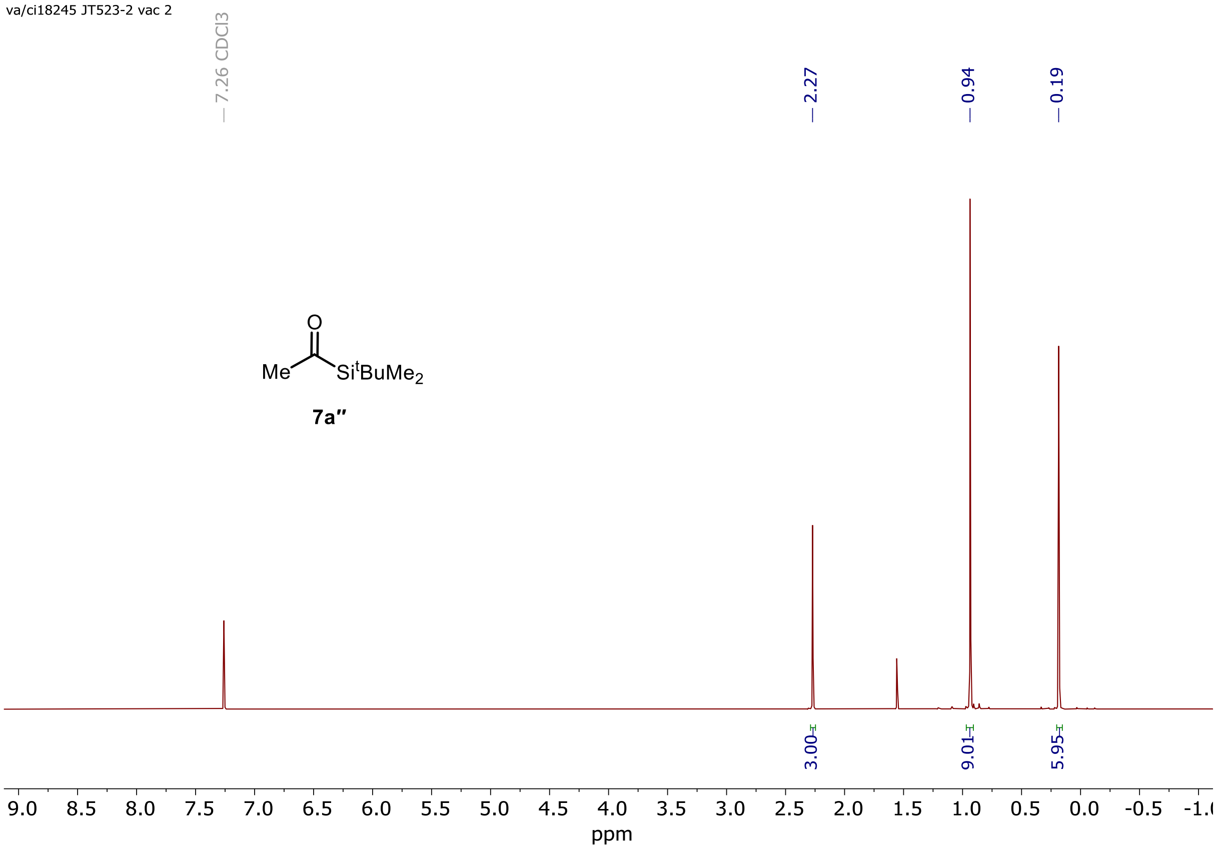

^{13}C NMR (126 MHz, $CDCl_3$) of **7a″**

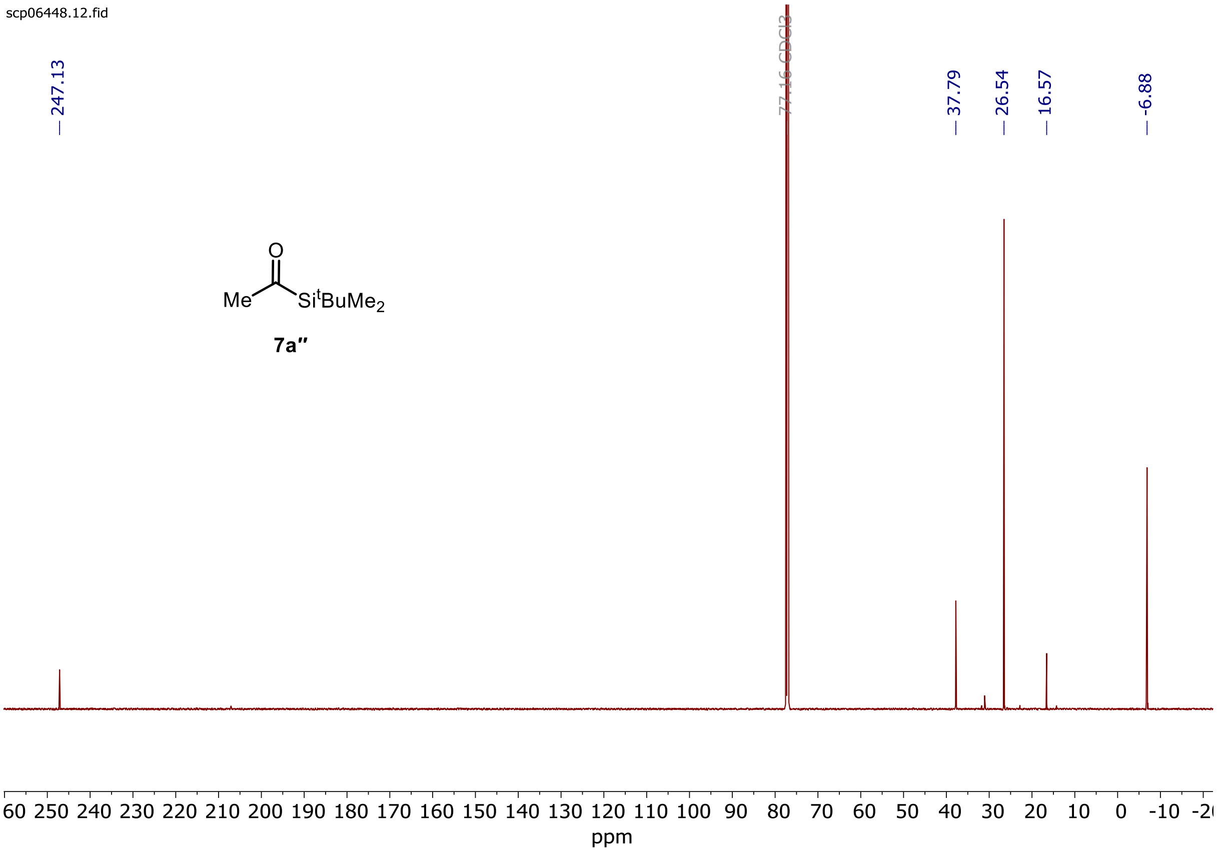

^{1}H NMR (500 MHz, $CDCl_3$) of **7b** (*see procedure*)

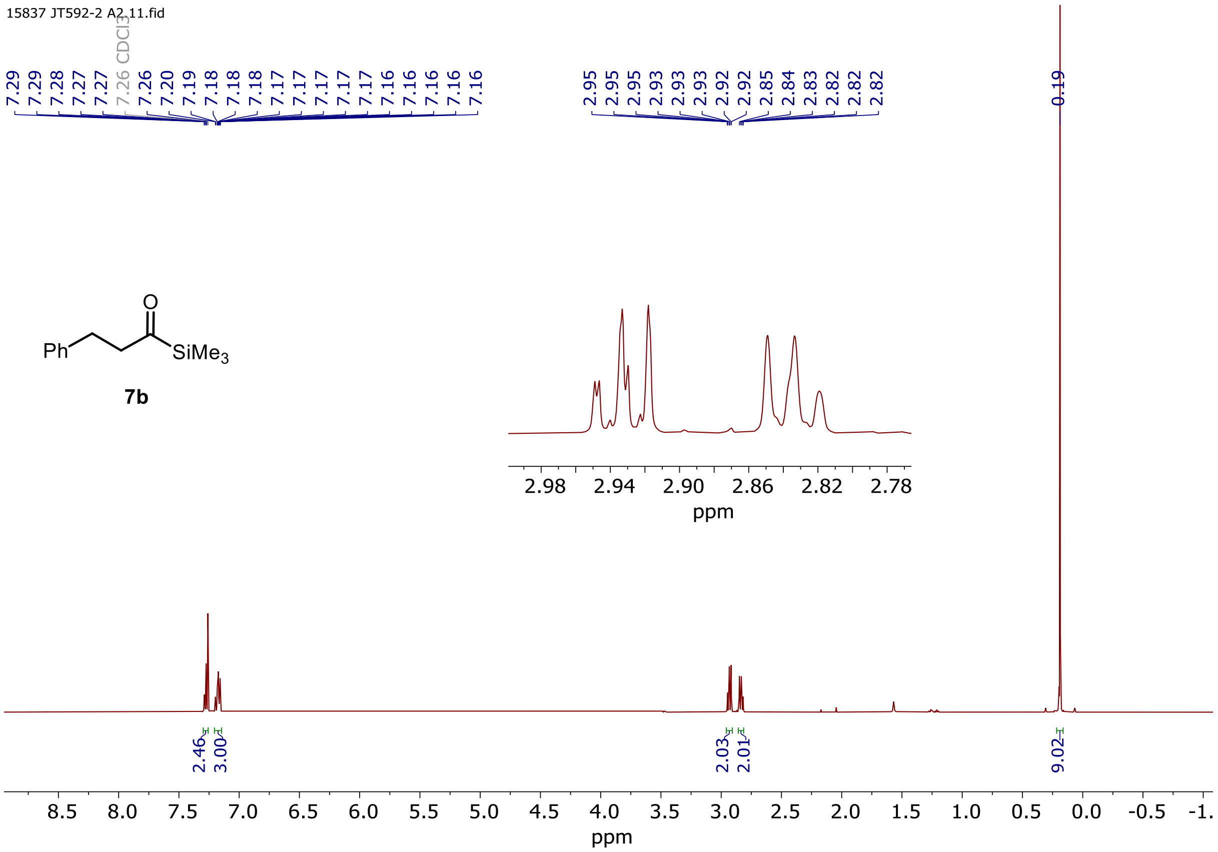

^{13}C NMR (126 MHz, $CDCl_3$) of **7b**

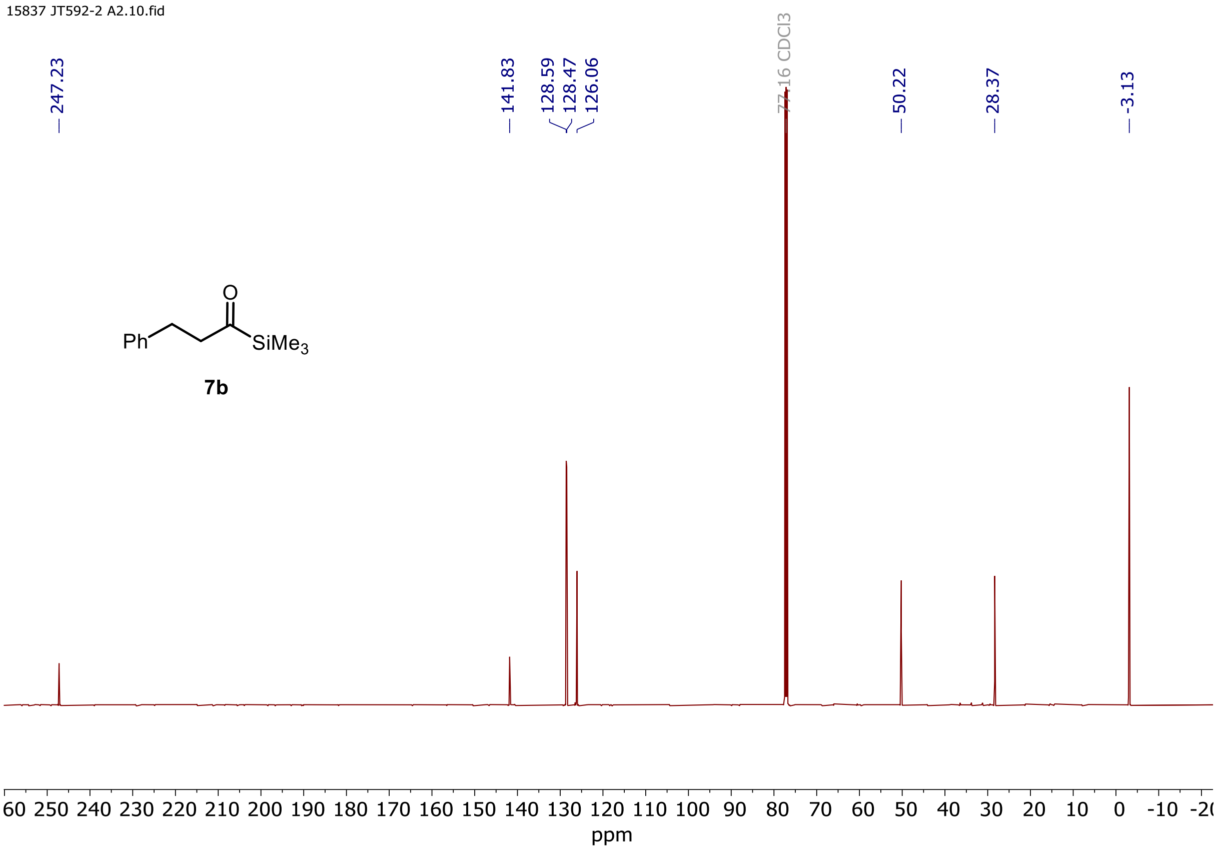

^{1}H NMR (500 MHz, $CDCl_3$) of **7c** (*see procedure*)

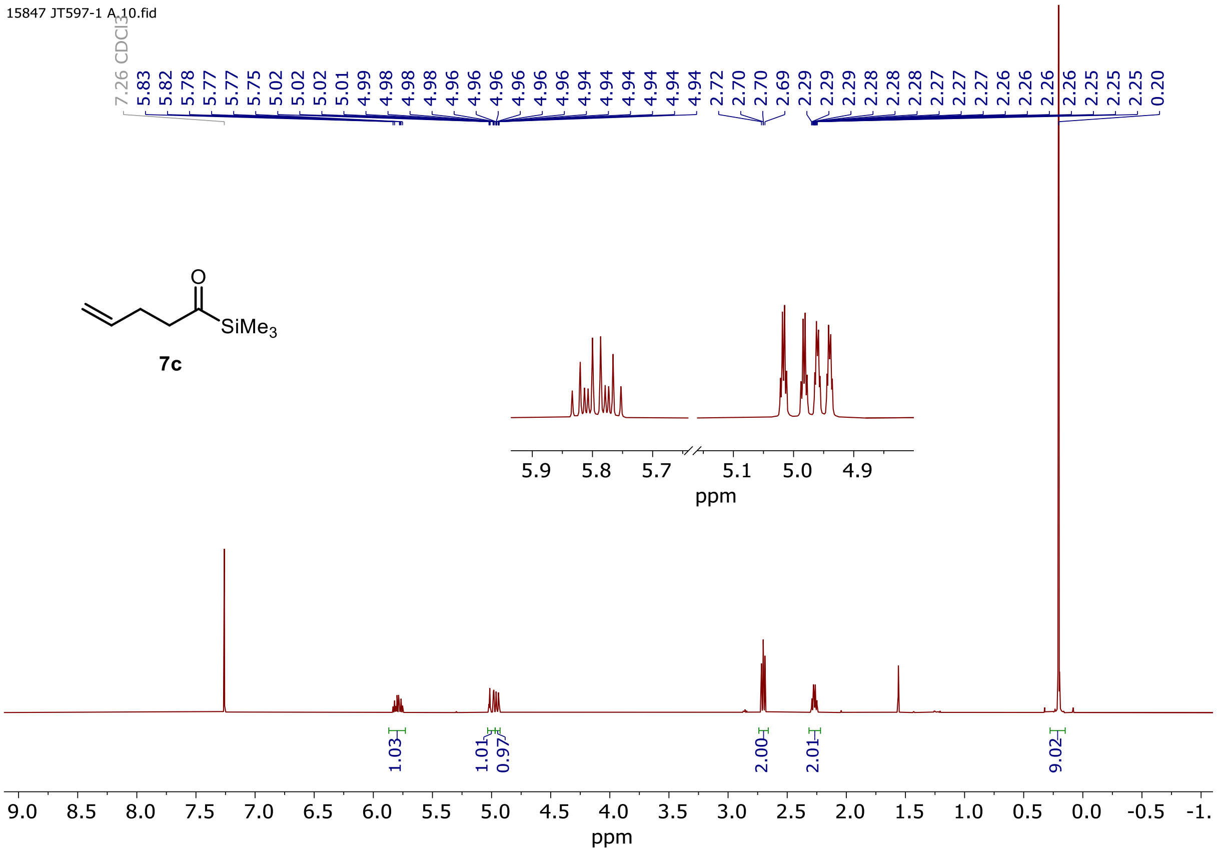

^{13}C NMR (126 MHz, $CDCl_3$) of **7c**

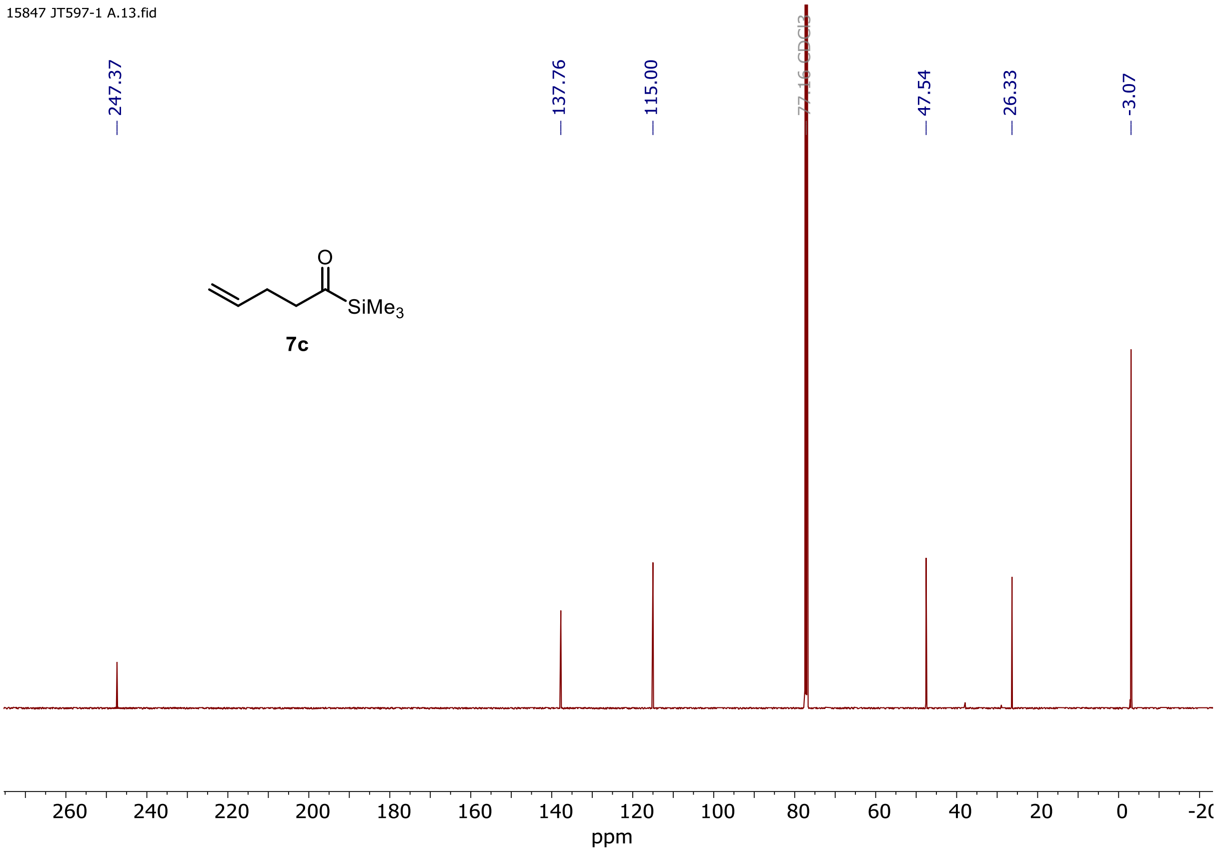

^{1}H NMR (500 MHz, $CDCl_3$) of **7d** (*see procedure*)

15875 JT599-1 A.11.fid

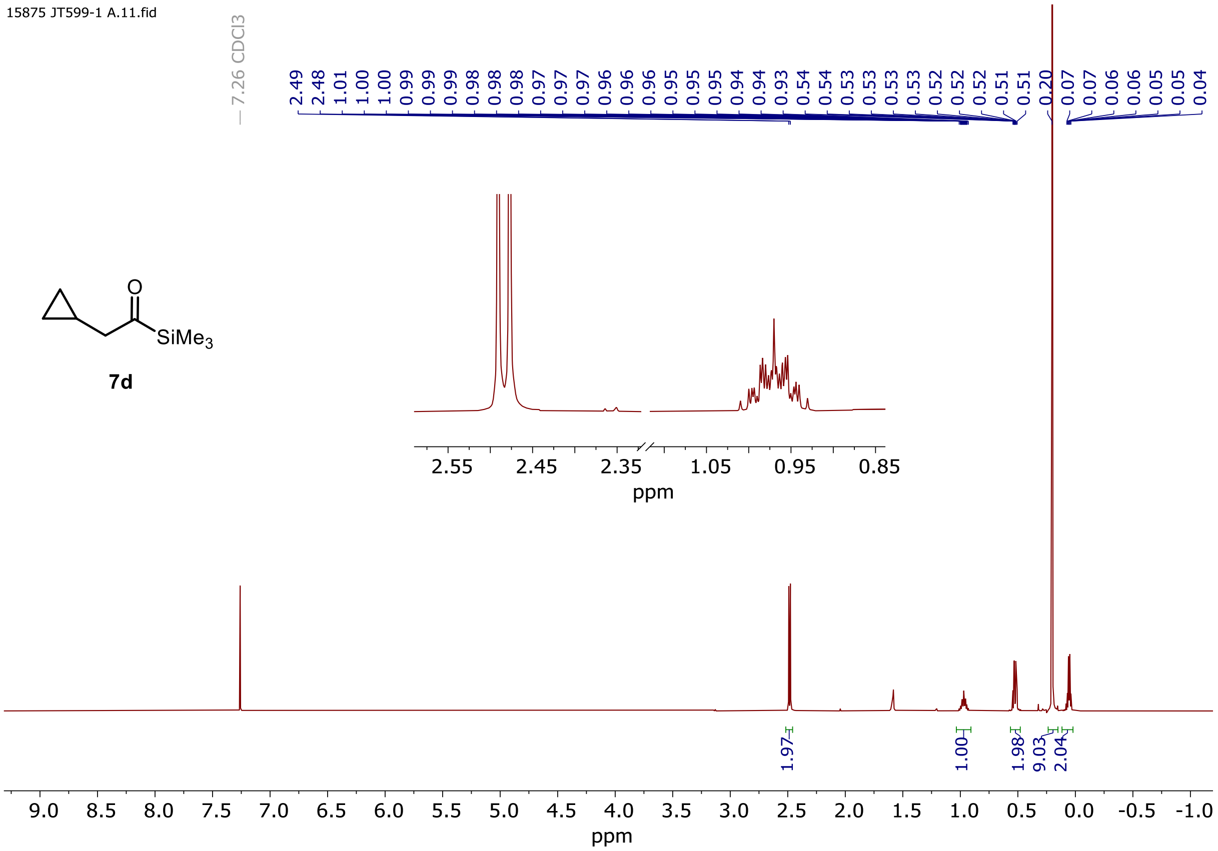

^{13}C NMR (126 MHz, $CDCl_3$) of **7d**

scp50685.14.fid

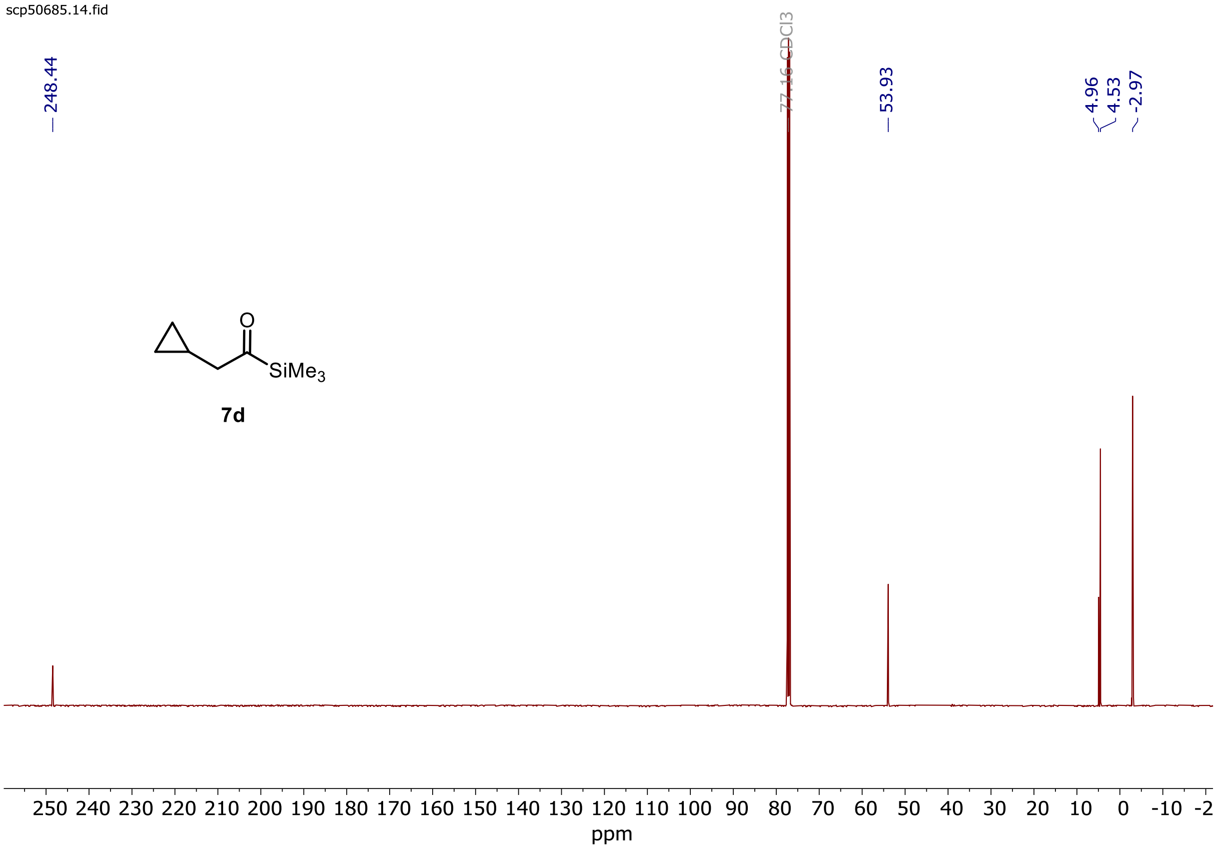

^{1}H NMR (500 MHz, $CDCl_3$) of **7e** (*see procedure*)

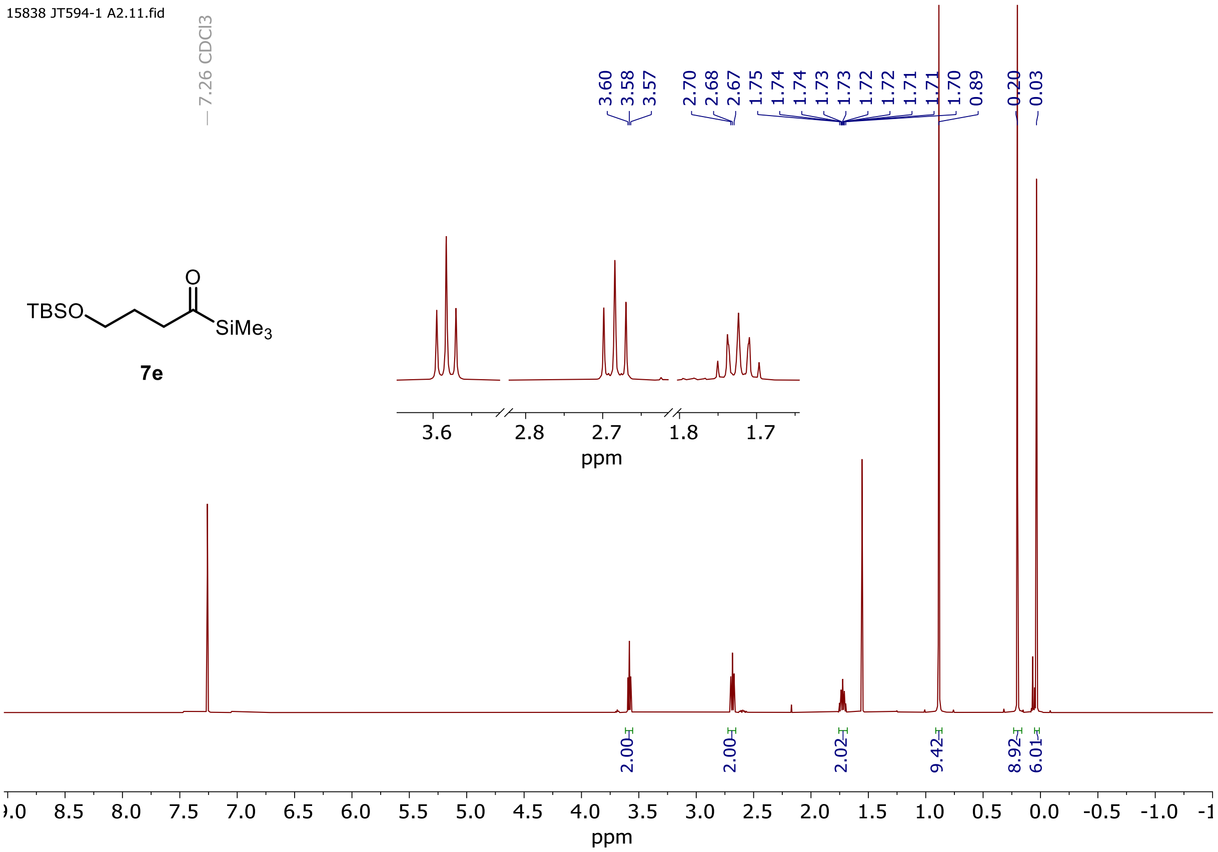

^{13}C NMR (126 MHz, $CDCl_3$) of **7e**

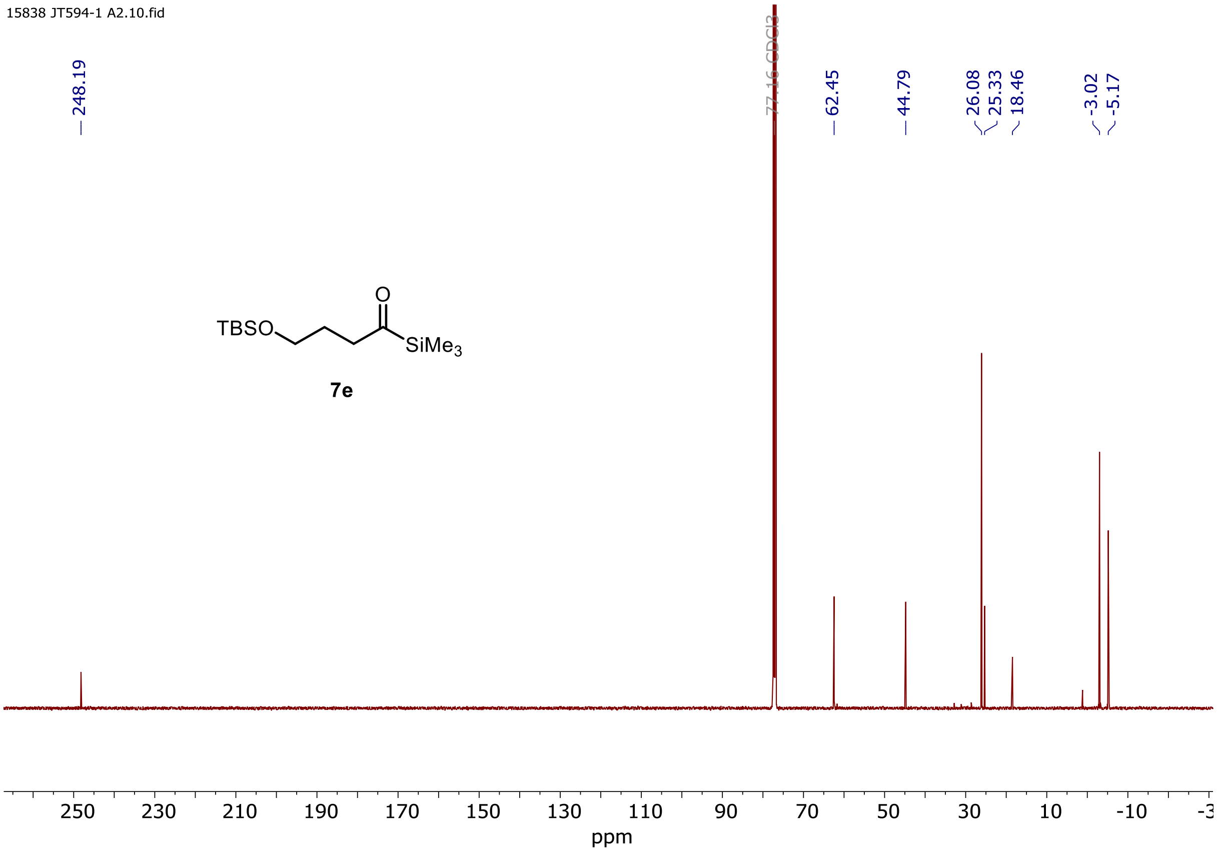

^{1}H NMR (500 MHz, $CDCl_3$) of **7f** (see procedure)

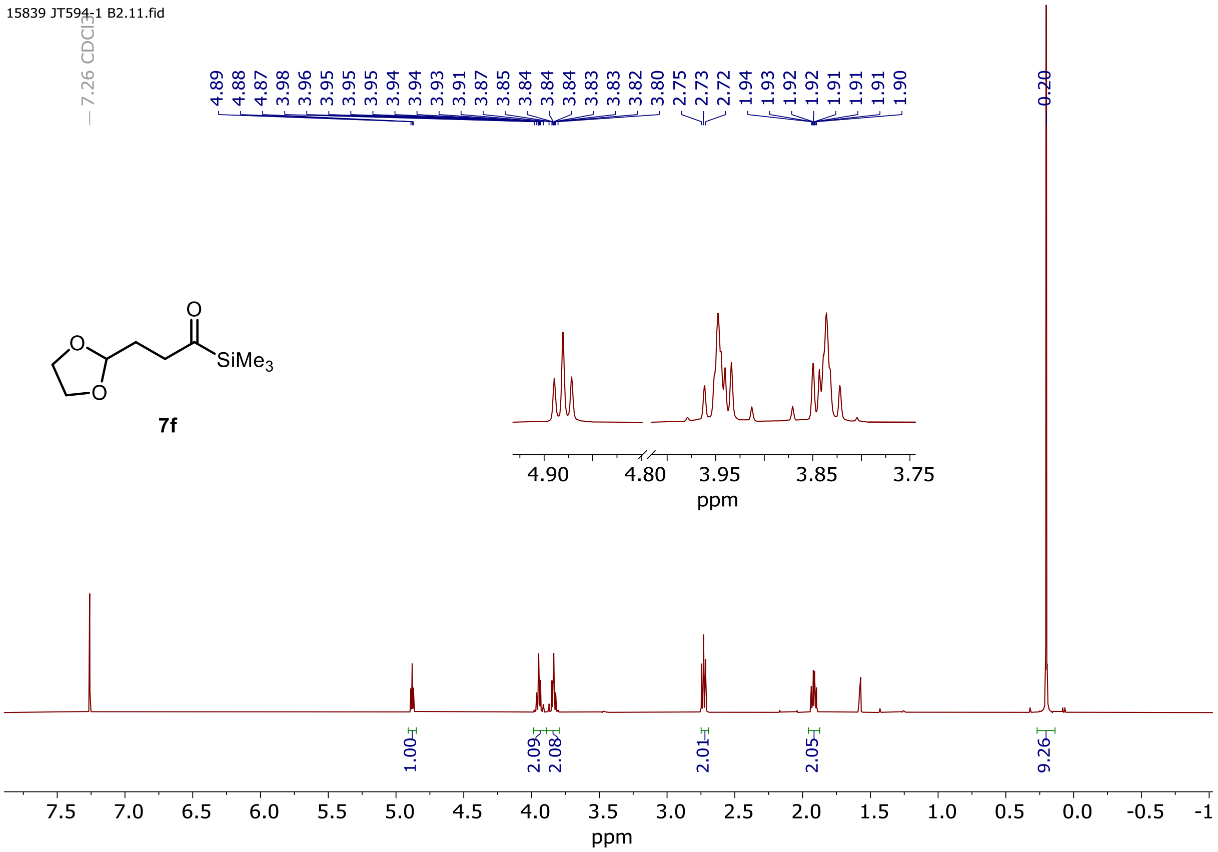

^{13}C NMR (126 MHz, $CDCl_3$) of **7f**

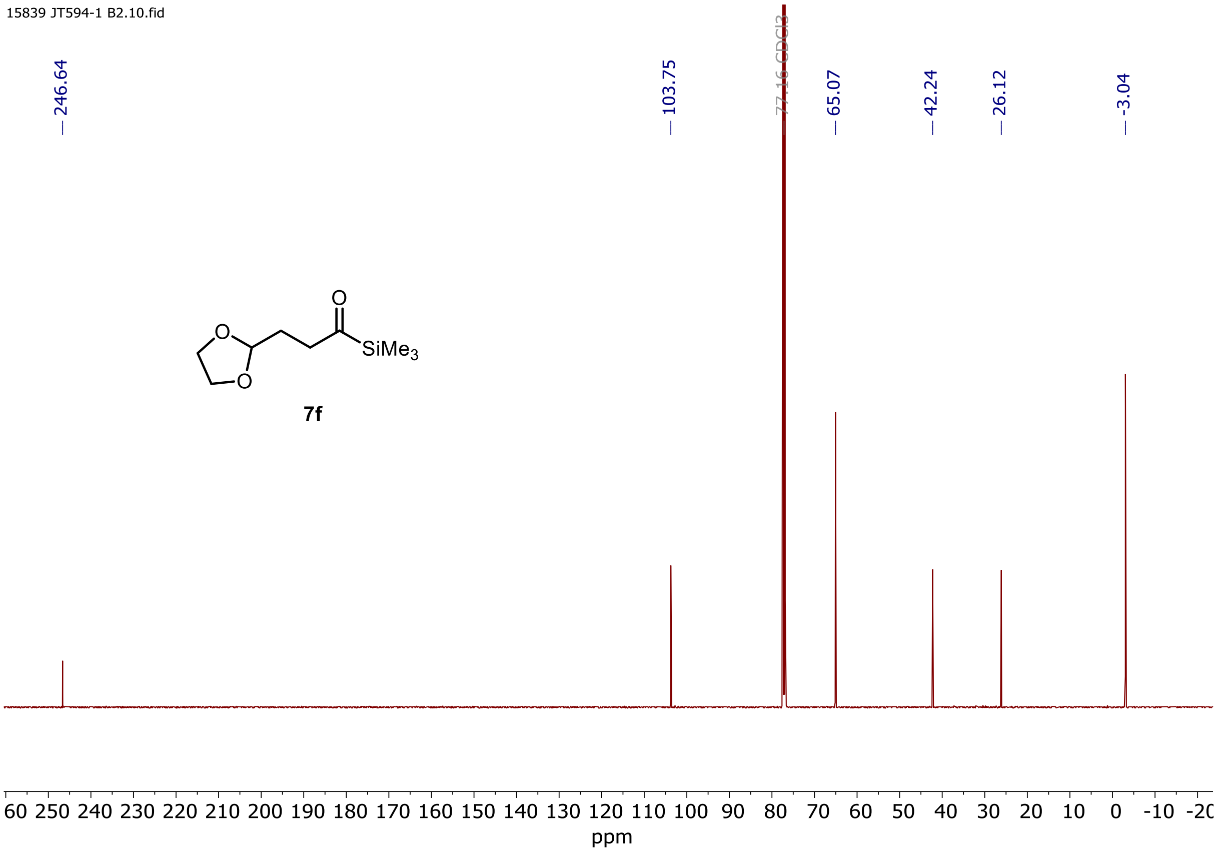

^{1}H NMR (400 MHz, $CDCl_3$) of **7g** (*see procedure*)

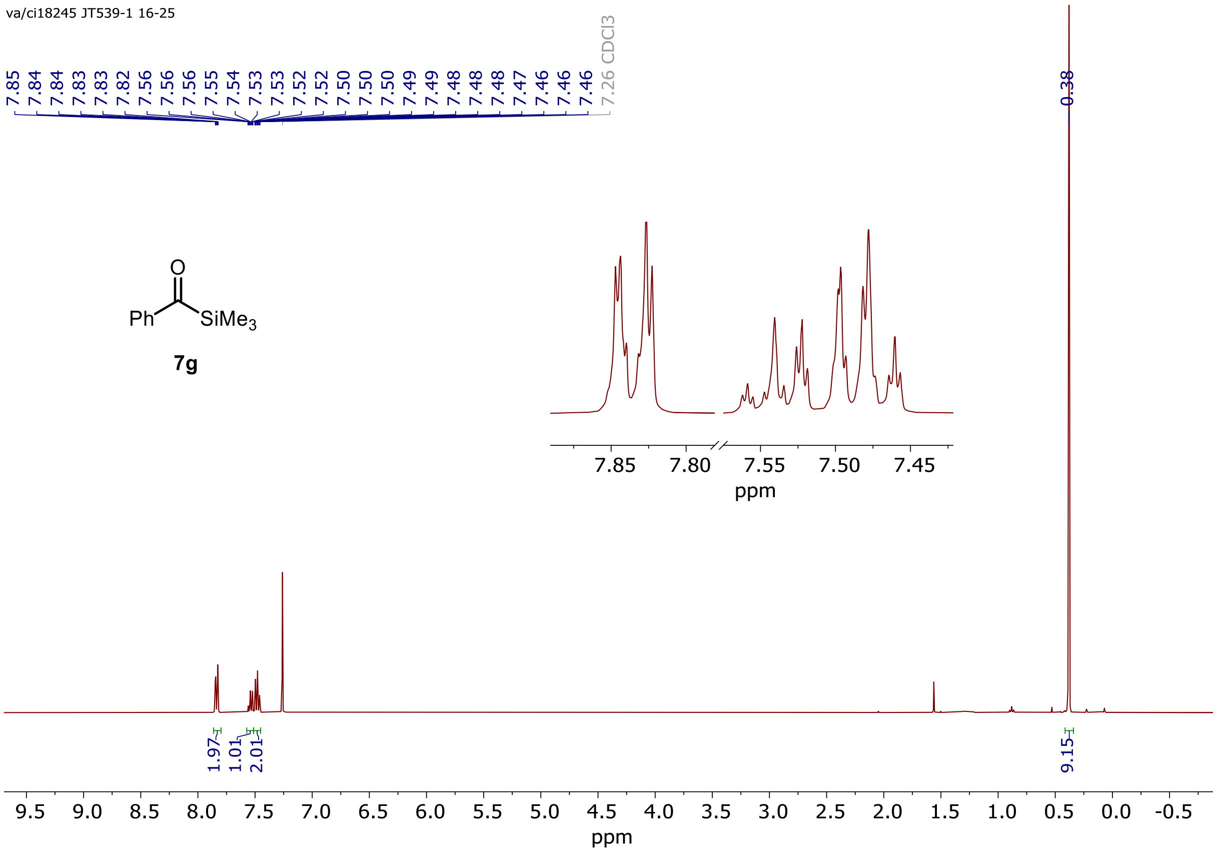

^{13}C NMR (126 MHz, $CDCl_3$) of **7g**

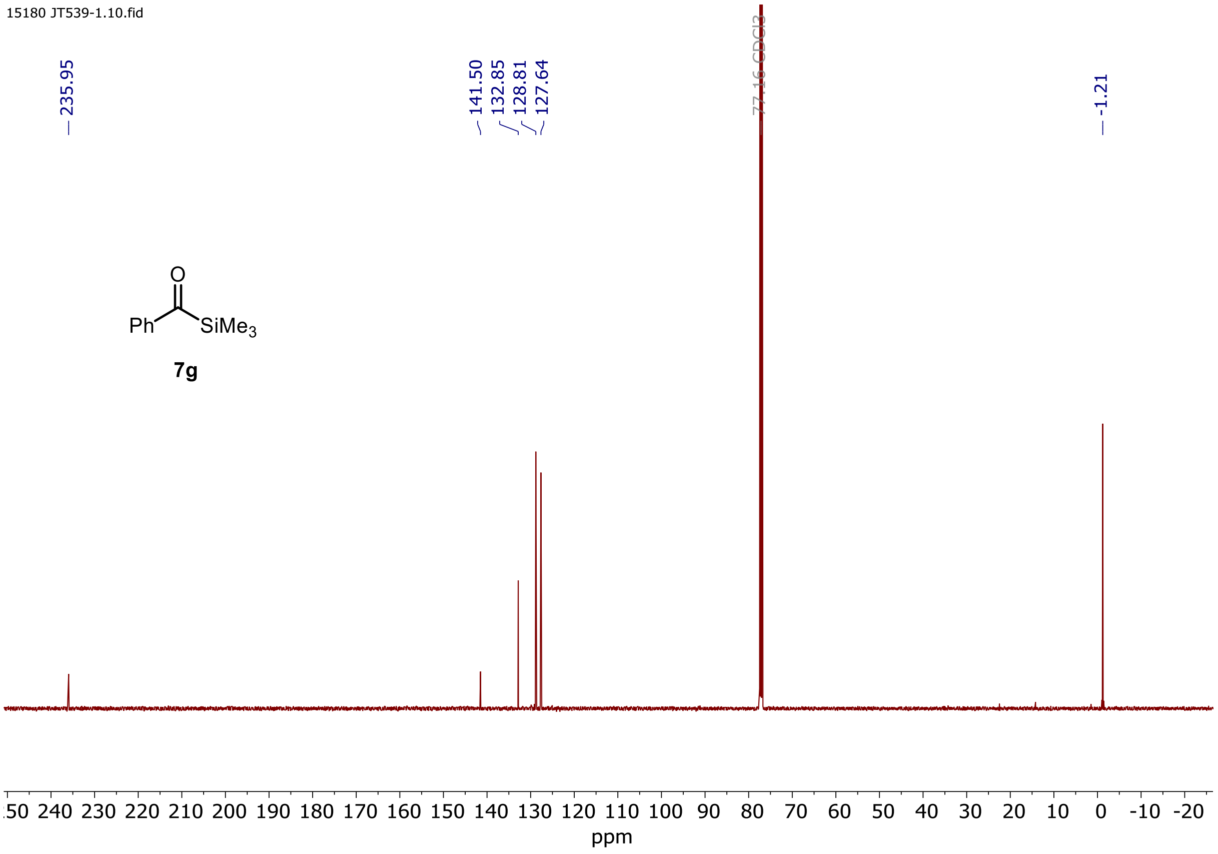

^{1}H NMR (400 MHz, $CDCl_3$) of **7g′** (*see procedure*)

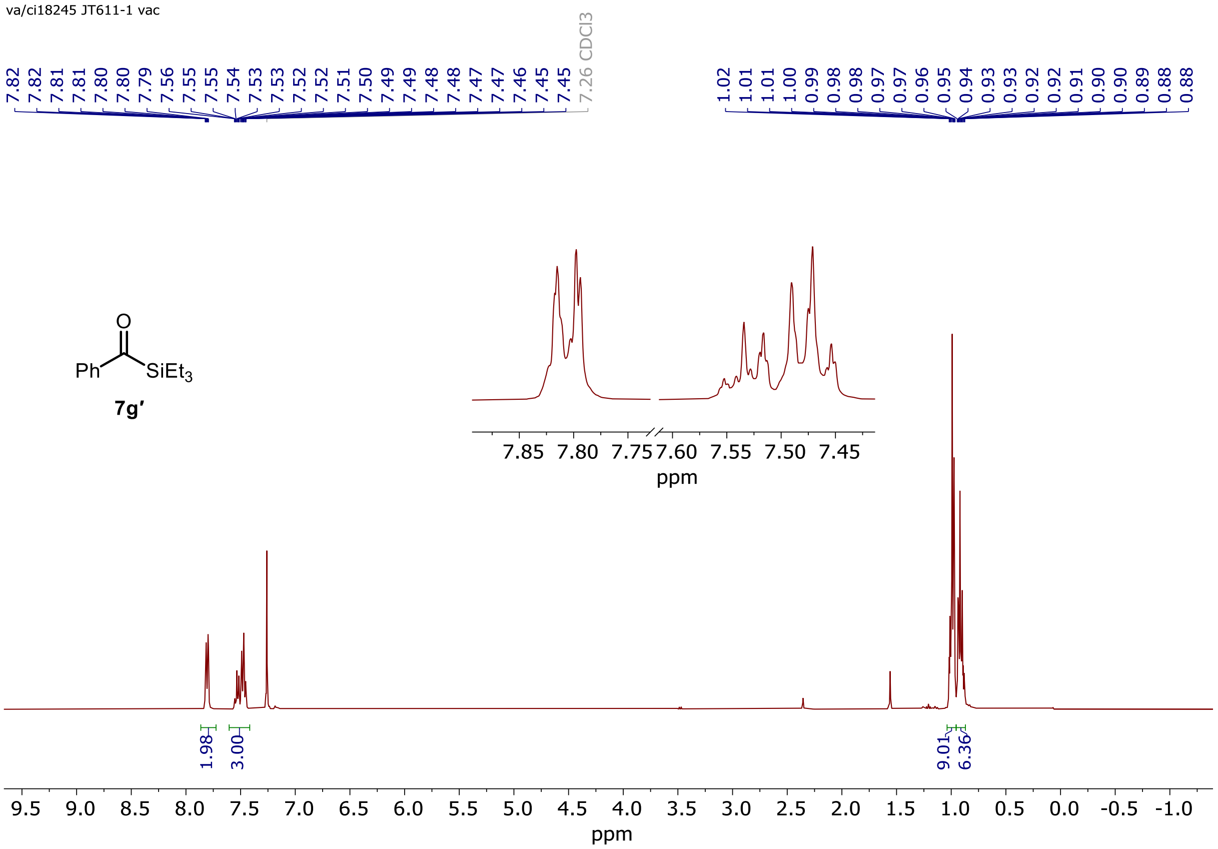

^{13}C NMR (126 MHz, $CDCl_3$) of **7g′**

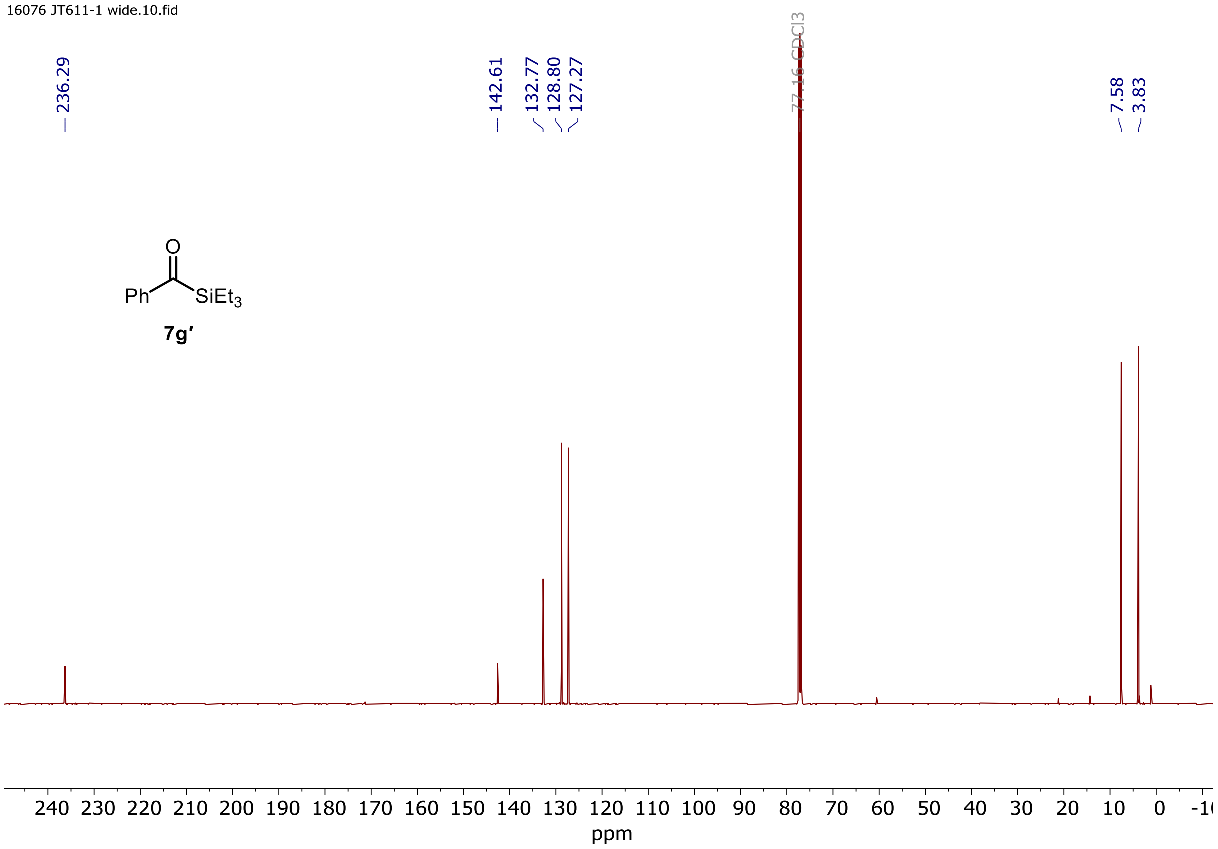

^{1}H NMR (400 MHz, $CDCl_3$) of **4a** (*see procedure*)

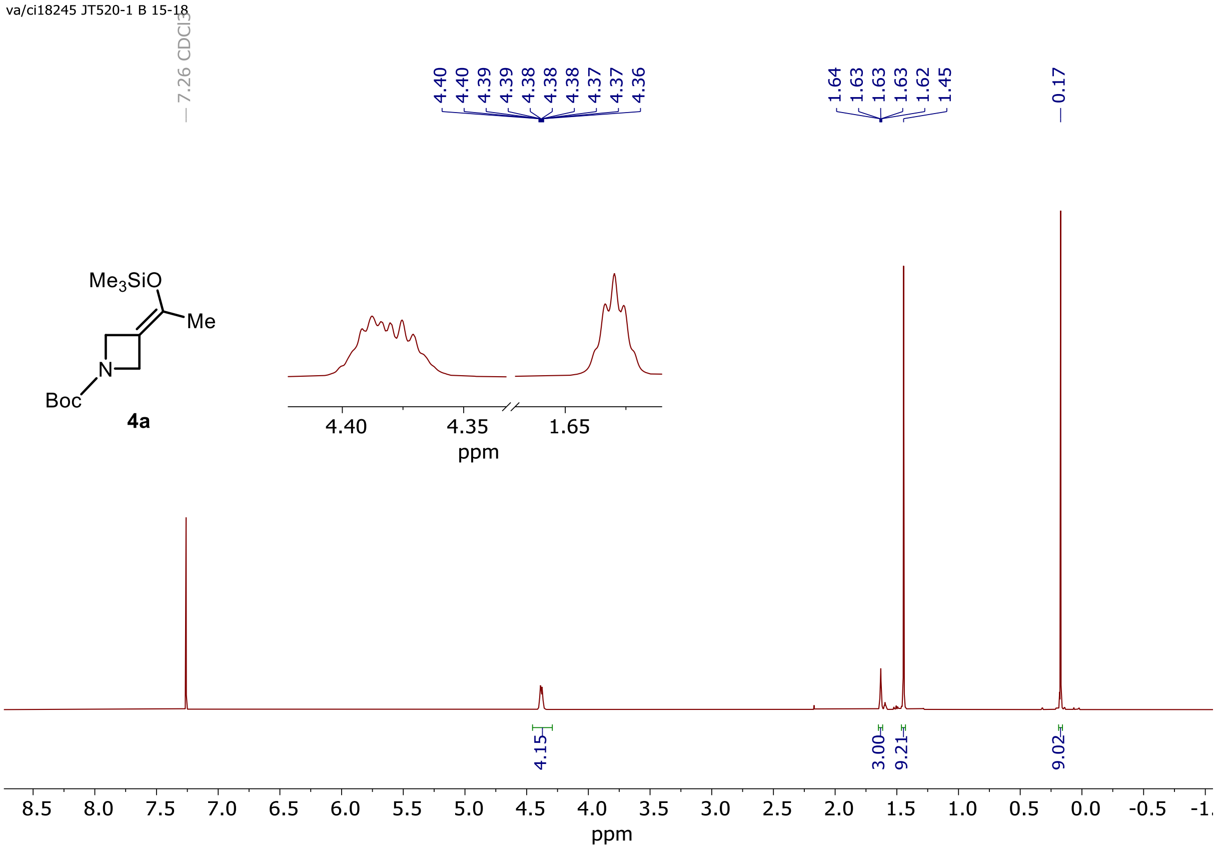

^{13}C NMR (126 MHz, $CDCl_3$) of **4a**

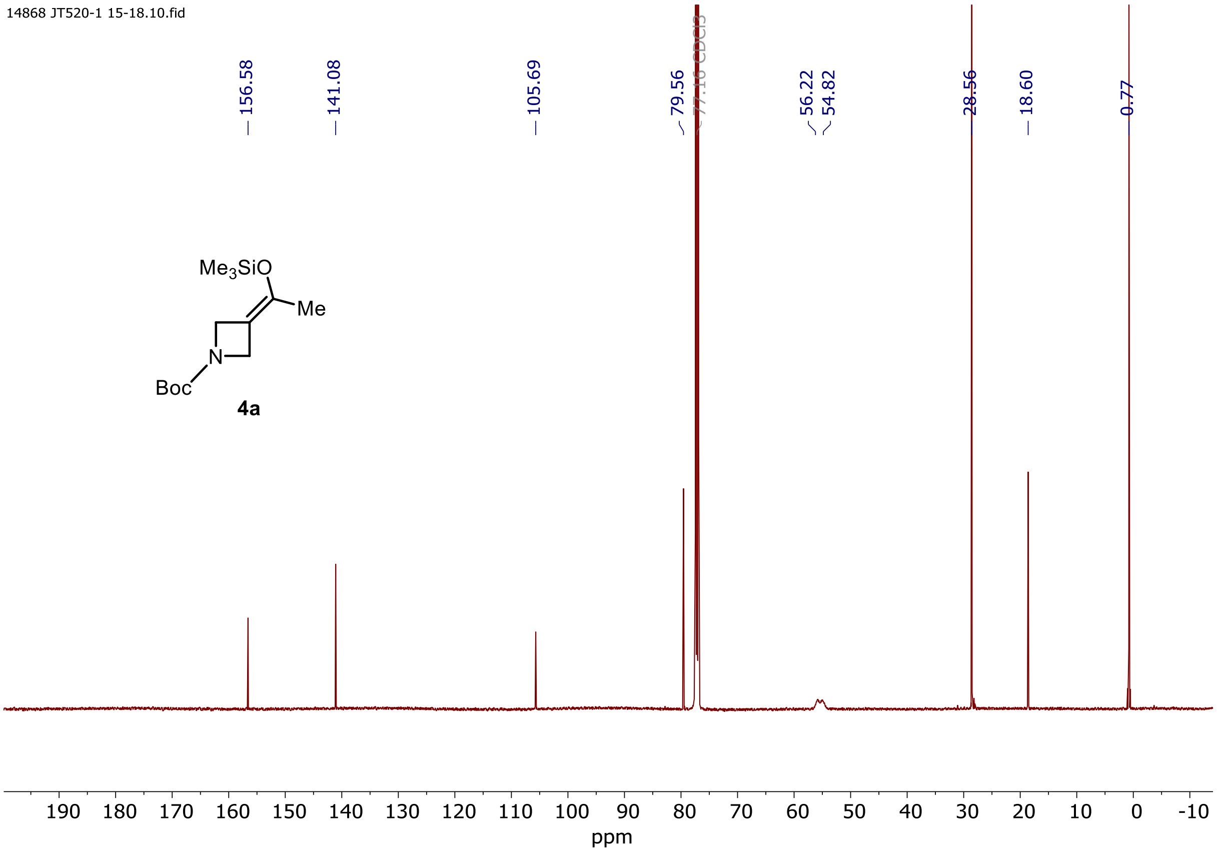

^{1}H NMR (400 MHz, $CDCl_3$) of **4a′** (see procedure)

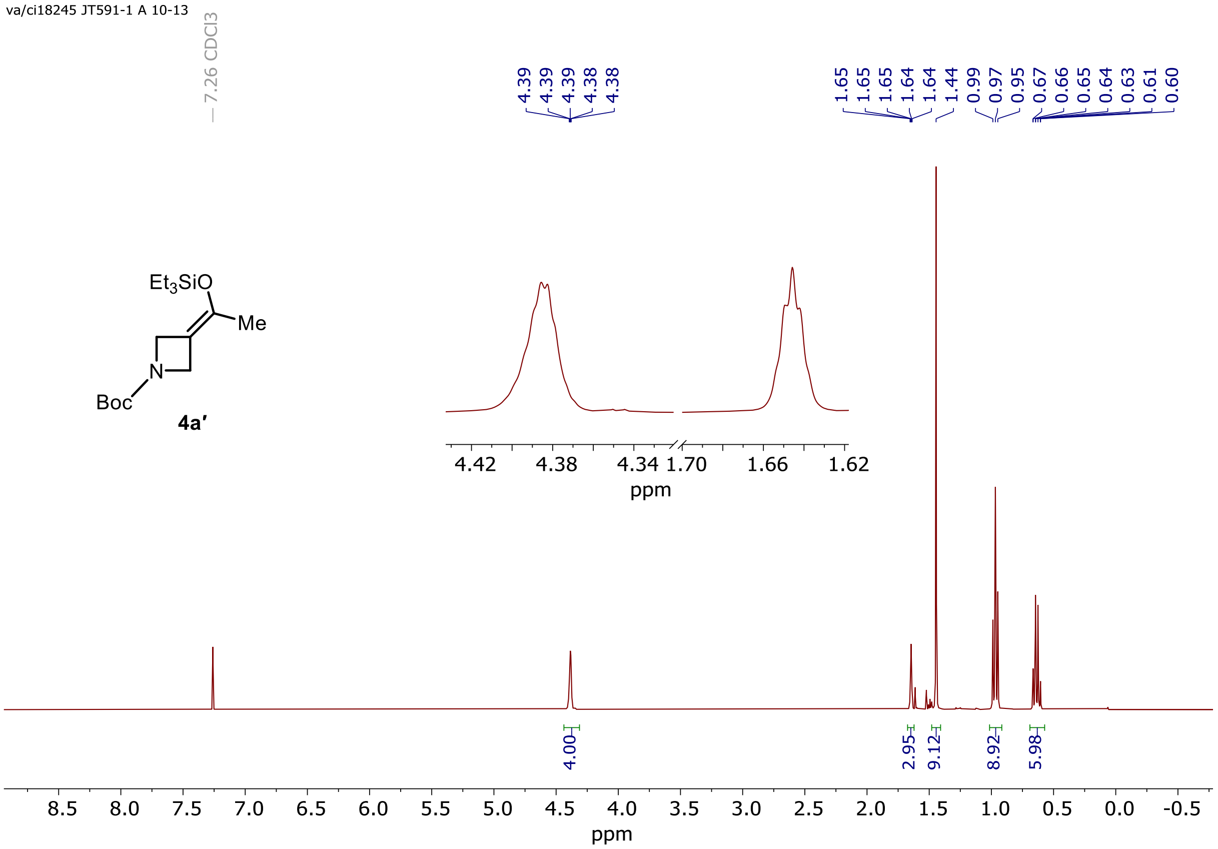

^{13}C NMR (126 MHz, $CDCl_3$) of **4a′**

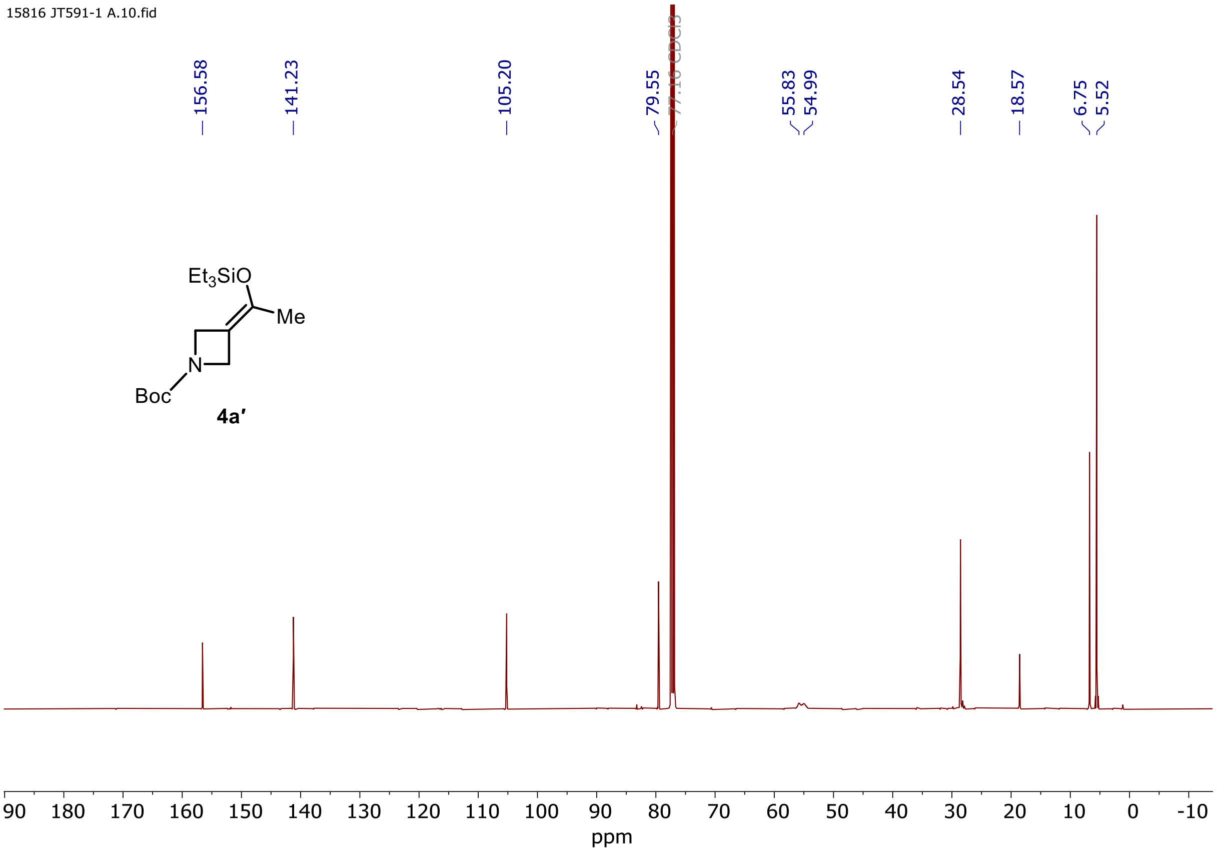

^{1}H NMR (400 MHz, $CDCl_3$) of **4a″** (see procedure)

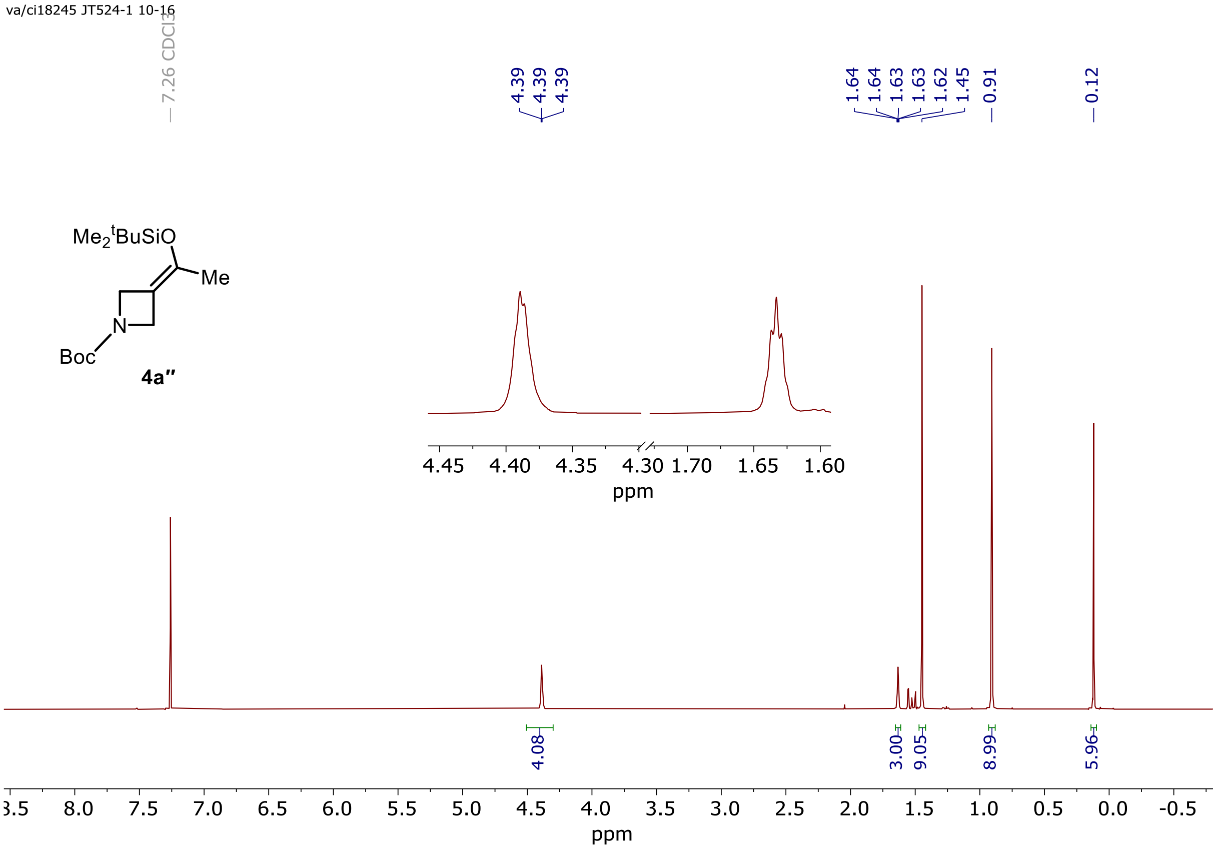

^{13}C NMR (126 MHz, $CDCl_3$) of **4a″**

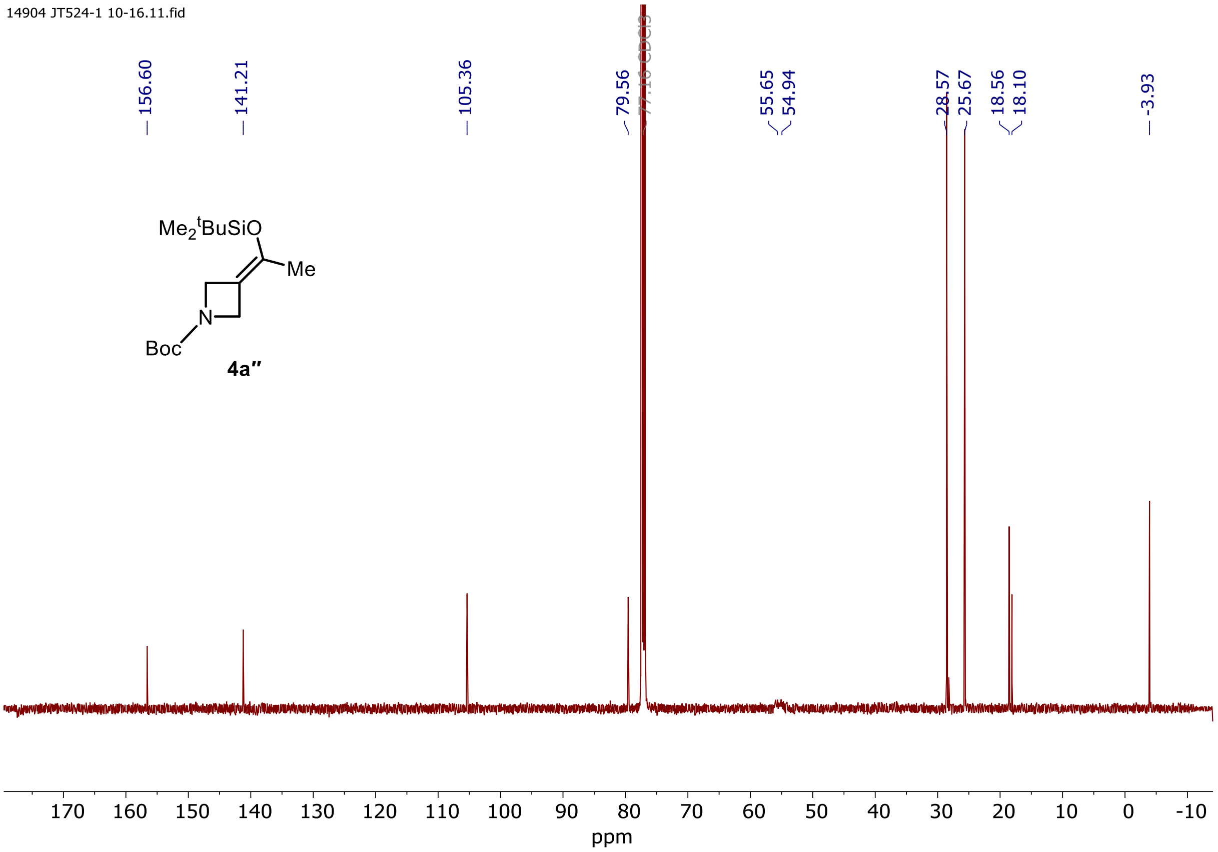

^{1}H NMR (400 MHz, $CDCl_3$) of **5a** (*see procedure*)

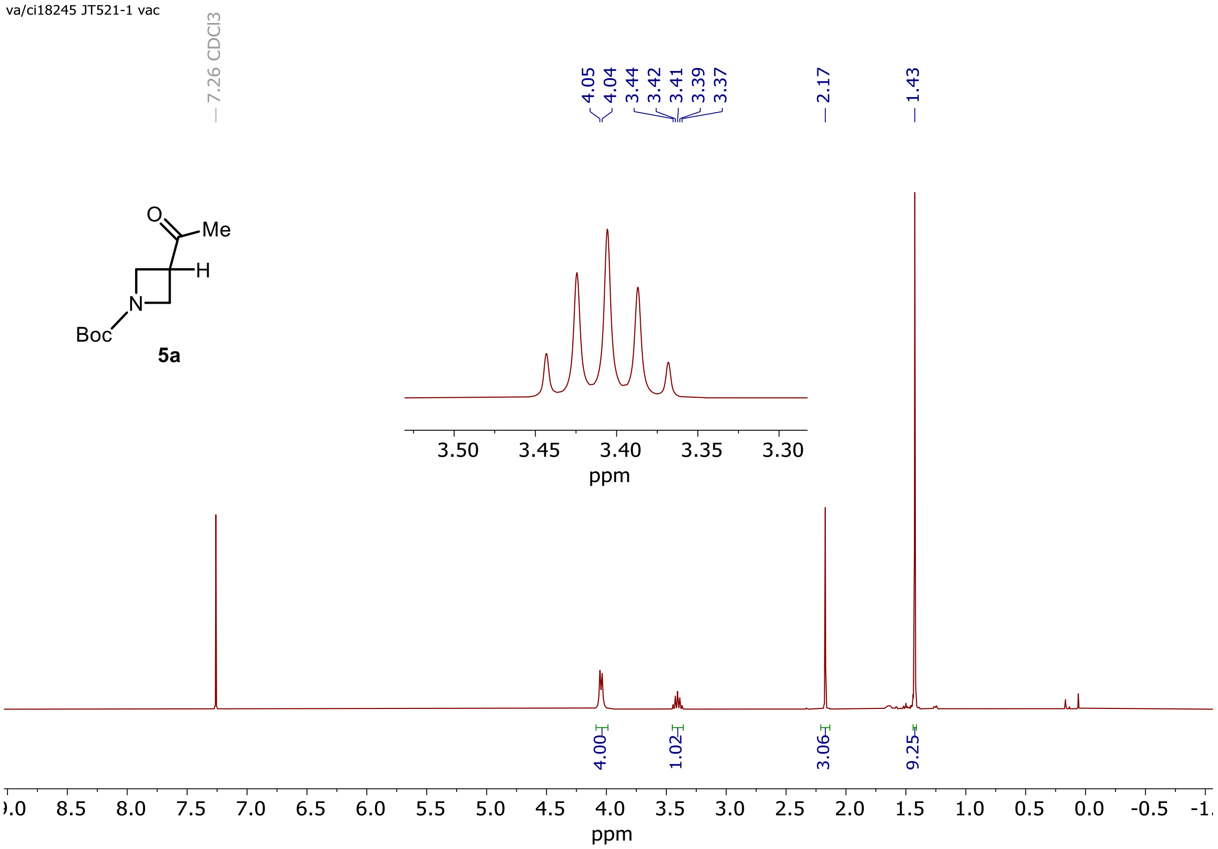

^{13}C NMR (126 MHz, $CDCl_3$) of **5a**

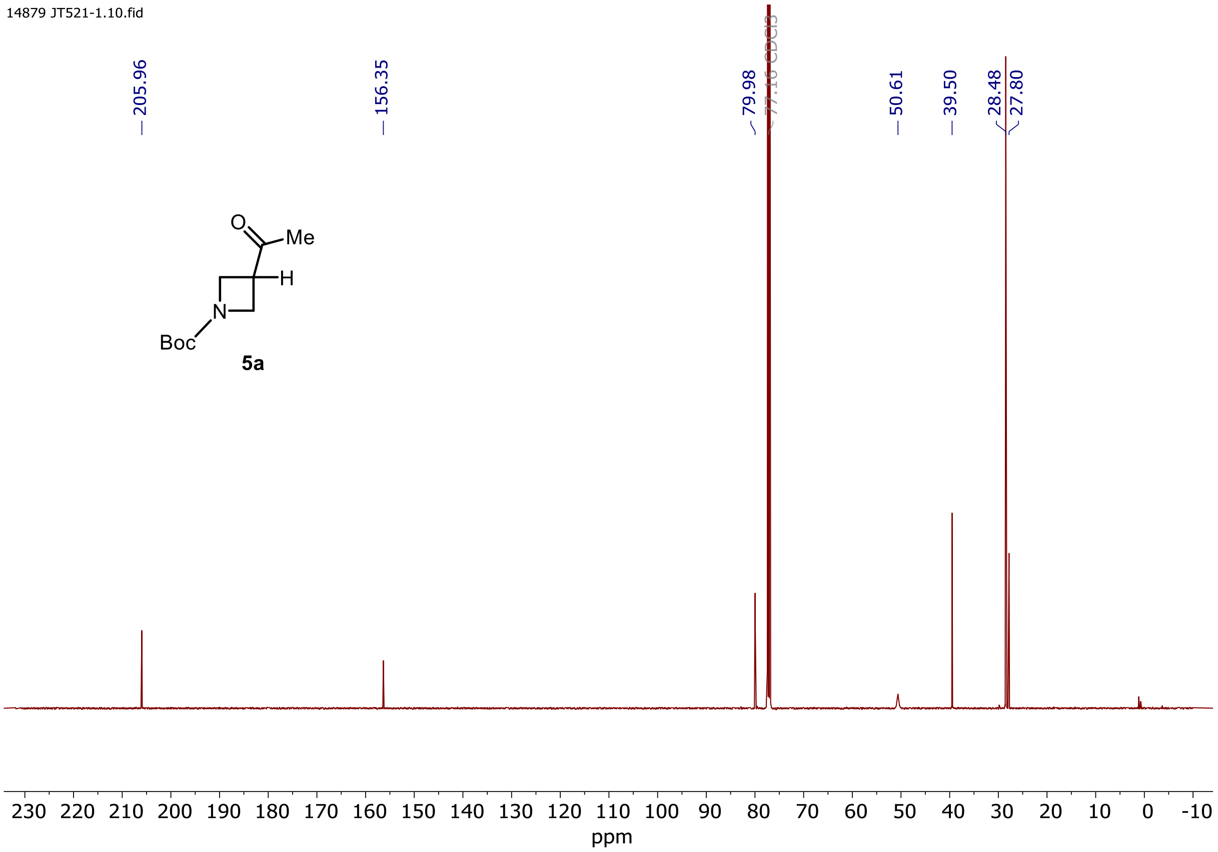

^{1}H NMR (500 MHz, $CDCl_3$) of **5b** (*see procedure*)

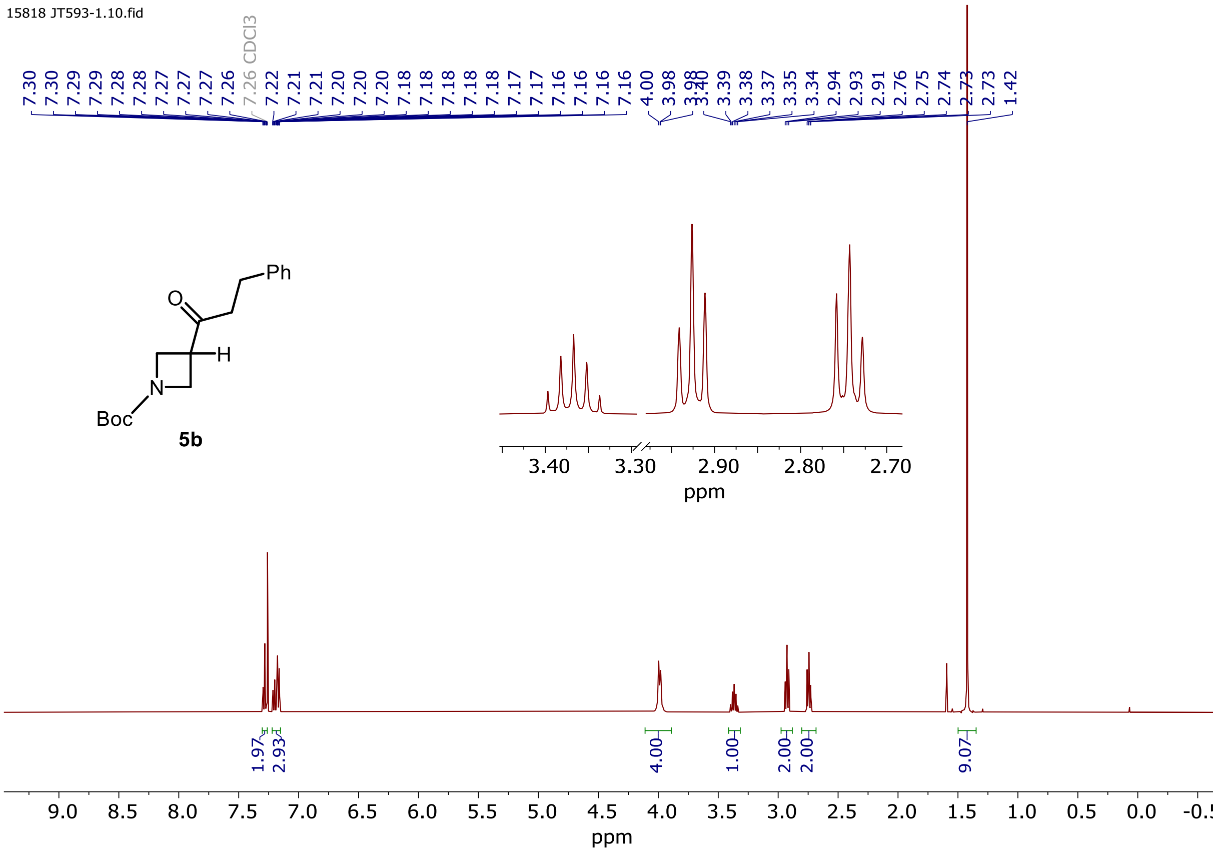

^{13}C NMR (126 MHz, $CDCl_3$) of **5b**

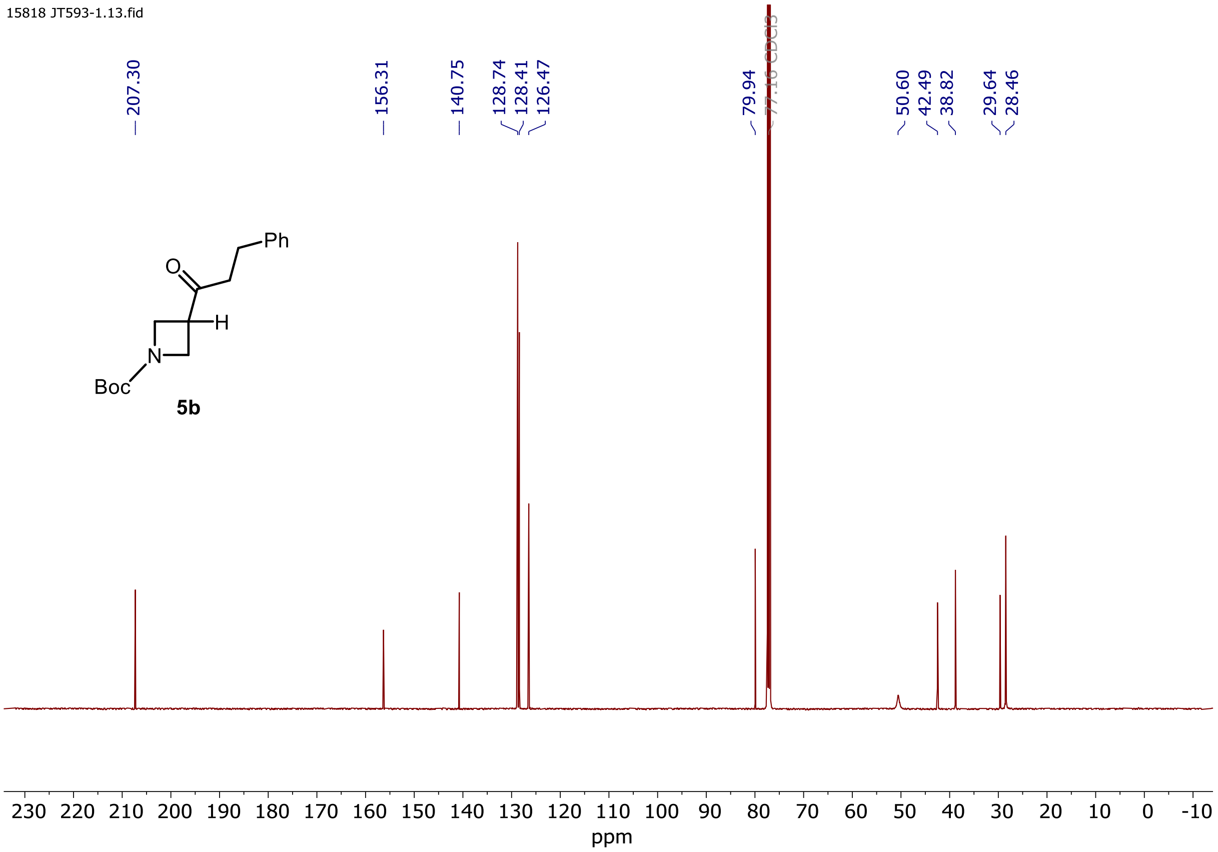

^{1}H NMR (500 MHz, $CDCl_3$) of **5c** (*see procedure*)

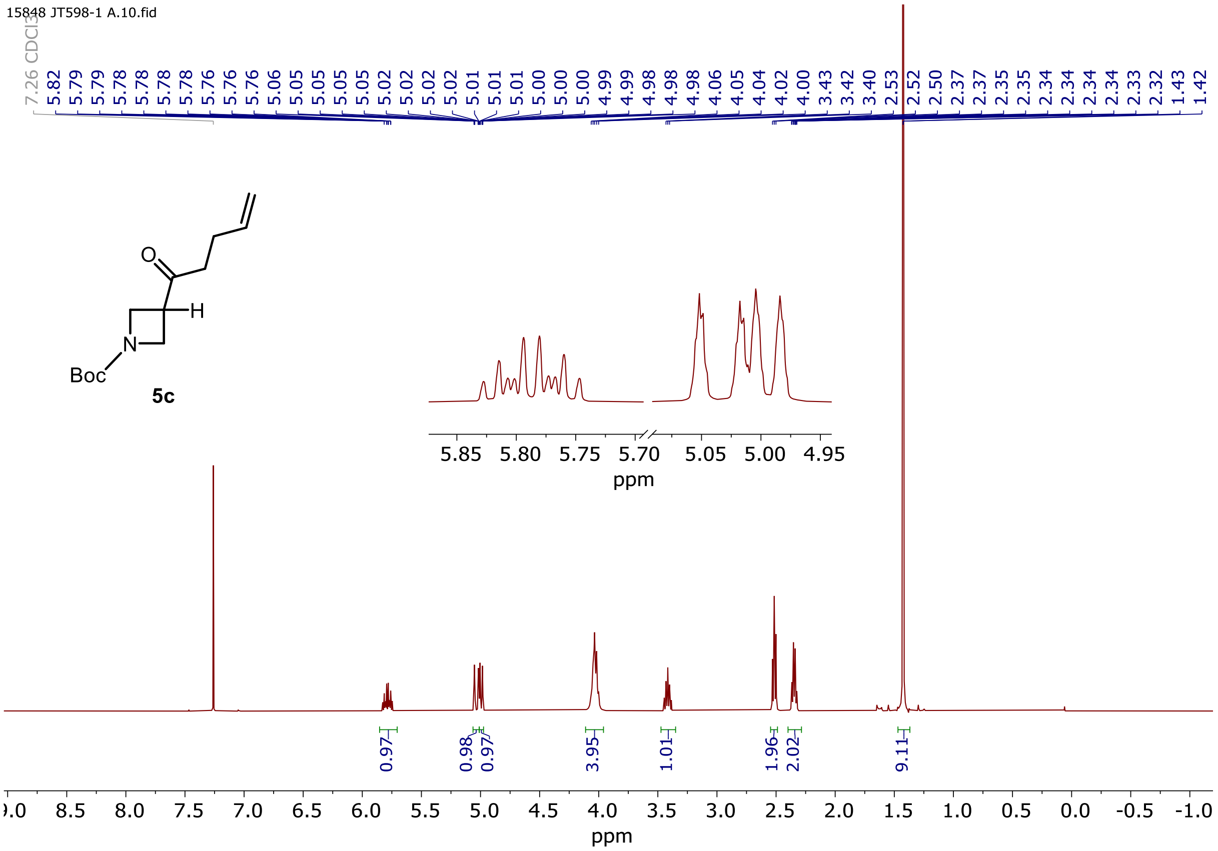

^{13}C NMR (126 MHz, $CDCl_3$) of **5c**

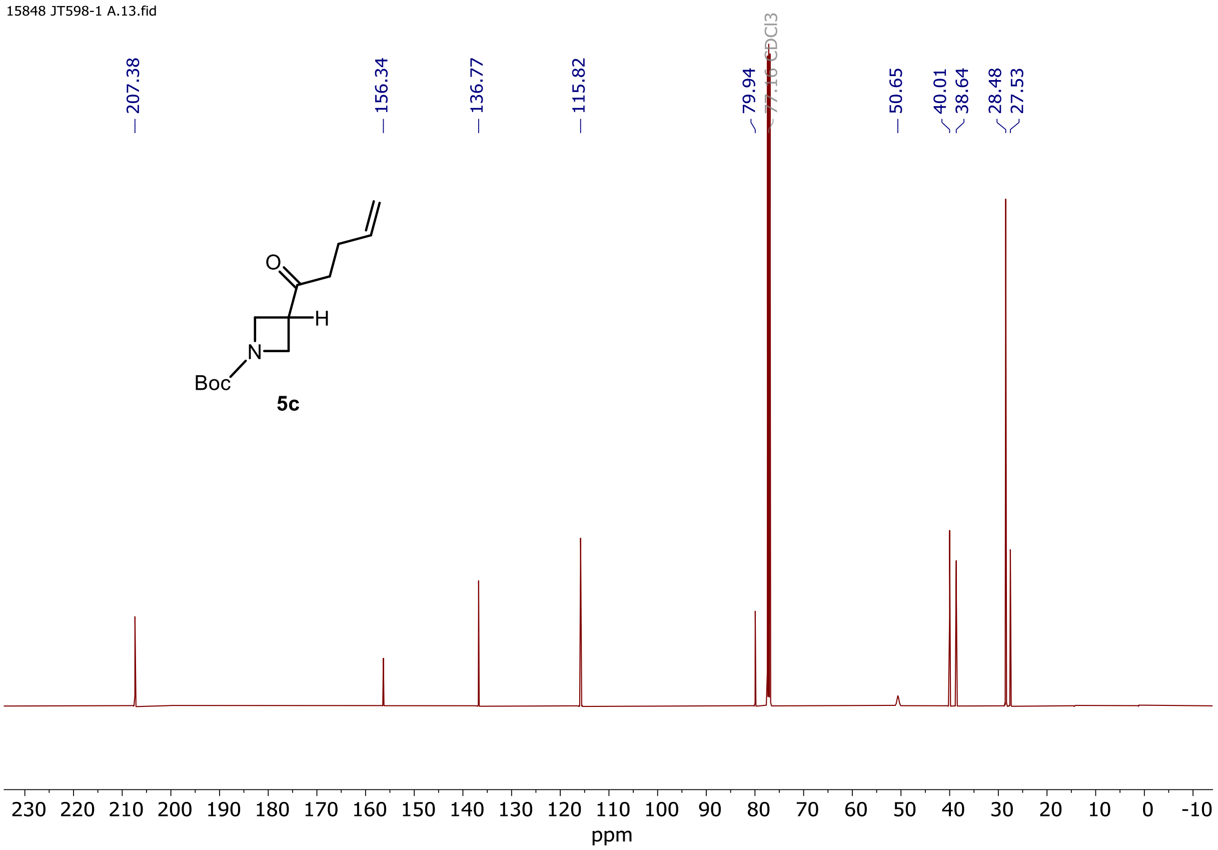

^{1}H NMR (500 MHz, $CDCl_3$) of **5d** (*see procedure*)

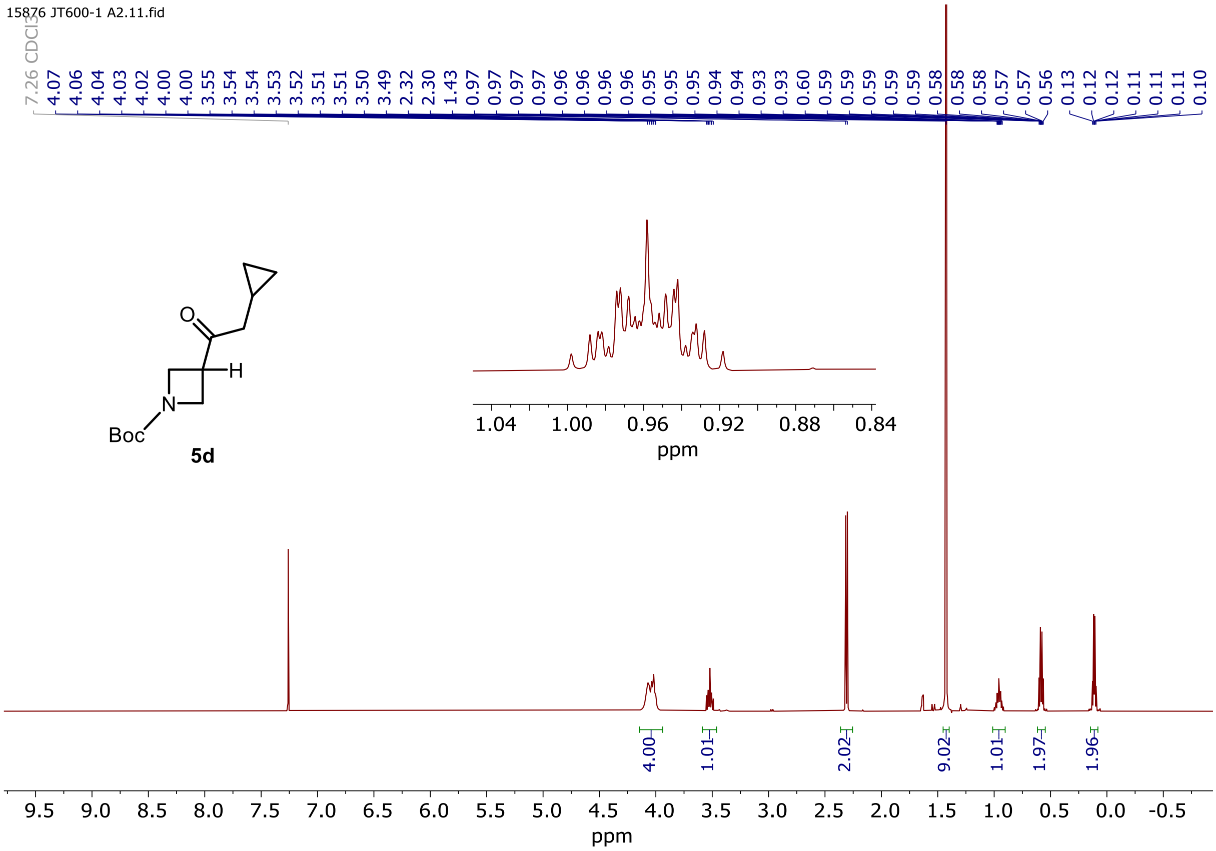

^{13}C NMR (126 MHz, $CDCl_3$) of **5d**

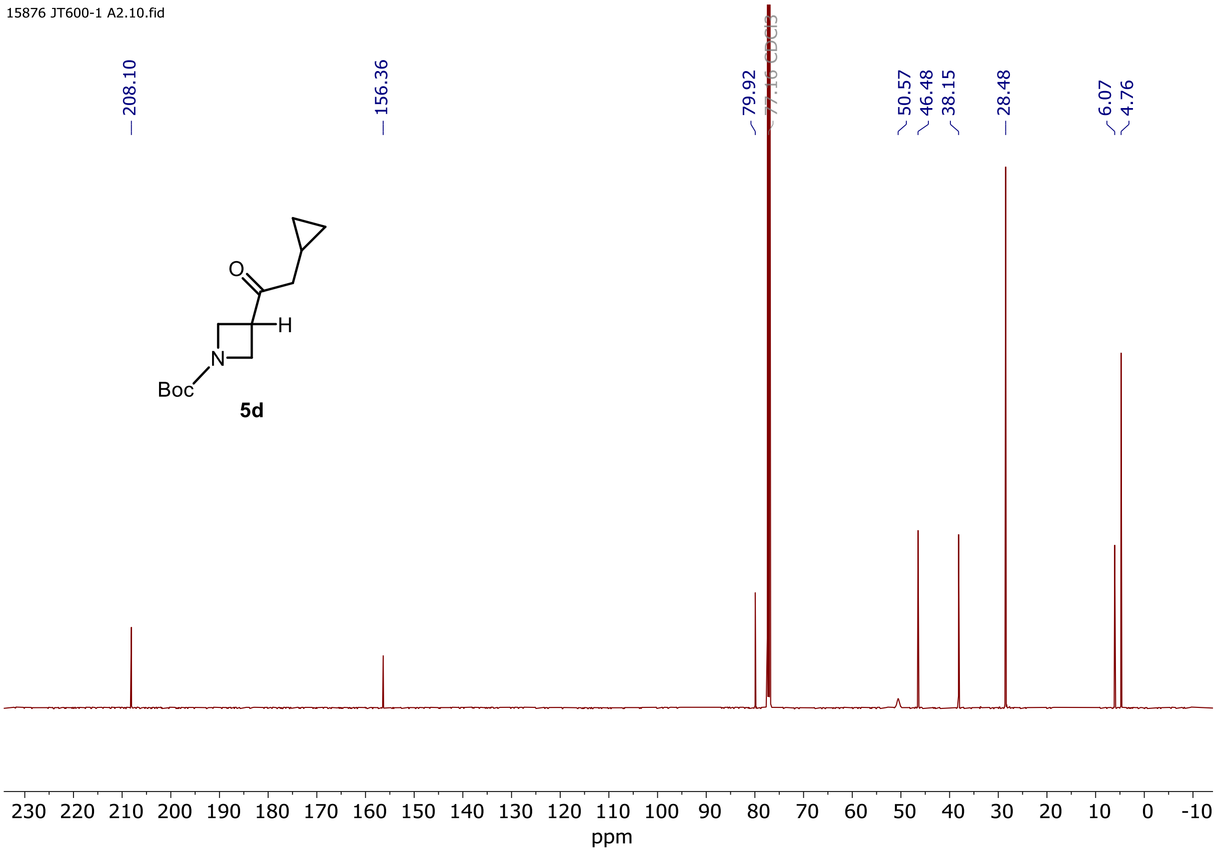

^{1}H NMR (500 MHz, $CDCl_3$) of **5e** (*see procedure*)

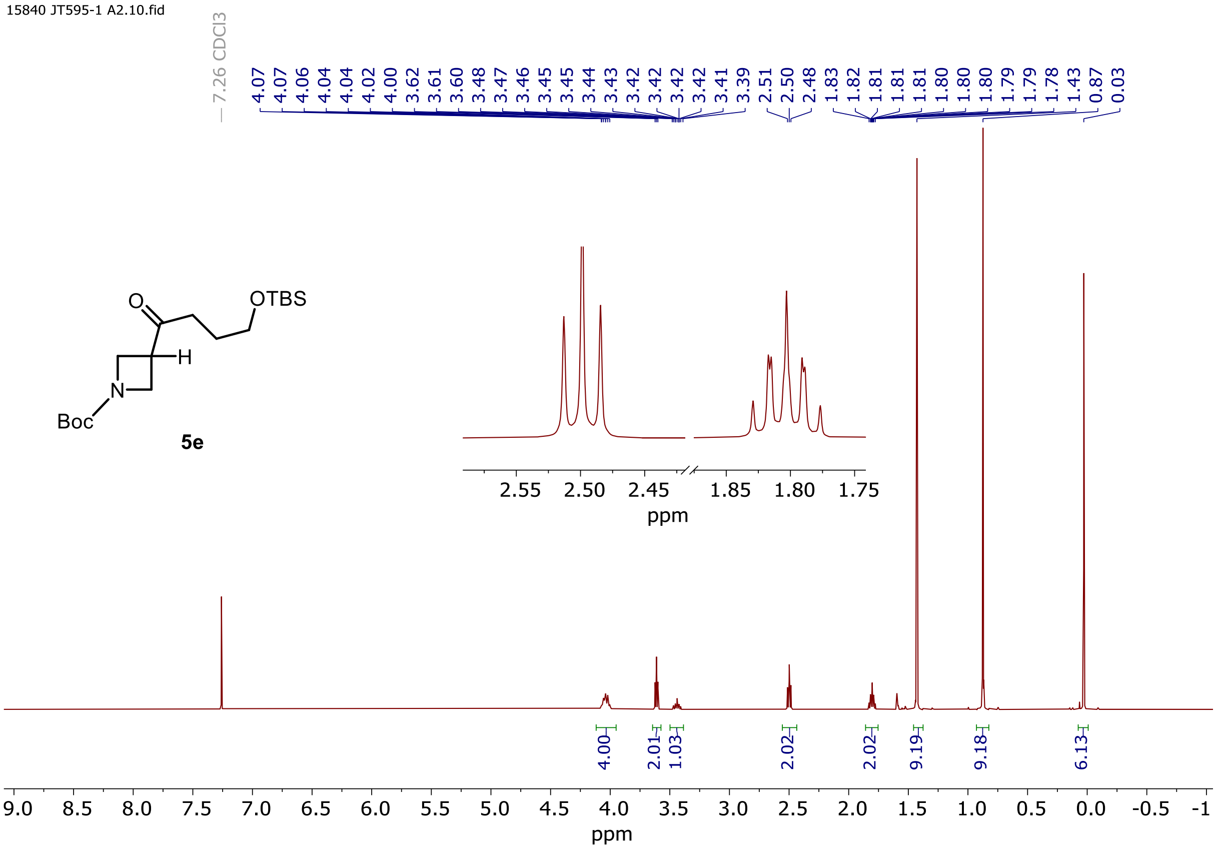

^{13}C NMR (126 MHz, $CDCl_3$) of **5e**

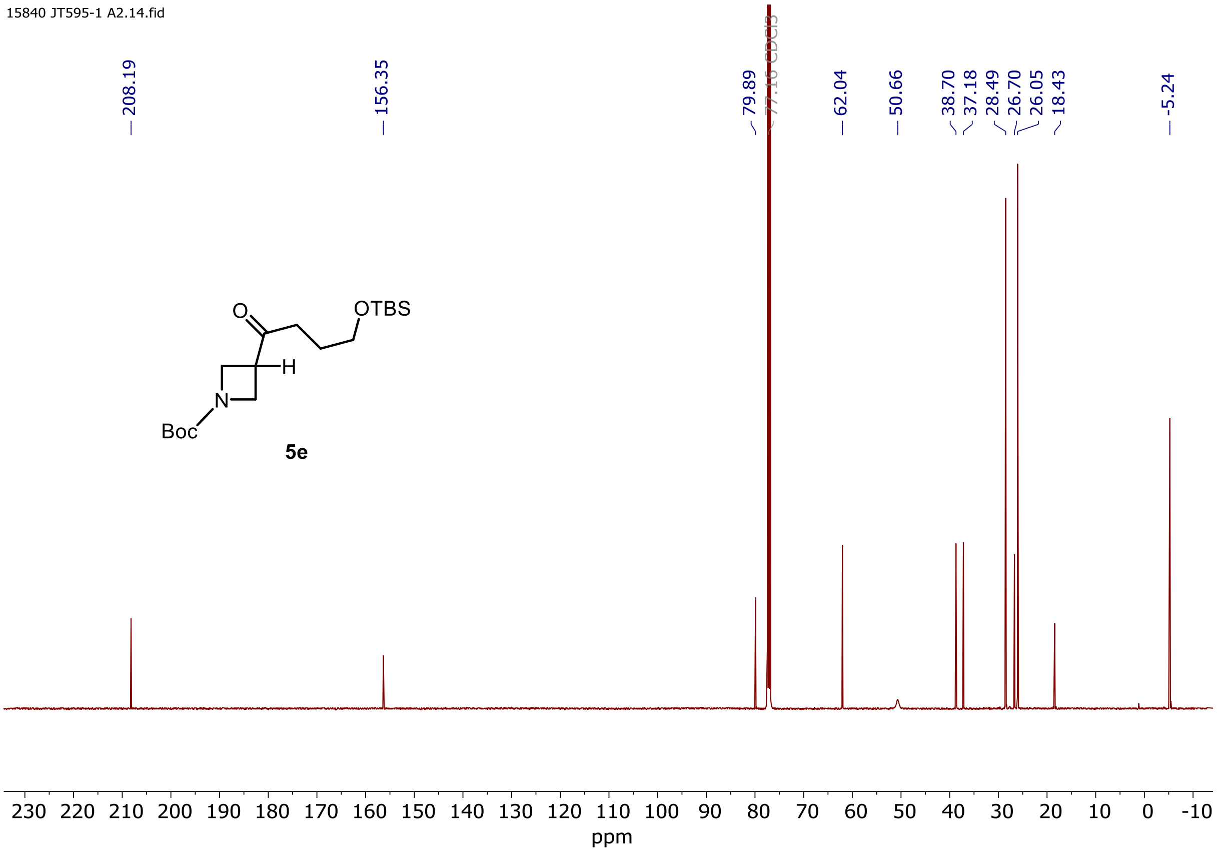

^{1}H NMR (500 MHz, $CDCl_3$) of **5f** (*see procedure*)

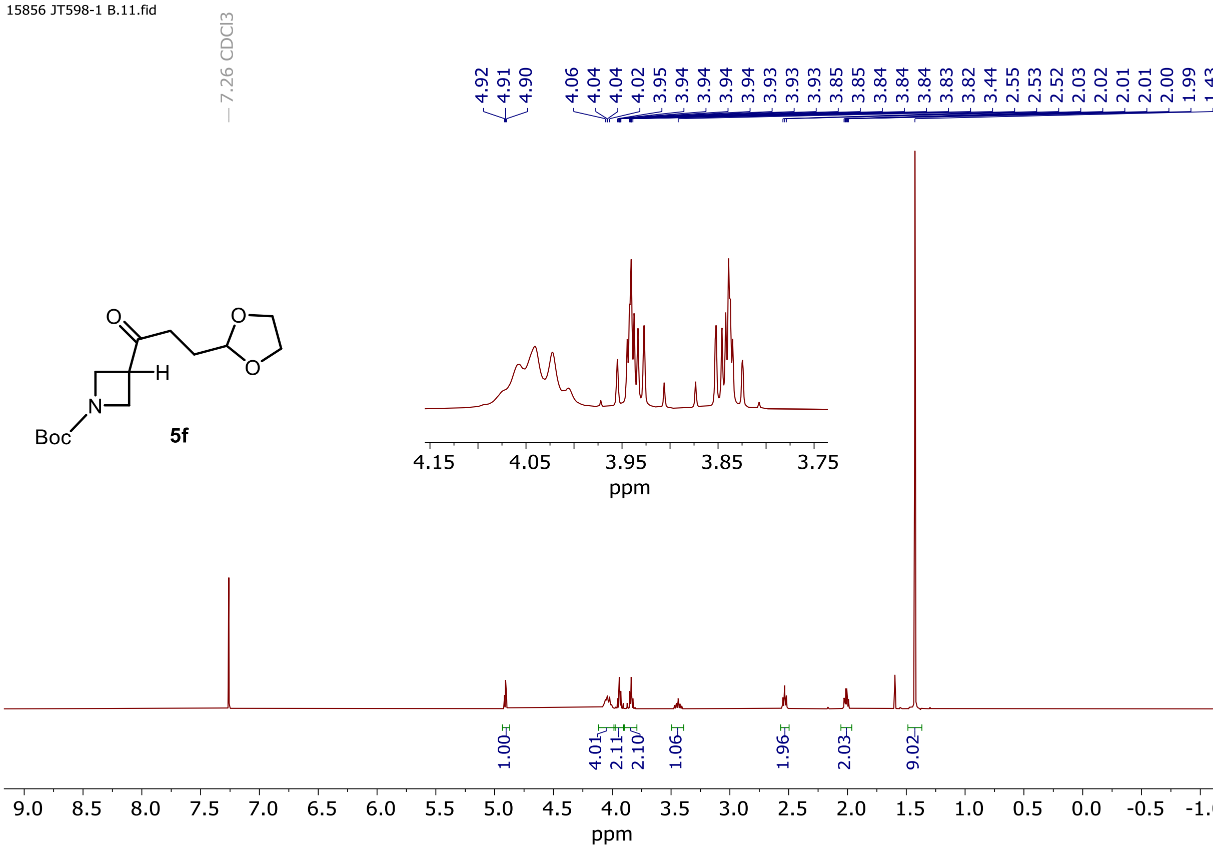

^{13}C NMR (126 MHz, $CDCl_3$) of **5f**

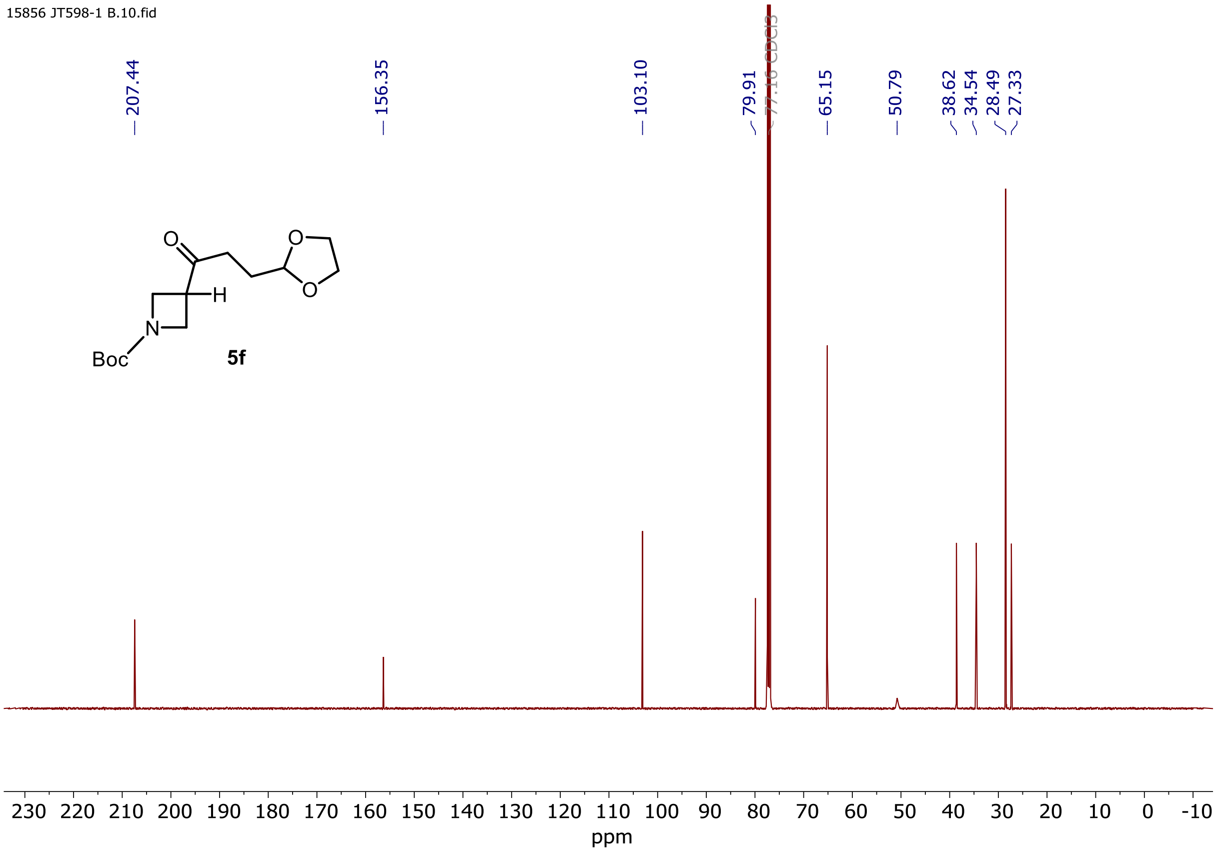

^{1}H NMR (500 MHz, $CDCl_3$) of **5g** (*see procedure*)

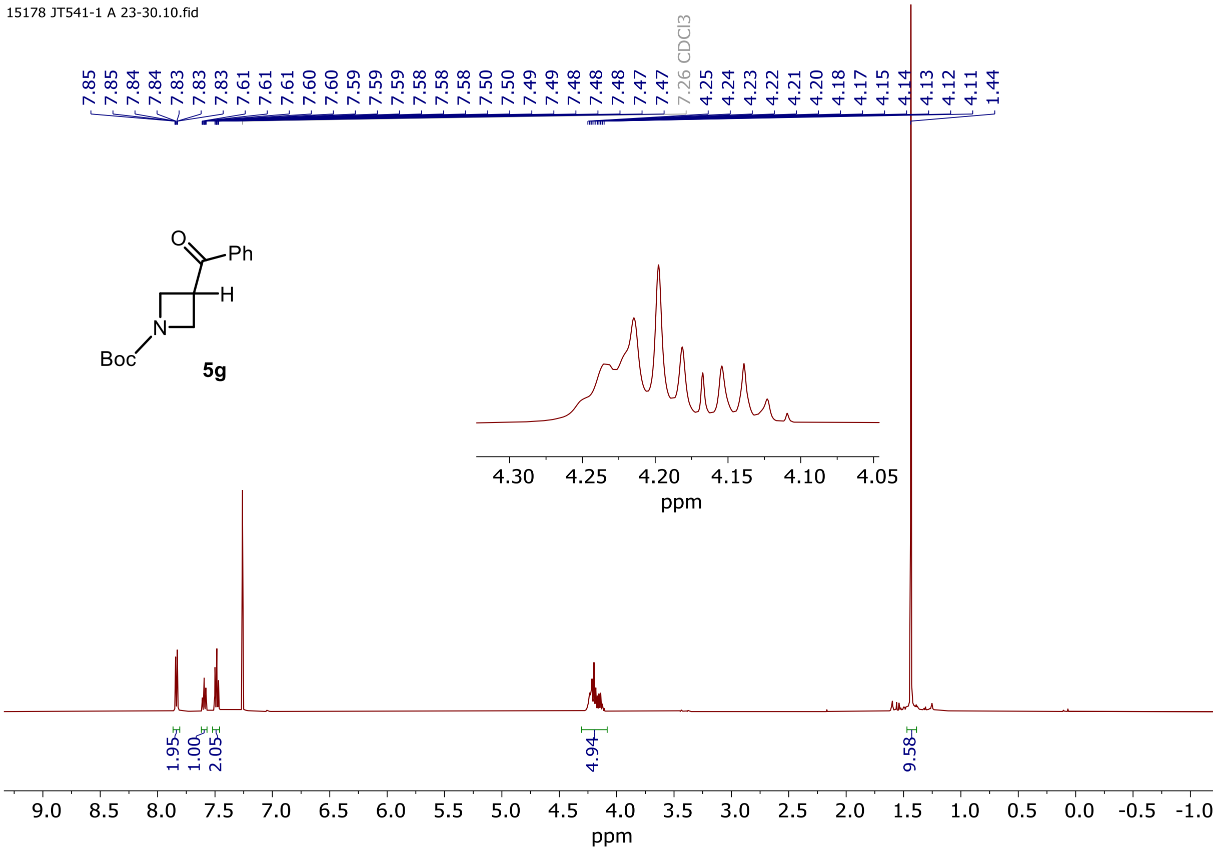

^{13}C NMR (126 MHz, $CDCl_3$) of **5g**

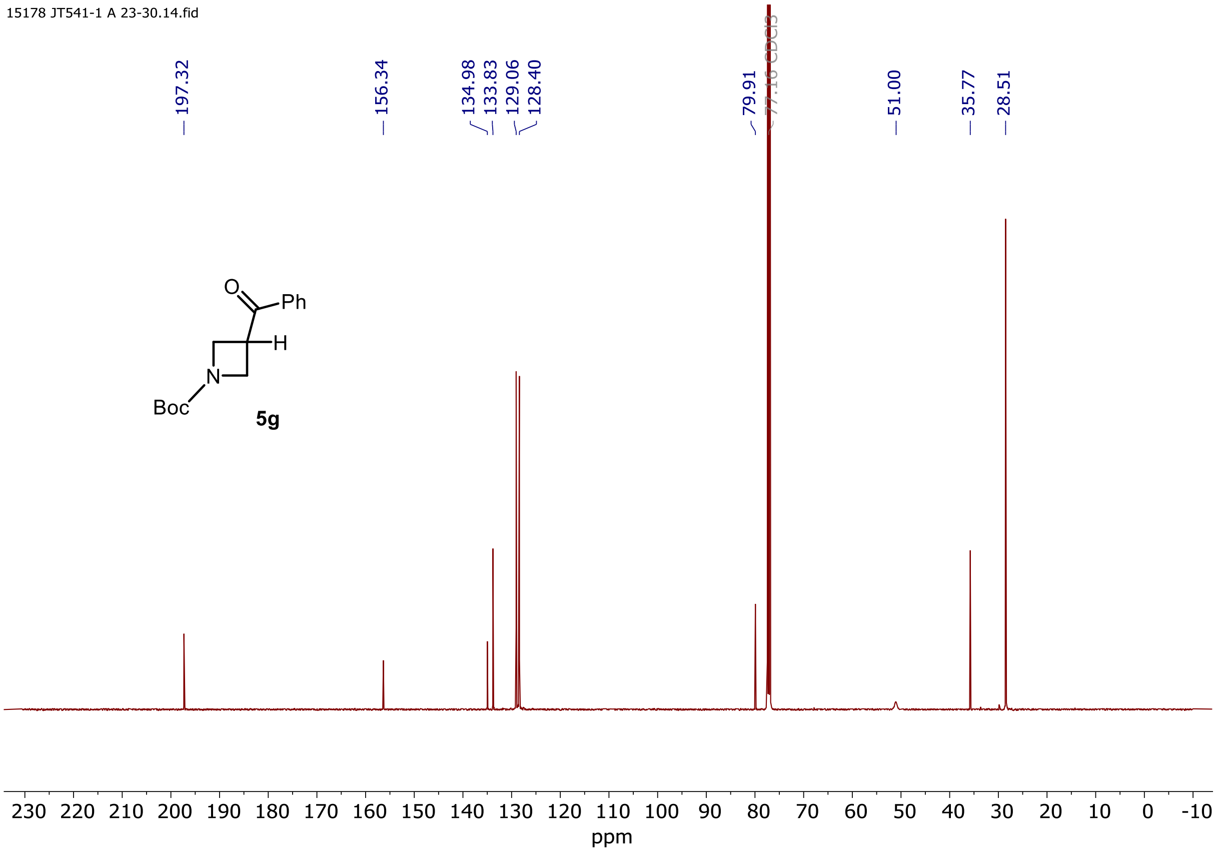

^{1}H NMR (500 MHz, $CDCl_3$) of **5h** (*see procedure*)

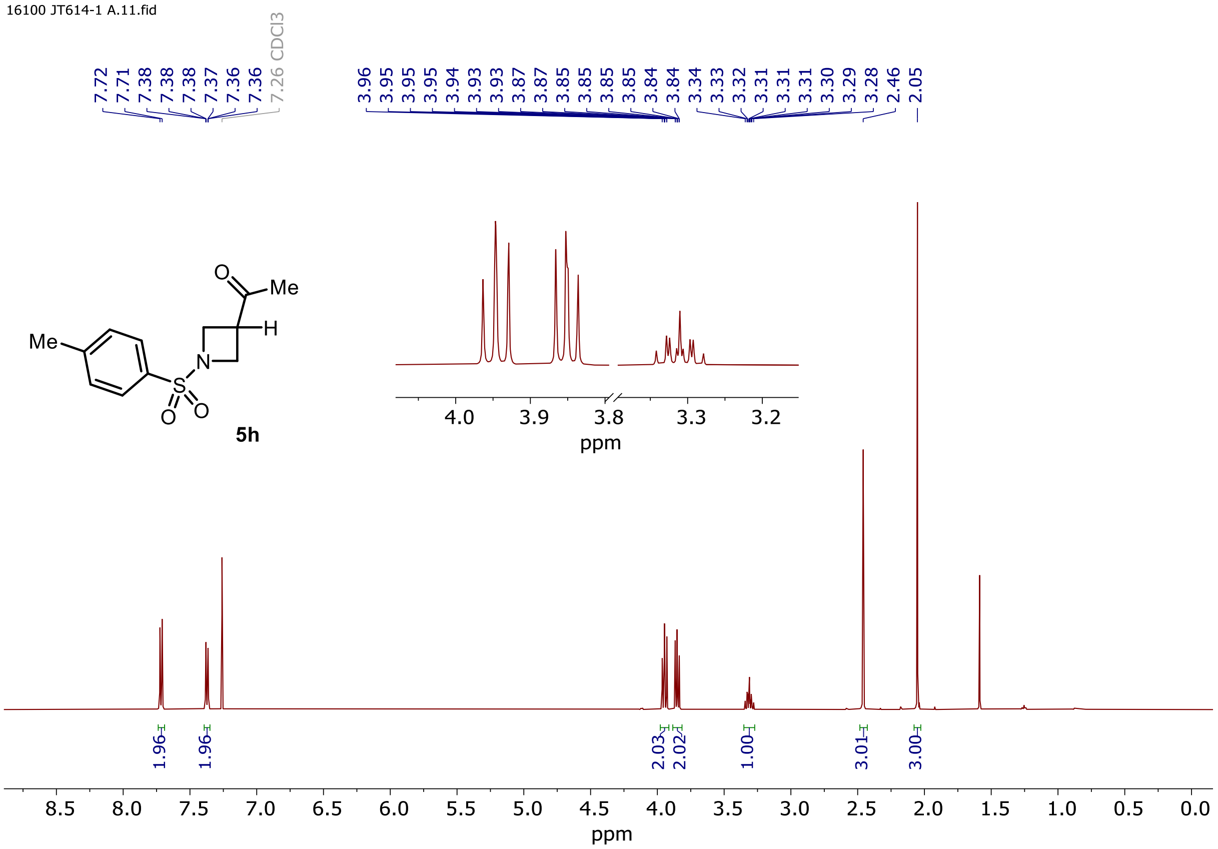

^{13}C NMR (126 MHz, $CDCl_3$) of **5h**

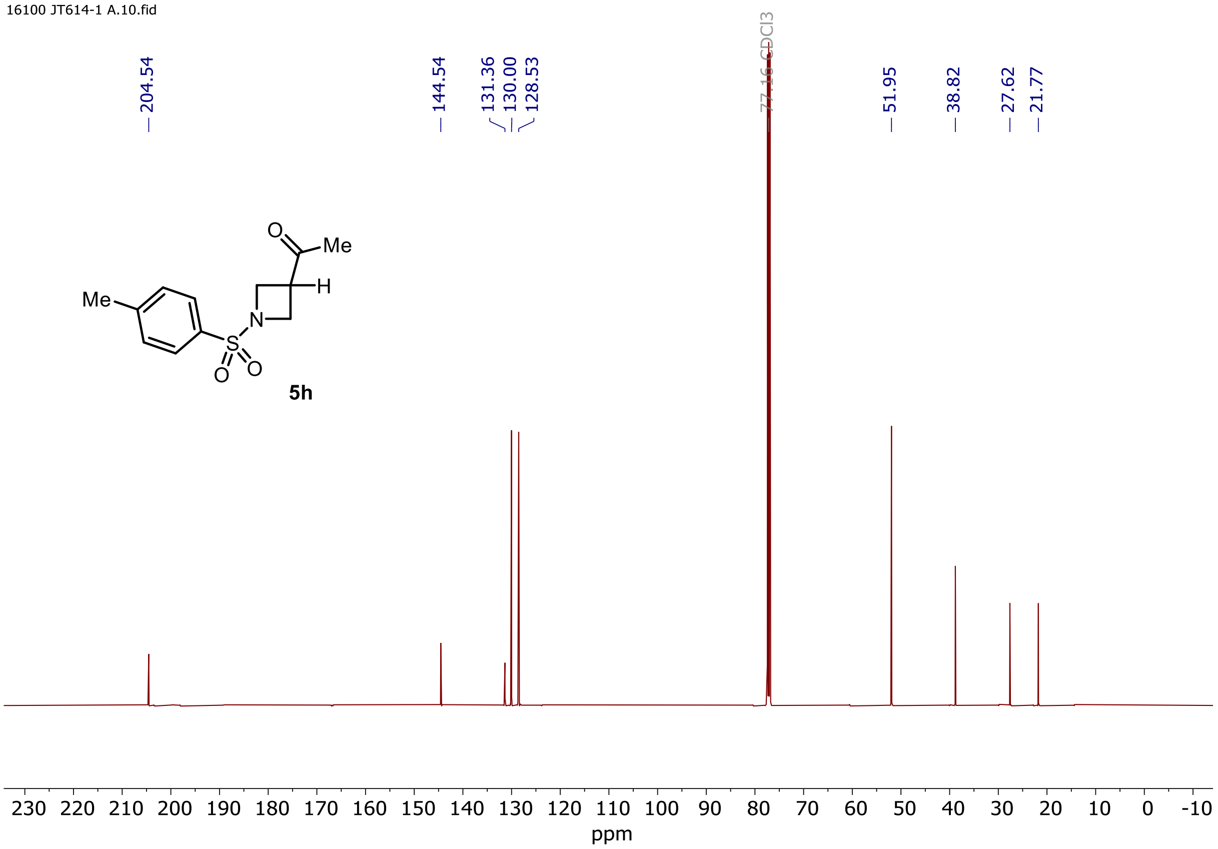

^{1}H NMR (500 MHz, $CDCl_3$) of **5i** (*see procedure*)

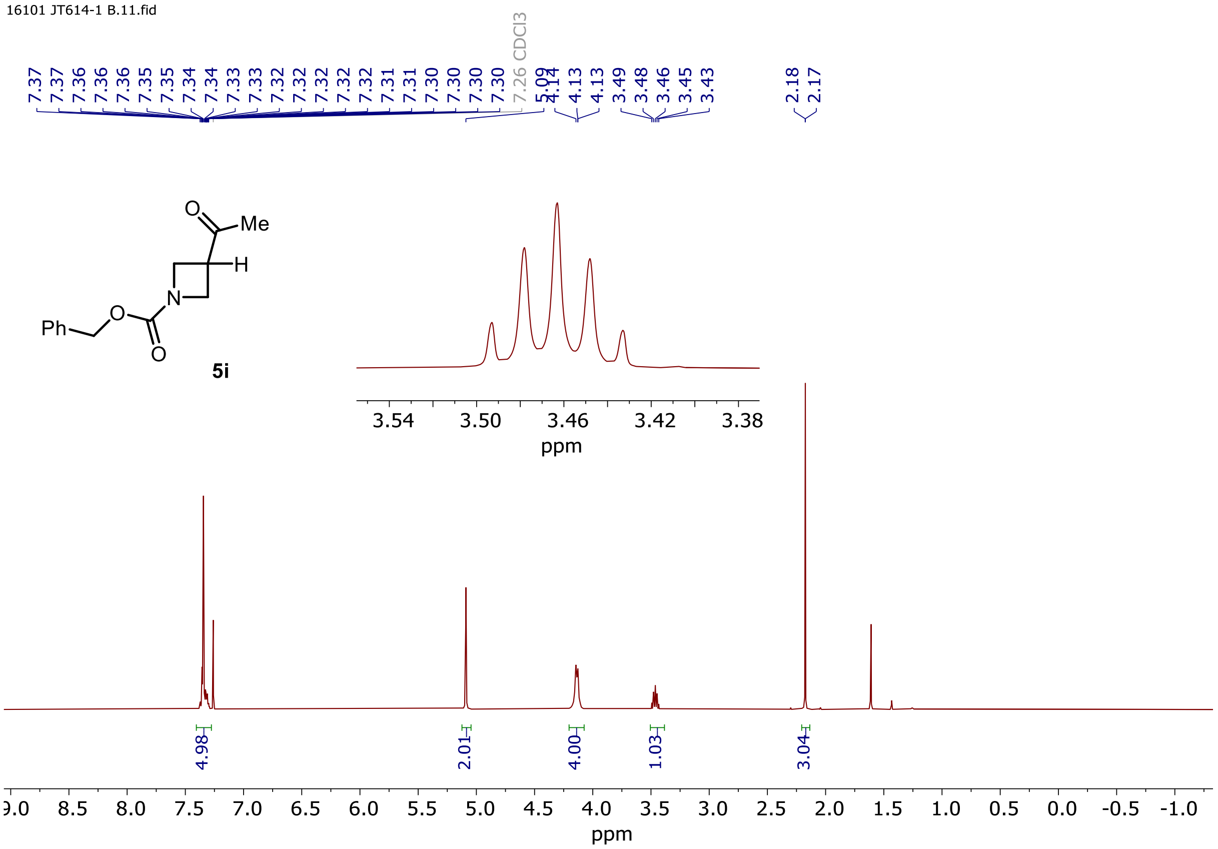

^{13}C NMR (126 MHz, $CDCl_3$) of **5i**

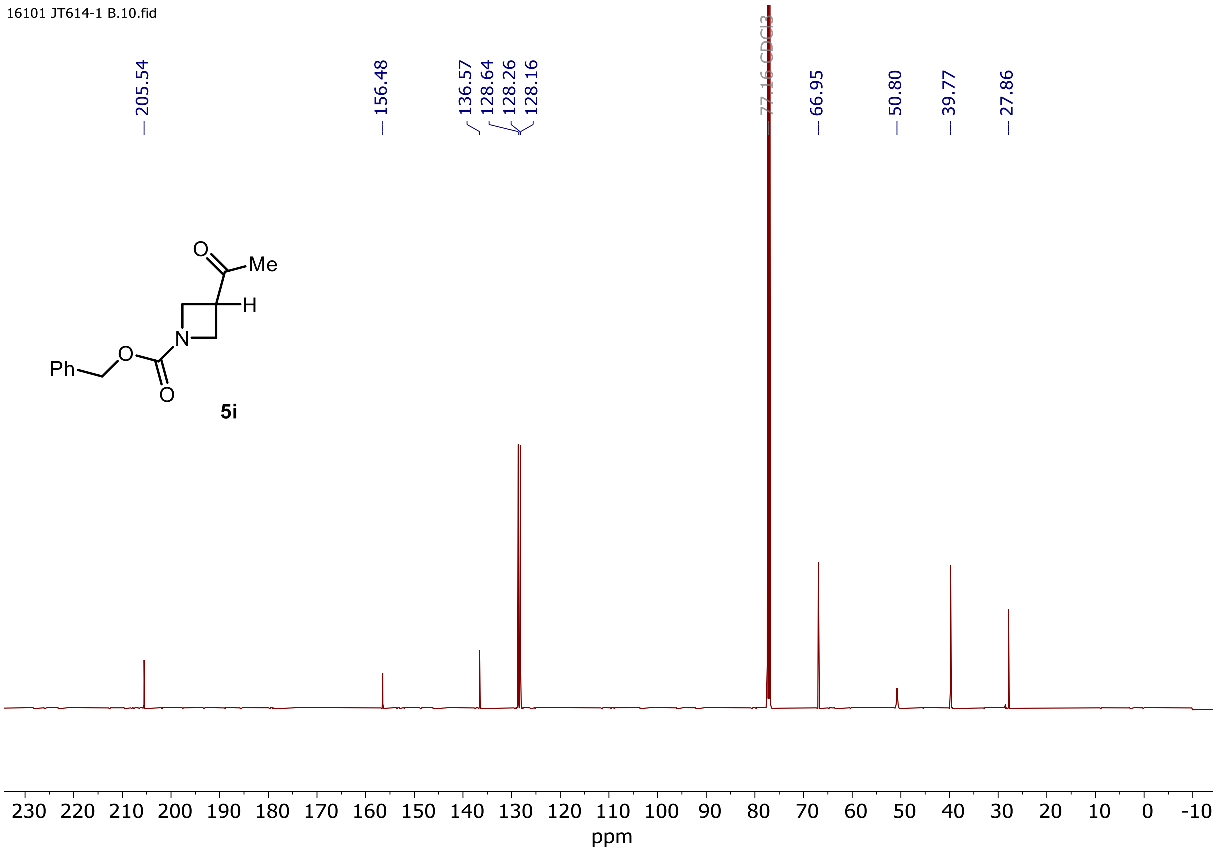

^{1}H NMR (500 MHz, CDCl$_3$) of **5j** (*see procedure*)

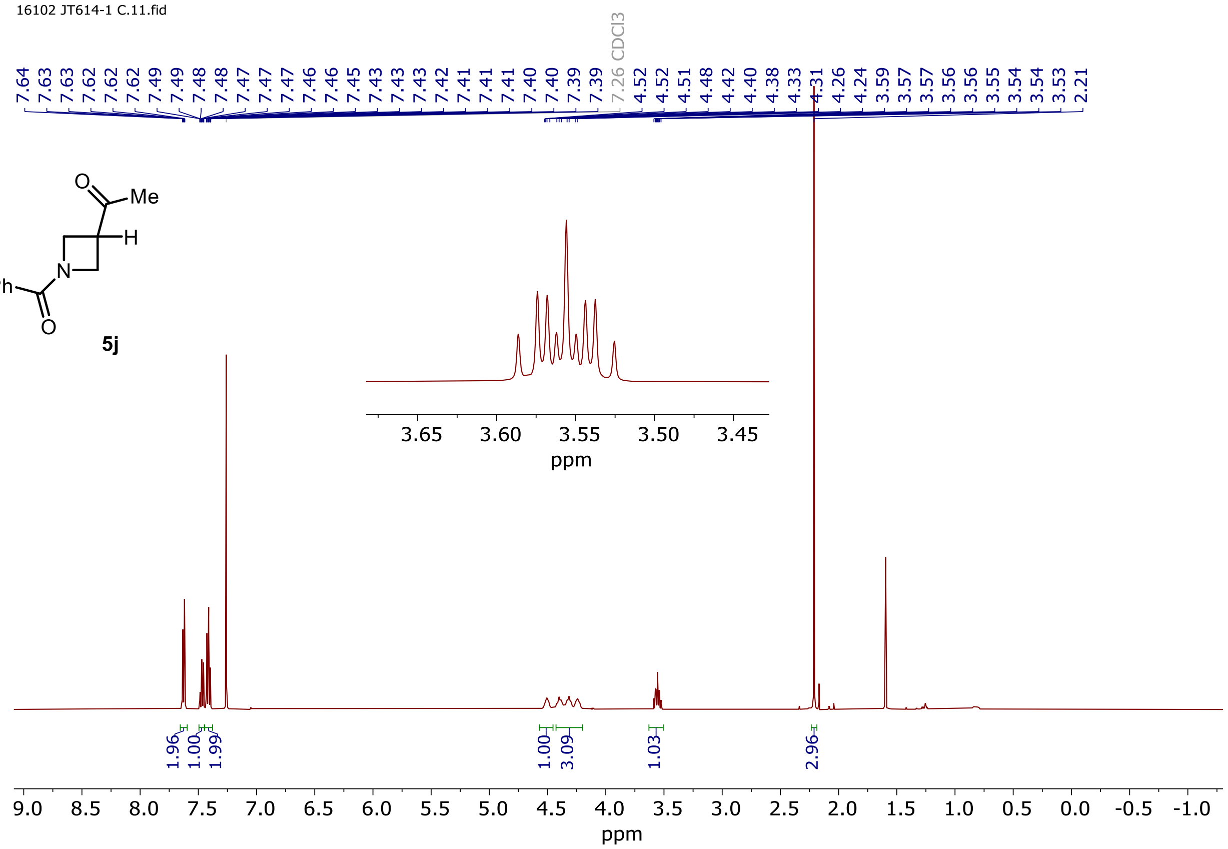

^{13}C NMR (126 MHz, CDCl$_3$) of **5j**

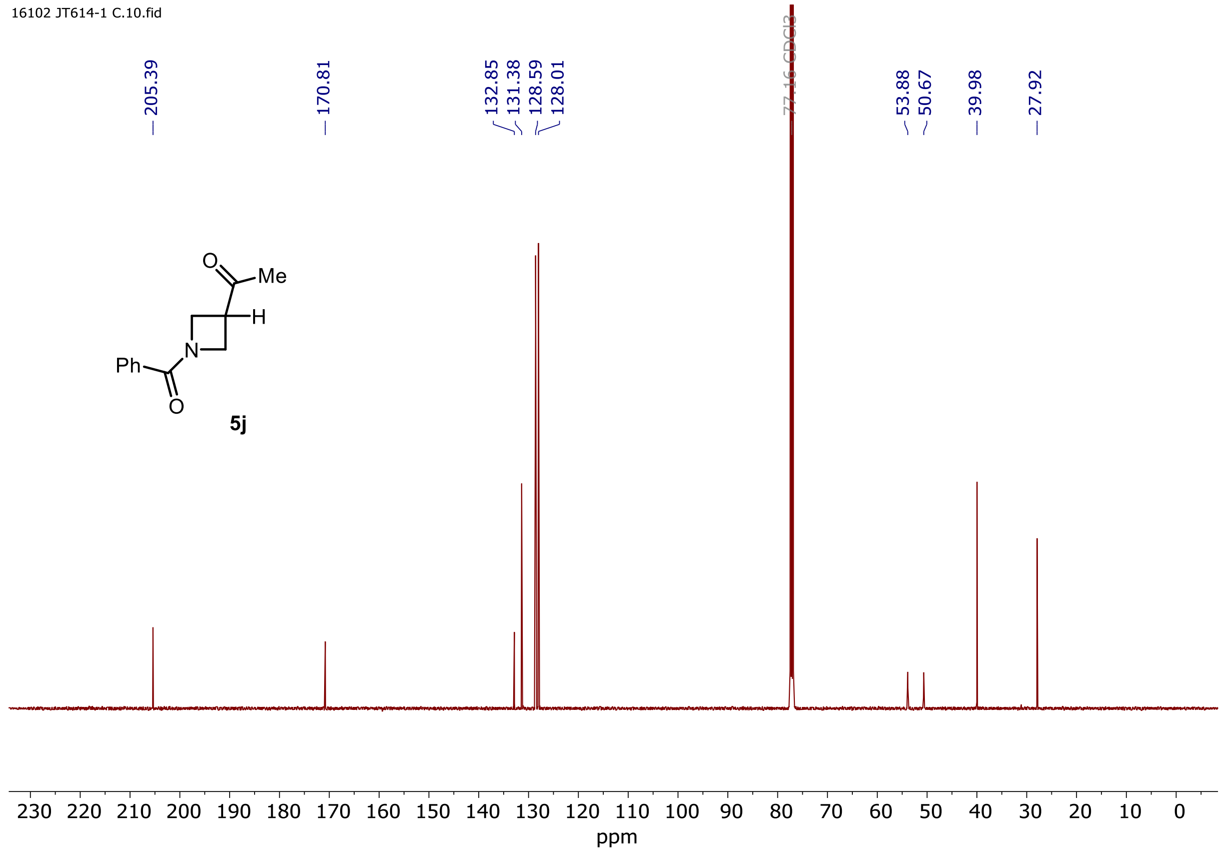

^{1}H NMR (500 MHz, $CDCl_3$) of **5k** (*see procedure*)

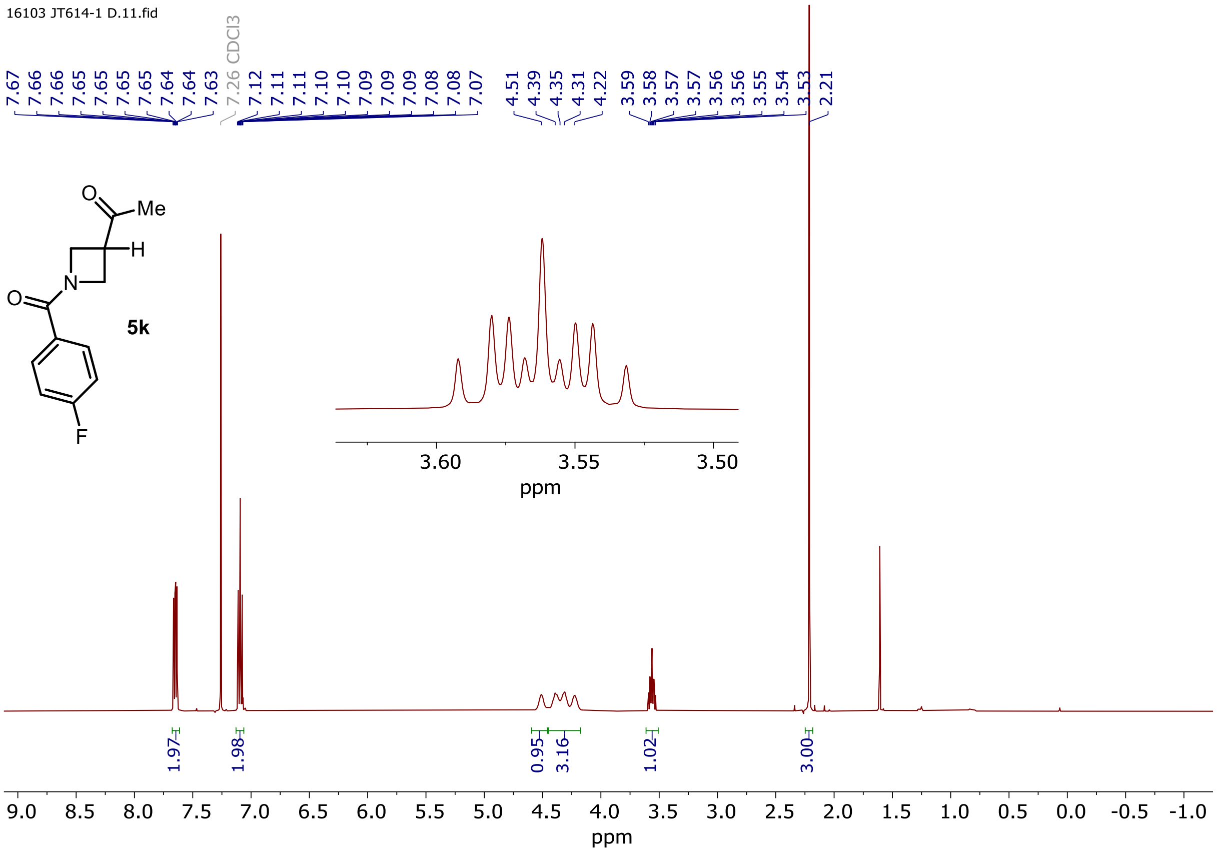

^{13}C NMR (126 MHz, $CDCl_3$) of **5k**

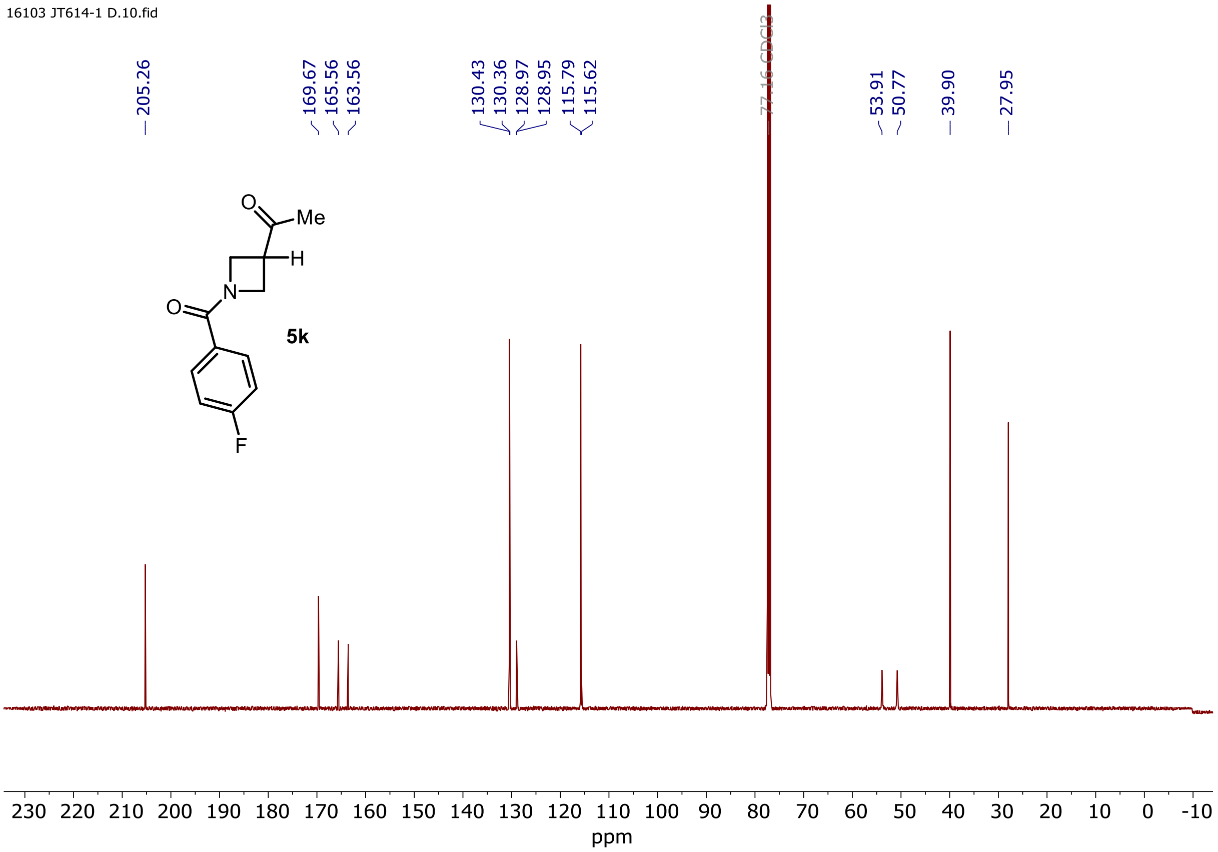

^{19}F NMR (376 MHz, $CDCl_3$) of **5k**

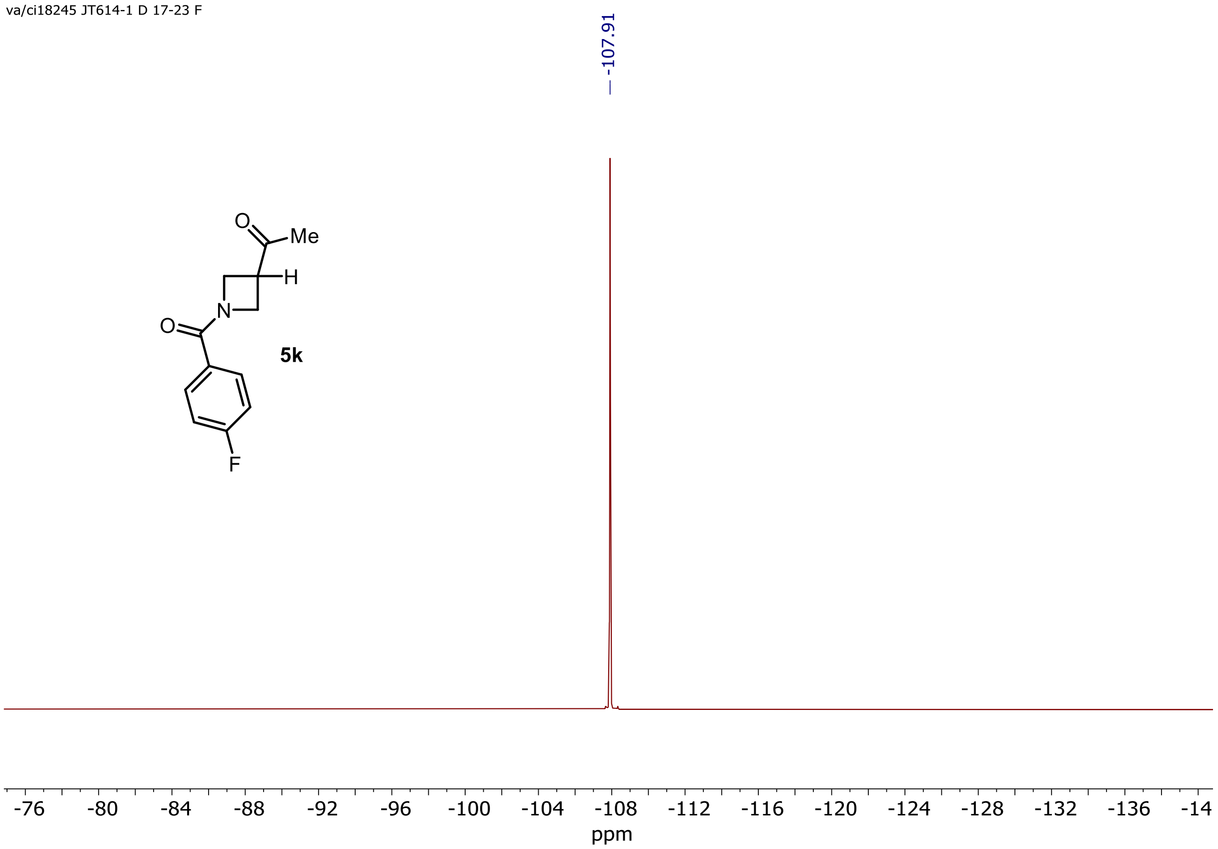

^{1}H NMR (500 MHz, $CDCl_3$) of **5l** (*see procedure*)

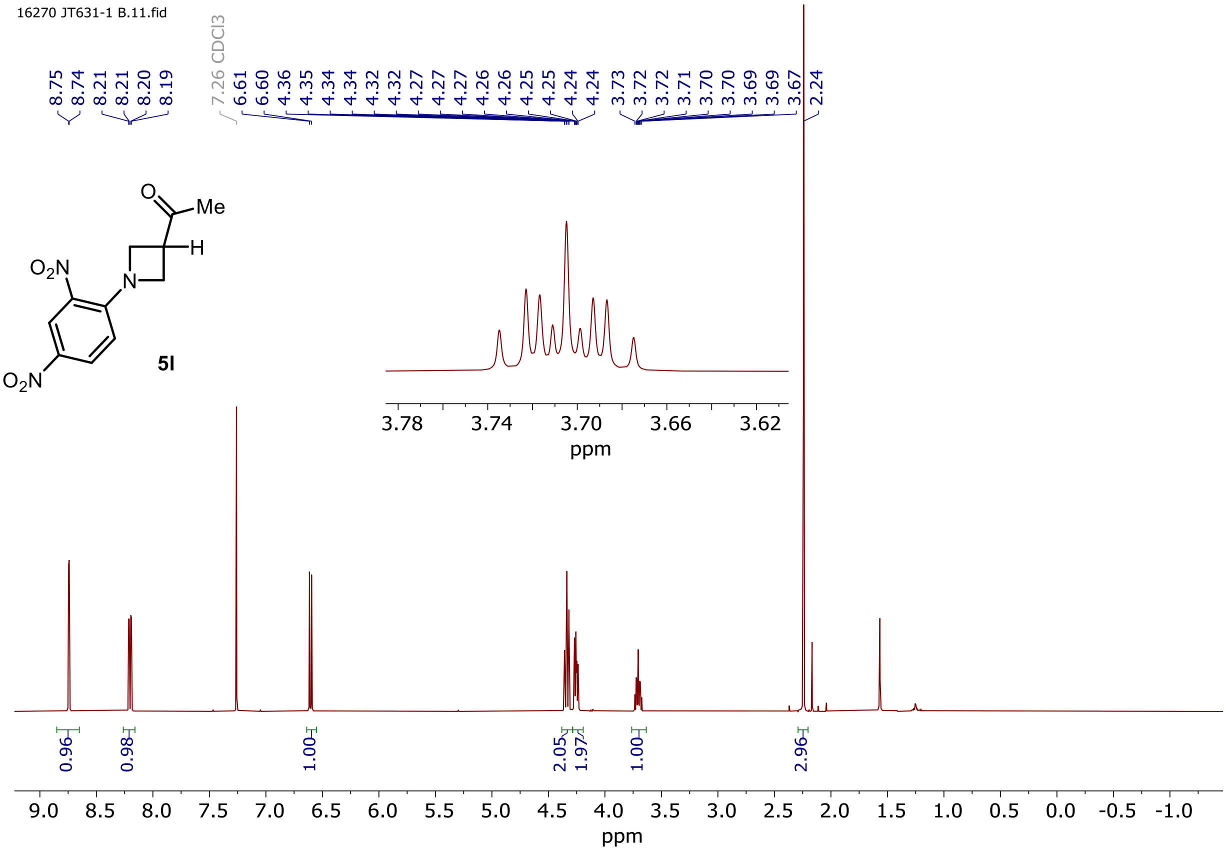

^{13}C NMR (126 MHz, $CDCl_3$) of **5l**

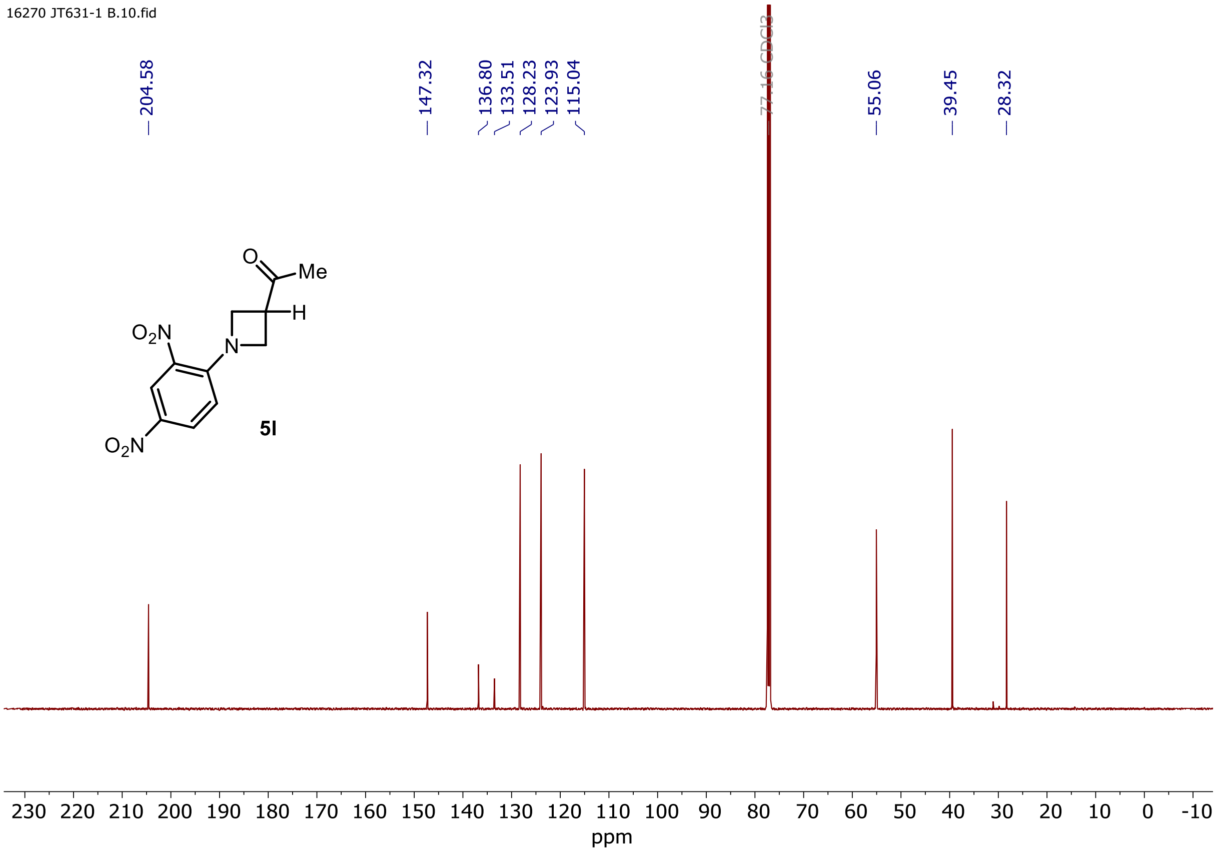

^{1}H NMR (400 MHz, $CDCl_3$) of **5m** (*see procedure*)

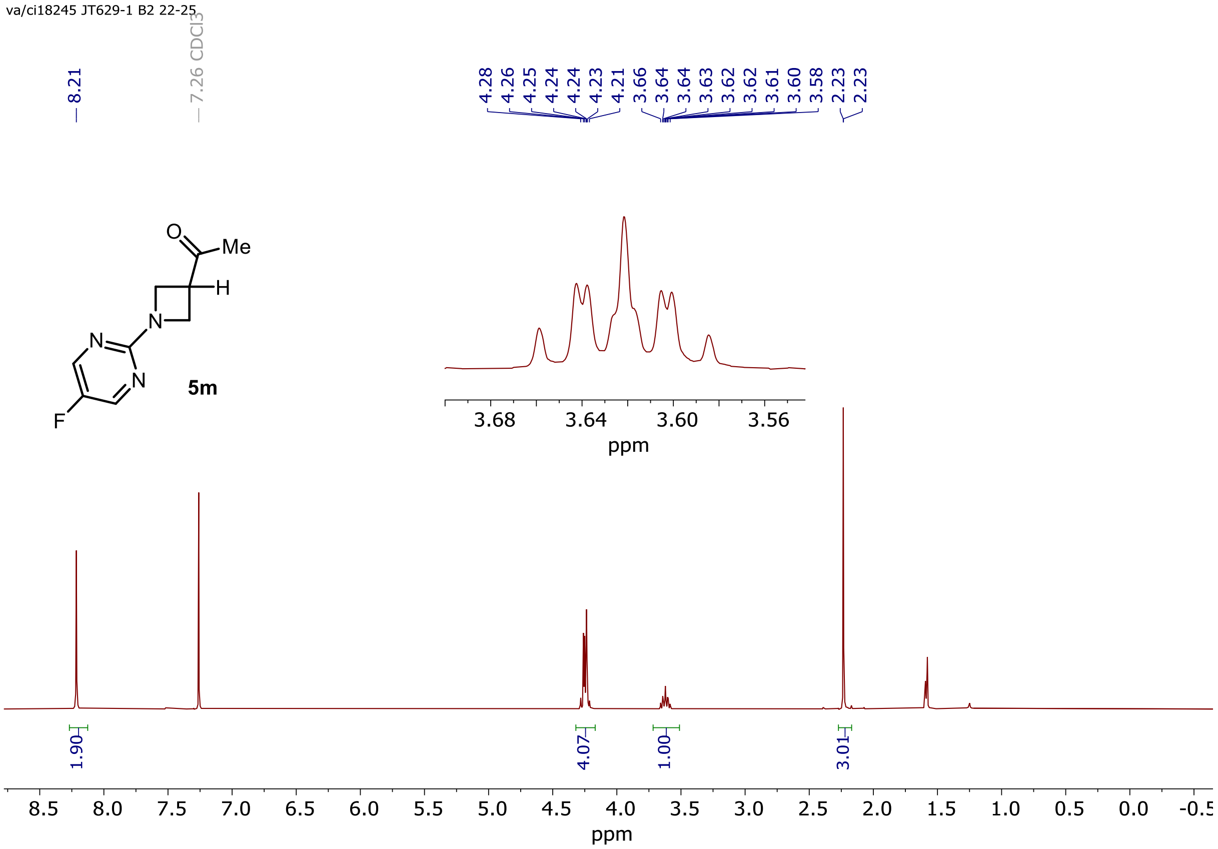

^{13}C NMR (126 MHz, $CDCl_3$) of **5m**

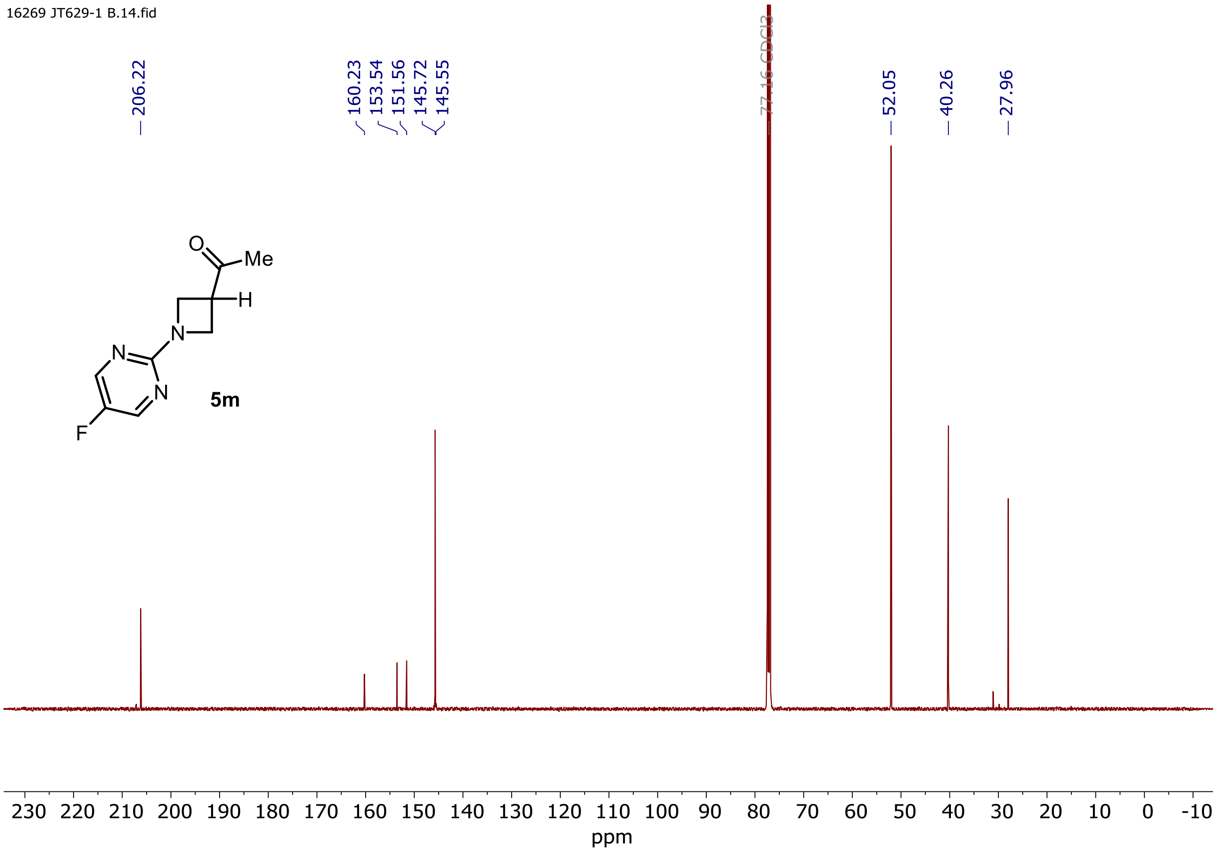

^{19}F NMR (376 MHz, $CDCl_3$) of **5m**

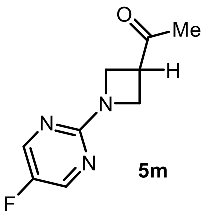

5m

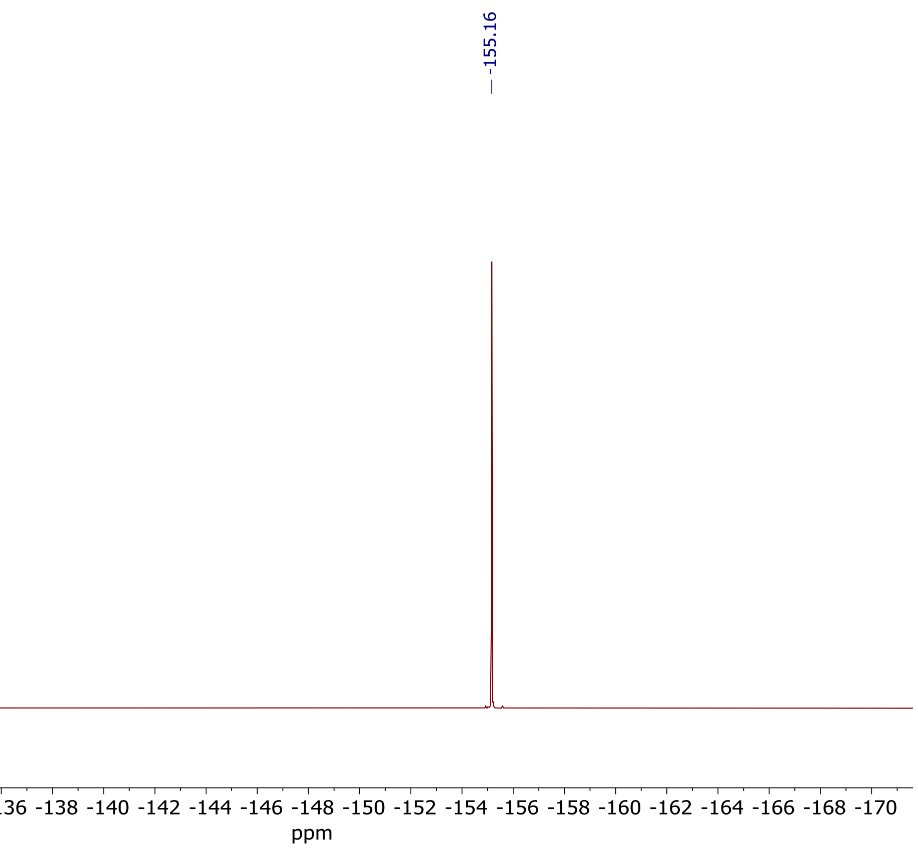

^{1}H NMR (500 MHz, $CDCl_3$) of **5n** (*see procedure*)

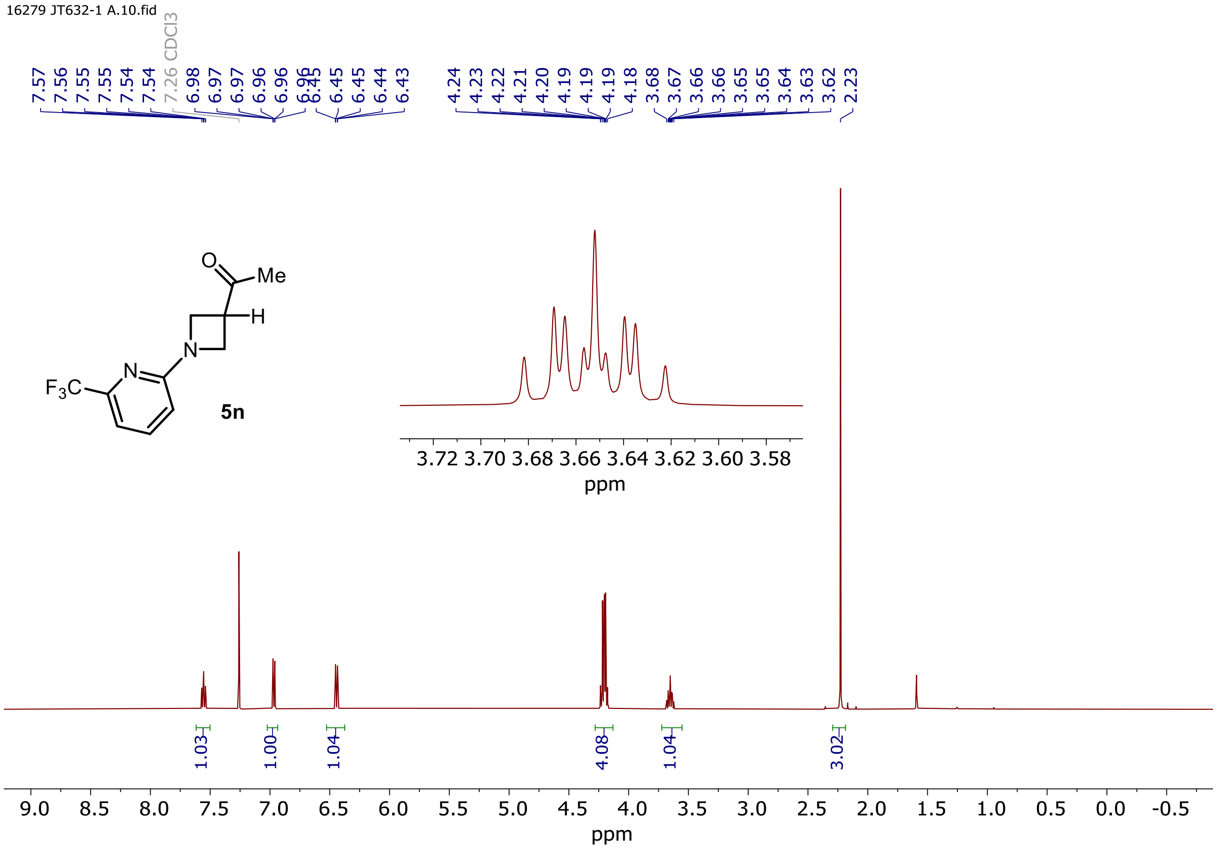

^{13}C NMR (126 MHz, $CDCl_3$) of **5n**

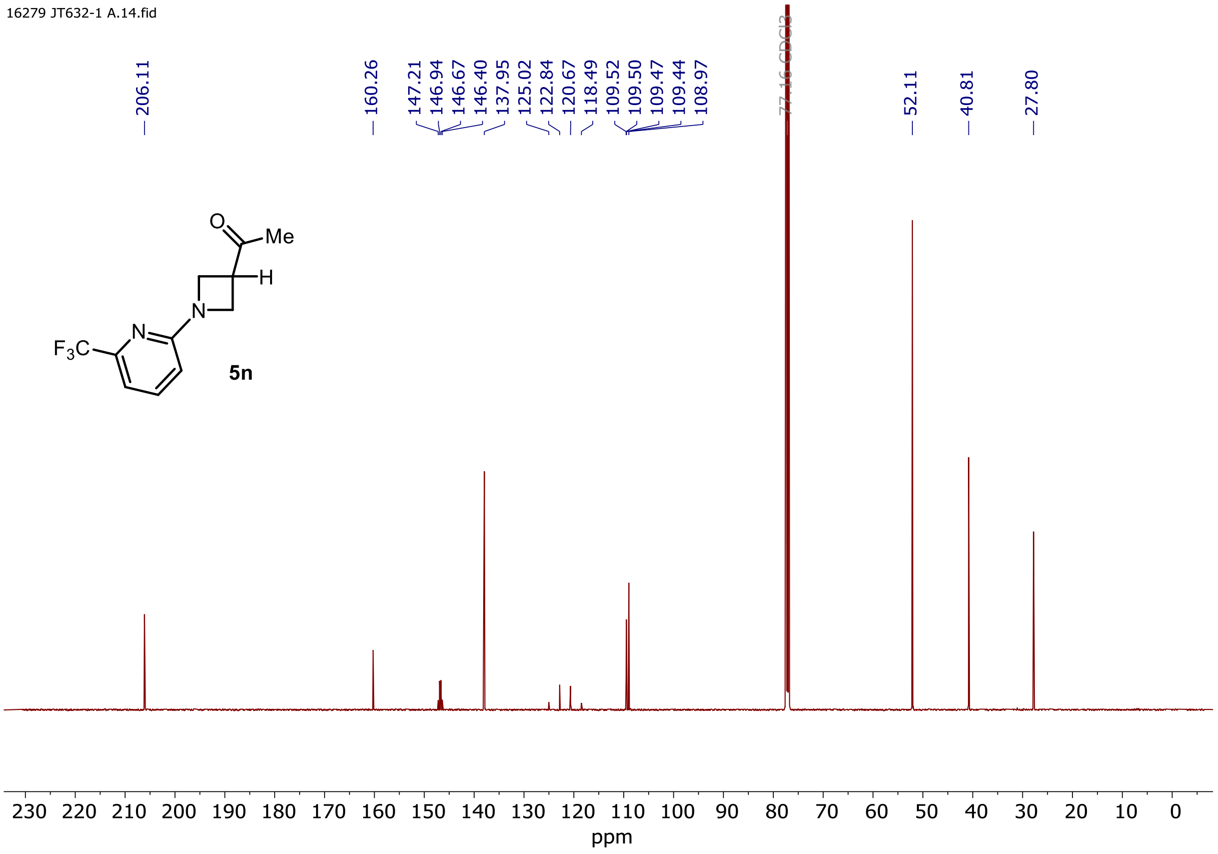

^{19}F NMR (376 MHz, $CDCl_3$) of **5n**

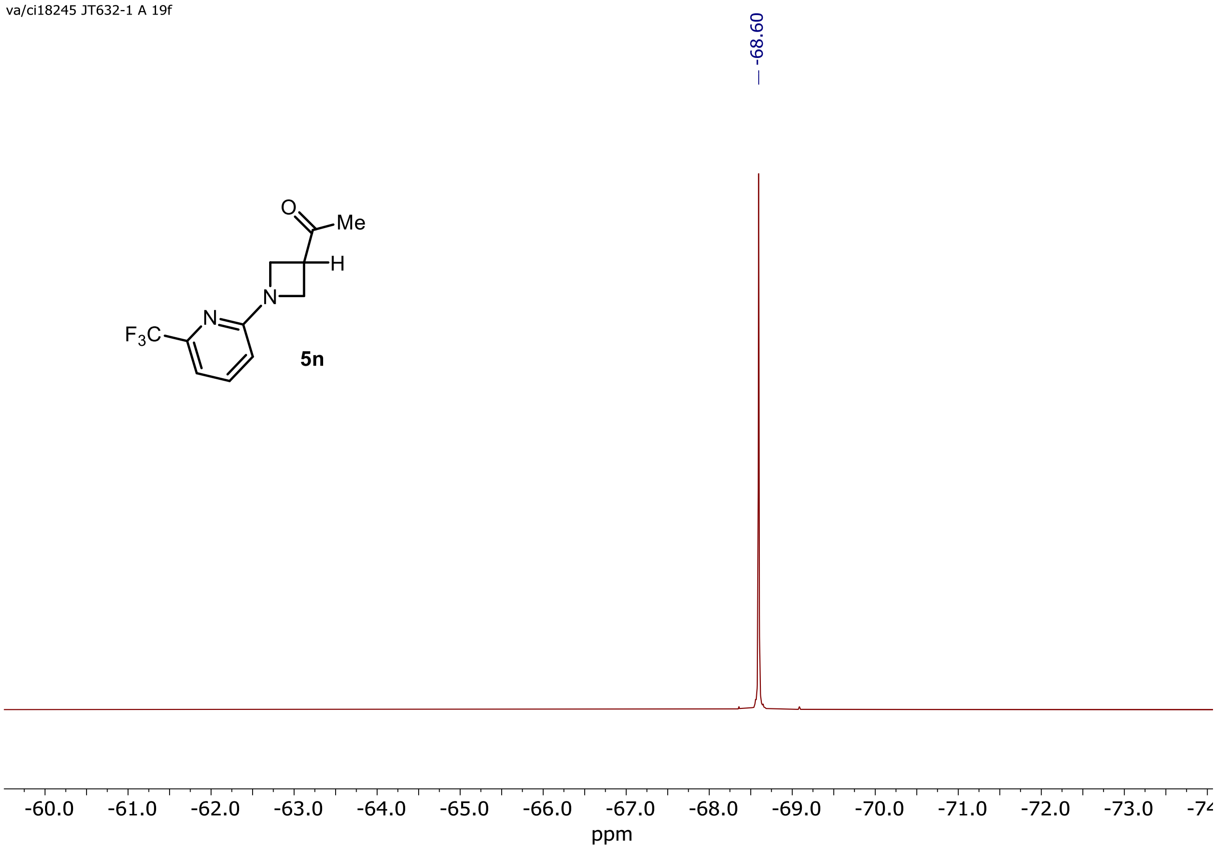

^{1}H NMR (500 MHz, $CDCl_3$) of **5o** (*see procedure*)

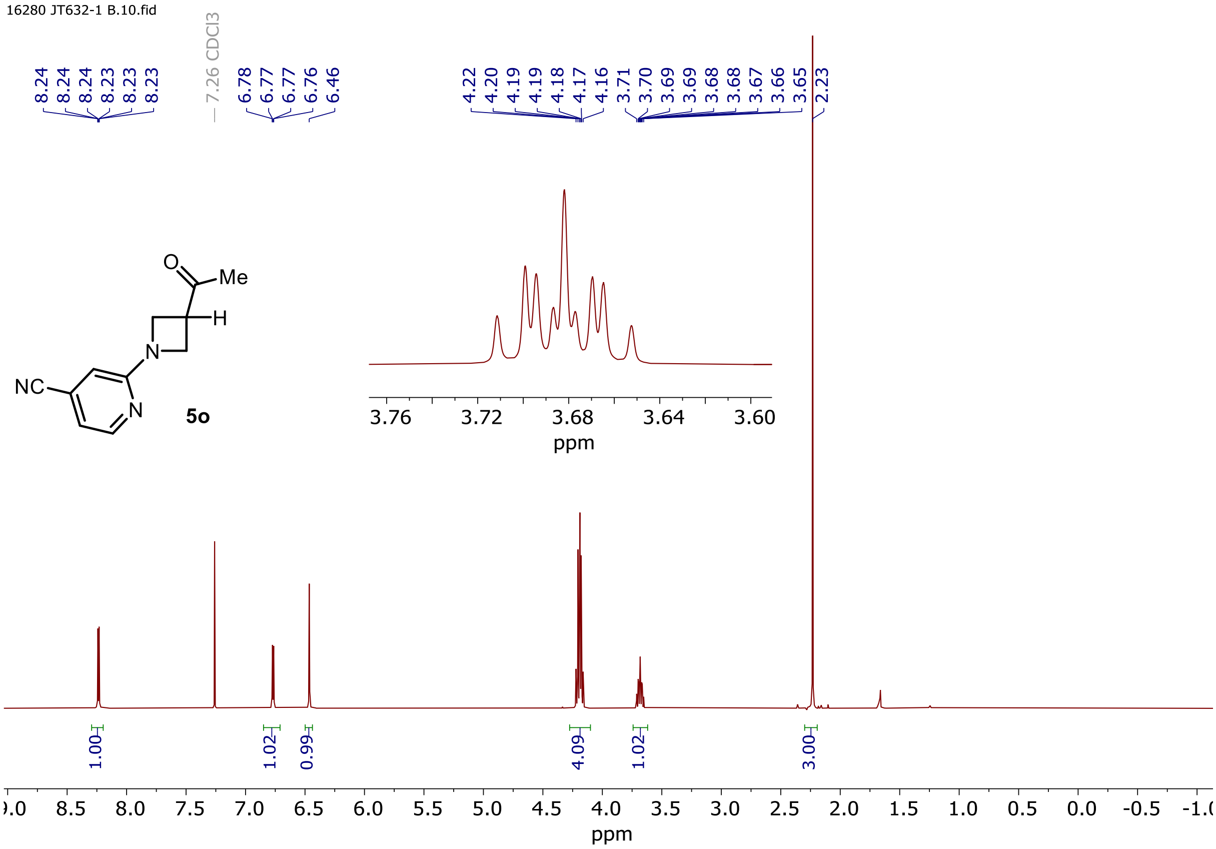

^{13}C NMR (126 MHz, $CDCl_3$) of **5o**

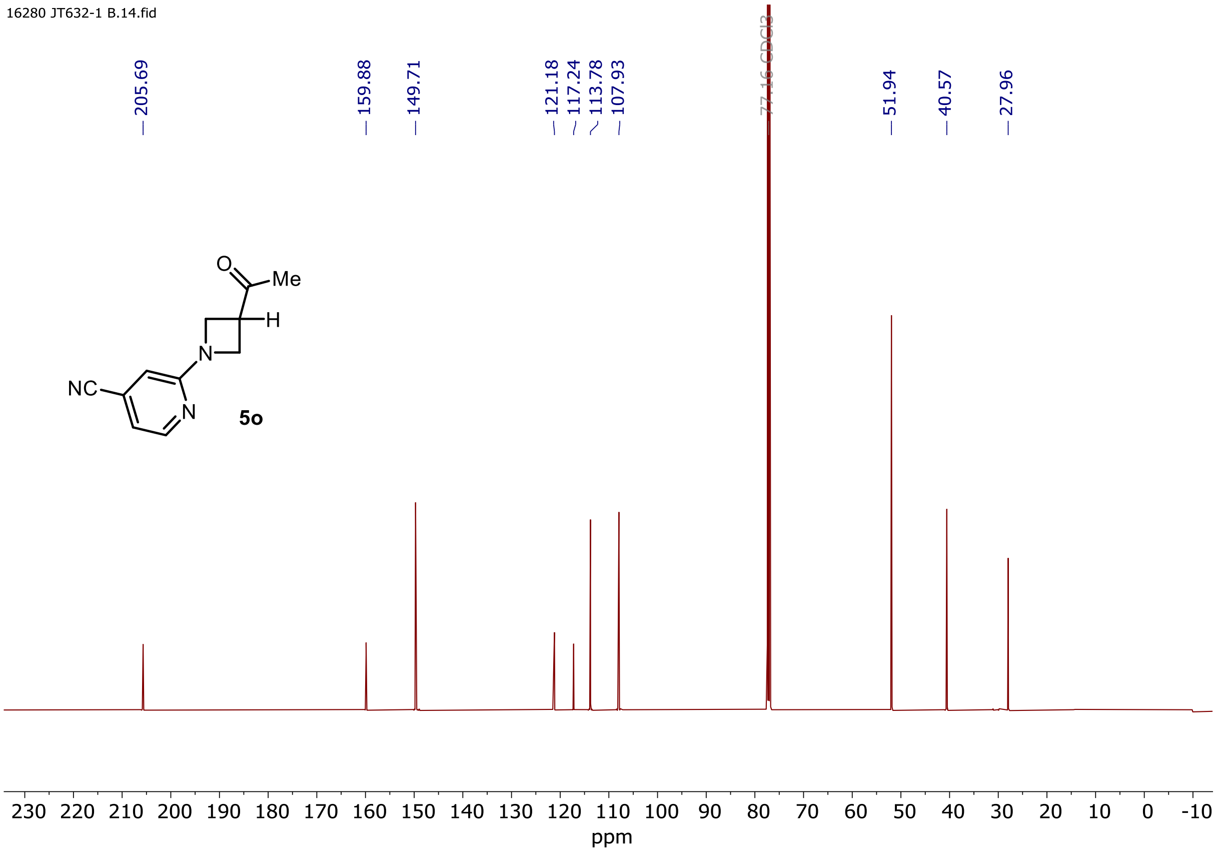

^{1}H NMR (500 MHz, $CDCl_3$) of **8a** (*see procedure*)

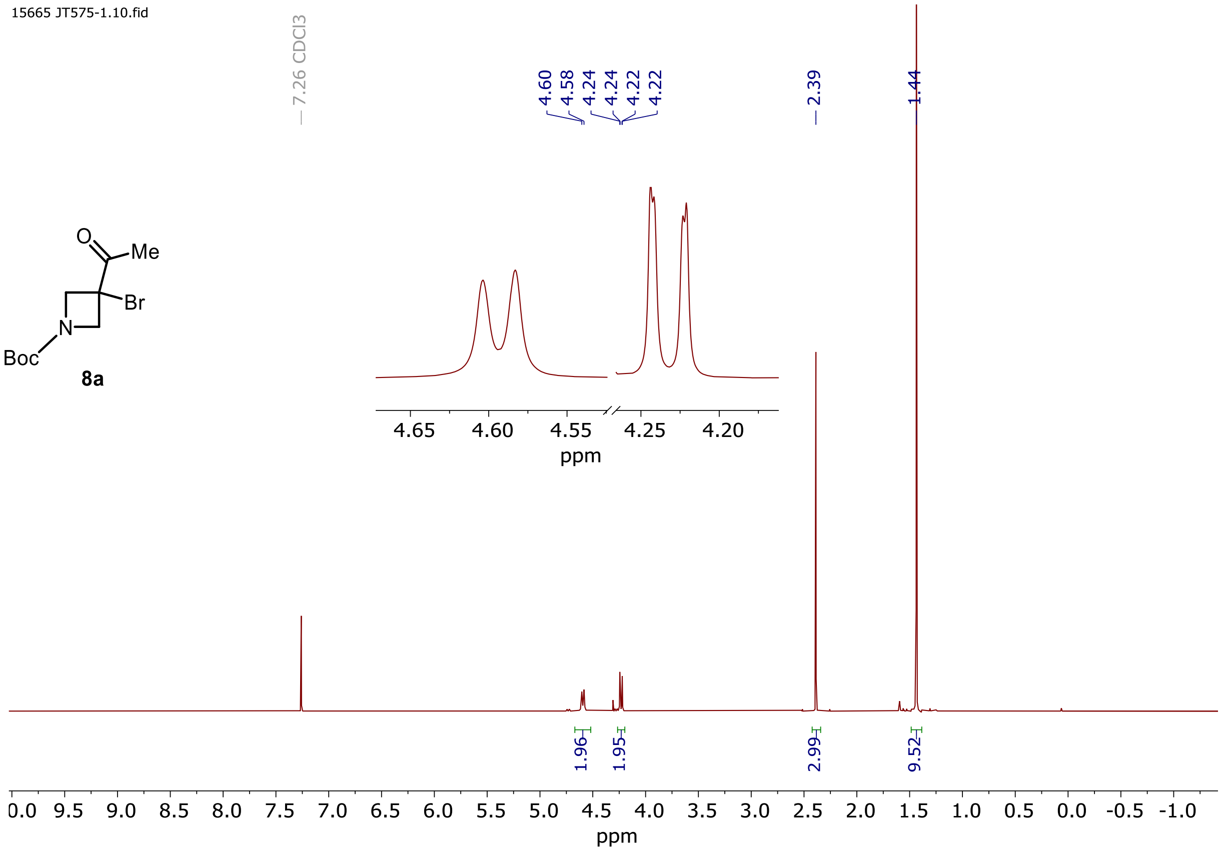

^{13}C NMR (126 MHz, $CDCl_3$) of **8a**

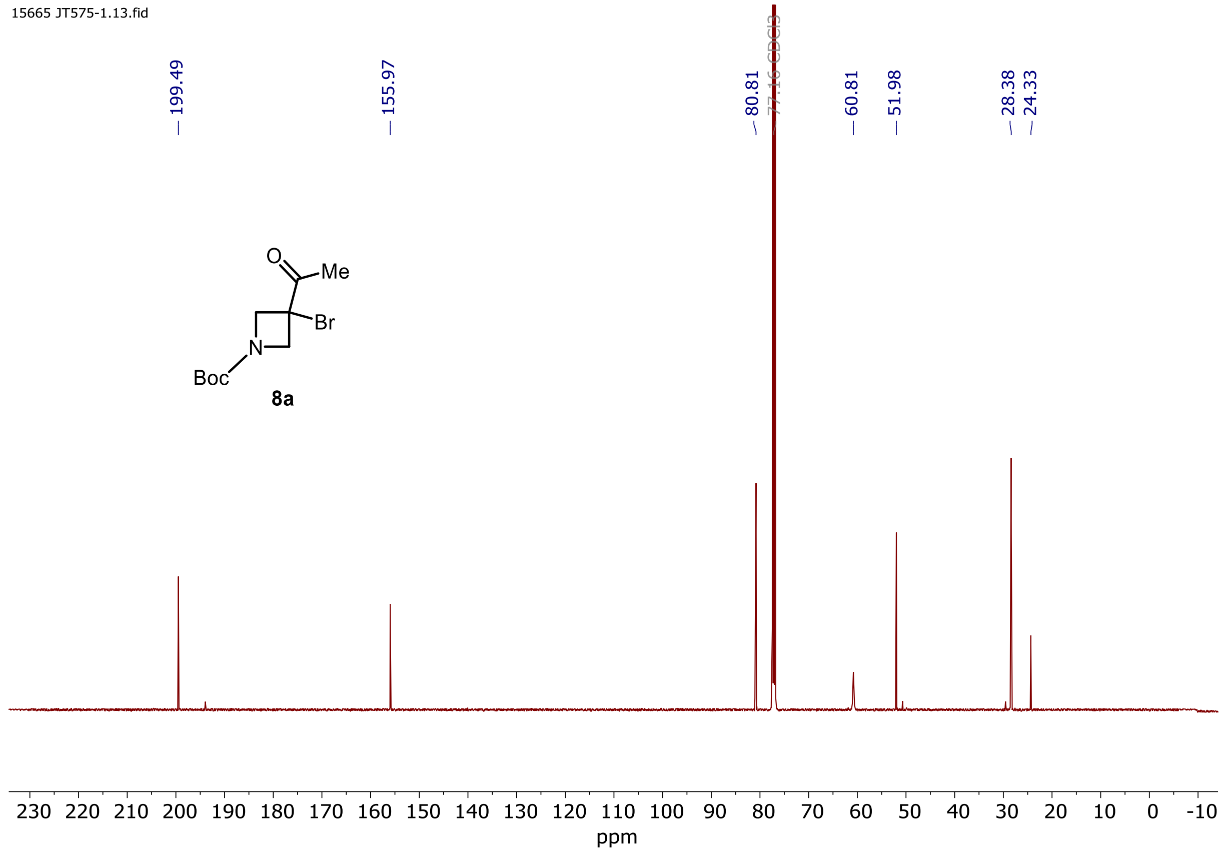

^{1}H NMR (500 MHz, $CDCl_3$) of **8b** (*see procedure*)

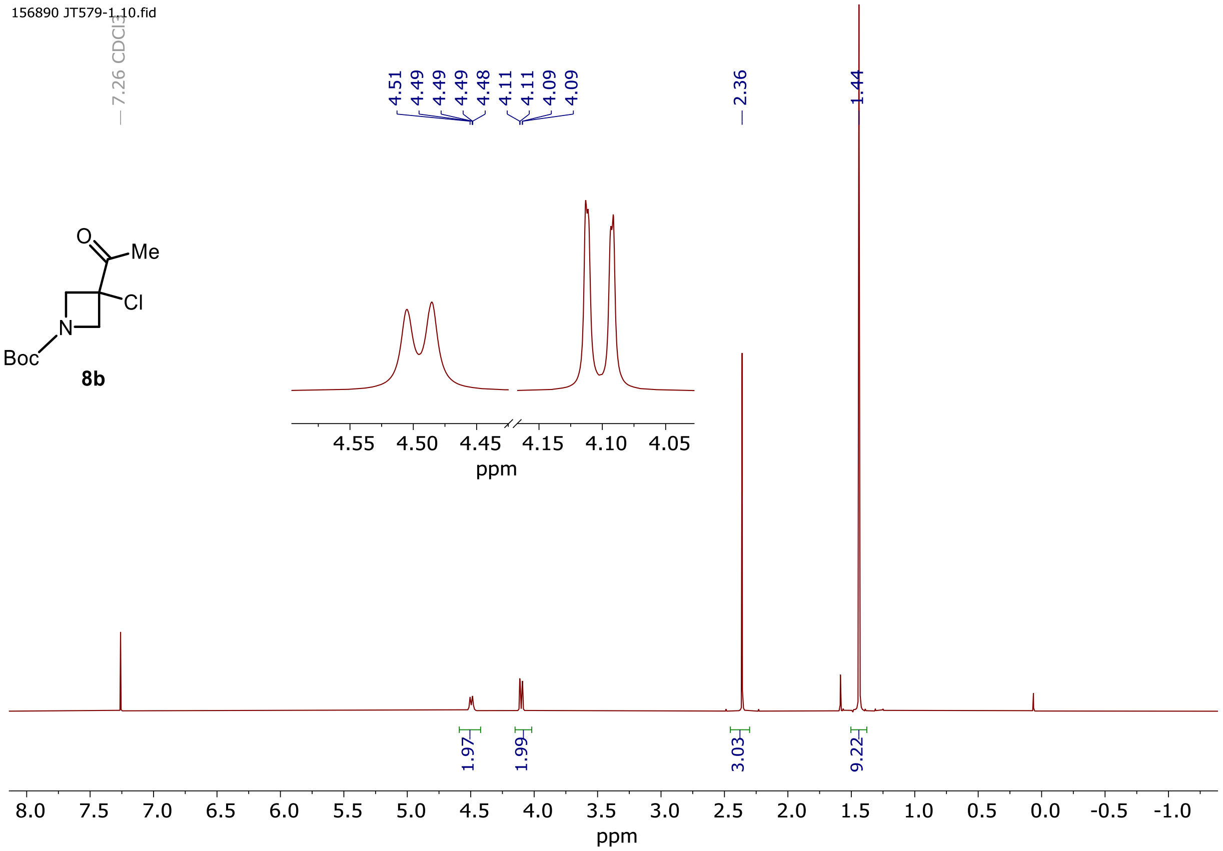

^{13}C NMR (126 MHz, $CDCl_3$) of **8b**

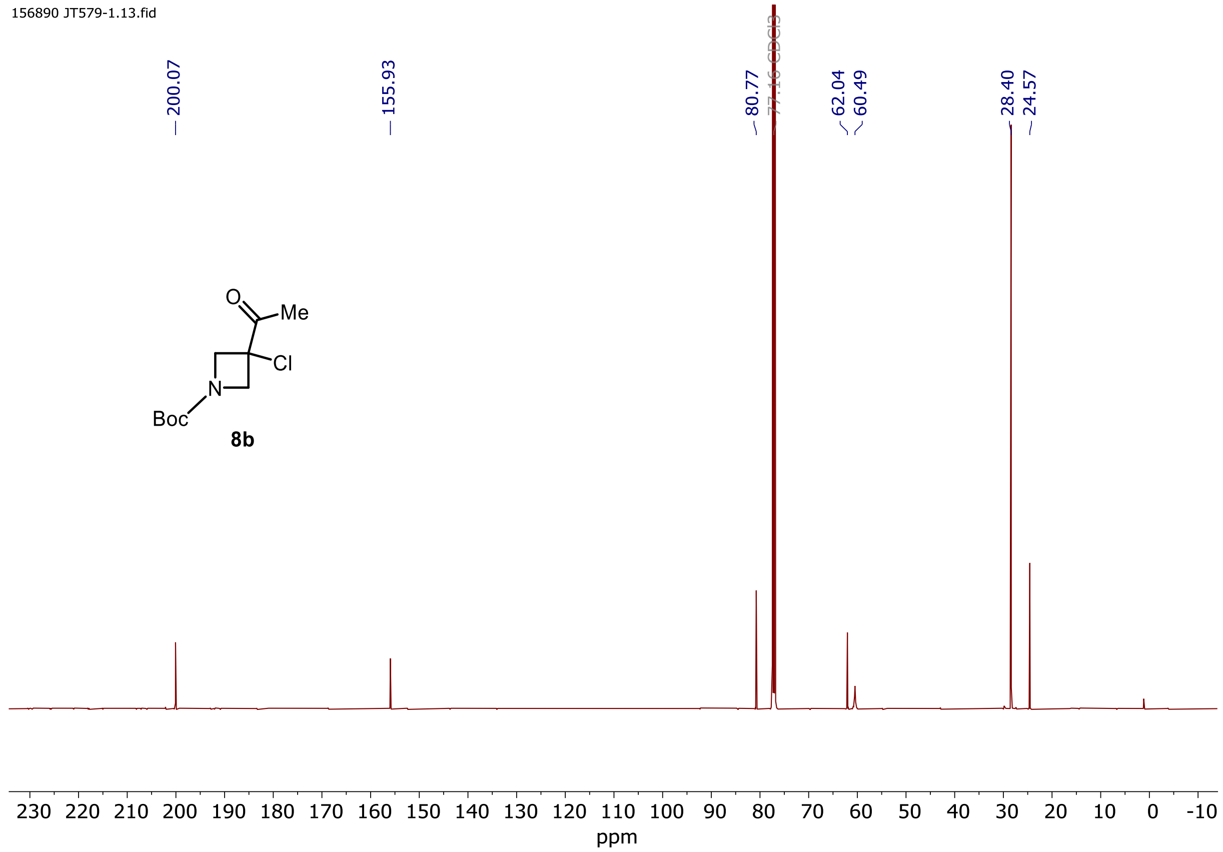

^{1}H NMR (500 MHz, $CDCl_3$) of **8c** (*see procedure*)

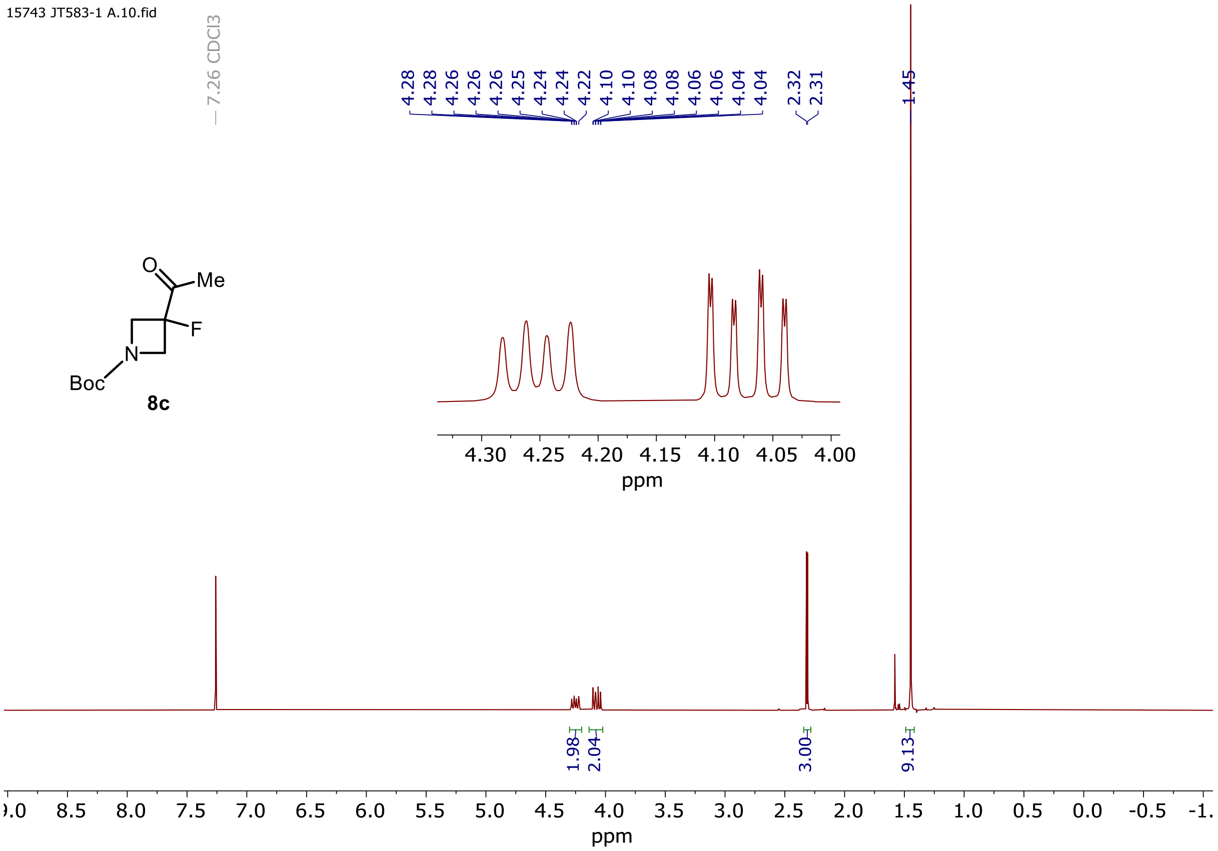

^{13}C NMR (126 MHz, $CDCl_3$) of **8c**

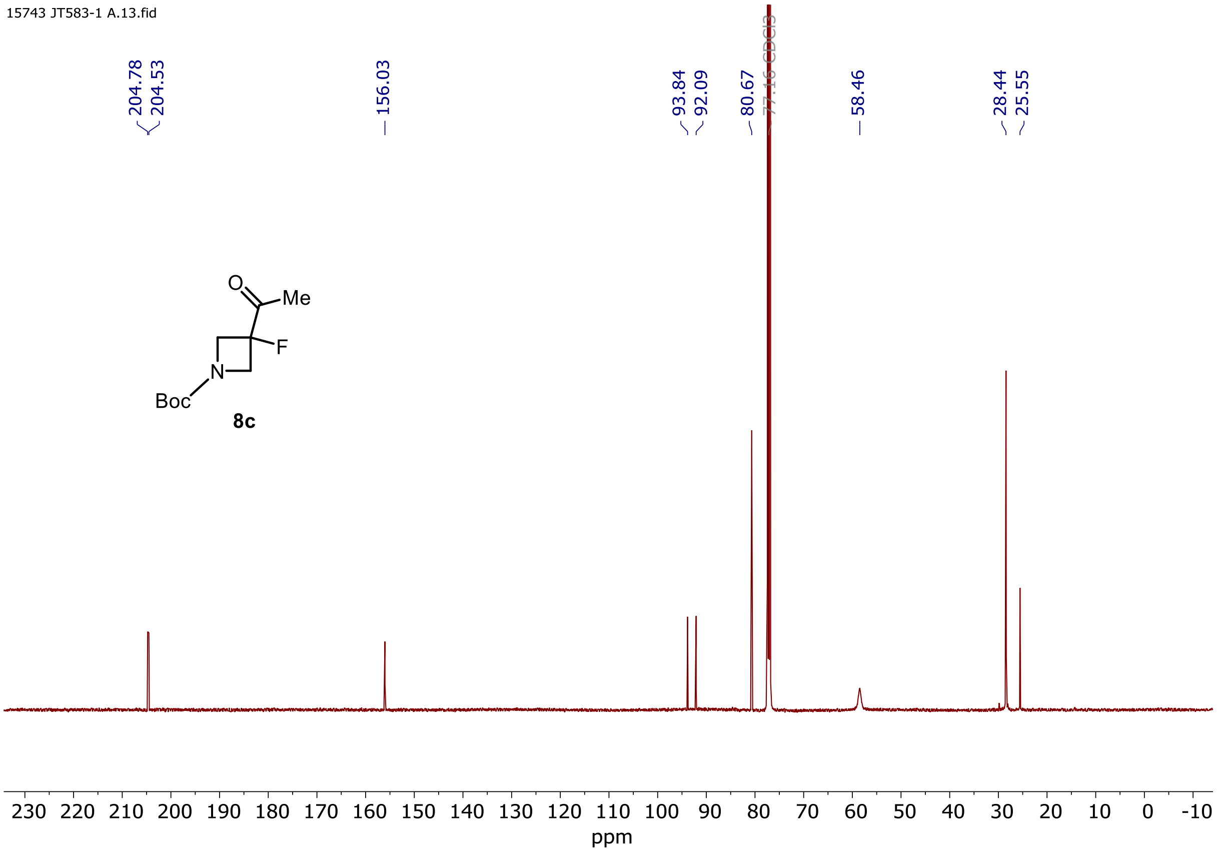

^{19}F NMR (376 MHz, $CDCl_3$) of **8c**

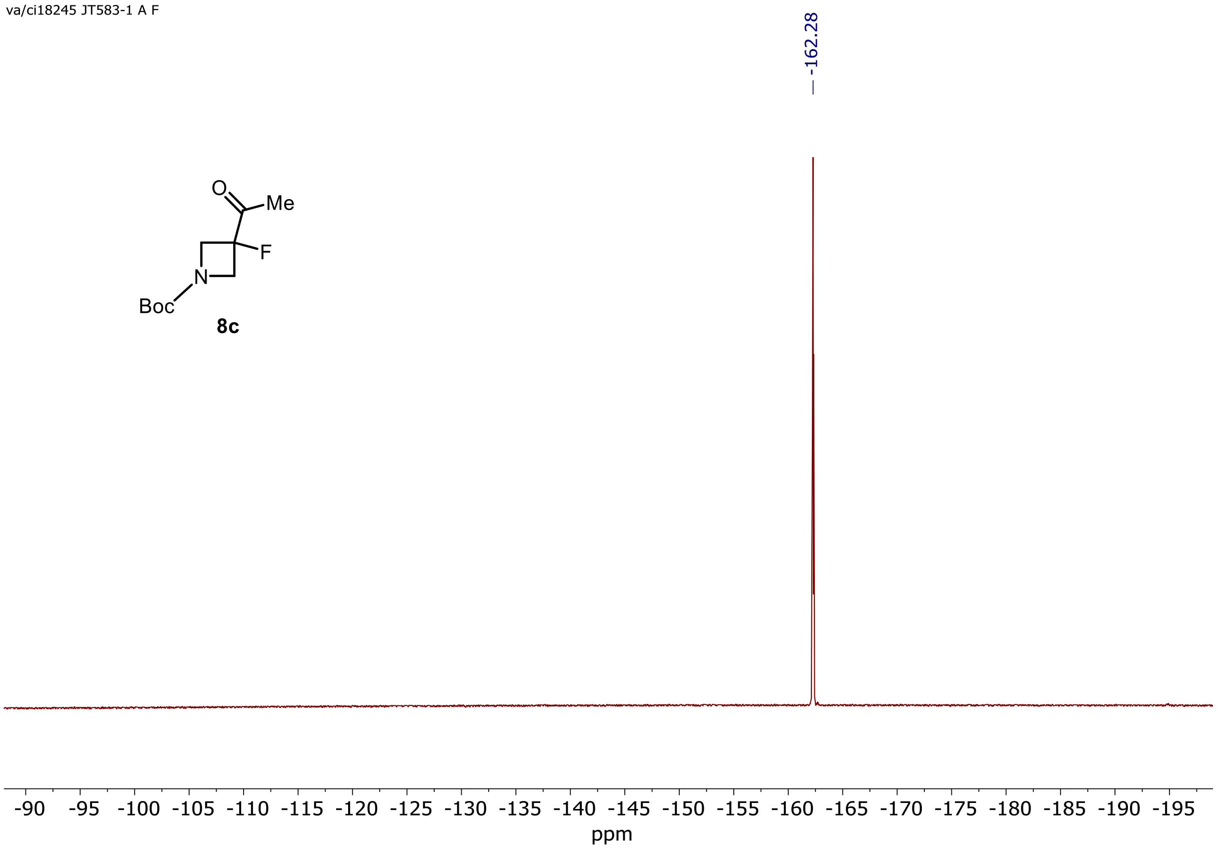

^{1}H NMR (500 MHz, $CDCl_3$) of **8d** (*see procedure*)

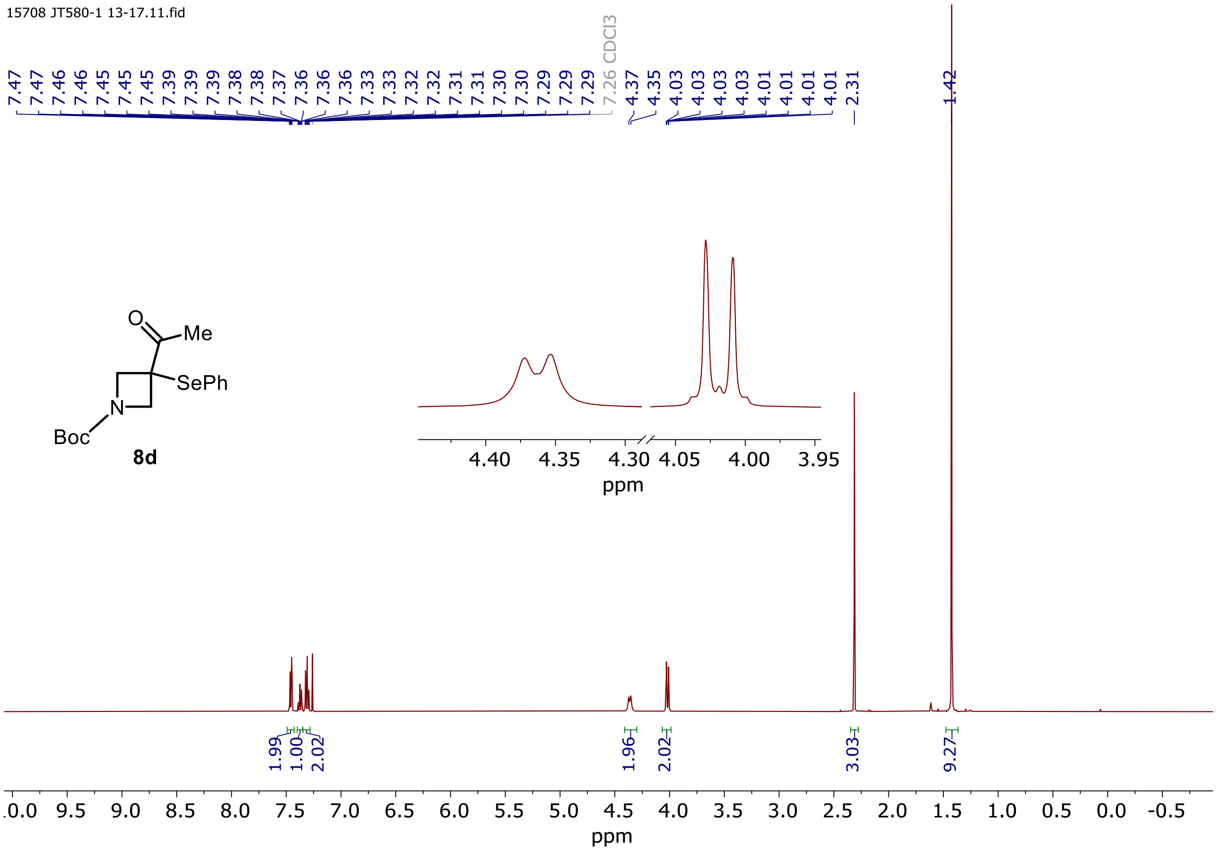

^{13}C NMR (126 MHz, $CDCl_3$) of **8d**

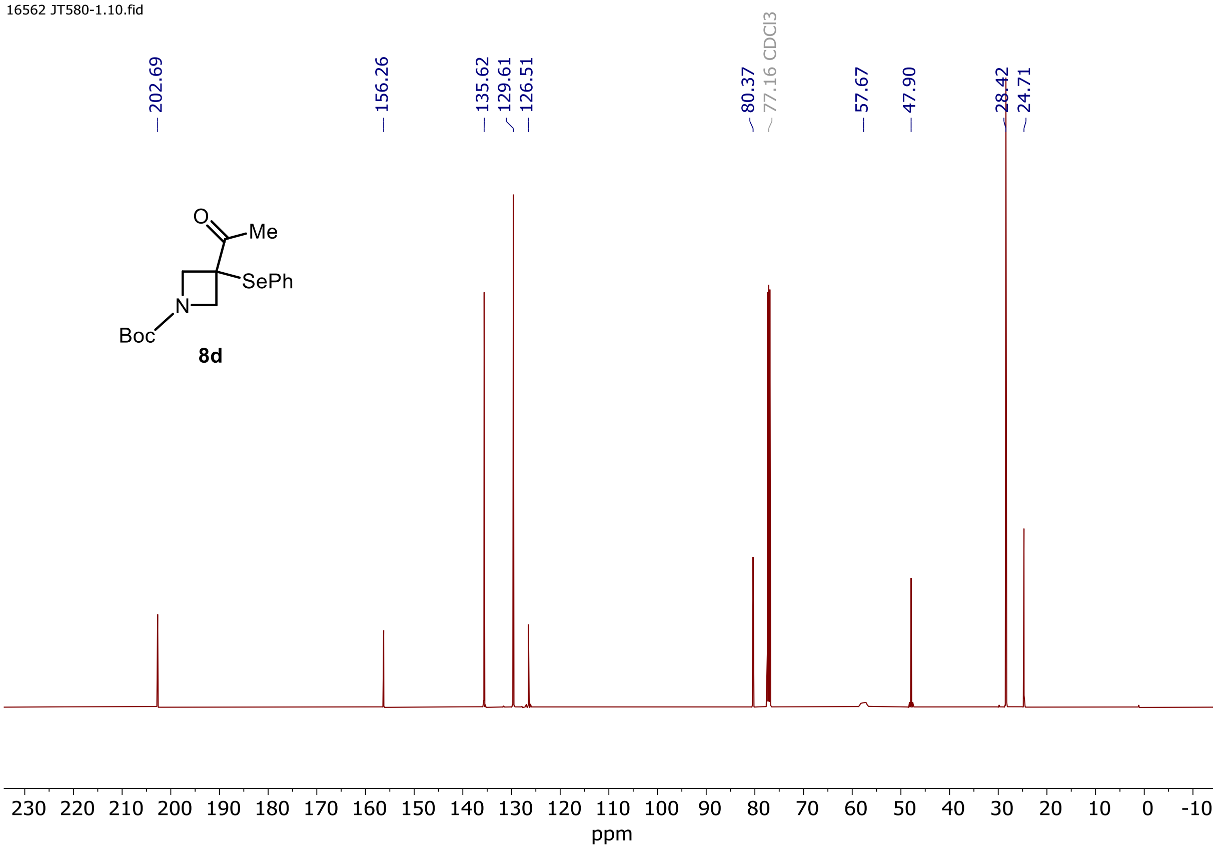

^{1}H NMR (500 MHz, $CDCl_3$) of **8e** (*see procedure*)

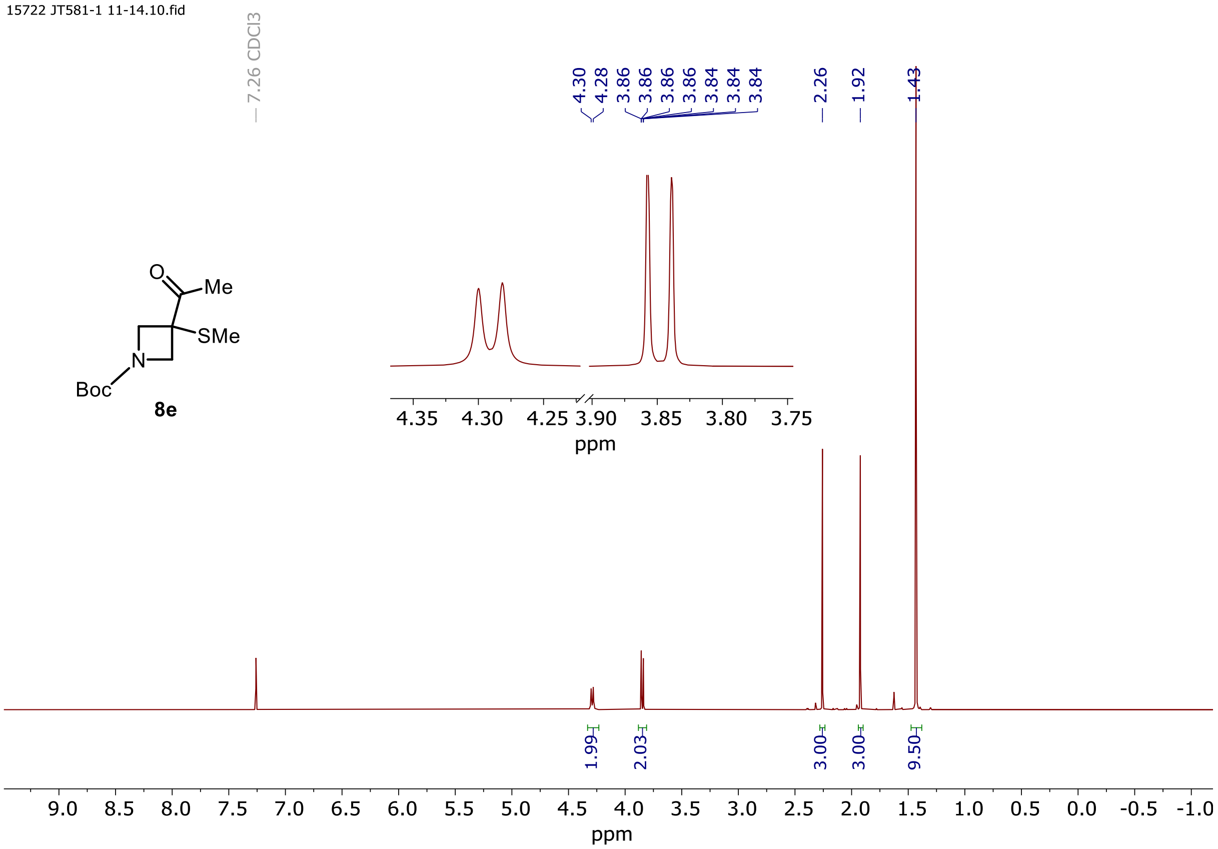

^{13}C NMR (126 MHz, $CDCl_3$) of **8e**

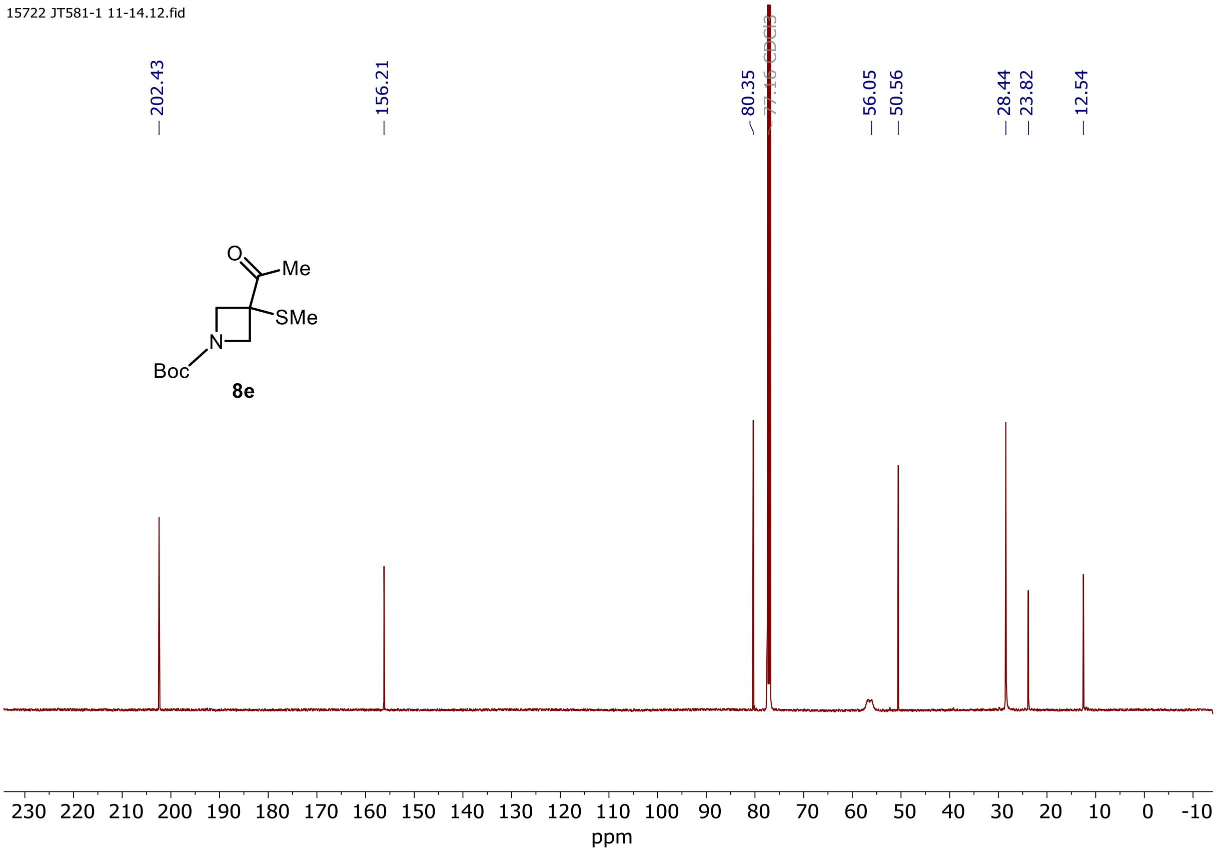

^{1}H NMR (500 MHz, $CDCl_3$) of **8f** (*see procedure*)

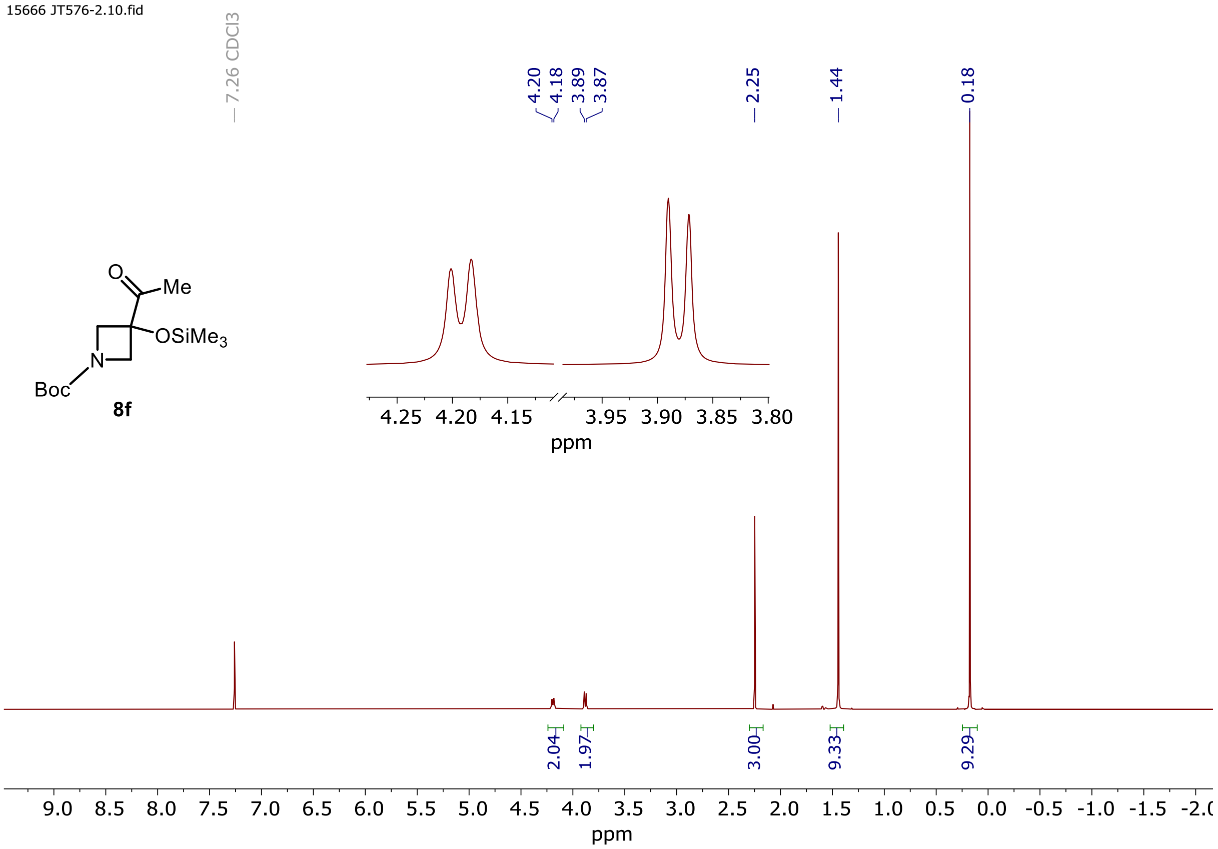

^{13}C NMR (126 MHz, $CDCl_3$) of **8f**

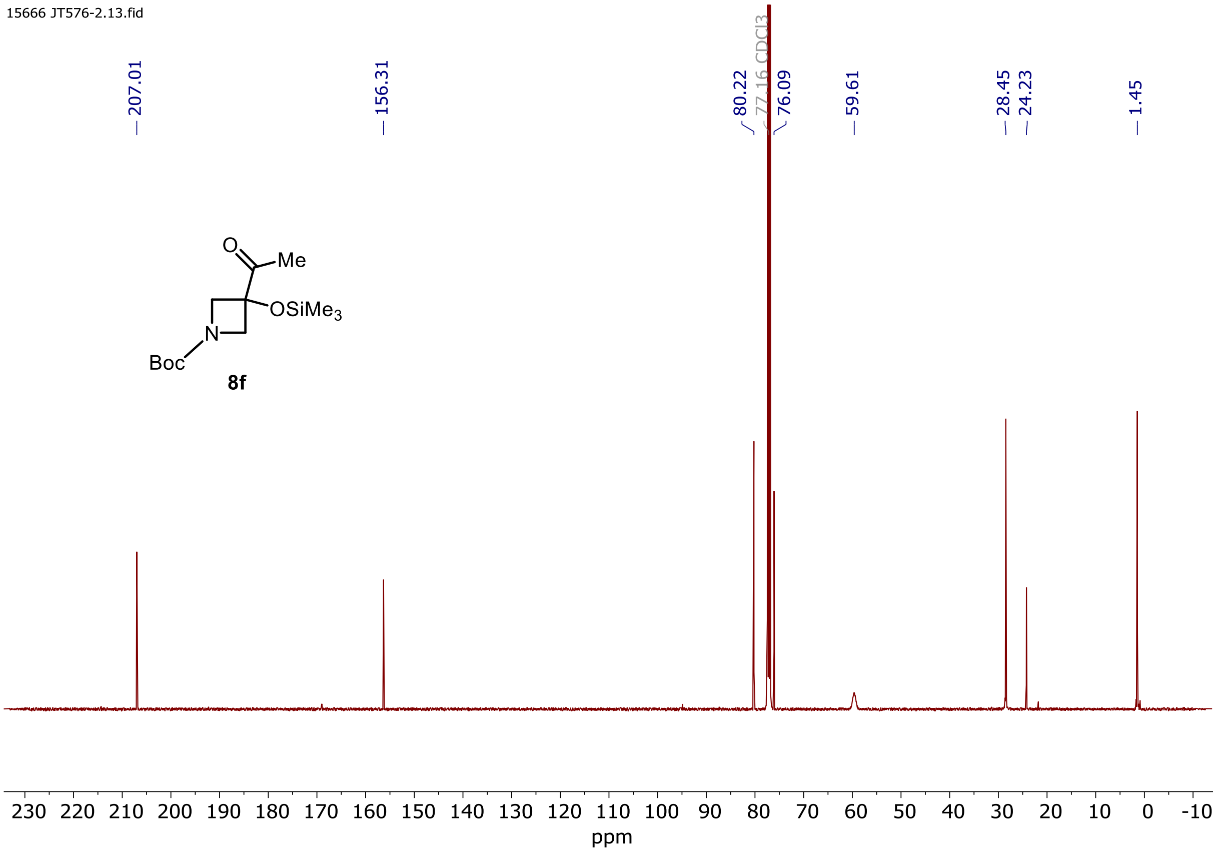

^{1}H NMR (400 MHz, $CDCl_3$) of **8g** (see procedure)

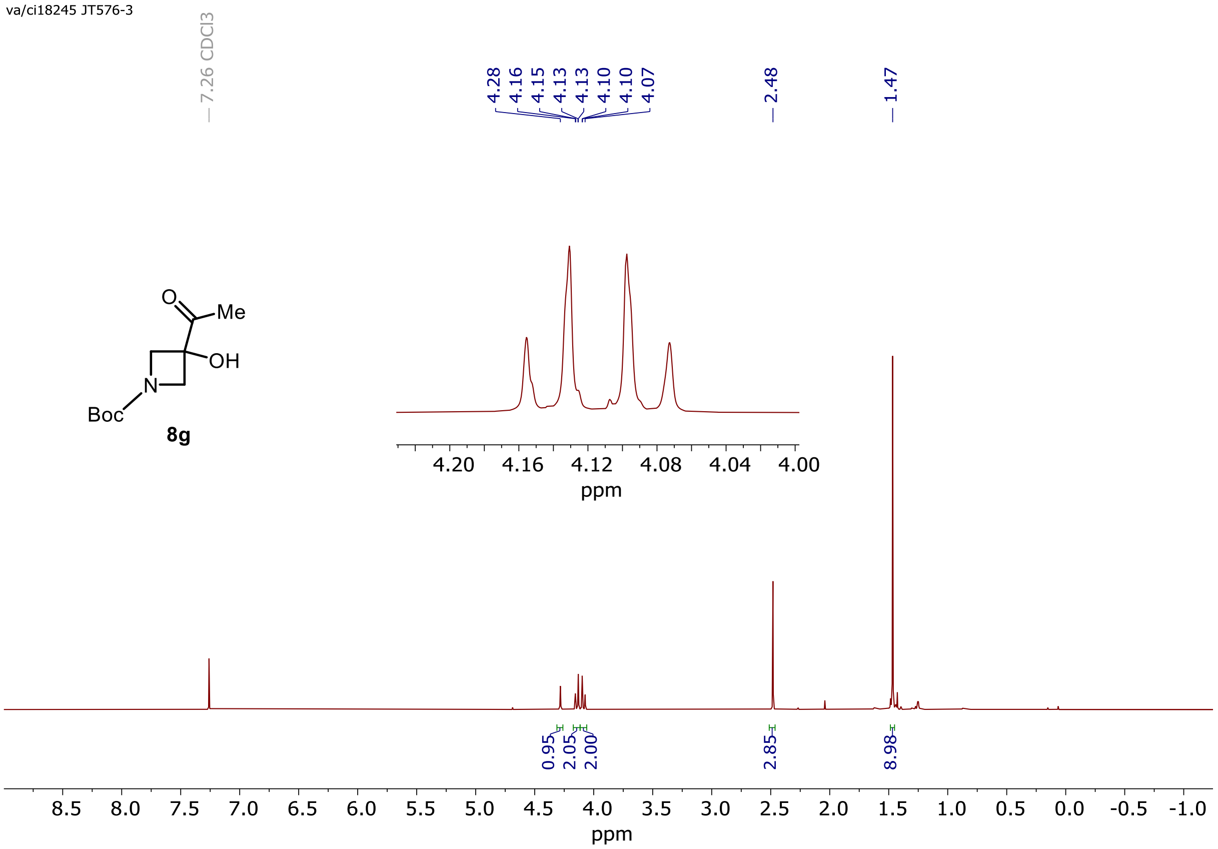

^{13}C NMR (126 MHz, $CDCl_3$) of **8g**

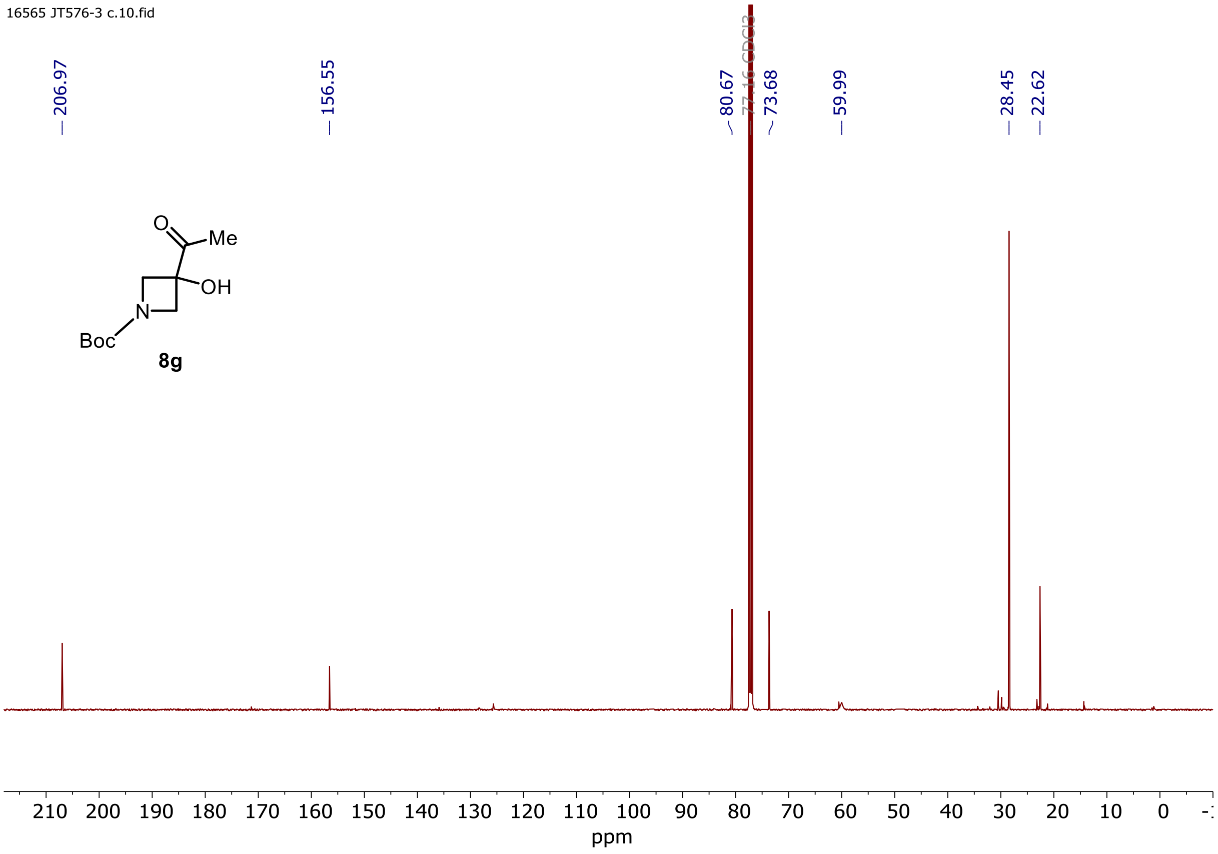

^{1}H NMR (400 MHz, $CDCl_3$) of **8h** (*see procedure*)

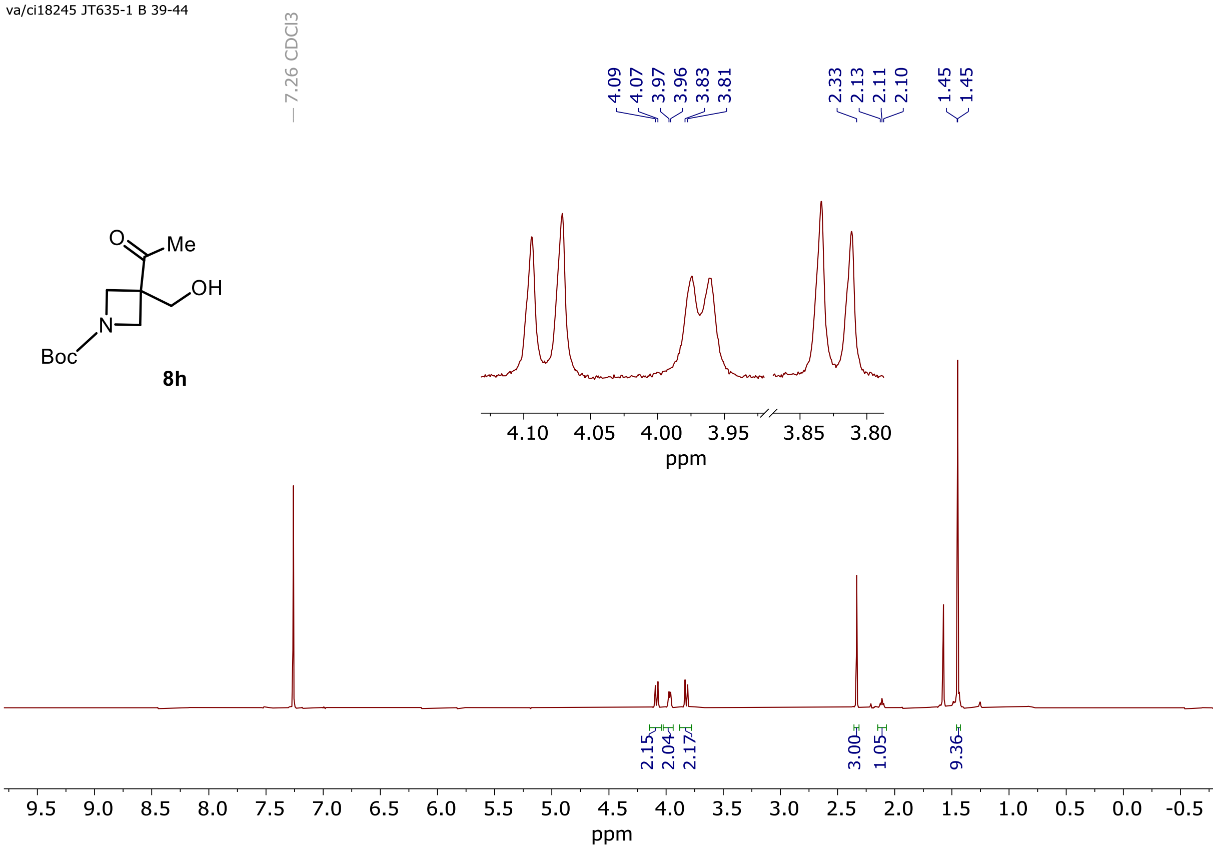

^{13}C NMR (126 MHz, $CDCl_3$) of **8h**

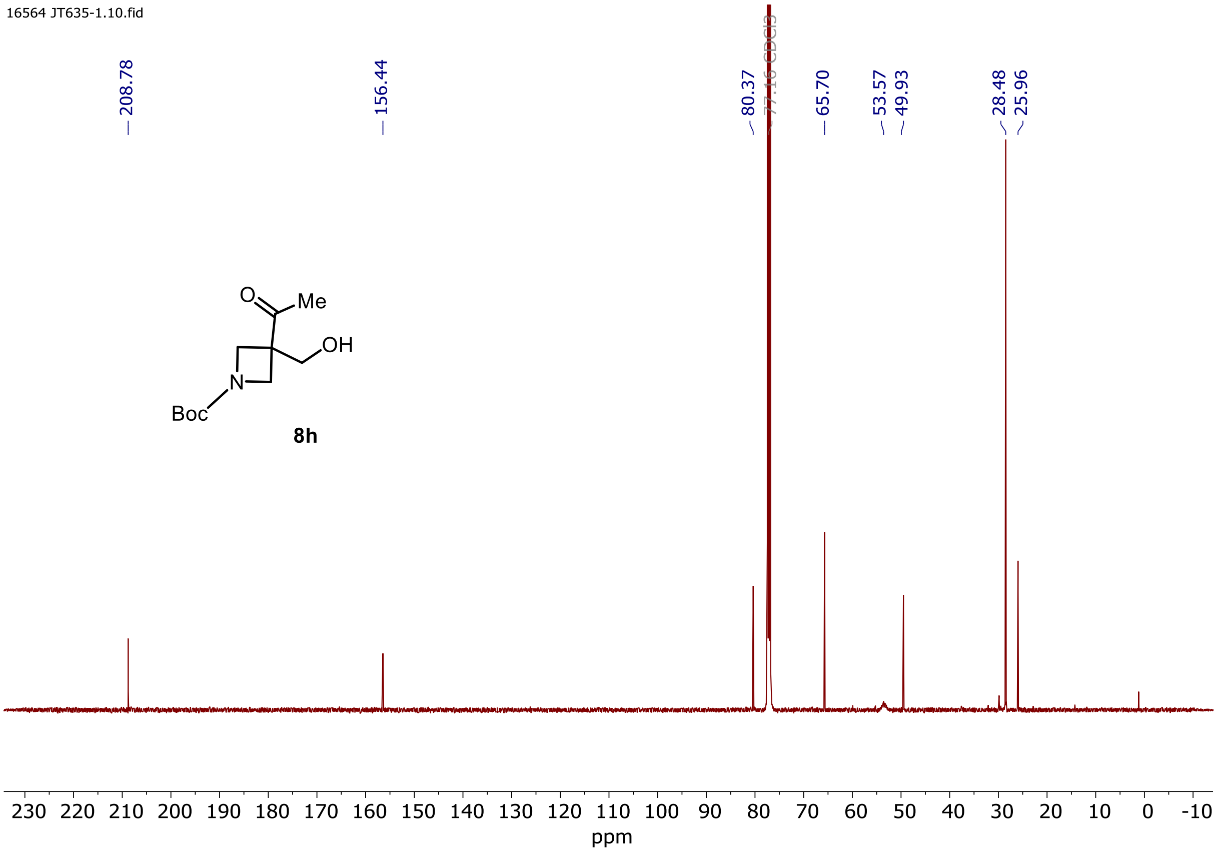

^{1}H NMR (500 MHz, $CDCl_3$) of **8i** (*see procedure*)

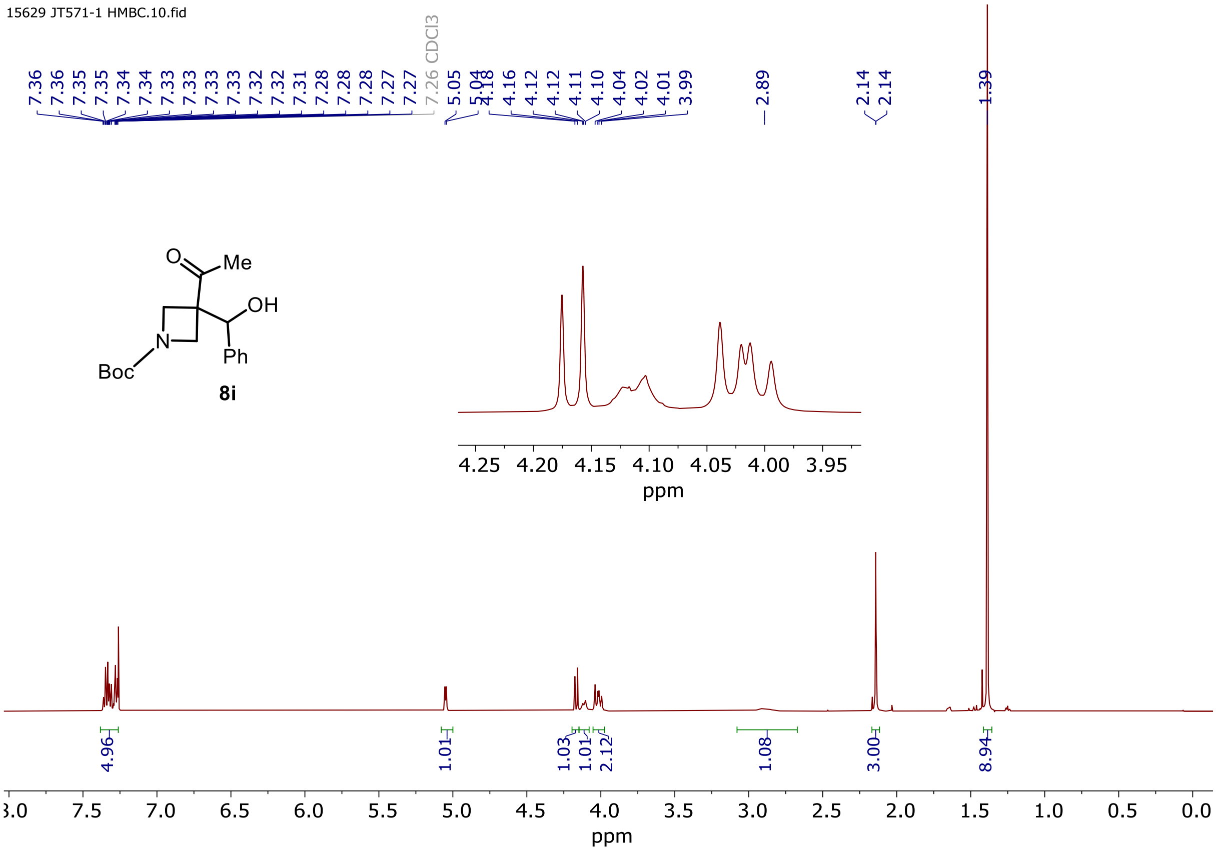

^{13}C NMR (126 MHz, $CDCl_3$) of **8i**

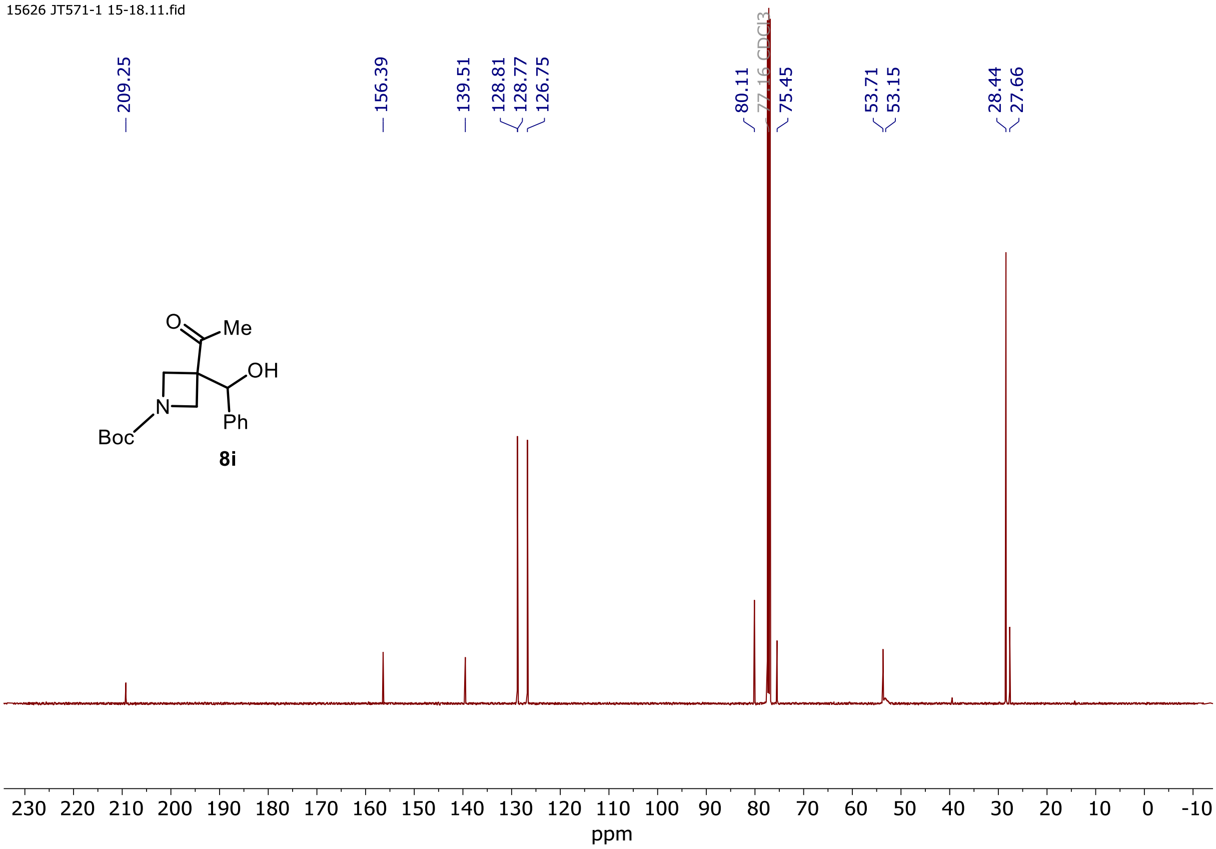

^{1}H NMR (500 MHz, $CDCl_3$) of **8j** (*see procedure*)

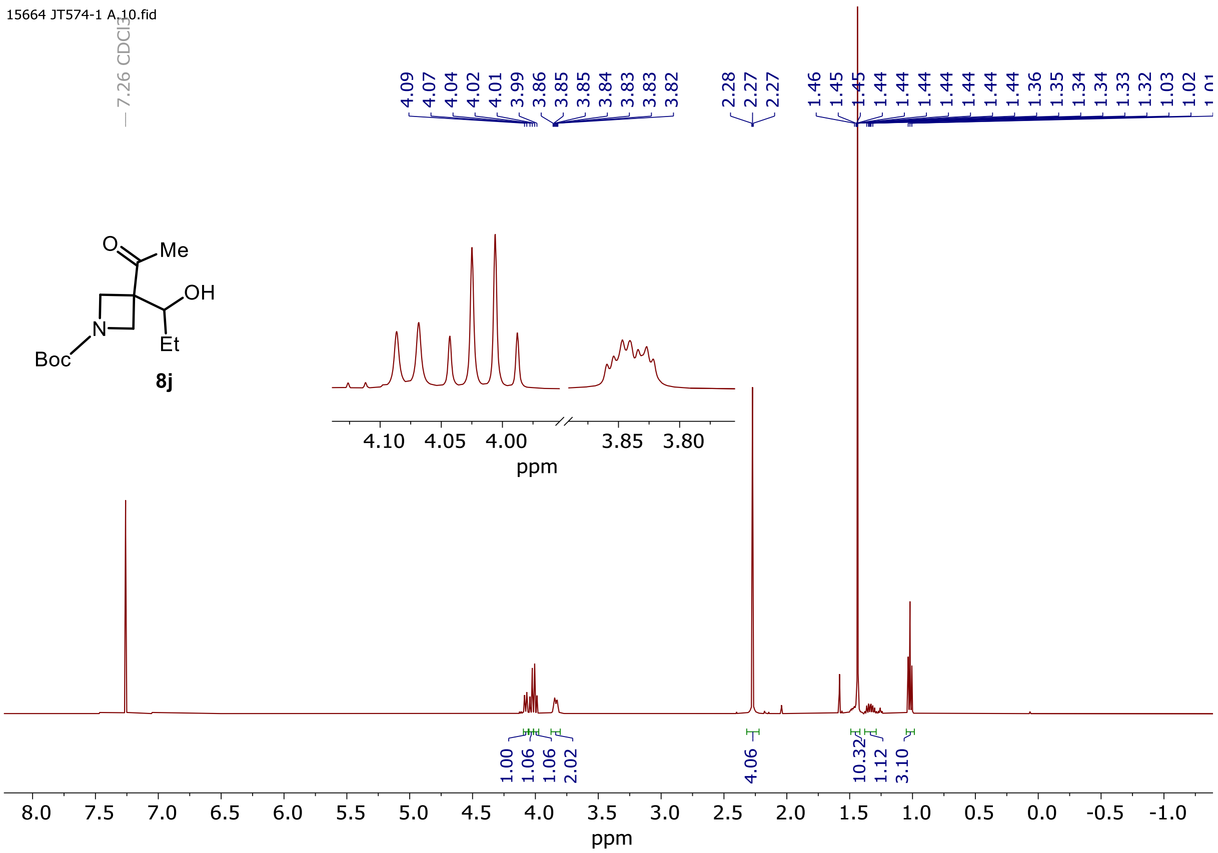

^{13}C NMR (126 MHz, $CDCl_3$) of **8j**

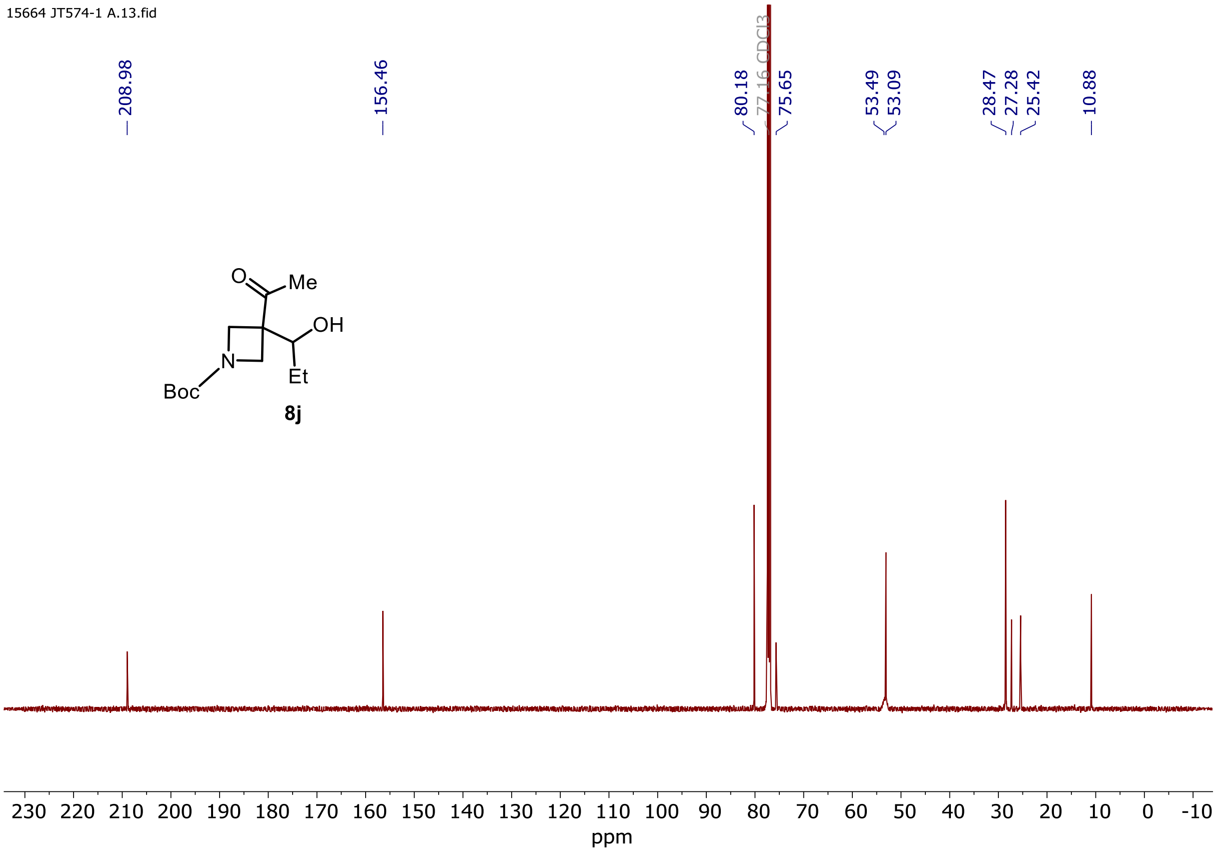

^{1}H NMR (500 MHz, $CDCl_3$) of **8k** (*see procedure*)

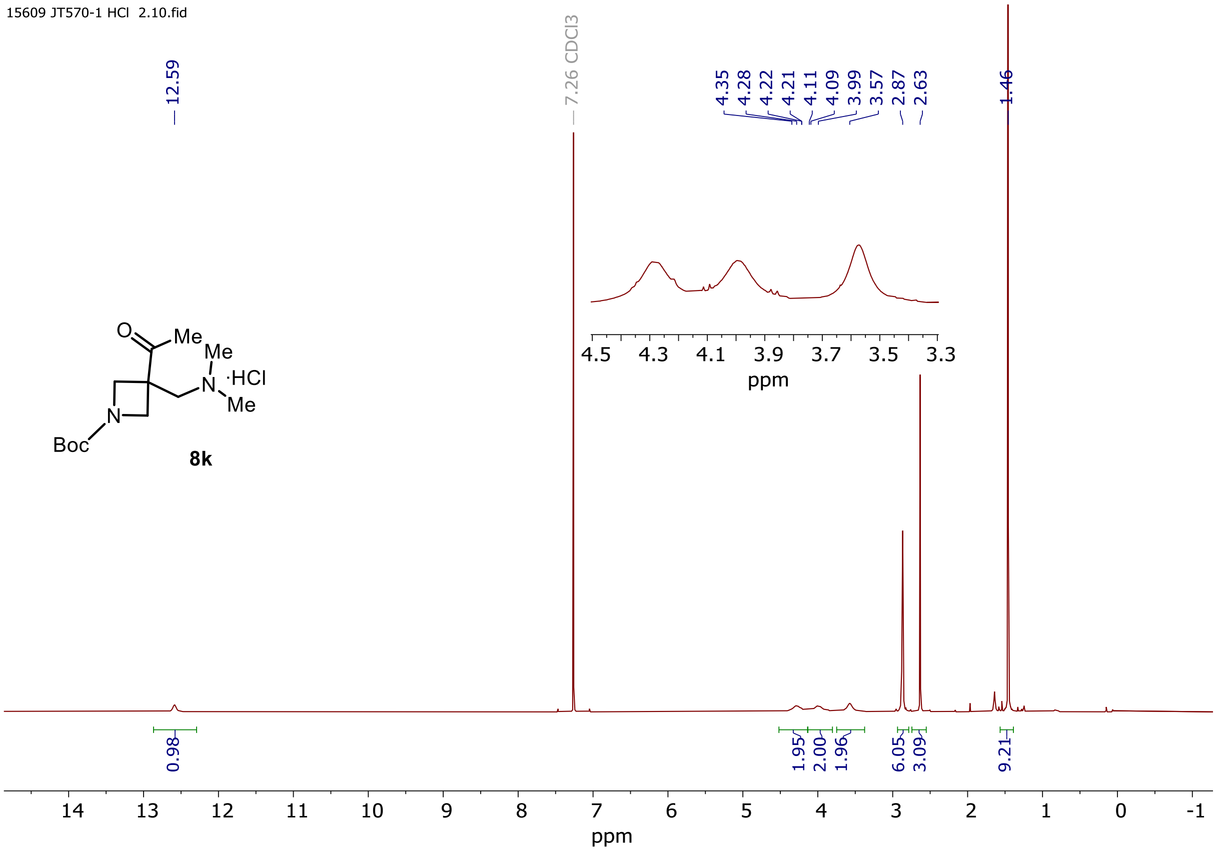

^{13}C NMR (126 MHz, $CDCl_3$) of **8k**

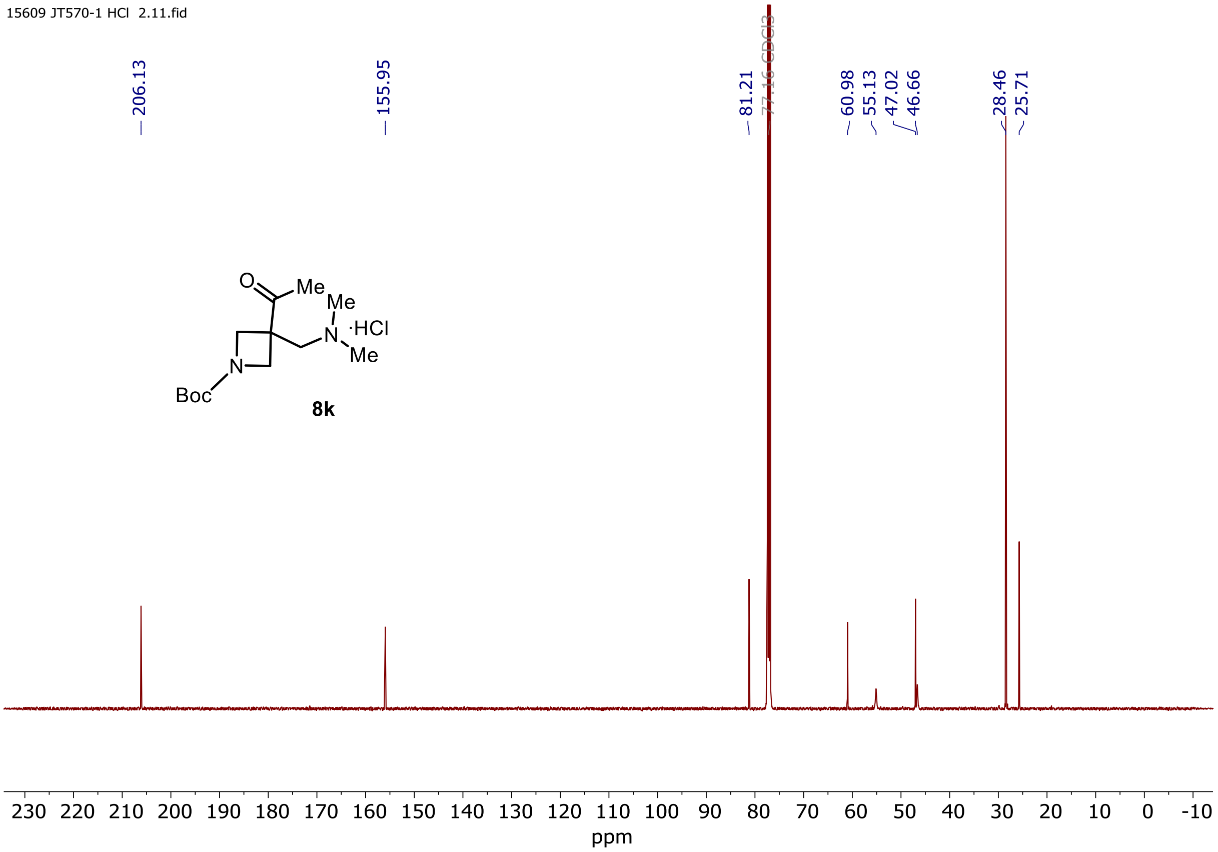

^{1}H NMR (500 MHz, $CDCl_3$) of **8l** (*see procedure*)

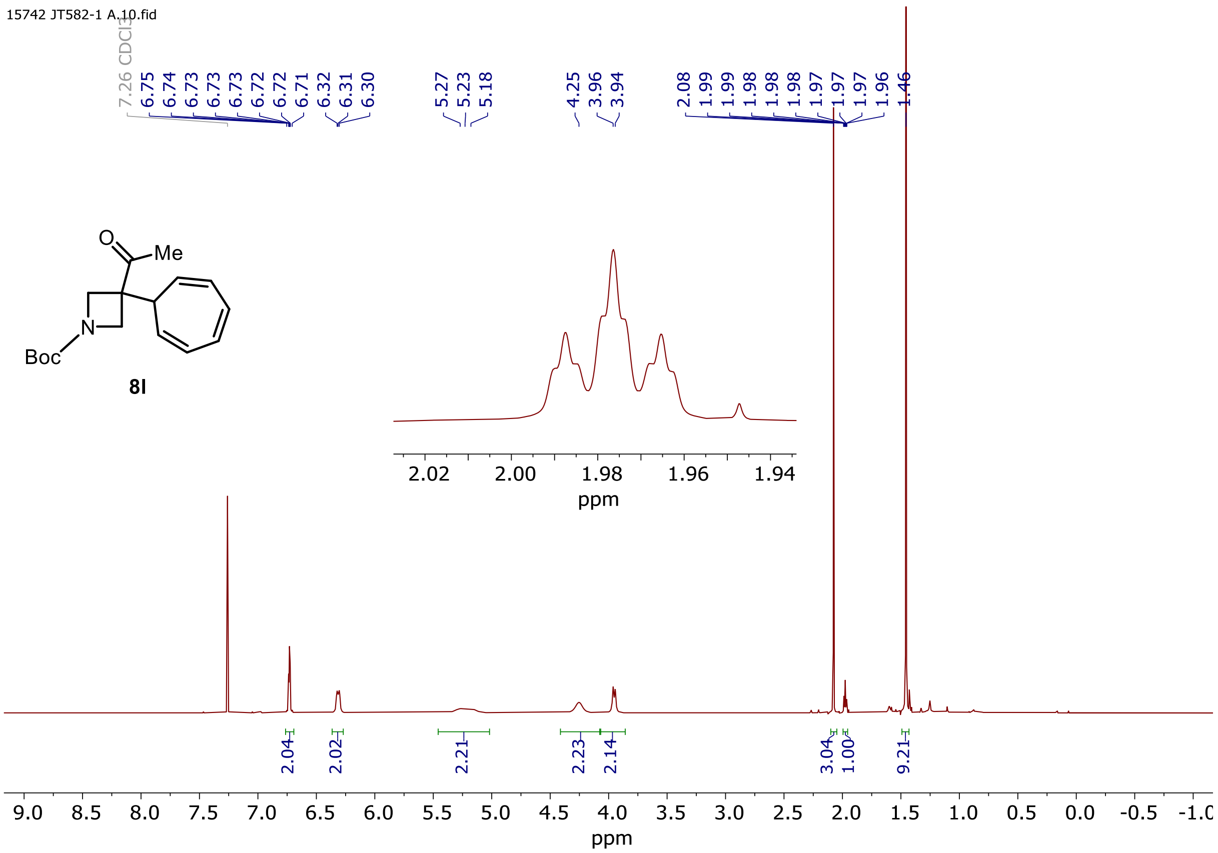

^{13}C NMR (126 MHz, $CDCl_3$) of **8l**

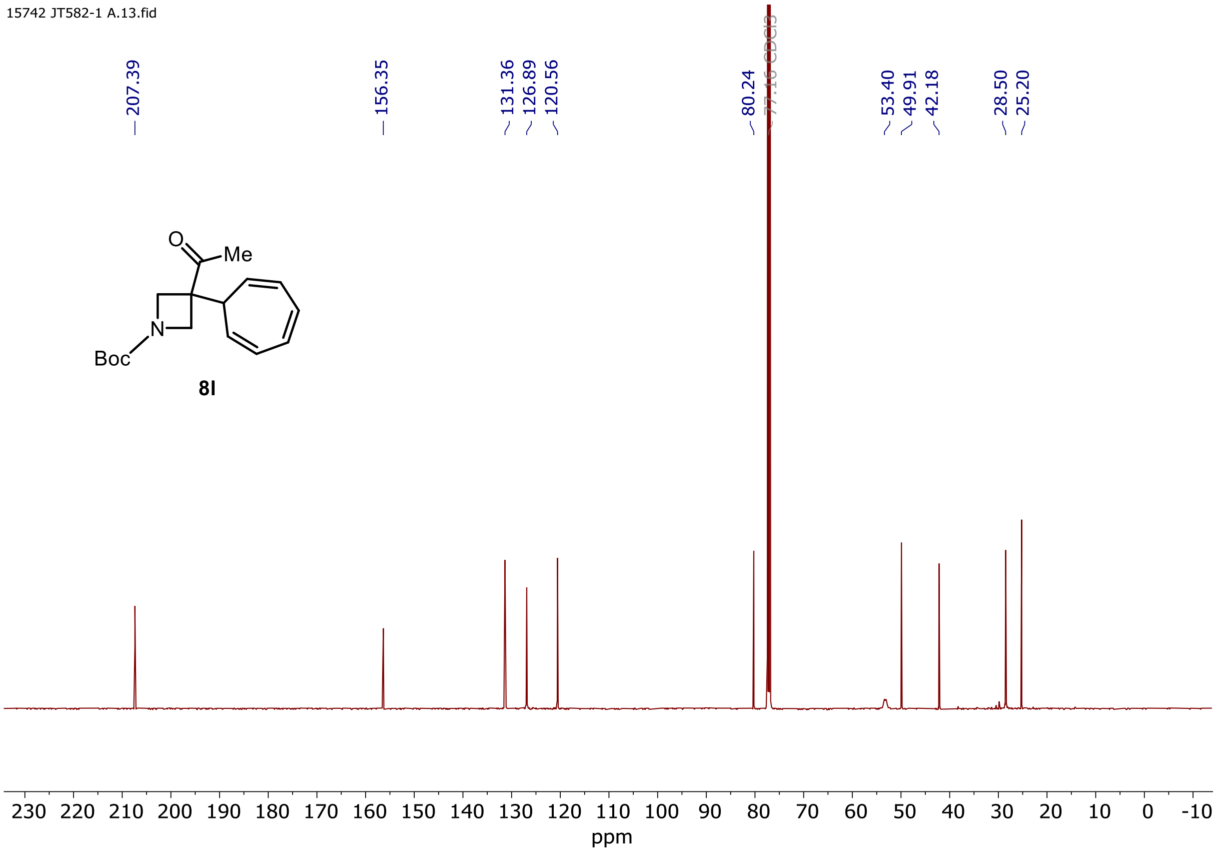

^{1}H NMR (400 MHz, $CDCl_3$) of **8m** (*see procedure*)

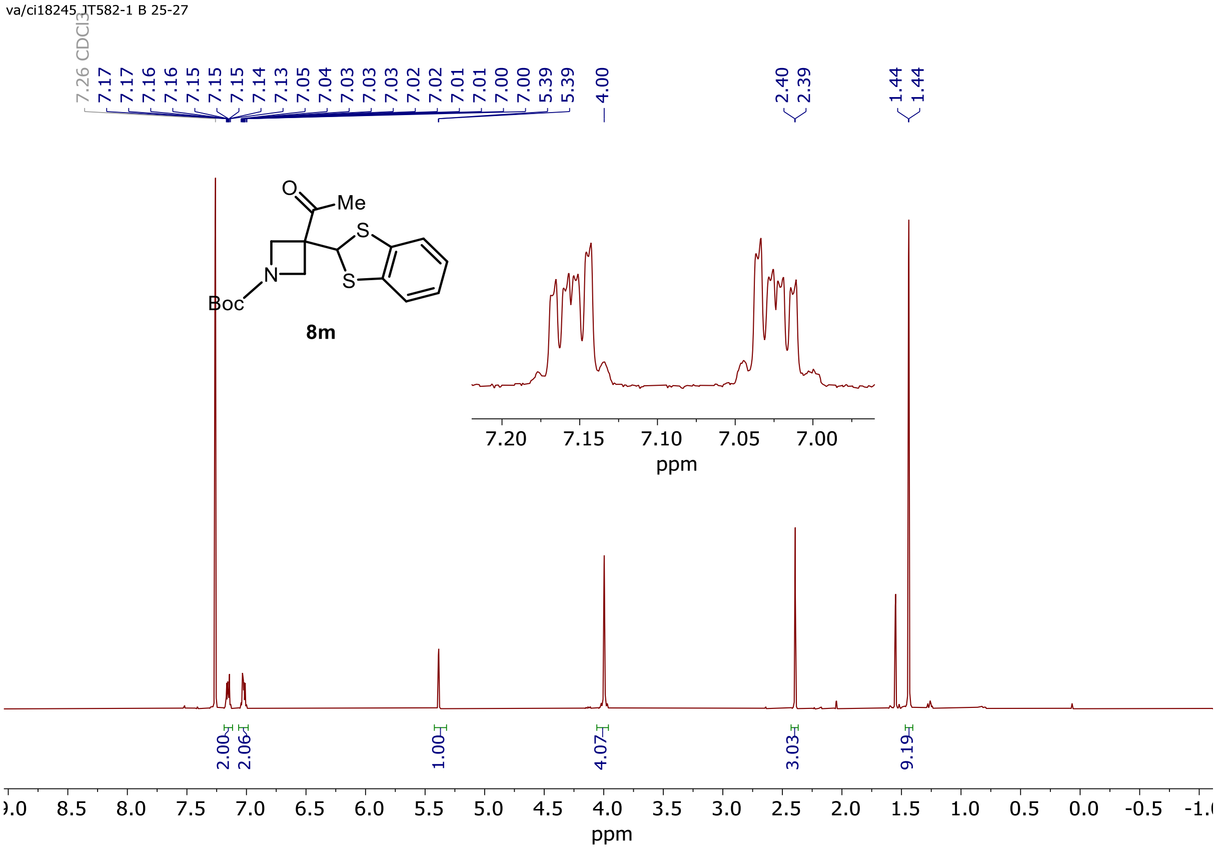

^{13}C NMR (126 MHz, $CDCl_3$) of **8m**

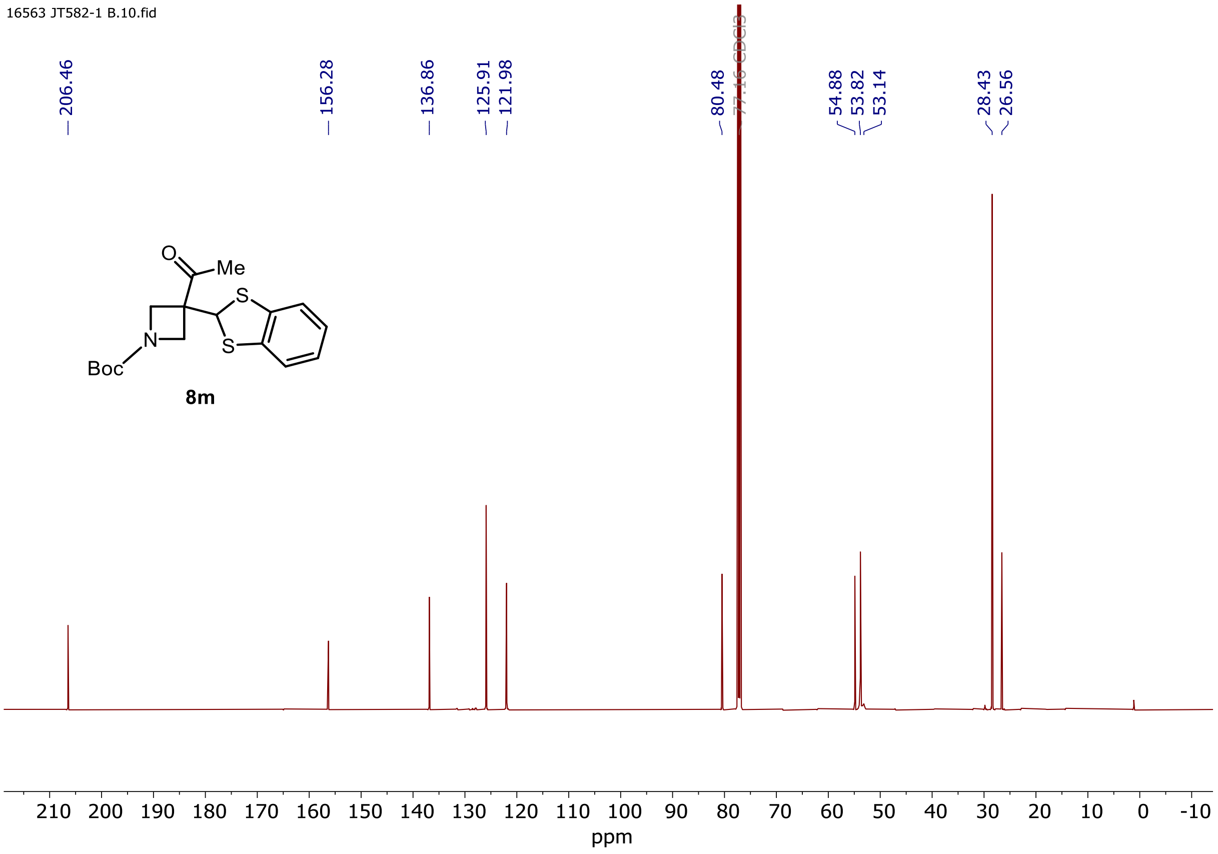

^{1}H NMR (500 MHz, $CDCl_3$) of **8n** (*see procedure*)

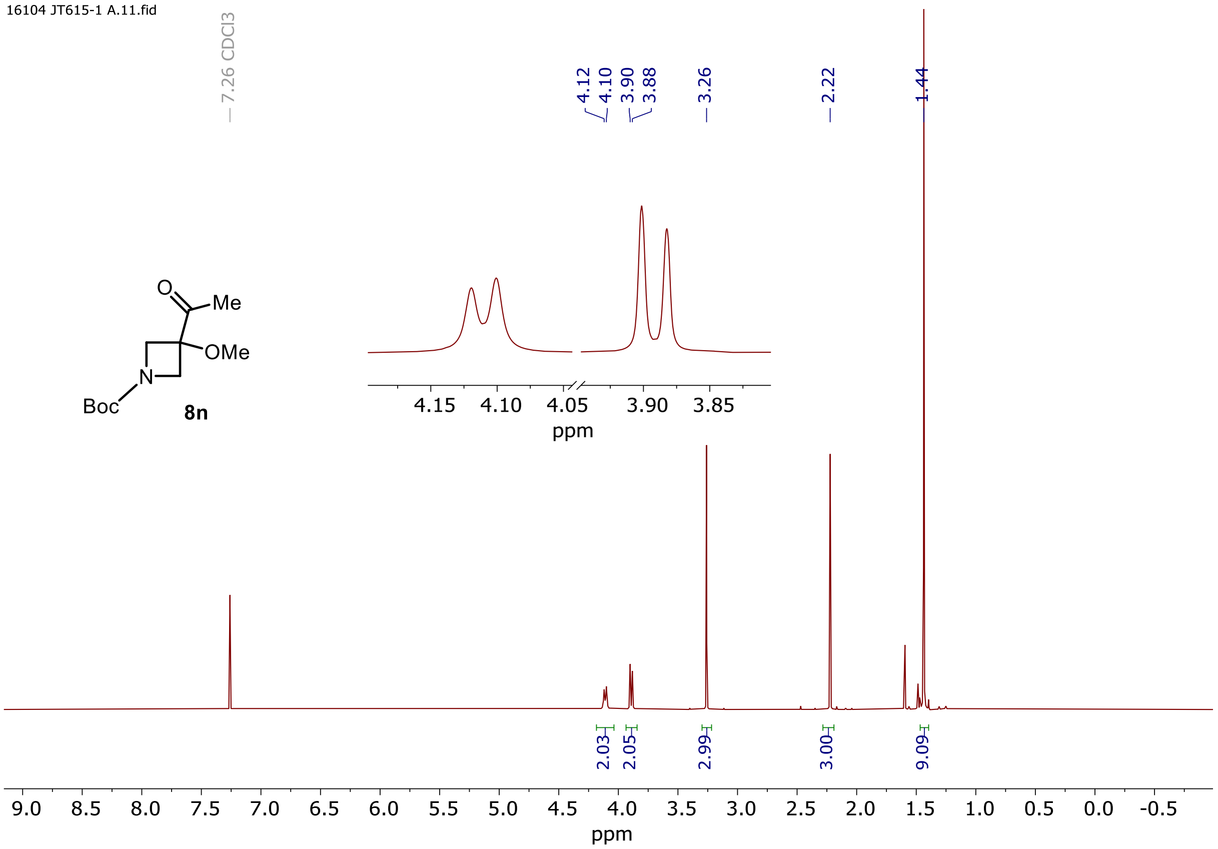

^{13}C NMR (126 MHz, $CDCl_3$) of **8n**

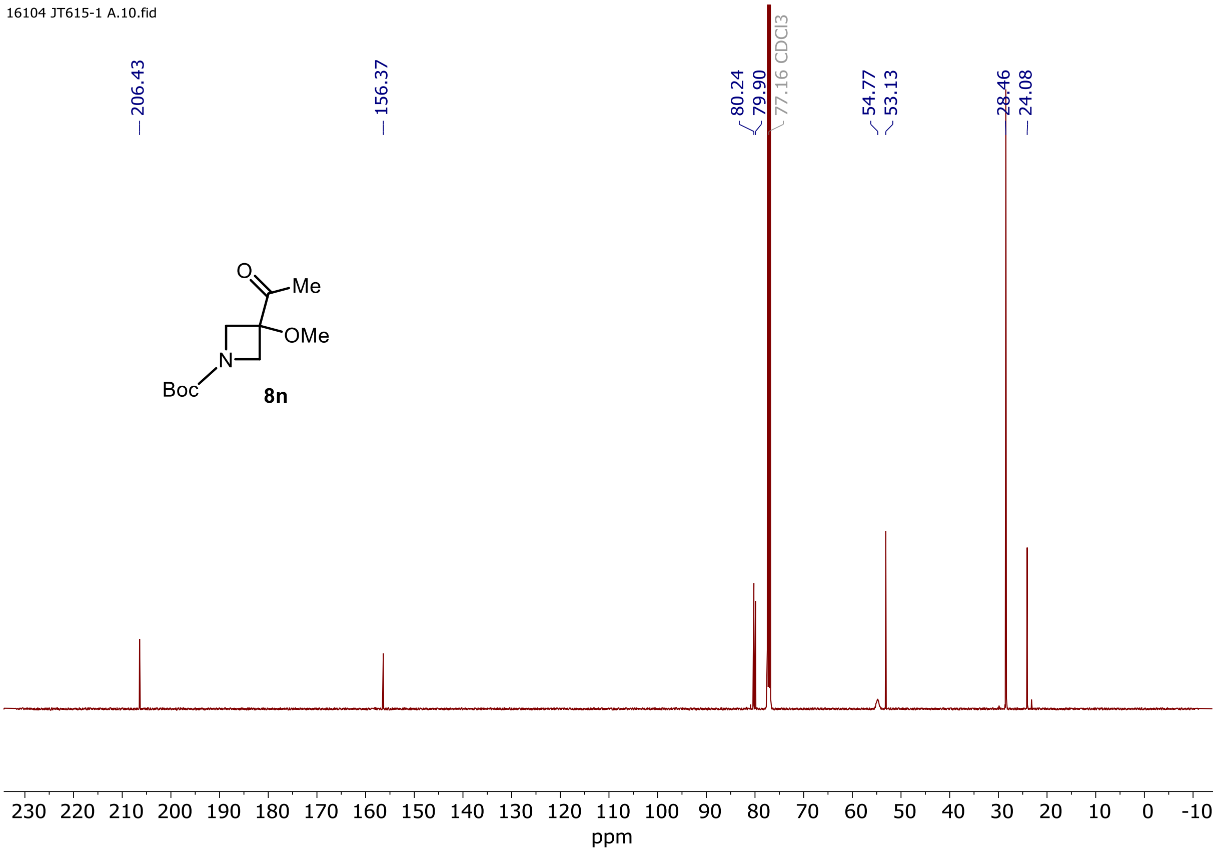

^{1}H NMR (500 MHz, $CDCl_3$) of **8o** (*see procedure*)

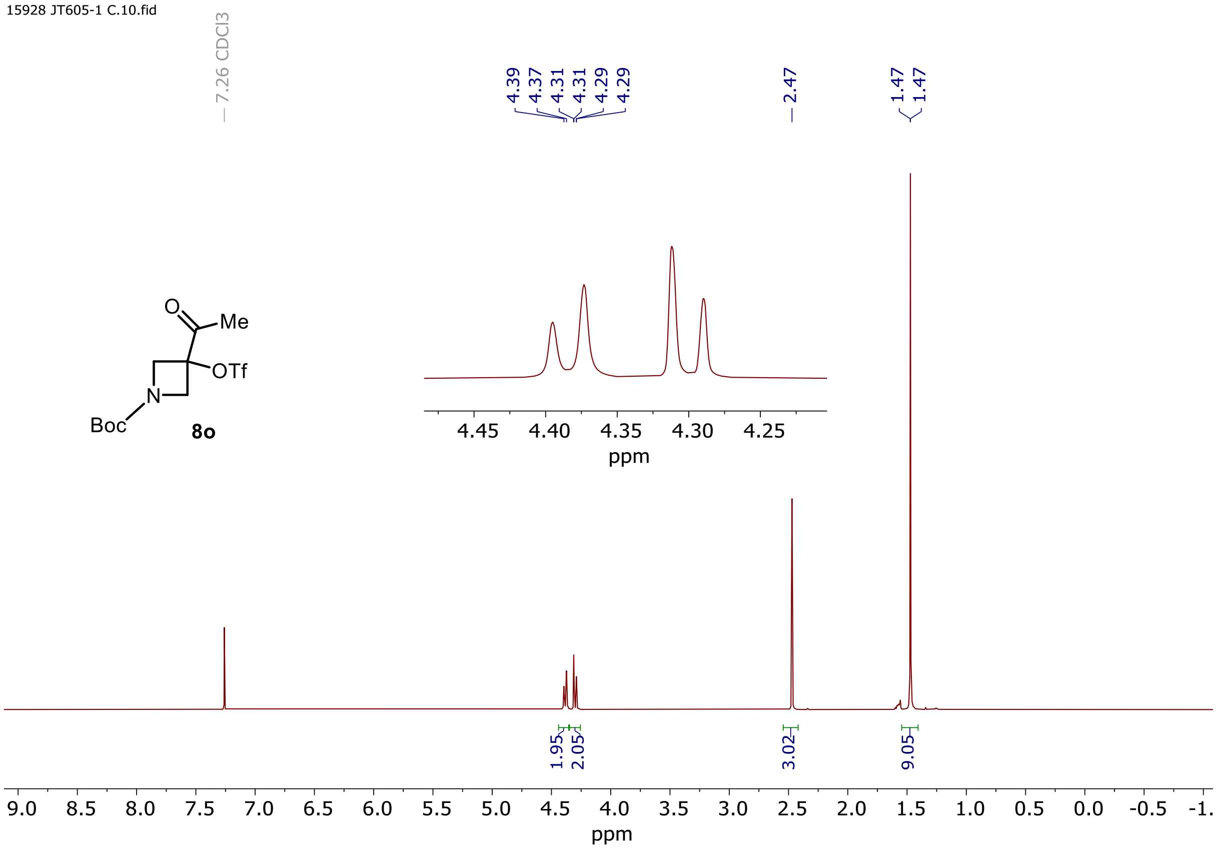

^{13}C NMR (126 MHz, $CDCl_3$) of **8o**

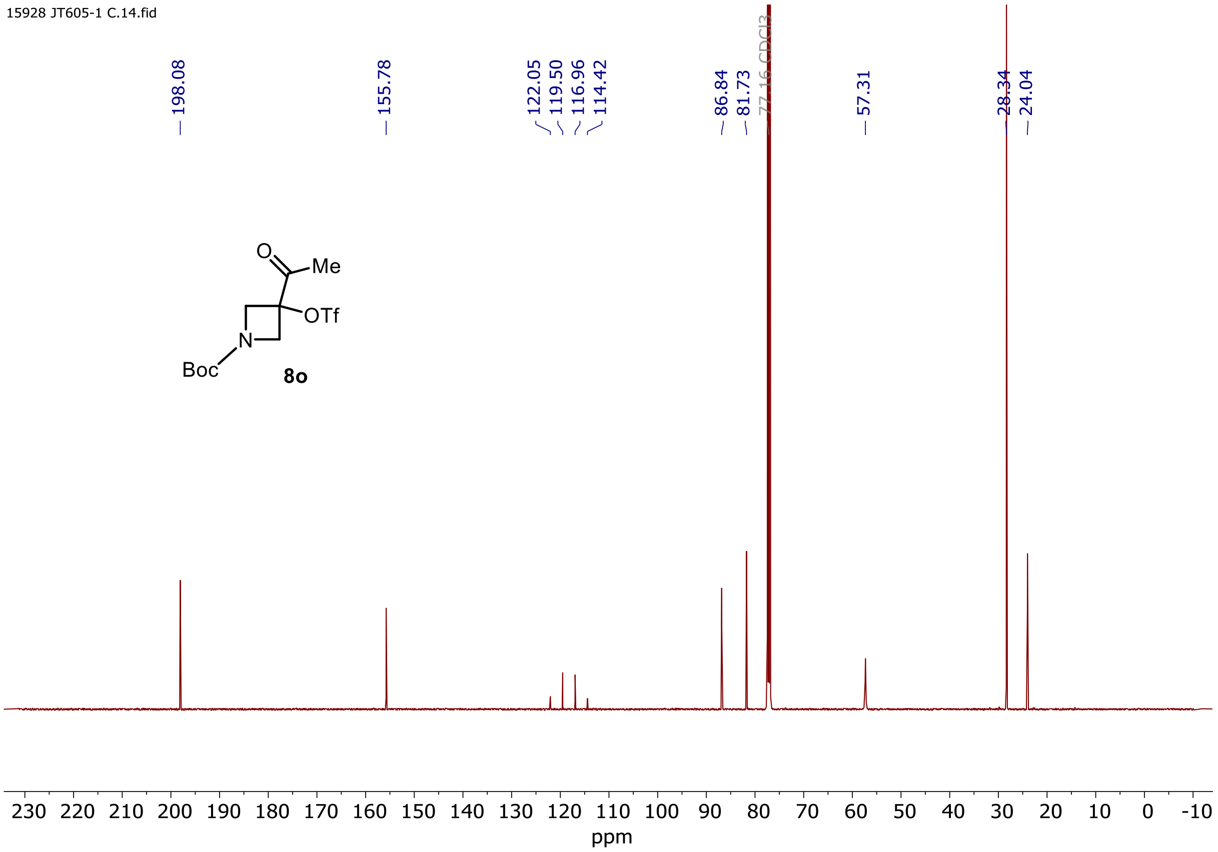

^{19}F NMR (376 MHz, $CDCl_3$) of **8o**

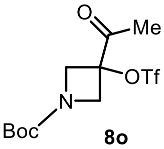

8o

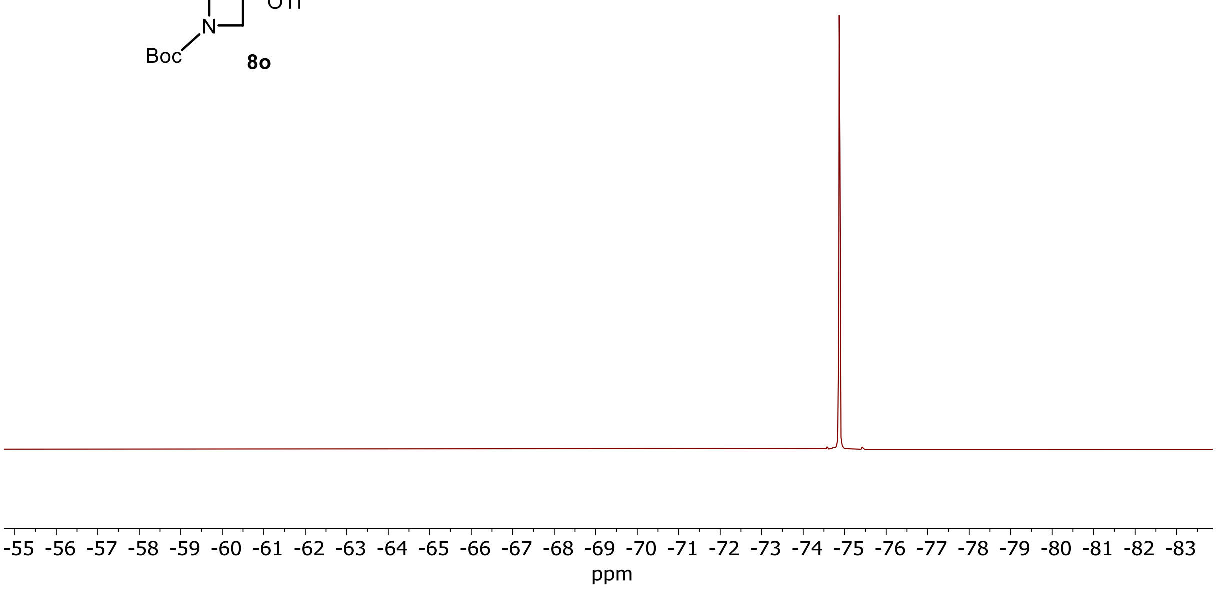

^{1}H NMR (400 MHz, $CDCl_3$) of **8p** (see procedure)

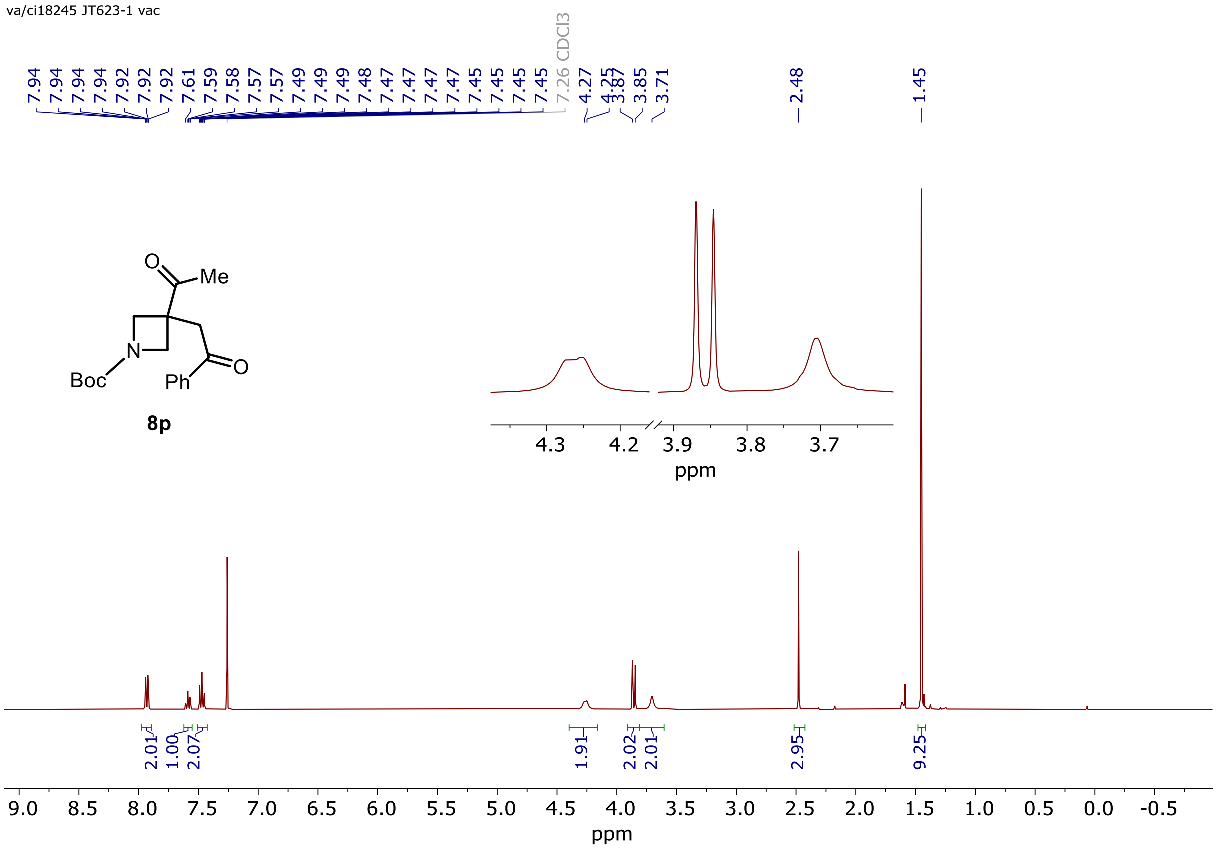

^{13}C NMR (126 MHz, $CDCl_3$) of **8p**

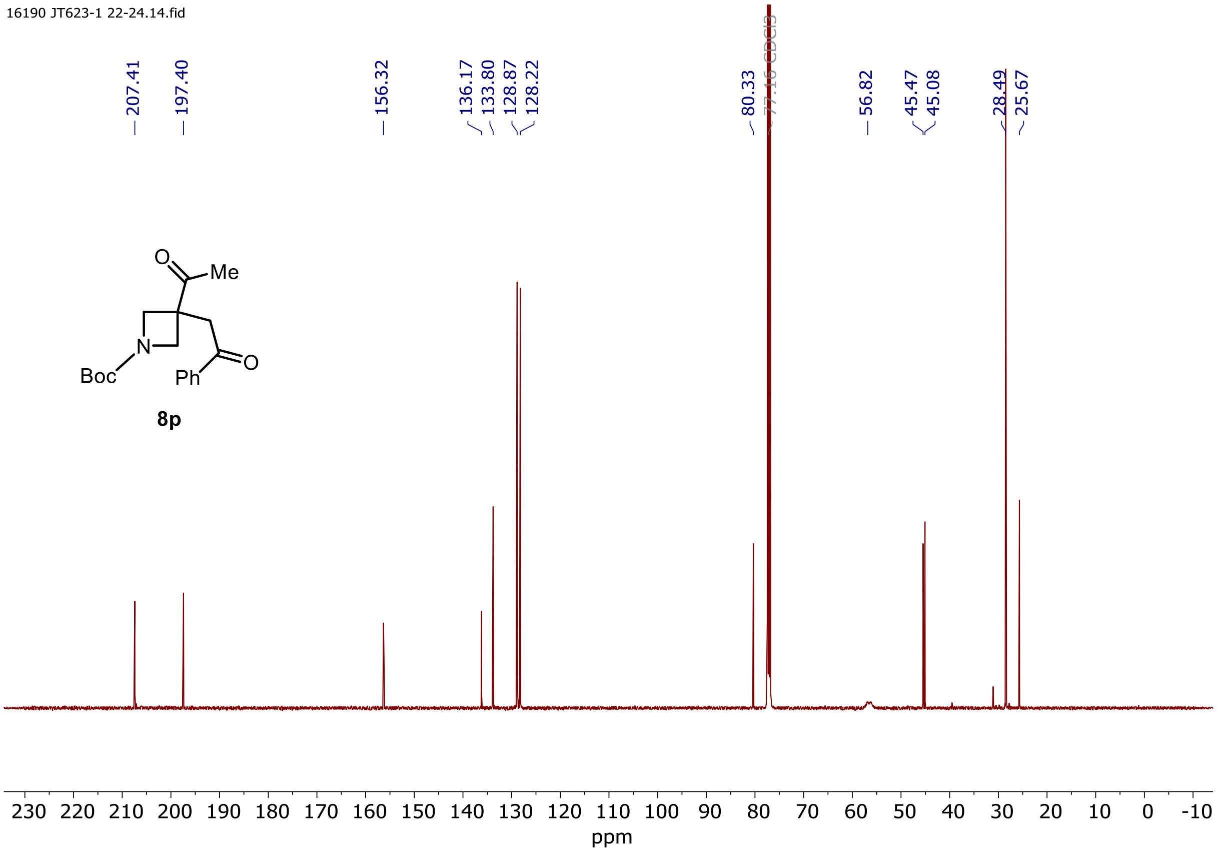

^{1}H NMR (500 MHz, $CDCl_3$) of **8q** (*see procedure*)

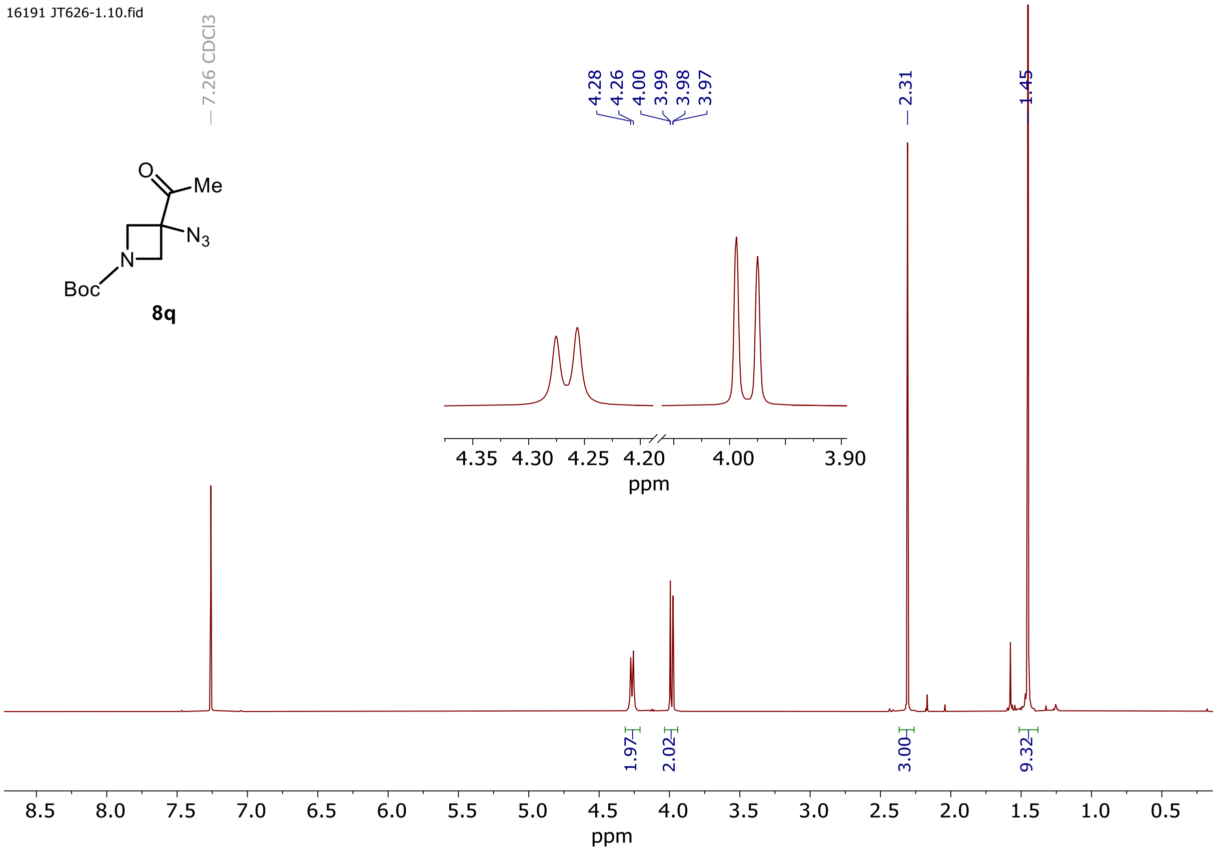

^{13}C NMR (126 MHz, $CDCl_3$) of **8q**

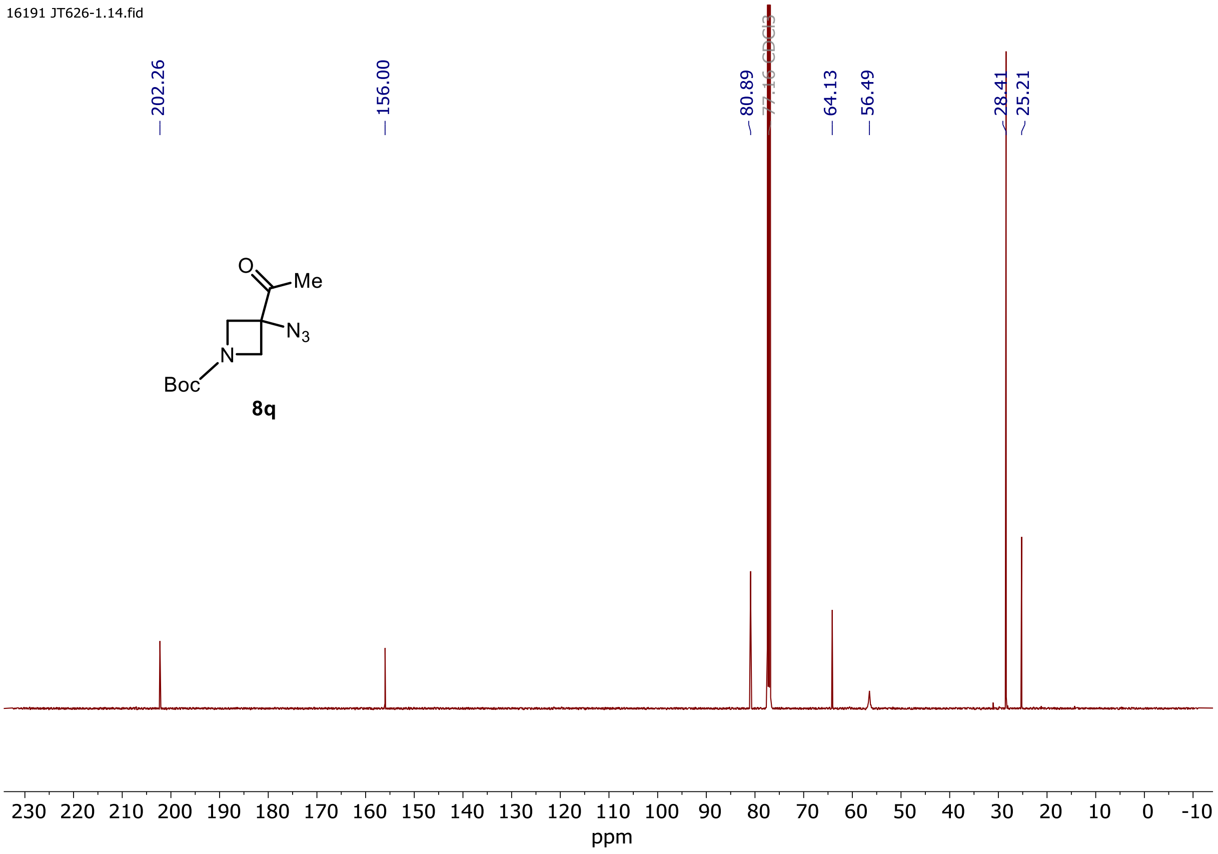

^{1}H NMR (500 MHz, $CDCl_3$) of **8r** (*see procedure*)

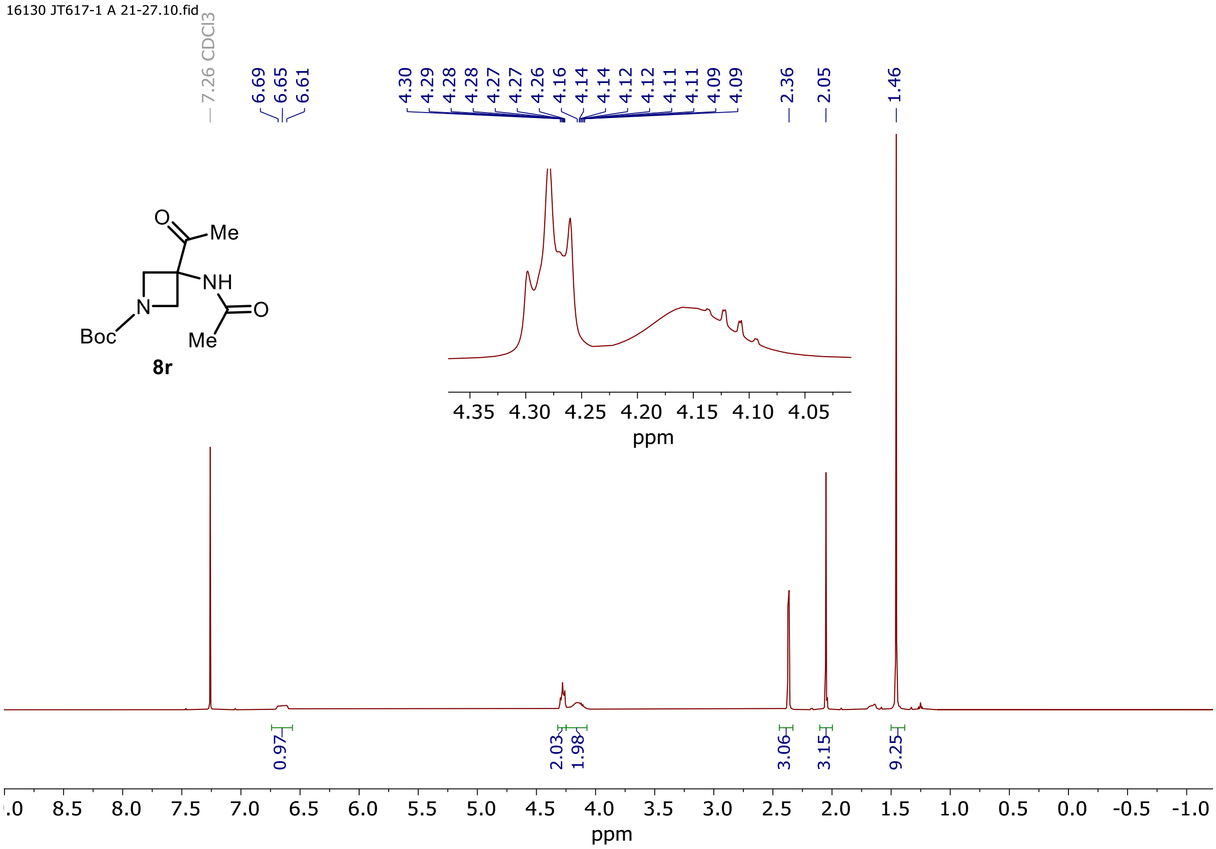

^{13}C NMR (126 MHz, $CDCl_3$) of **8r**

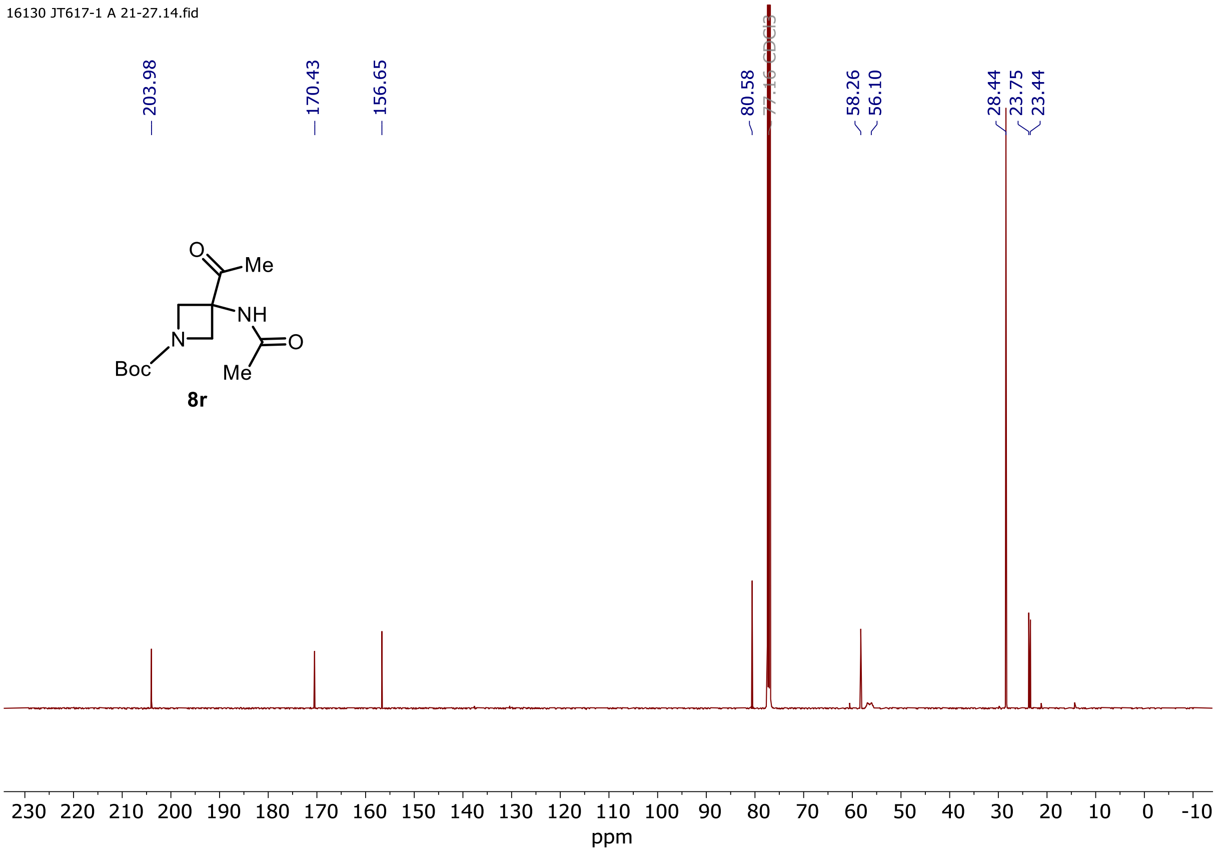

^{1}H NMR (400 MHz, $CDCl_3$) of **9** (*see procedure*)

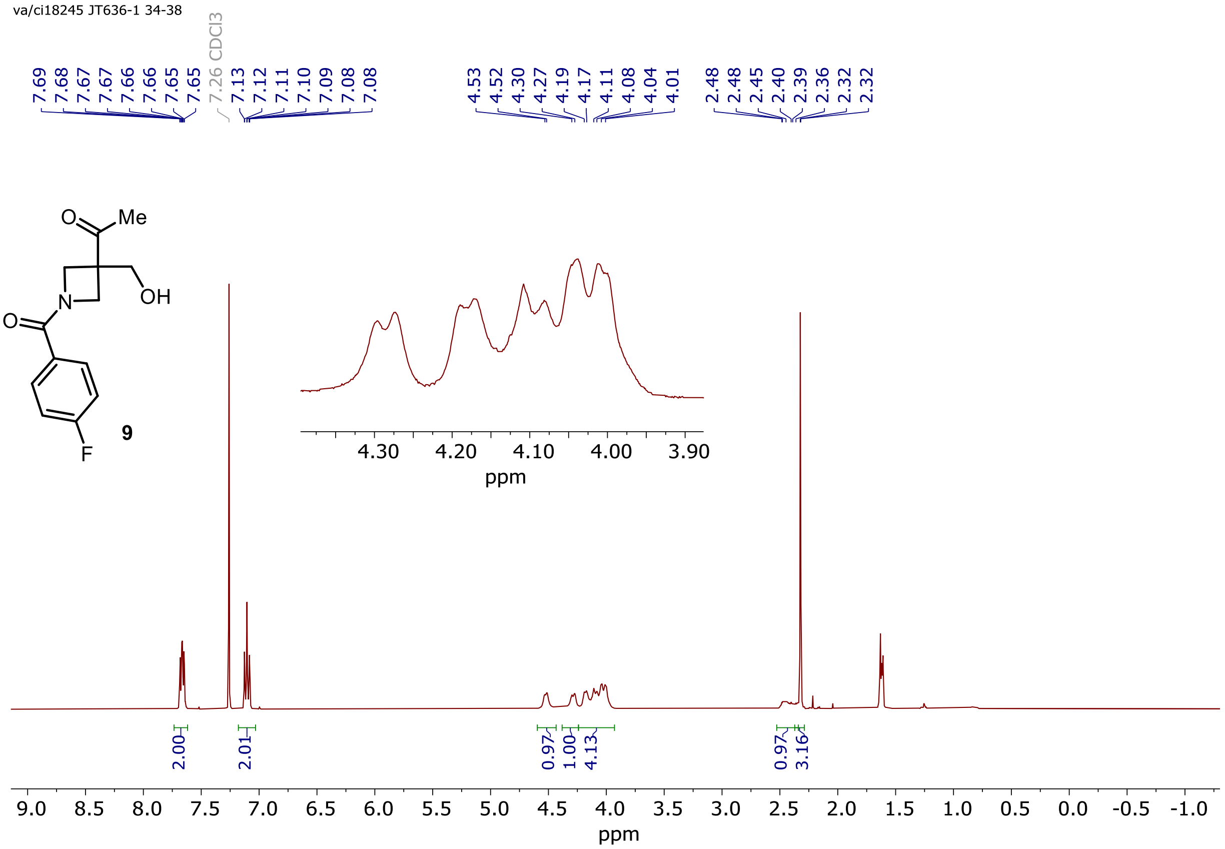

^{13}C NMR (126 MHz, $CDCl_3$) of **9**

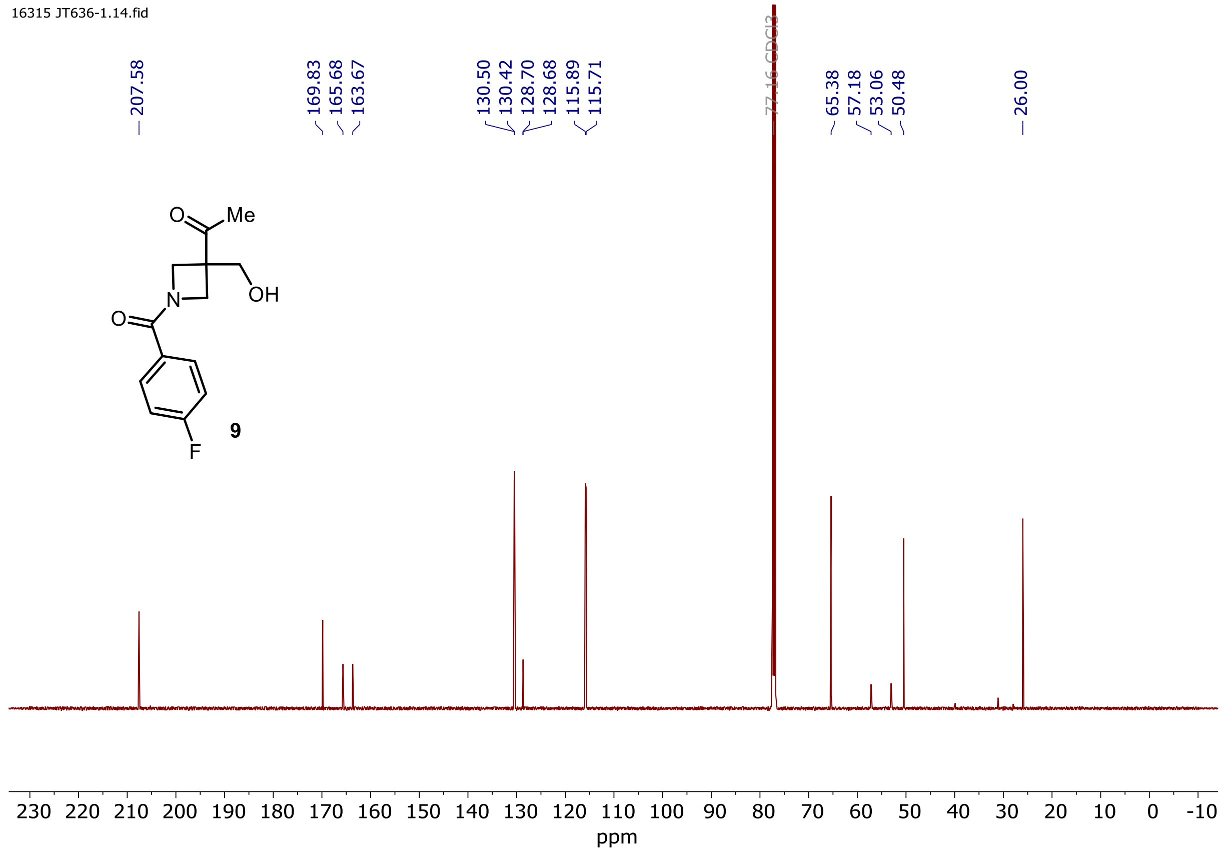

^{19}F NMR (376 MHz, $CDCl_3$) of **9**

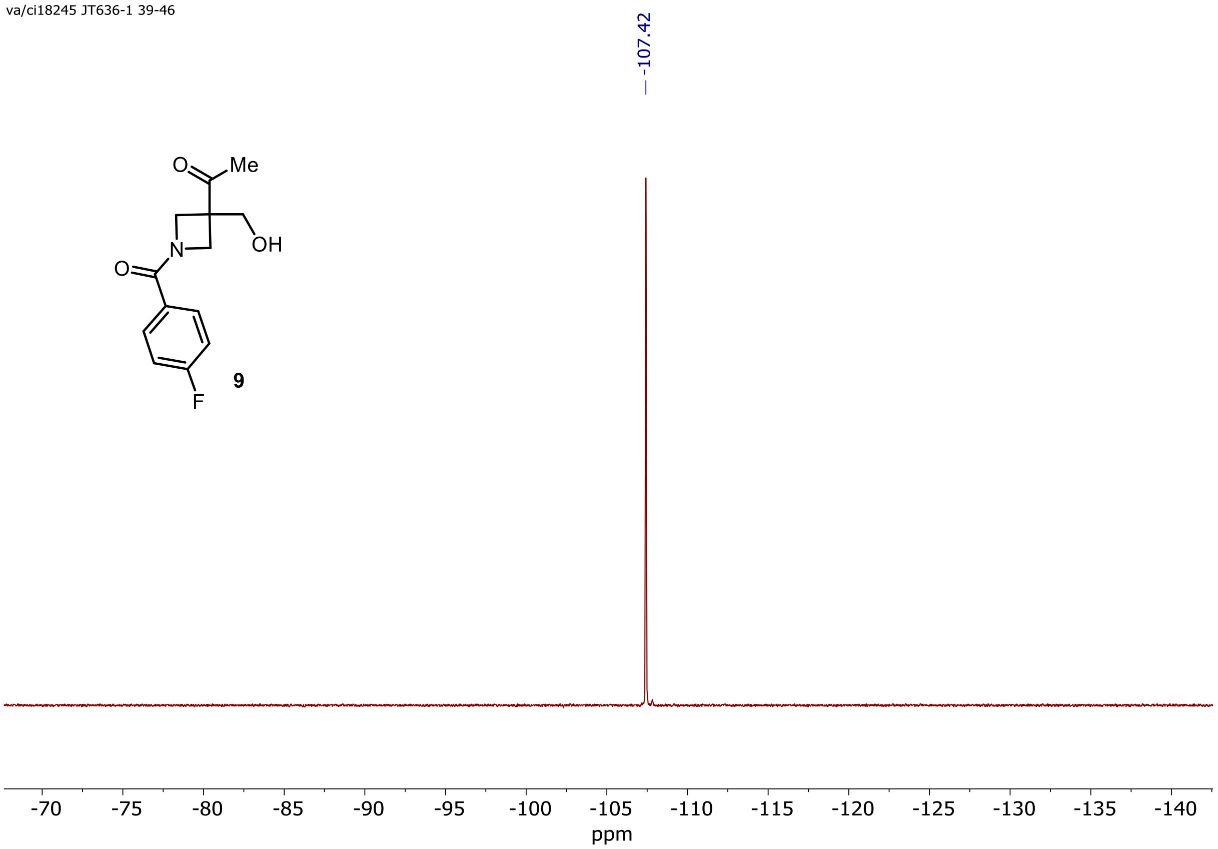

^{1}H NMR (400 MHz, CDCl$_3$) of **10** (*see procedure*)

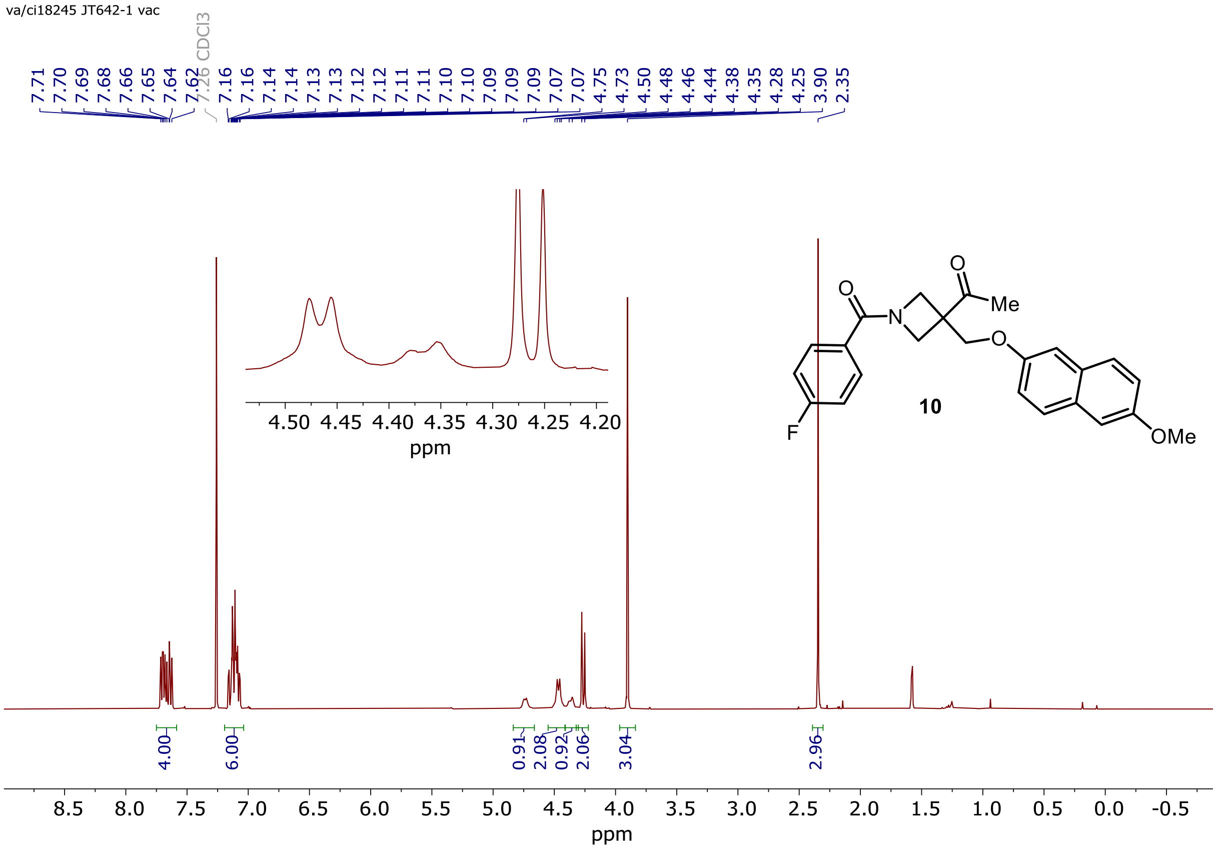

^{13}C NMR (126 MHz, CDCl$_3$) of **10**

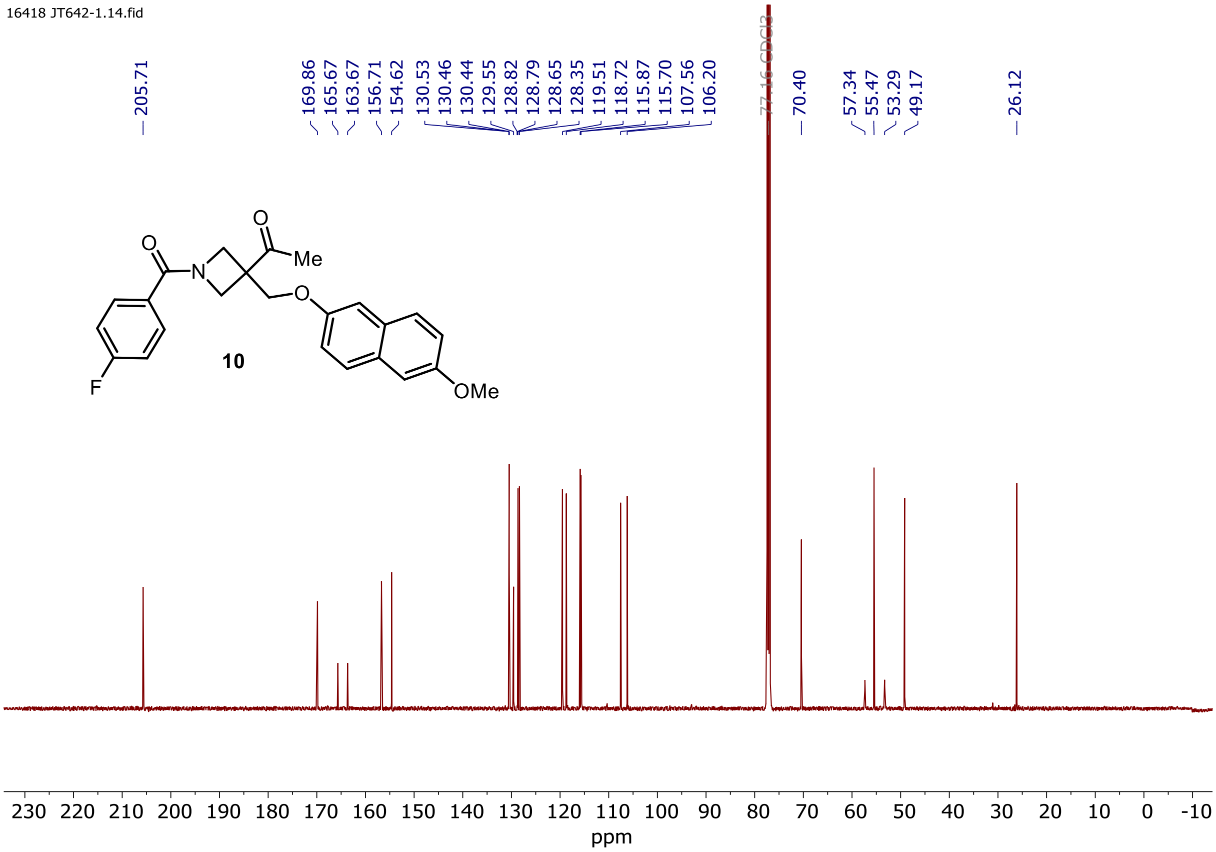

^{19}F NMR (376 MHz, $CDCl_3$) of **10**

va/ci18245 JT642-1 vac

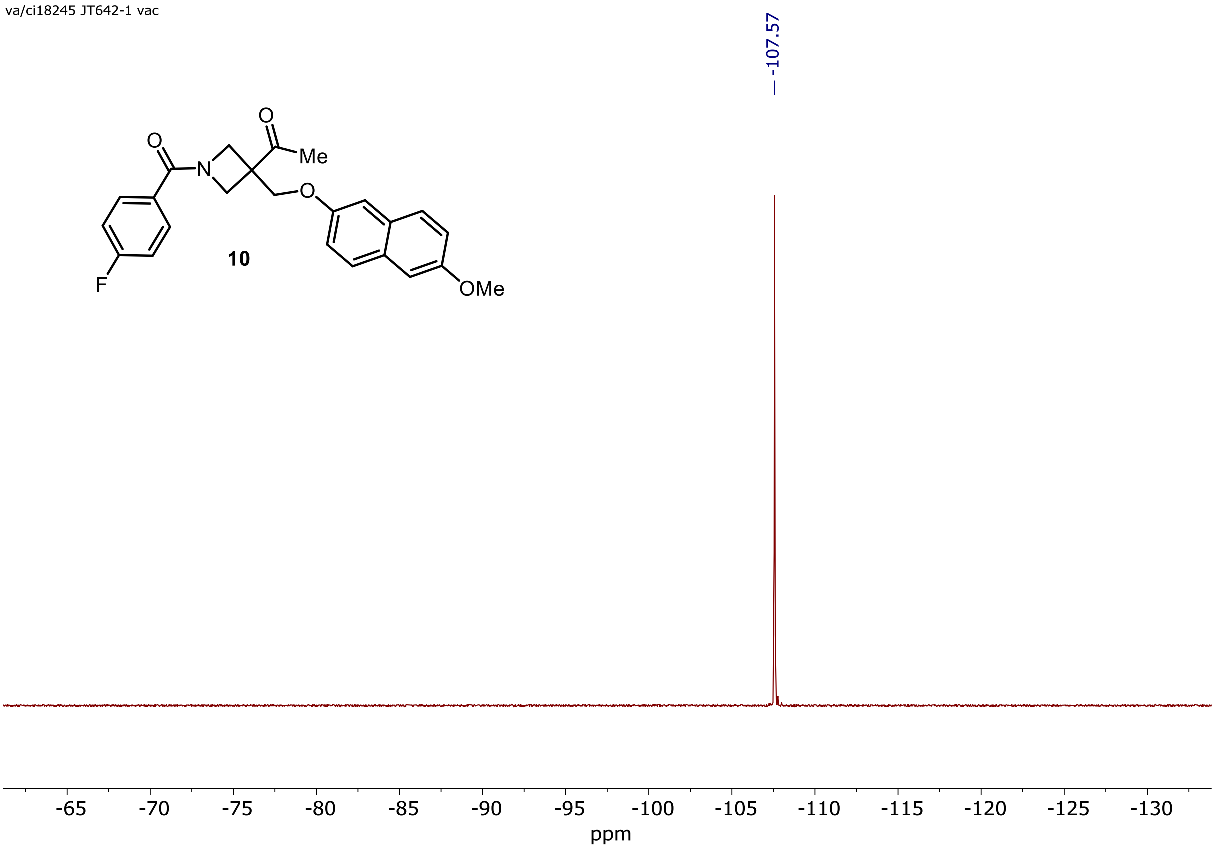

^{1}H NMR (400 MHz, $CDCl_3$) of **PF-04418948** (*see procedure*)

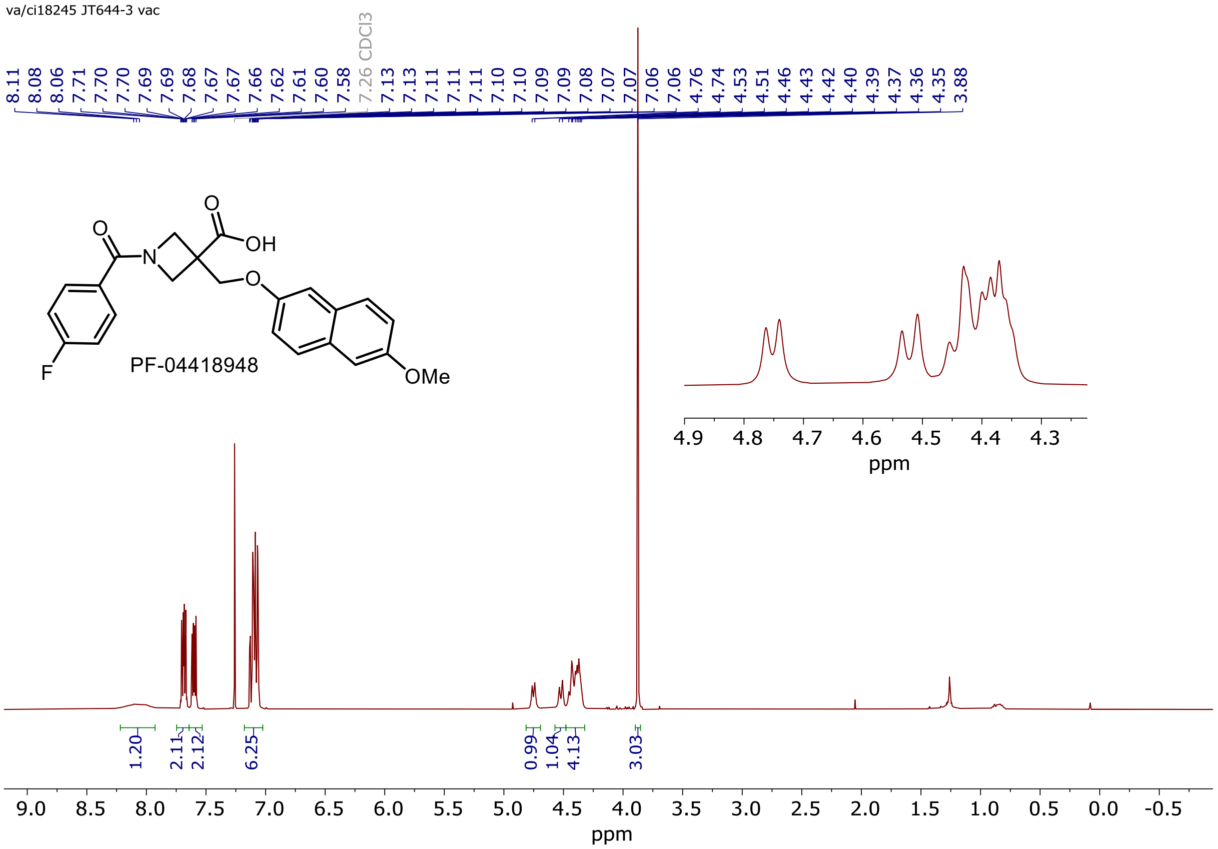

^{13}C NMR (126 MHz, $CDCl_3$) of **PF-04418948**

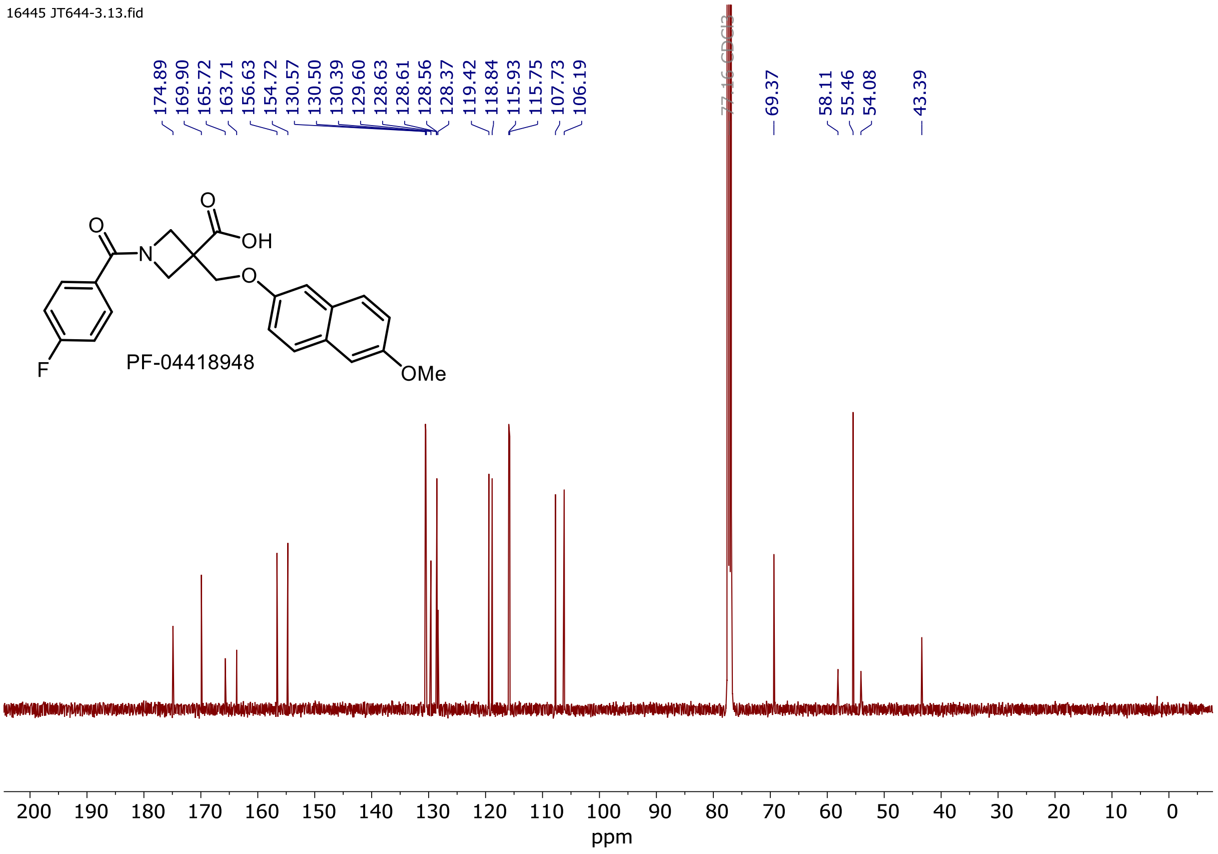

^{19}F NMR (376 MHz, $CDCl_3$) of **PF-04418948**

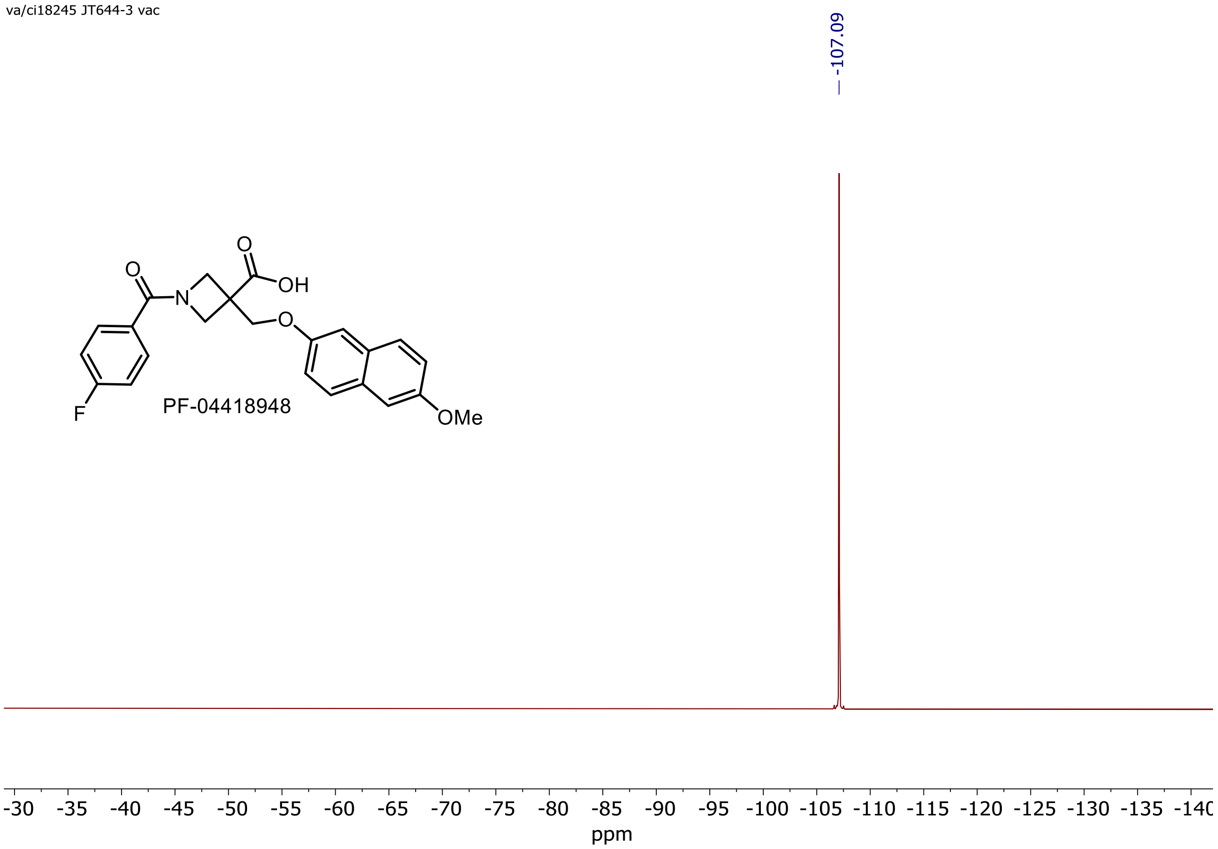

6. REFERENCES

1) (a) A. Fedinchyk, M. Herasymchuk, V. O. Smirnov, K. P. Melnykov, D. V. Yarmoliuk, A. A. Kyrylchuk, O. O. Grygorenko, *Eur. J. Org. Chem.* **2022**, e202200274. (b) K. Hattori, A. Yamada, S. Kuroda, T. Chiba, M. Murata, K. Sakane, *Bioorg. Med. Chem. Lett.* **2002**, *12*, 383–386.

2) C. Stuckhardt, M. Wissing, A. Studer *Angew. Chem. Int. Ed.* **2021**, *60*, 18605−18611.

3) J. L. Schwarz, R. Kleinmans, T. O. Paulisch, F. Glorius, *J. Am. Chem. Soc.* **2020**, *142*, 2168–2174.

4) P. Musci, T. von Keutz, F. Belaj, L. Degennaro, D. Cantillo, C. O. Kappe, R. Luisi, *Angew. Chem. Int. Ed.* **2021**, *60*, 6395−6399.

5) M. Ratushnyy, A. V. Zhukhovitskiy, *J. Am. Chem. Soc.* **2021**, *143*, 17931–17936.

6) X. Lu, J. Zhang, L. Xu, W. Shen, F. Yu, L. Ding, G. Zhong, *Org. Lett.* **2020**, *22*, 5610–5616.

7) T. A. Chappie, J. M. Humphrey, M. P. Allen, K. G. Estep, C. B. Fox, L. A. Lebel, S. Liras, E. S. Marr, F. S. Menniti, J. Pandit, C. J. Schmidt, M. Tu, R. D. Williams, F. V. Yang, *J. Med. Chem*. **2007**, *50*, 182–185.

8) G. R. Krow, G. Lin, S. B. Herzon, A. M. Thomas, K. P. Moore, Q. Huang, P. J. Carroll, *J. Org. Chem*. **2003**, *68*, 7562–7564.
